# Spy *vs.* spy: selecting the best reporter for ^19^F NMR competition experiments†
†Electronic supplementary information (ESI) available: Compound synthesis and characterization, experimental procedures for protein expression and biophysical experiments (raw data and fitting). See DOI: 10.1039/c8cc09790a


**DOI:** 10.1039/c8cc09790a

**Published:** 2019-01-15

**Authors:** Guilherme Vieira de Castro, Alessio Ciulli

**Affiliations:** a Division of Biological Chemistry and Drug Discovery , School of Life Sciences , University of Dundee , Dow Street , Dundee , DD1 5EH , UK . Email: a.ciulli@dundee.ac.uk

## Abstract

Systematic characterization of a series of fluorinated VHL ligands, varying binding affinity and position of the trifluoromethyl group, qualifies a spy molecule for competitive ^19^F NMR screening and reveals guiding principles to develop highly sensitive assays with low material consumption.

## Abstract 2

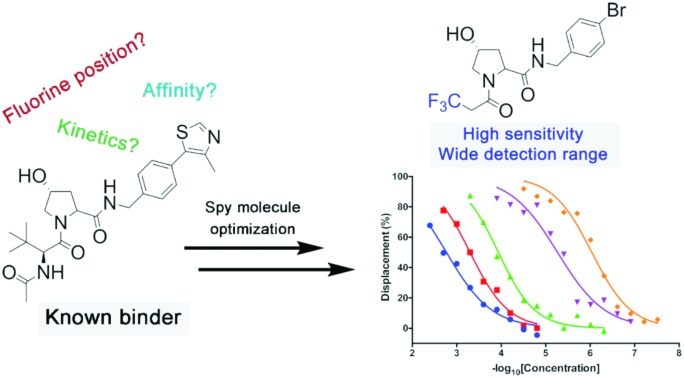
Characterization of a series of fluorinated compounds for competitive ^19^F NMR reveals the principles that can guide developing highly sensitive assays.

## 

Over the past few years, the application of nuclear magnetic resonance (NMR) to study small molecule interactions with biomolecular targets has increased. Both the large number of methods available and the ability to detect weak interactions imperceptible to other biophysical techniques make NMR a valuable resource for pharmaceutical research, from fragment-based drug discovery (FBDD) to lead optimization campaigns.^1^

A powerful approach to detect binders across a range of affinities consists in competition ligand-observed NMR experiments.^2^ Competitive ligands are detected by monitoring the signals of a known binder, also referred to as a reporter or spy molecule. When a competing ligand is present, the spy molecule will be displaced from its binding site. The increased relative population of free *vs.* bound state of the spy molecule can be detected by monitoring different NMR parameters and is dependent on the affinity of the competitor.^3^ If the binding affinity of the spy molecule is known, the affinity of the competitors can be estimated from the extent of the displacement.

In this context, the usage of fluorinated compounds as spy molecules presents yet further advantages. Fluorine atoms are absent in most common solvents, buffer components and biomolecules, resulting in simpler datasets to analyze when compared to proton-based methods. Furthermore, the high chemical shift anisotropy (CSA) of fluorine yields very clear responses to changes in the chemical environment, making the binding detection extremely sensitive.4*a* Despite many successful applications of ^19^F NMR competition experiments,^5^ the properties required to achieve sensitive spy molecules remain understudied. To date, spy molecule selection has consisted in either picking hits from the screening of a fluorinated compound library, or preparing fluorinated analogues of a known ligand in an unguided manner.^6^ Although aspects of spy molecule design have been discussed, including use of CF or CF_3_ groups, residence time, and fluorine local environment,^4^,^6^ the majority of these features are evaluated solely from a theoretical point of view. It thus remains unclear how much optimization is needed to obtain the best spy molecule. Here, a series of fluorinated ligands of a well-characterized ligand–protein binding system were developed and their potential as spy molecules was evaluated.

The von Hippel Lindau (VHL) E3 ligase was chosen as the target, because ligand binding to its hydroxyproline (Hyp) recognition site is deeply characterized.^7^ Many Hyp containing ligands are available with crystallographically characterized binding modes and a wide range of dissociation constants (*K*_D_). Moreover, the development of a ^19^F NMR competition assay for probing the Hyp site could see many valuable applications in the development of novel VHL ligands and VHL-based chemical degraders,^8^ and distinguishing Hyp site binders from those interacting with other sites.^7^,^9^

For spy molecule design, a trifluoromethyl (CF_3_) modification was preferred over, *e.g.*, fluoromethyl to generate a larger set of analogues (high availability of CF_3_ containing starting materials) and to yield increased signal to background. The potent binder VH032 (PDB: ; 4W9H)7*c* was used as a template in the design, and five positions were chosen for placing the CF_3_ modification (Fig. 1). These positions were selected by considering potential clashes with the protein, truncating left- and right-hand side groups to modulate the binding affinity. In total twenty-two compounds were synthesized by adapting previously described synthetic routes to prepare VHL ligands.^7^

**Fig. 1:**
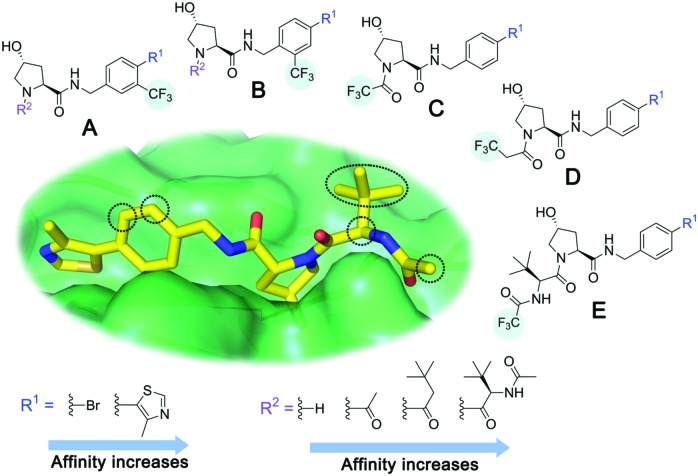
Design of a fluorinated spy molecule series. The co-crystal structure of VHL binder VH032 (PDB: 4W9H) inspired the choice of five positions (dashed circles) to attach a CF_3_ group, either on aromatic (series A and B) or aliphatic (C–E) regions.

Due to the large CSA of fluorine and the significant differences in the fluorine isotropic chemical shift (*δ*_F_) between the free and bound states, the transverse relaxation rate (*R*_2_) is a well-established sensitive parameter to detect ligand binding.^4^ Therefore, fluorine NMR experiments including a Carr–Purcell–Meiboom–Gill (CPMG) spin-echo filter^10^ before acquisition were employed to monitor ligand binding to VHL by measuring peak intensity, as changes in other NMR parameters, such as chemical shift and peak width, were not as significant. The *R*_2_ values of the CF_3_ peak of each compound in the absence and in the presence of protein were determined by performing multiple ^19^F CPMG experiments varying the CPMG filter (Fig. 2a). To quantify the shift in *R*_2_ upon binding, the *R*_2_ contrast (*C*_2_)^11^ was determined. Ideally, a good spy molecule should have a large increase in *R*_2_ (fast relaxation) when protein is present, resulting in a large assay window for the competition experiments (Fig. 2b).

**Fig. 2:**
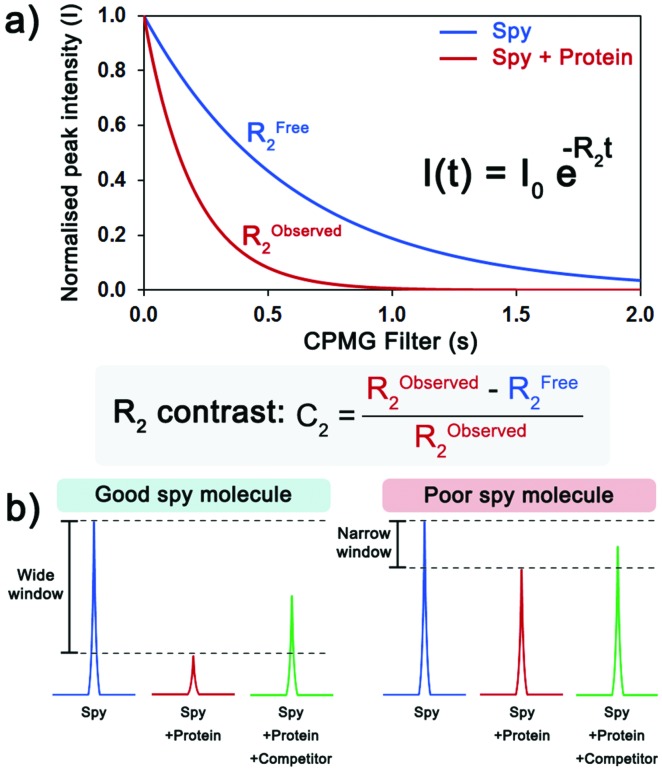
Evaluation of the sensitivity of a spy molecule. (a) Measurement of the transverse relaxation rate (*R*_2_) of the spy molecule in the absence (blue) and the presence of protein (red). The *C*_2_ quantifies the extent of the shift in *R*_2_ in the presence of protein. (b) Upon addition of protein, the ^19^F CPMG signal of a good spy molecule (left) is greatly reduced, resulting in a large window to detect and rank displacers, while for a poor spy molecule (right), the difference between the signals is too small.

The *K*_D_ of each compound to VHL was determined by surface plasmon resonance (SPR) experiments. The *K*_D_ of the majority of the compounds could be measured confidently (Table 1), with the exception of cases where the binding responses were too low (*K*_D_ > 1.5 mM) or the responses were above the theoretical maximum response (promiscuous or unspecific binders).

**Table 1:** Characterisation of spy molecules by SPR and ^19^F NMR

Series	Compound	R^1^	R^2^	*K*_D, SPR_ (μM)	*C*_2_ ^*a*^ (%)
A	**1**	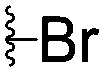	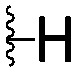	>1500	4.1 ± 3.1
	**2**	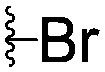	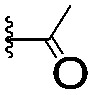	657 ± 49^*b*^	6.5 ± 2.2
	**3**	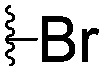	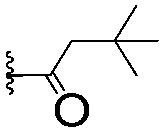	407 ± 5	28.2 ± 2.3
	**4**	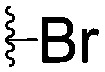	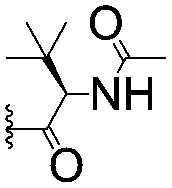	4.39 ± 0.12	38.9 ± 2.5
	**5**	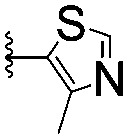	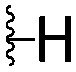	>1500	2.2 ± 1.8
	**6**	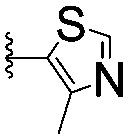	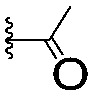	878 ± 98^*b*^	37.7 ± 2.6
	**7**	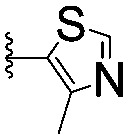	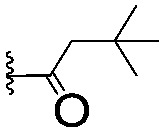	67.1 ± 14.0	62.1 ± 3.0
	**8**	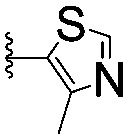	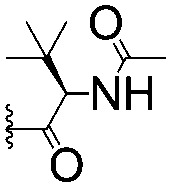	2.99 ± 0.75	17.7 ± 2.2
B	**9**	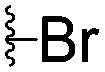	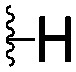	>1500	0.9 ± 2.7
	**10**	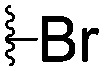	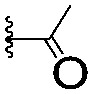	1352 ± 94 ^*b*^	51.9 ± 2.1
	**11**	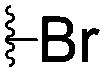	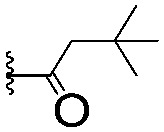	110 ± 9	47.9 ± 4.1
	**12**	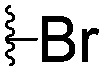	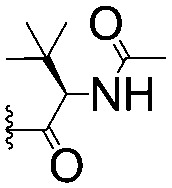	1.14 ± 0.12	7.6 ± 2.4
	**13**	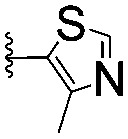	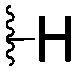	^*c*^	3.2 ± 2.0
	**14**	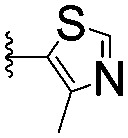	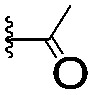	^*c*^	53.9 ± 2.5
	**15**	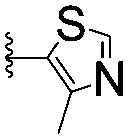	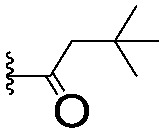	35.2 ± 7.0	41.3 ± 2.7
	**16**	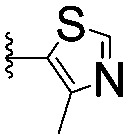	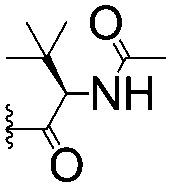	0.268 ± 0.030	1.3 ± 2.4
C	**17**	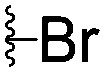	—	645 ± 100^*b*^	62.0 ± 2.6
	**18**	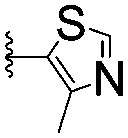	—	24.8 ± 3.4	76.0 ± 3.2
D	**19**	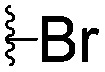	—	145 ± 29	70.1 ± 4.5
	**20**	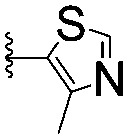	—	12.4 ± 1.9	48.4 ± 5.6
E	**21**	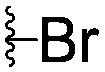	—	0.447 ± 0.085	3.7 ± 8.8
	**22**	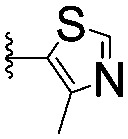	—	0.0969 ± 0.0068	0.8 ± 8.6

^*a*^Conditions: spy molecule at 100 μM in the absence or presence of protein at 1 μM.

^*b*^Intermediate-weak binders, *K*_D_ obtained by fitting the data with a 1-to-1 binding model as the maximum response (*R*_MAX_) could not be obtained experimentally.

^*c*^High responses in the SPR experiments, promiscuous or unspecific binders.

A number of trends can be observed. The weakest binders of the series (**1**, **5** and **9**) were among the spy molecules with the lowest *C*_2_. In this case the concentration of the spy–protein complex was much lower than *K*_D_, so too small a percentage of the molecules contributed to the signal, resulting in a low *C*_2_ value. On the other hand, as expected the tightest binders of the series (**12**, **16**, **21** and **22**) also presented a very low *C*_2_. This is due to their higher residence time in the binding site, not allowing many molecules to interact with the protein during the time of the NMR experiment. Notably, despite the high affinities, the dissociation rates of these compounds were too high to be determined accurately by SPR, showing that only remarkably fast binding kinetics can yield a sensitive spy molecule at low protein concentrations.

By plotting *C*_2_*versus* p*K*_D_ (Fig. 3a), two binding affinity limits where the spy molecule sensitivity decays could be observed, only molecules of p*K*_D_ = 3.0–5.5 presented good values of *C*_2_. To understand the effect of the position of the CF_3_ group, we plotted *C*_2_*versus* p*K*_D_ for ligands within the series A (Fig. 3b) and B (Fig. 3c). In both cases the data are distributed as a bell-shaped curve, whereby ideal spy molecules should be weak enough to possess very fast kinetics, but not too weak, so the amount of spy–protein complex is sufficiently populated to allow for detecting overall changes in relaxation. This observation agrees with the theoretical prediction by Dalvit *et al.* varying the residence time of the fluorinated ligand.4*b*

**Fig. 3:**
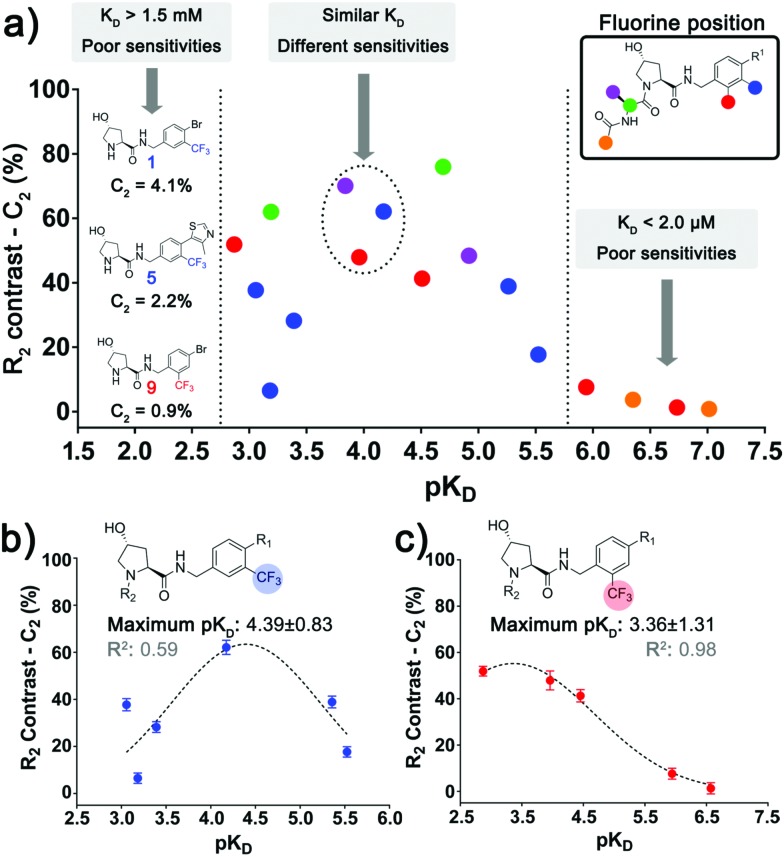
Relationship between sensitivity, affinity and position of the fluorinated group. (a) Correlation between *C*_2_ and the respective p*K*_D_ of all the spy molecules where the affinity could be estimated, highlighting the regions where the sensitivity of the spy molecules decays considerably. Dashed circles display molecules with similar p*K*_D_ that possess different *C*_2_ due to the variation in the fluorine attachment. The same plot was also made for the aromatic series A (b) and B (c), where the trend of an ideal intermediate affinity can be observed.

Interestingly, the best affinity range varied across the two series, with optimal p*K*_D_ ≈ 4.4 (*K*_D_ ≈ 40 μM) for series A and p*K*_D_ ≈ 3.4 (*K*_D_ 400 μM) for series B. This result could be explained by differences in association rates (*k*_on_) or Δ*δ*_F_ (bound and free states) of equivalent compounds between the series, leading to different sensitivities at the same *K*_D_. This observation shows that not only the absolute affinity, but also the chemical environment surrounding the fluorine atom in the context of the bound ligand affected the sensitivity of the spy molecules. In further support of the importance of the fluorine position, **7**, **11** and **19** (Fig. 3a, dashed circle) all had similar affinities (p*K*_D_ ≈ 3.8–4.2) but different *C*_2_ due to varying CF_3_ attachments.

The most sensitive spy molecules (Table 1, highest *C*_2_) were **7**, **17**, **18** and **19**. Although molecule **18** presented the overall highest sensitivity, molecule **19** was selected for setting the competition assay. As **19** is seven times weaker than **18**, it was hypothesized that **19** would be more readily displaced by weak competitor fragments, allowing a wider range of *K*_D_ detection. Molecule **19** was also preferred over **17** because its affinity could be determined more accurately by SPR (ESI,† Fig. S1).

Spy molecule **19** consistently displayed good sensitivity under different conditions. When the concentrations of spy and protein were simultaneously lowered, a binding response could still be observed even at the lowest concentrations (**19** at 5 μM, VHL at 125 nM), albeit with lower signal-to-noise ratio (S/N) and *C*_2_, requiring longer experiment times (Fig. 4a). Most conditions displayed a reasonable *C*_2_ (≥40%), allowing the detection of competitors even with low material consumption and number of scans (4 minutes per sample). In any case, conditions with *C*_2_ as high as possible should be aimed for whenever possible.

**Fig. 4:**
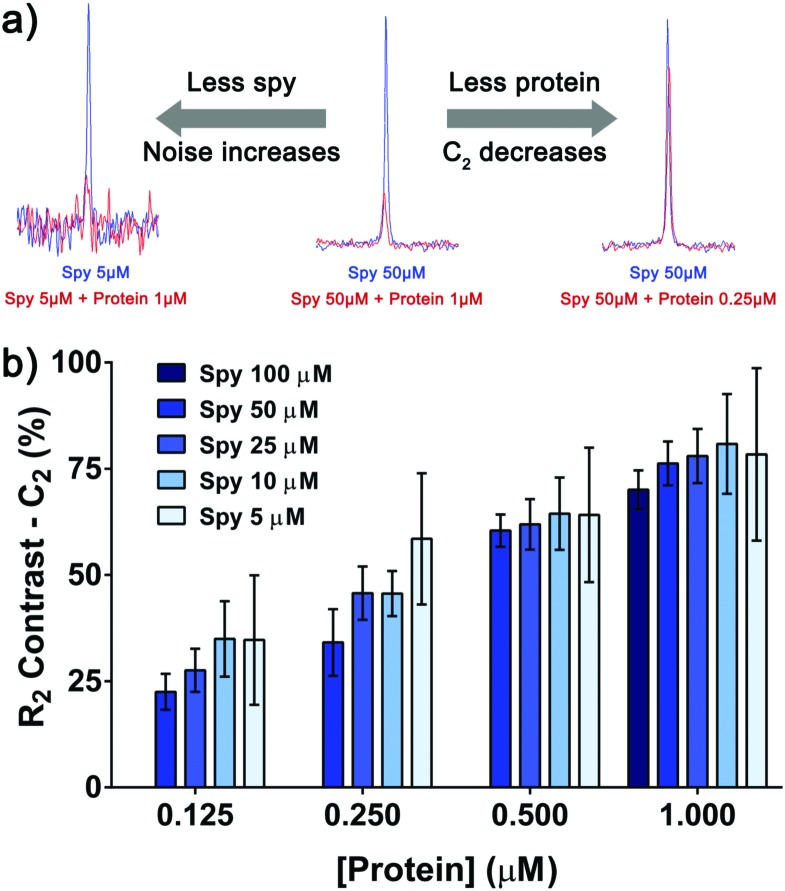
Binding response can be observed at low concentrations of spy molecule **19** and protein. (a) Overlay of the ^19^F CPMG peak (200 ms filter) of spy molecule **19** in the absence (blue) and presence of protein (red). When lowering the concentrations of spy molecule and protein, the S/N and *C*_2_ values respectively decreased. (b) Measurement of the *C*_2_ of spy molecule **19** at different concentrations (in triplicates), in the presence of four concentrations of protein. The error progressively increases as the concentration of spy molecule goes down, due to the increase in noise of the NMR data.

Noticeably, the *C*_2_ values were affected to a great extent by the total amount of protein rather than the total amount of spy molecule (Fig. 4b). At 1.0 μM of protein the *C*_2_ values varied between 70 and 80% for all the concentrations of spy tested, even if they were around 1.5–15 times lower than the *K*_D_. In this way, for selecting the most sensitive spy molecule of a series, a single screening at fixed concentration of protein is adequate to determine the best compounds to set up the competition assay, even if affinities vary significantly and sub-*K*_D_ concentrations are used.

Lastly, the utility and scope of using spy molecule **19** to quantify binding of competitors across a wide range of affinities was evaluated (Fig. 5). Five compounds were titrated against **19** at 50 μM and VHL at 1.0 μM. In all cases the displacement of the spy molecule was concentration-dependent and the *K*_i_ (inhibition constant) values were derived from the respective IC_50_ using a competitive binding model.^12^ The measured binding affinities compared remarkably well with the respective values obtained by SPR across 4-log units. The same experiment was attempted with spy molecules **6** and **11** under the same conditions, and in these cases, the assay could not differentiate the binders as efficiently (ESI,† Fig. S2–S4), since this would require higher protein concentration. Binders of other pockets present in the VHL E3 ligase previously reported did not displace spy molecule **19** as expected, showing that the displacement is site-specific (ESI,† Fig. S5).

**Fig. 5:**
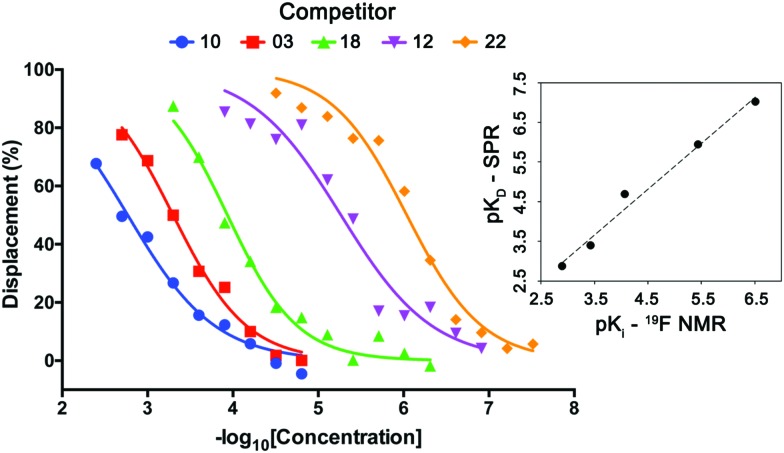
Determination of the affinities of VHL binders using spy molecule **19**. Displacement of spy molecule **19** in the presence of different concentrations of five VHL binders (molecules **3**, **10**, **12**, **18** and **22**) was concentration dependent. By knowing the concentrations of spy molecule (50 μM), and protein (1 μM) and the *K*_D_ between spy molecule **19** and VHL, the *K*_i_ of each competitor was determined, having good correlation with the respective *K*_D_ values obtained by SPR (top right inset graph).

In summary, we qualify a broad-scope high-quality spy molecule for the E3 ligase VHL able to differentiate a wide range of *K*_D_ values with good correlation with orthogonal techniques. This advance will aid future screening efforts for novel VHL ligands and chemical degraders. More broadly, this work provides a blueprint for obtaining the most sensitive reporter for a given protein–ligand system, while minimizing protein and spy molecule consumption, and argues for exploring chemical space with fluorine at different positions of a binding ligand, especially in the early stages of projects where only weak binders might be available. This guidance will prove useful to many groups in academia and industry that develop NMR assays for FBDD or hit optimization campaigns.

This project has received funding from the European Research Council (ERC) under the European Union's Seventh Frame-work Programme (FP7/2007–2013) as a Starting Grant to A. C. (grant agreement no. ERC-2012-StG-311460 DrugE3CRLs) and the Coordenação de Aperfeiçoamento de Pessoal de Nível Superior (CAPES, PhD Studentship 7148-14-3 to G. V. C.). Biophysics and drug discovery activities were supported by Wellcome Trust strategic awards to Dundee (100476/Z/12/Z and 094090/Z/10/Z, respectively). We thank D. Fletcher and A. Bortoluzzi for support with NMR and helpful discussions.

## Conflicts of interest

The Ciulli laboratory receives sponsored research support from Boehringer Ingelheim and Nurix, Inc. A. C. is a scientific founder, director and shareholder of Amphista Therapeutics, which is developing targeted protein degradation platforms.

## Supplementary Material

Supplementary information
