# Supplementary material for: Spy vs. spy: selecting the best reporter for 19F NMR competition experiments
Source: Chem Commun (Camb). 2019 Jan 15;55(10):1482–5. doi: 10.1039/c8cc09790a (PMC6369734; doi:10.1039/c8cc09790a)
Supplement: Supplementary file 1 [file CC-055-C8CC09790A-s001.pdf]

Electronic Supplementary Material (ESI) for Chemical Communications.
This journal is © The Royal Society of Chemistry 2019

SUPPORTING INFORMATION

Spy vs. Spy: Selecting the best reporter for ^{19}F NMR competition experiments

Guilherme V. Castro and Alessio Ciulli

Division of Biological Chemistry and Drug Discovery, School of Life Sciences, University of Dundee, Dow Street, Dundee, DD1 5EH, Scotland, United Kingdom

Contents

1. Supplementary figures 2
2. Compound synthesis and characterization 5
3. Protein expression, purification and biotinylation 21
4. Surface plasmon resonance experiments 22
5. Measurement of the transverse relaxation rates by ^{19}F CPMG NMR 23
6. Competition experiments by ^{19}F NMR 24
7. Surface plasmon resonance – Sensorgrams and data fitting 26
8. ^{19}F CPMG signal intensity versus CPMG filter 48
   8.1 Spy molecules at 100 µM in absence or in presence of VBC 1 µM 48
   8.2 Spy molecule **19** and VBC at different concentrations 59
   8.3 Spy molecules **6** and **11** at 50 µM in absence or in presence of VBC 1 µM 61
9. NMR spectra of synthesized compounds 62
10. References 147

1. Supplementary figures

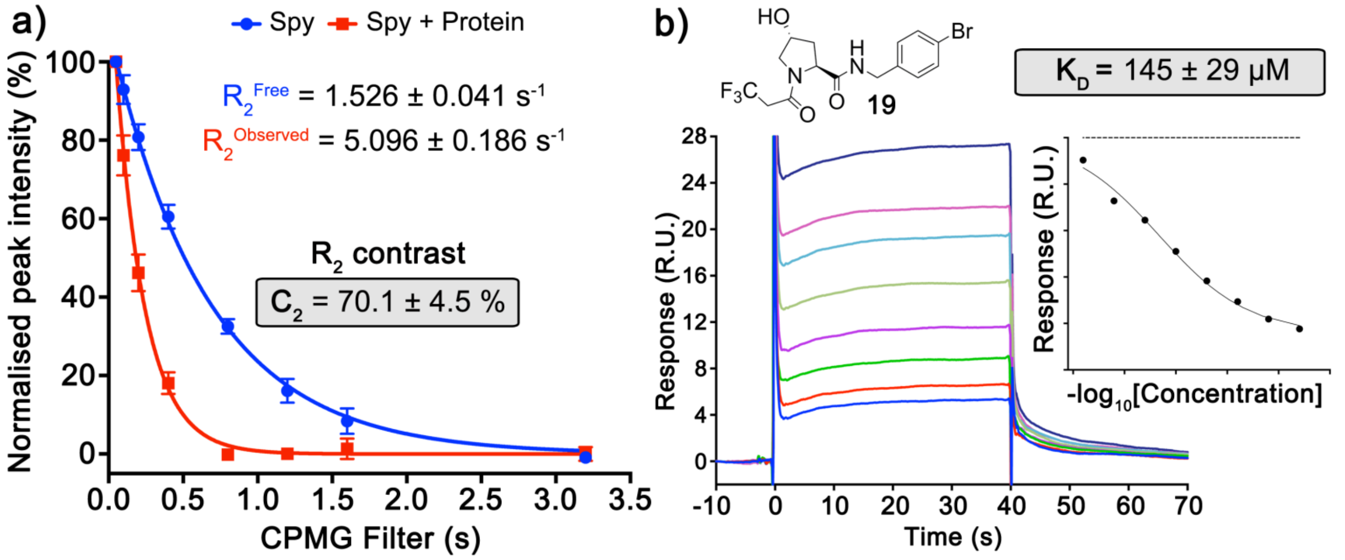

Supplementary Figure 1. Biophysical characterization of spy molecule 19 by ^{19}F NMR and SPR. (a) Measurement of the transverse relaxation rate (R_2) of **19** at 100 µM in absence (blue) and in presence (red) of protein at 1 µM. **(b)** Measurement of the K_D of **19** by SPR.

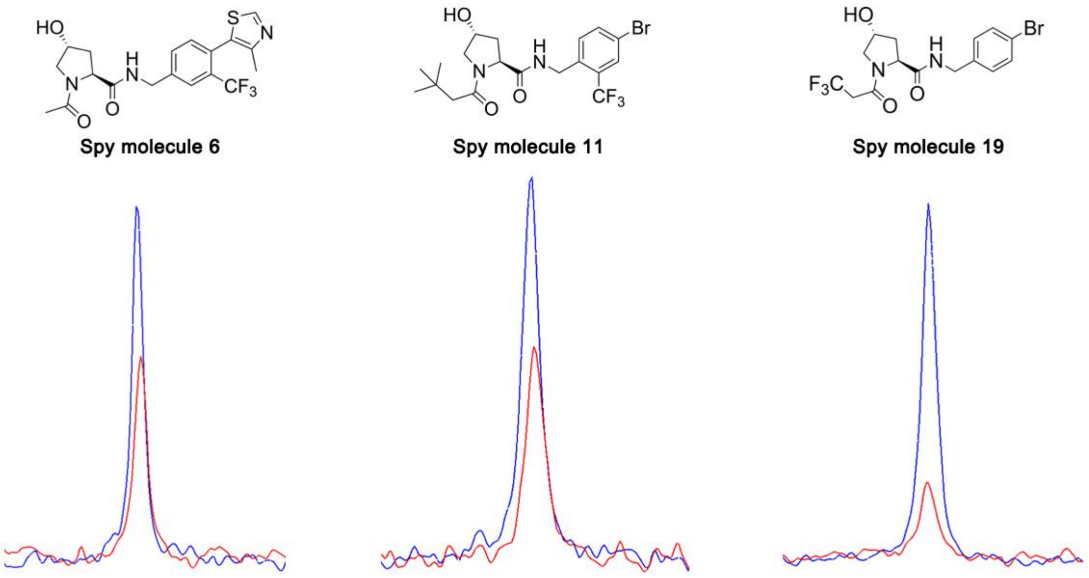

Supplementary Figure 2. Comparison of the assay window of spy molecules 6, 11 and 19 at the same conditions. Overlay of the main ^{19}F CPMG peak (40 scans) of spy molecules **6**, **11** and **19** at 50 µM in absence (blue) or in presence of VBC 1 µM, highlighting the wider assay window of spy molecule **19**. The CPMG delays used for spy molecules **6**, **11** and **19** were, respectively, 634, 447 and 345 ms (see section 6 for details regarding the choice of CPMG delay)

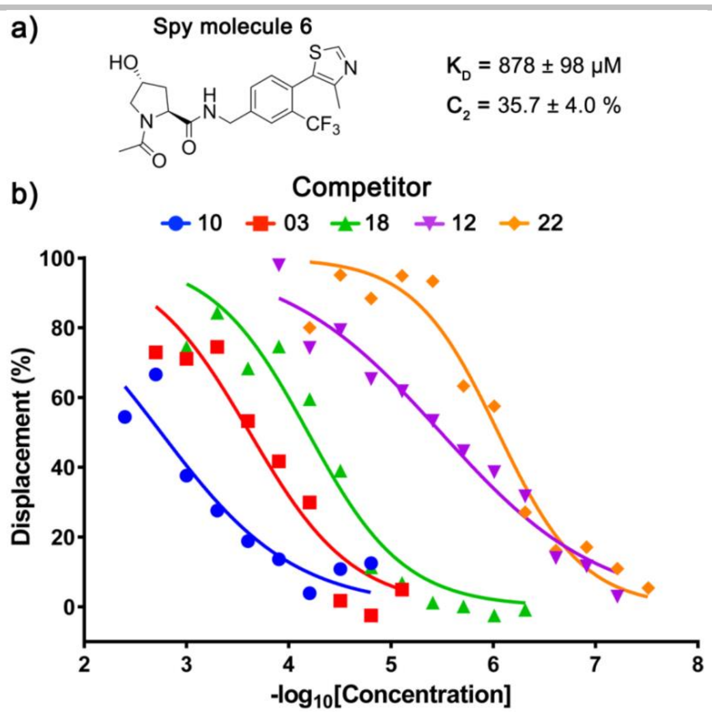

Supplementary Figure 3. Determination of the affinities of VHL binders using spy molecule 6. (a) Structure, dissociation constant and R_2 contrast of spy molecule **6**. The C_2 was obtained with spy molecule at 50 µM and VBC at 1 µM. **(b)** Displacement of spy molecule **6** in presence of different concentrations of five VHL binders (molecules **3**, **10**, **12**, **18** and **22**). Data obtained from ^{19}F CPMG experiments using a CPMG delay of 634 ms and 40 scans.

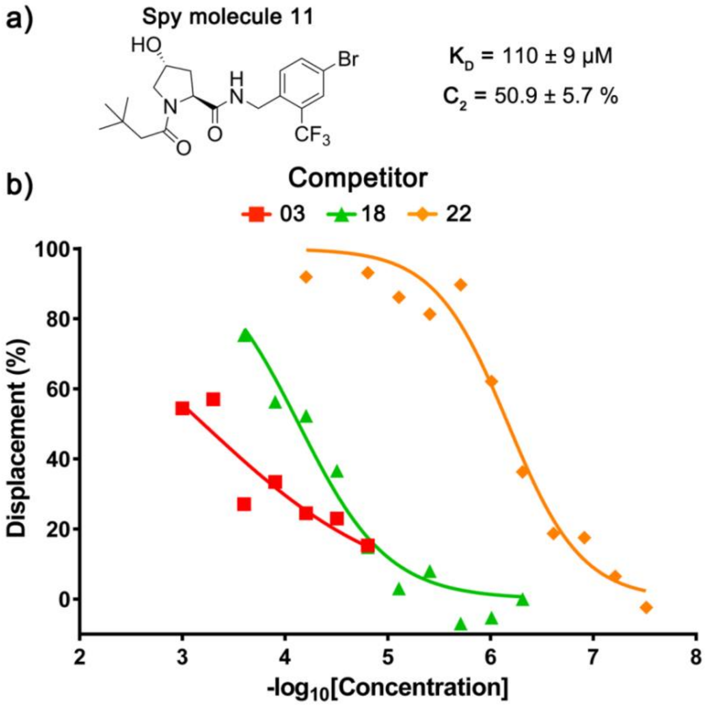

Supplementary Figure 4. Determination of the affinities of VHL binders using spy molecule 11. (a) Structure, dissociation constant and R_2 contrast of spy molecule **11**. The C_2 was obtained with spy molecule at 50 µM and VBC at 1 µM. (b) Displacement of spy molecule **11** in presence of different concentrations of five VHL binders (molecules **3**, **18** and **22**). Competitors **10** and **12** were not used in this case because the chemical shifts of their fluorine peaks overlayed with the spy molecule. Data obtained from ^{19}F CPMG experiments using a CPMG delay of 447 ms and 40 scans.

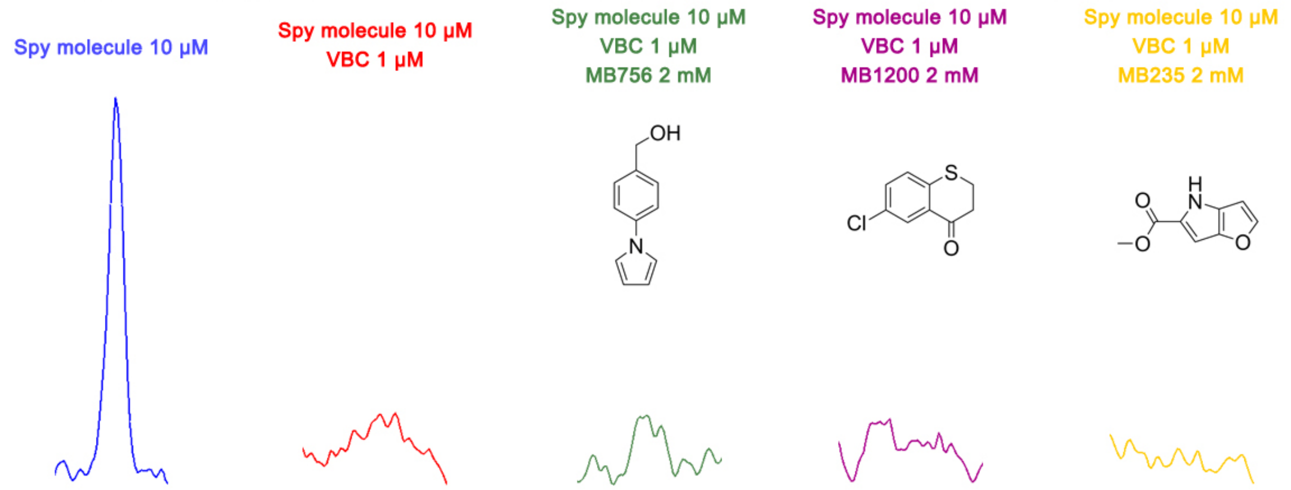

Supplementary Figure 5. Binders of other sites present in the VBC complex do not displace spy molecule 19. ^{19}F CPMG NMR peak of spy molecule **19** at different conditions: free in solution (blue), in presence of VBC (red) and in presence of protein and molecules MB756 (green), MB1200 (violet) and MB235 (yellow), binders of other sites present in VBC previously reported.[1] As these compounds bind to sites not targeted by the spy molecule, no displacement was observed, showing that the competition experiment is site specific. Experiments were performed with a CPMG delay of 258 ms and 120 scans.

2. Compound synthesis and characterization

All the compounds, reagents and solvents used, aside from the compounds specifically prepared for this work, were obtained from commercial sources and used without further purification. For compound purification of intermediates, flash column chromatography was performed using a Teledyne Isco Combiflash Rf or Rf200i, with RediSep Rf Disposable Columns (Normal phase). Where specified, compounds were purified using a Gilson Preparative HPLC System equipped with a Waters X-Bridge C18 column (100 mm x 19 mm; 5 µm particle size) using an eleven minutes gradient (25 mL/min) of: 1) 5% to 95% of acetonitrile : 0.1% formic acid, or 2) 5% to 95% of acetonitrile : 0.1% ammonia.

The NMR characterization was performed either on a Bruker 500 Ultrashield or Bruker Ascend 400 spectrometers. The splitting of the NMR signals are described as follows: singlet (s), doublet (d), triplet (t), quartet (q), multiplet (m) and combinations in case of multiple signal splitting. Chemical shifts are described as parts per million (ppm) and coupling constants (*J*) were calculated in hertz (Hz). The proton (^{1}H) and carbon (^{13}C) spectra were referenced as follows: d6-Chloroform – $CDCl_3$ (δ_H = 7.26 ppm / δ_C = 77.1 ppm) and d5-Methanol – CD_3OD (δ_H = 3.34 ppm / δ_C = 49.1 ppm). For compounds where amide rotamers could be observed, just the signal of the major rotamer was listed.

Reactions were monitored using an Agilent Technologies 1200 series analytical HPLC connected to an Agilent Technologies 6130 quadrupole LC/MS containing an Agilent diode array detector and a Waters XBridge column (50 mm × 2.1 mm, 3.5 µm particle size) for separation of the compounds. Samples were eluted with a 3 minutes gradient of 5% to 95% acetonitrile : 0.1% formic acid.

Abbreviations: ACN (acetonitrile), DCM (dichloromethane), DIPEA (N,N-diisopropylethylamine), DMA (dimethylacetamide), DMF (N,N-dimethylformamide), EtOAc (ethyl acetate), Et_2O (diethyl ether), HATU (1-[bis(dimethylamino)methylene]-1H-1,2,3-triazolo[4,5-b]pyridinium 3-oxide hexafluorophosphate), $MgSO_4$ (Magnesium sulphate), MeOH (methanol), THF (Tetrahydrofuran) and TEA (triethylamine).

For describing the synthesis and characterisation of spy molecules and intermediates, compounds were numbered as shown in the two schemes below. The respective compound numbering in the main text is indicated where applicable (*e.g.* compound **S3a** corresponds to **spy molecule 1** in the main text, and so on).

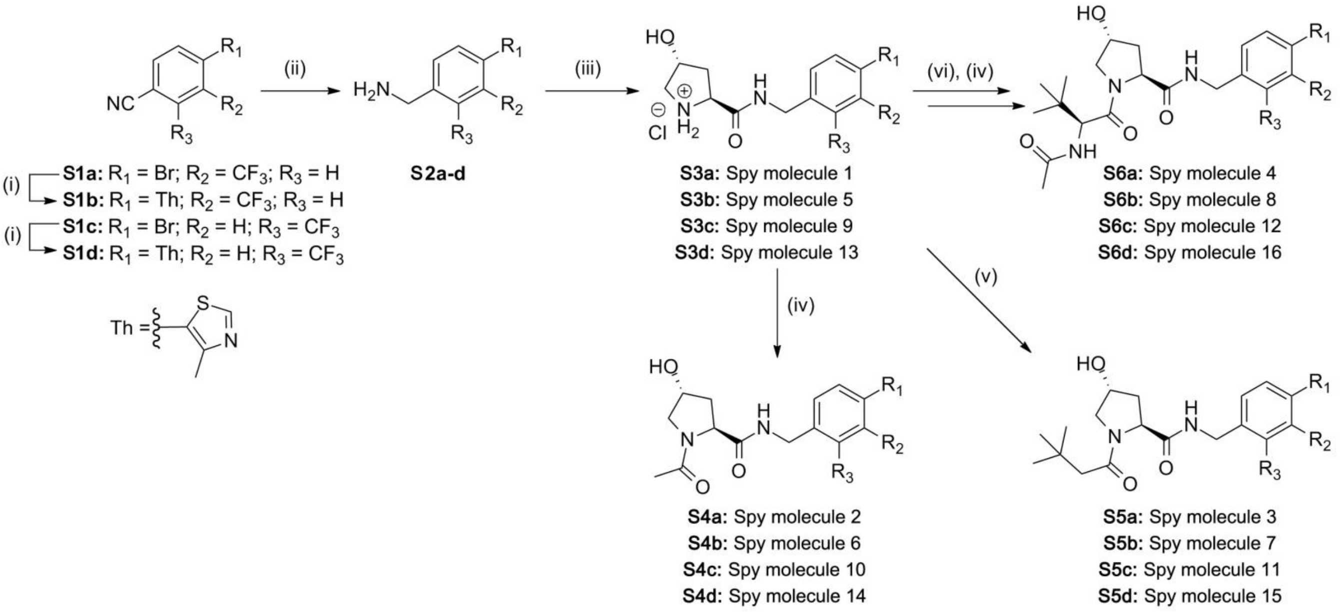

Scheme 2.1 Synthesis of spy molecules with a trifluoromethyl modification on an aromatic position.

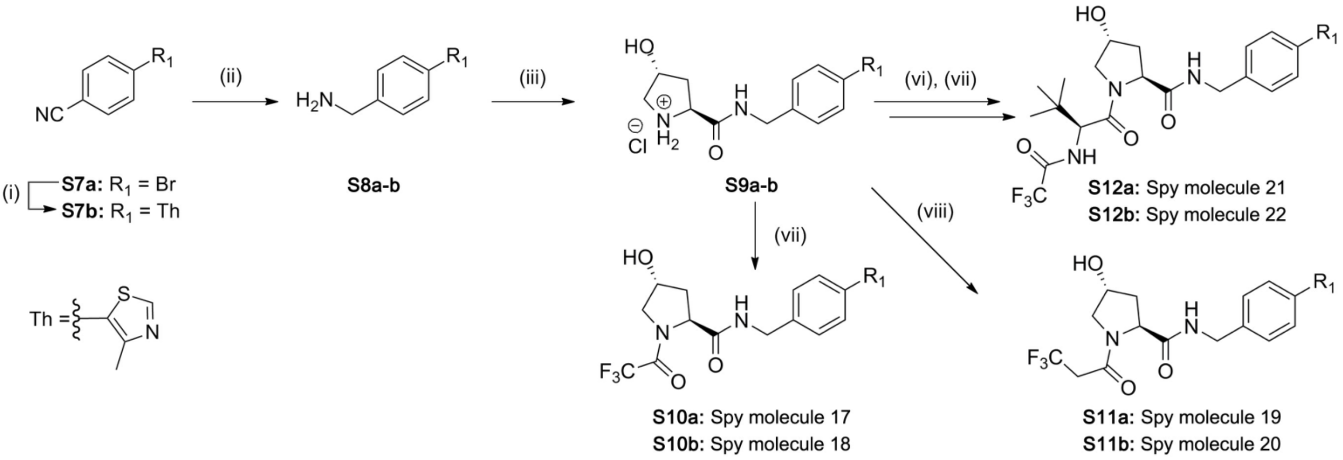

Scheme 2.2 Synthesis of spy molecules with a trifluoromethyl modification on an aliphatic position.

Compounds **S1a**, **S1c** and **S8a** were purchased from commercial sources and used without further purification. The spectroscopic characterization and yields for intermediates **S7b**, **S8b** and **S9b** can be found elsewhere, as these were previously prepared by our group,[2] while all the remaining compounds were synthesized and characterized as described below. For the NMR characterization of compounds **S3a-d** and **S9a-b**, either a chloride or a formate salt was obtained depending on the purification strategy used.

General procedure i. Coupling of aryl bromides with 4-methylthiazole – Synthesis of S1b, S1d and S7b

The aryl bromide (1 equiv.) was dissolved in dimethylacetamide (3 mL per mmol of bromide), followed by the sequential addition of 4-methythiazole (2 equiv.), potassium acetate (2 equiv.) and palladium (II) acetate (0.02 equiv.). The reaction was stirred for approximately two hours at 150 °C under nitrogen atmosphere. The mixture was extracted with brine and dichloromethane. Combined organic phases were concentrated and DMA was removed in the vacuum pump. The desired product was then purified by flash column chromatography with an increasing elution of ethyl acetate (0-100%) in heptane.

4-(4-methylthiazol-5-yl)-3-(trifluoromethyl)benzonitrile (S1b):

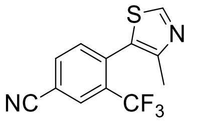

Prepared from 0.3163 g (1.3 mmol) of the respective bromide (**S1a**), resulting in 0.221 g (0.8 mmol, 65%) of the desired product as a white solid. ^{1}H NMR ($CDCl_3$, 400 MHz) δ: 8.84 (s, 1H); 8.08 (d, $J_{H\text{-}H}$ = 1.4 Hz, 1H); 7.89 (dd, $J_{H\text{-}H}$ = 7.9, 1.4 Hz, 1H); 7.54 (d, $J_{H\text{-}H}$ = 7.9 Hz, 1H); 2.25 (s, 3H); ^{13}C NMR ($CDCl_3$, 100 MHz) δ: 152.75, 152.15, 136.04 (q, $^{3}J_{C\text{-}F}$ = 1.7 Hz), 135.06, 134.93, 132.37 (q, $^{2}J_{C\text{-}F}$ = 31.3 Hz), 130.36 (q, $^{3}J_{C\text{-}F}$ = 5.4 Hz), 125.06, 122.56 (q, $^{1}J_{C\text{-}F}$ = 274.6 Hz), 117.16, 113.76, 15.72; ^{19}F NMR ($CDCl_3$, 470 MHz) δ: -60.10.

4-(4-methylthiazol-5-yl)-2-(trifluoromethyl)benzonitrile (S1d):

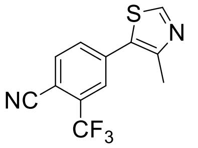

Prepared from 0.8932 g (3.6 mmol) of the respective bromide (**S1c**), resulting in 0.555 g (2.2 mmol, 61%) of the desired product as a bright yellow solid. ^{1}H NMR ($CDCl_3$, 400 MHz): 8.81 (s, 1H); 7.91 (d, $J_{H\text{-}H}$ = 8.08 Hz, 1H); 7.86 (d, $J_{H\text{-}H}$ = 1.5 Hz, 1H); 7.76 (dd, $J_{H\text{-}H}$ = 1.5, 8.08 Hz, 1H); 2.59 (s, 3H); ^{13}C NMR ($CDCl_3$, 125 MHz): 152.49, 151.25, 137.69, 135.29, 133.61 (q, $^{2}J_{C\text{-}F}$ = 32.8 Hz), 132.50, 128.96, 127.27 (q, $^{3}J_{C\text{-}F}$ = 4.5 Hz), 122.28 (q, $^{1}J_{C\text{-}F}$ = 273.8 Hz), 115.32, 109.14, 16.58; ^{19}F NMR ($CDCl_3$, 470 MHz) δ: -62.07.

4-(4-methylthiazol-5-yl)benzonitrile (S7b):

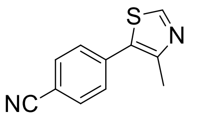

Previously prepared and characterized.[2]

General procedure ii. Reduction of nitriles to amines – Synthesis of S2a-d and S8b

To a stirring solution of the nitrile (1 equiv.) in THF under nitrogen atmosphere, was added a solution of $LiAlH_4$ (1 equiv. from a 1M solution in THF). After an overnight period, the mixture was cooled down in an ice bath and diluted with diethyl ether. Water was then slowly added (1 µL per mg of $LiAlH_4$ added), followed by the addition of 15% NaOH (1 µL per mg of $LiAlH_4$ added) and once more water (3 µL per mg of $LiAlH_4$ added). The ice bath was removed and the mixture. $MgSO_4$ was added and after 15 additional minutes stirring the mixture was filtered, then extracted with HCl solution (pH ≈ 2). The organic phase was discarded and the pH of the aqueous phase was raised to 12 by adding NaOH 1 M. This solution was extracted three times with DCM and the organic phase was concentrated and purified (purification strategy specified for each compound).

(4-bromo-3-(trifluoromethyl)phenyl)methanaminium formate (S2a):

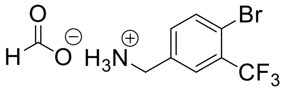

Prepared from 0.264 g (1.06 mmol) of the respective nitrile (**S1a**), resulting in 0.110 g (0.35 mmol, 35%) of the desired product as a white solid. Compound obtained as a formate salt after purification in reverse phase HPLC using a 5 to 95% gradient of formic acid 0.1% and acetonitrile. ^{1}H NMR (CD_3OD, 400 MHz) δ: 8.55 (Formic acid), 7.95-7.89 (m, 2H), 7.64-7.60 (m, 1H), 4.19-4.16 (s, 2H); ^{13}C NMR (CD_3OD, 100 MHz) δ: 170.08 (Formic acid), 137.05, 136.10, 135.20, 131.73 (q, $^{2}J_{C\text{-}F}$ = 31.2 Hz), 129.66 (q, $^{3}J_{C\text{-}F}$ = 5.2 Hz), 124.34 (q, $^{1}J_{C\text{-}F}$ = 273.3 Hz), 121.31, 43.57; ^{19}F NMR (CD_3OD, 470 MHz) δ: -64.05 (CF_3).

(4-(4-methylthiazol-5-yl)-3-(trifluoromethyl)phenyl)methanaminium formate (S2b):

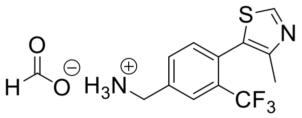

Prepared from 0.221 g (0.82 mmol) of the respective nitrile (**S1b**), resulting in 0.092 g (0.29 mmol, 36%) of the desired product as a yellow solid. Compound obtained as a formate salt after purification in reverse phase HPLC using a 5 to 95% gradient of formic acid 0.1% and acetonitrile. ^{1}H NMR (CD_3OD, 500 MHz) δ: 9.04 (s, 1H), 8.55 (Formic acid), 8.02-7.99 (s, 1H), 7.82-7.79 (d, $J_{H\text{-}H}$ = 7.9 Hz, 1H), 7.58-7.55 (d, $J_{H\text{-}H}$ = 7.9 Hz, 1H), 4.26 (s, 2H), 2.22 (s, 3H); ^{19}F NMR (CD_3OD, 470 MHz) δ: -62.36 (CF_3).

(4-bromo-2-(trifluoromethyl)phenyl)methanaminium formate (S2c):

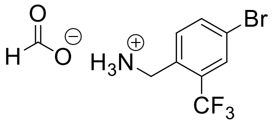

Prepared from 0.523 g (2.09 mmol) of the respective nitrile (**S1c**), resulting in 0.195 g (0.65 mmol, 31%) of the desired product as a white solid. Compound obtained as a formate salt after purification in reverse phase HPLC

using a 5 to 95% gradient of formic acid 0.1% and acetonitrile. ^{1}H NMR (CD_3OD, 500 MHz) δ: 8.52 (Formic acid), 7.98-7.97 (d, J $_{H-H}$ = 1.6 Hz, 1H), 7.95-7.92 (dd, J $_{H-H}$ = 1.6, 8.3 Hz, 1H), 7.65-7.62 (d, J $_{H-H}$ = 8.3 Hz, 1H), 4.24 (s, 2H); ^{13}C NMR (CD_3OD, 125 MHz) δ: 169.65 (Formic acid), 137.32, 133.94, 133.75, 131.55 (q, ^{2}J $_{C-F}$ = 31.2), 130.71 (q, ^{3}J $_{C-F}$ = 5.8), 124.76 (q, ^{1}J $_{C-F}$ = 273.62), 124.04, 40.71 (q, ^{4}J $_{C-F}$ = 2.68); ^{19}F NMR (CD_3OD, 470 MHz) δ: -61.08 (CF_3).

(4-(4-methylthiazol-5-yl)-2-(trifluoromethyl)phenyl)methanamine (S2d):

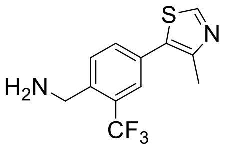

Prepared from 0.398 g (1.49 mmol) of the respective nitrile (**S1d**), resulting in 0.157 g (0.49 mmol, 33%) of the desired product as a yellow solid. Compound obtained as a free amine after purification in reverse phase HPLC using a 5 to 95% gradient of ammonia 0.1% and acetonitrile. ^{1}H NMR (CD_3OD, 500 MHz) δ: 8.96 (s, 1H), 7.84-7.76 (m, 3H), 4.05 (s, 2H), 2.53 (s, 3H); ^{13}C NMR (CD_3OD, 100 MHz) δ: 153.72, 150.26, 142.43, 134.38, 132.24, 131.86, 131.77, 129.35 (q, ^{2}J $_{C-F}$ = 30.2 Hz), 127.50 (q, ^{3}J $_{C-F}$ = 5.8 Hz), 125.81 (q, ^{1}J $_{C-F}$ = 273.7 Hz), 42.83, 15.87; ^{19}F NMR (CD_3OD, 470 MHz) δ: -59.67 (CF_3).

(4-(4-methylthiazol-5-yl)phenyl)methanamine (S8b):

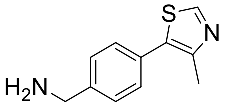

Previously prepared and characterized.[2]

General procedure iii. Amide coupling with Boc-L-hydroxyproline and deprotection – Synthesis of S3a-d and S9a-b

To a solution of amine (1 equiv.) in DMF, Boc-L-hydroxyproline (1 equiv.) was added and the mixture was stirred at room temperature. DIPEA (2 equiv.) was added dropwise and the mixture was stirred for 5 minutes at room temperature. HATU (1.1 equiv.) was added and the mixture was stirred at room temperature for 30-90 minutes (LCMS monitoring). Water was added and the mixture was extracted with ethyl acetate. The combined organic phases were washed with brine, dried over $MgSO_4$ and evaporated under reduced pressure to give the corresponding crude, which was purified by flash column chromatography with an increasing gradient of DCM and 20% MeOH in DCM to yield the desired product. The Boc protected compound was dissolved in DCM, followed by the dropwise addition of a 4M HCl solution in dioxane (at least 3 equiv.). Often an insoluble precipitate starts to be formed. A few drops of MeOH were added to make the solution homogeneous, being kept stirring for approximately one hour. The DCM and the HCl were removed by flushing nitrogen into the solution and residual solvents were evaporated under reduced pressure. To remove traces of impurities, compounds S3a, S3b, S3d and S9a were furtherly purified by preparative HPLC, being obtained as a formate salt, while the remaining compounds were obtained as a chloride salt.

(2*S*,4*R*)-2-((4-bromo-3-(trifluoromethyl)benzyl)carbamoyl)-4-hydroxypyrrolidin-1-ium formate (S3a - spy molecule 1):

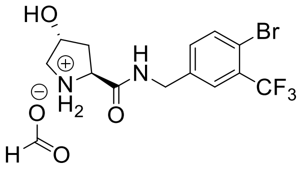

Prepared from 198 mg (0.78 mmol) of amine **S2a**, resulting in 248 mg (0.60 mmol, 78%) of the desired product as a white solid. Compound obtained as a formate salt after purification in reverse phase HPLC using a 5 to 95% gradient of formic acid 0.1% and acetonitrile. HRMS (ESI) $[M+H]^+$ (*m/z*): Calculated for $C_{13}H_{14}BrF_3N_2O_2$: 367.0264; Observed: 367.0299; ^{1}H NMR (CD_3OD, 400 MHz) δ: 8.50 (Formic acid), 7.80 (d, $J_{H\text{-}H}$ = 8.2 Hz, 1H), 7.74 (d, $J_{H\text{-}H}$ = 1.7 Hz, 1H), 7.49 (dd, $J_{H\text{-}H}$ = 1.7, 8.2 Hz, 1H), 4.58 (m, 1H), 4.49 (s, 2H), 4.42 (dd, $J_{H\text{-}H}$ = 7.6, 10.3 Hz, 1H), 3.38 (dd, $J_{H\text{-}H}$ = 3.7, 12.1 Hz, 1H), 3.26 (d, $J_{H\text{-}H}$ = 12.1 Hz, 1H), 2.44 (ddd, $J_{H\text{-}H}$ = 1.5, 7.6, 13.4 Hz, 1H), 2.04 (ddd, $J_{H\text{-}H}$ = 4.2, 10.3, 13.4 Hz, 1H); ^{13}C NMR (CD_3OD, 100 MHz) δ: 171.98, 170.29 (Formic acid), 140.99, 137.34, 134.74, 131.96 (q, $^2J_{C\text{-}F}$ = 31.3 Hz), 129.06 (q, $^3J_{C\text{-}F}$ = 5.6 Hz), 125.25 (q, $^1J_{C\text{-}F}$ = 273.3 Hz), 120.22, 72.47, 60.96, 56.09, 44.19, 40.96; ^{19}F NMR (CD_3OD, 470 MHz) δ: -64.01.

(2*S*,4*R*)-2-((4-(4-methylthiazol-5-yl)-3-(trifluoromethyl)benzyl)carbamoyl)-4-hydroxypyrrolidin-1-ium formate (S3b - spy molecule 5):

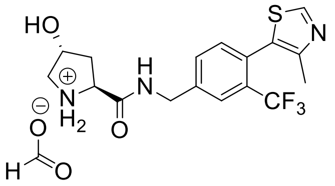

Prepared from 179 mg (0.56 mmol) of amine **S2b**, resulting in 203 mg (0.47 mmol, 84%) of the desired product as a white solid. Compound obtained as a formate salt after purification in reverse phase HPLC using a 5 to 95% gradient of formic acid 0.1% and acetonitrile. MS $[M+H]^+$ (*m/z*): 386.1; ^{1}H NMR (CD_3OD, 400 MHz) δ: 9.02 (s, 1H), 8.51 (Formic acid), 7.81 (s, 1H), 7.66 (d, $J_{H\text{-}H}$ = 7.9 Hz, 1H), 7.46 (d, $J_{H\text{-}H}$ = 7.9 Hz, 1H), 4.58 (s, 2H), 4.55 (m, 1H), 4.35 (dd, $J_{H\text{-}H}$ = 7.7, 9.9 Hz, 1H), 3.30 (m, 1H), 3.20 (dt, $J_{H\text{-}H}$ = 1.4, 12.0 Hz, 1H), 2.41 (ddt, $J_{H\text{-}H}$ = 1.5, 7.6, 13.3 Hz, 1H), 2.20 (s, 3H), 2.03 (ddd, $J_{H\text{-}H}$ = 4.3, 9.9, 13.4 Hz, 1H); ^{13}C NMR (CD_3OD, 100 MHz) δ: 172.51, 169.65 (Formic acid), 154.51, 152.18, 142.12, 135.37, 132.41, 131.77 (q, $^2J_{C\text{-}F}$ = 30.1 Hz), 130.17, 128.59, 126.72 (q, $^3J_{C\text{-}F}$ = 5.6 Hz), 125.19 (q, $^1J_{C\text{-}F}$ = 273.7 Hz), 72.11, 60.40, 55.55, 43.62, 40.41, 15.33; ^{19}F NMR (CD_3OD, 470 MHz) δ: -59.37.

(2*S*,4*R*)-2-((4-bromo-2-(trifluoromethyl)benzyl)carbamoyl)-4-hydroxypyrrolidin-1-ium chloride (S3c - spy molecule 9):

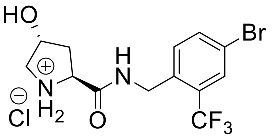

Prepared from 11 mg (0.043 mmol) of amine **S2c**, resulting in 14 mg (0.035 mmol, 80%) of the desired product as a white solid. HRMS (ESI) $[M+H]^+$ (*m/z*): Calculated for $C_{13}H_{14}BrF_3N_2O_2$: 367.0264; Observed: 367.0280; ^{1}H NMR (CD_3OD, 500 MHz) δ: 7.88-7.85 (d, $J_{\text{H-H}}$ = 1.8 Hz, 1H), 7.82-7.79 (dd, $J_{\text{H-H}}$ = 1.8, 8.4 Hz, 1H), 7.47-7.44(d, $J_{\text{H-H}}$ = 8.4 Hz, 1H), 4.61-4.53 (m, 2H), 4.41-4.38 (m, 1H), 4.00-3.76 (t, $J_{\text{H-H}}$ = 8.3 Hz, 1H), 3.06-3.01 (dd, $J_{\text{H-H}}$ = 4.0, 12.0 Hz, 1H), 2.96-2.91 (dt, $J_{\text{H-H}}$ = 1.8, 12.0 Hz, 1H), 2.23-2.17 (ddt, $J_{\text{H-H}}$ = 1.8, 8.0, 13.4 Hz, 1H), 1.91-1.85 (ddd, $J_{\text{H-H}}$ = 5.0, 8.7, 13.4 Hz, 1H); ^{13}C NMR (CD_3OD, 125 MHz) δ: 177.58, 137.72, 136.72, 132.33, 130.63 (q, $^2J_{\text{C-F}}$ = 31.5 Hz), 130.09 (q, $^3J_{\text{C-F}}$ = 5.8 Hz) , 125.02 (q, $^1J_{\text{C-F}}$ = 273.6 Hz), 122.07, 73.67, 60.82, 56.15, 40.99, 40.19 (q, $^4J_{\text{C-F}}$ = 2.9 Hz) ; ^{19}F NMR (CD_3OD, 470 MHz) δ: -61.86 (CF_3).

(2*S*,4*R*)-2-((4-(4-methylthiazol-5-yl)-2-(trifluoromethyl)benzyl)carbamoyl)-4-hydroxypyrrolidin-1-ium chloride (S3d - spy molecule 13):

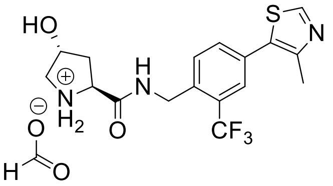

Prepared from 164 mg (0.60 mmol) of amine **S2d**, resulting in 239 mg (0.55 mmol, 92%) of the desired product as a white solid. Compound obtained as a formate salt after purification in reverse phase HPLC using a 5 to 95% gradient of formic acid 0.1% and acetonitrile. HRMS (ESI) $[M+H]^+$ (*m/z*): Calculated for $C_{17}H_{18}F_3N_3O_2S$: 386.1145; Observed: 386.1168; ^{1}H NMR (CD_3OD, 500 MHz) δ: 8.98 (s, 1H), 8.55 (Formic acid), 7.80 (s, 1H), 7.77 (dd, $J_{\text{H-H}}$ = 1.4, 8.1 Hz, 1H), 7.66 (d, $J_{\text{H-H}}$ = 8.1 Hz, 1H), 4.69 (m, 2H), 4.48 (m, 1H), 4.18 (t, $J_{\text{H-H}}$ = 8.7 Hz, 1H), 3.18 (dd, $J_{\text{H-H}}$ = 3.5, 11.7 Hz, 1H), 3.07 (d, $J_{\text{H-H}}$ = 12.1 Hz, 1H), 2.53 (s, 3H), 2.32 (ddt, $J_{\text{H-H}}$ = 1.6, 7.8, 13.5 Hz, 1H), 1.98 (ddd, $J_{\text{H-H}}$ = 4.7, 9.3, 13.5 Hz, 1H); ^{13}C NMR (CD_3OD, 125 MHz) δ: 174.57, 170.18 (Formic acid), 153.88, 150.39, 138.01, 134.30, 132.82, 131.65, 131.37, 129.60 (q, $^2J_{\text{C-F}}$ = 30.9 Hz), 127.78 (q, $^3J_{\text{C-F}}$ = 5.9 Hz), 125.64 (q, $^1J_{\text{C-F}}$ = 273.4 Hz), 72.77, 60.56, 55.82, 40.64, 15.87; ^{19}F NMR (CD_3OD, 470 MHz) δ: -60.11.

(2*S*,4*R*)-2-((4-bromobenzyl)carbamoyl)-4-hydroxypyrrolidin-1-ium chloride (S9a):

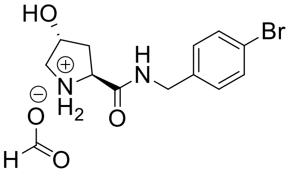

Prepared from 182 mg (1.00 mmol) of amine **S8a**, resulting in 294 mg (0.47 mmol, 84%) of the desired product as a white solid. Compound obtained as a formate salt after purification in reverse phase HPLC using a 5 to 95% gradient of formic acid 0.1% and acetonitrile. ^{1}H NMR (CD_3OD, 500 MHz) δ: 7.51-7.47 (m, 2H), 7.26-7.23 (m, 2H), 4.52-4.49 (m, 1H), 4.41-4.38 (s, 2H), 4.27-4.23 (dd, $J_{\text{H-H}}$ = 7.9, 9.4 Hz, 1H), 3.27-3.23 (dd, $J_{\text{H-H}}$ = 3.8, 12.1

Hz, 1H), 3.16-3.13 (m, 1H), 2.37-2.32 (m, 1H), 2.01-1.95 (ddd, $J_{\text{H-H}}$ = 4.5, 9.6, 13.5 Hz, 1H); ^{13}C NMR (CD_3OD, 100 MHz) δ: 173.09, 170.38 (Formic acid), 139.11, 132.76, 130.63, 122.13, 72.35, 60.39, 55.57, 43.57, 40.50.

(2*S*,4*R*)-4-hydroxy-2-((4-(4-methylthiazol-5-yl)benzyl)carbamoyl)pyrrolidin-1-ium chloride (S9b):

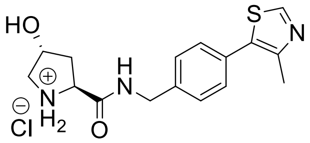

Previously prepared and characterized.[2]

General procedure iv. Amine acetylation – Synthesis of S4a-d and S6a-d

The amine (1 equiv.) was dissolved in DMF, followed by the addition of triethylamine (2.0 equiv.) and 1-acetylimidazole (1.0 equiv.). The mixture was kept stirring overnight, followed by extraction with ethyl acetate and brine. Combined organic phases were dried with $MgSO_4$, concentrated and purified in the acidic Gilson HPLC system, yielding the desired product.

(2*S*,4*R*)-1-acetyl-N-(4-bromo-3-(trifluoromethyl)benzyl)-4-hydroxypyrrolidine-2-carboxamide (S4a - spy molecule 2):

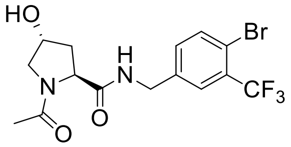

Prepared from 50.3 mg (0.125 mmol) of hydrochloride salt of amine **S3a**, resulting in 47.7 mg (0.117 mmol, 93%) of the desired product as a white solid. HRMS (ESI) $[M+H]^+$ (*m/z*): Calculated for $C_{15}H_{17}BrF_3N_2O_3$: 409.0369; Observed: 409.0387; ^{1}H NMR (CD_3OD, 400 MHz) δ: 7.79 (d, $J_{\text{H-H}}$ = 8.3 Hz, 1H), 7.73 (d, $J_{\text{H-H}}$ = 1.6 Hz, 1H), 7.49 (dd, $J_{\text{H-H}}$ = 1.6, 8.3 Hz, 1H), 4.48 (m, 4H), 3.80 (dd, $J_{\text{H-H}}$ = 4.2, 11.0 Hz, 1H), 3.58 (dd, $J_{\text{H-H}}$ = 1.8, 11.0 Hz, 1H), 2.27 (dddd, $J_{\text{H-H}}$ = 1.6, 3.0, 8.0, 13.1 Hz, 1H), 2.12 (s, 3H), 2.07 (ddd, $J_{\text{H-H}}$ = 4.6, 8.3, 13.1 Hz, 1H); ^{13}C NMR (CD_3OD, 100 MHz) δ: 175.88, 173.49, 141.40, 137.19, 134.38, 131.77 (q, $^2J_{\text{C-F}}$ = 31.3 Hz), 128.72 (q, $^3J_{\text{C-F}}$ = 5.5 Hz), 125.32 (q, $^1J_{\text{C-F}}$ = 273.1 Hz), 119.77, 71.70, 61.21, 58.21, 43.87, 40.35, 23.18; ^{19}F NMR (CD_3OD, 470 MHz) δ: -63.86 (CF_3).

(2*S*,4*R*)-1-acetyl-4-hydroxy-N-(4-(4-methylthiazol-5-yl)-3-(trifluoromethyl)benzyl)pyrrolidine-2-carboxamide (S4b - spy molecule 6):

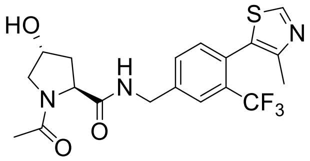

Prepared from 26 mg (0.060 mmol) of formate salt of amine **S3b**, resulting in 20 mg (0.047 mmol, 78%) of the desired product as a white solid. HRMS (ESI) $[M+H]^+$ (*m/z*): Calculated for $C_{19}H_{20}F_3N_3O_3S$: 428.1250;

Observed: 428.1272; ^{1}H NMR (CD_3OD, 500 MHz) δ: 9.04-9.02 (s, 1H), 7.83-7.81 (s, 1H), 7.74-7.66 (d, J_{H-H} = 7.9 Hz, 1H), 7.49-7.44 (d, J_{H-H} = 7.9 Hz, 1H), 4.66-4.44 (m, 4H), 3.83-3.79 (dd, J_{H-H} = 4.3, 11.1 Hz, 1H), 3.61-3.57 (dd, J_{H-H} = 1.7, 11.1 Hz, 1H), 2.48-2.27 (m, 1H), 2.22-2.20 (s, 3H), 2.20-2.07 (m, 1H), 2.14-1.96 (s, 3H); ^{13}C NMR (CD_3OD, 125 MHz) δ: 175.20, 172.75, 154.45, 152.11, 142.44, 135.25, 132.04, 131.57 (q, $^{2}J_{C-F}$ = 29.8 Hz), 129.70 (q, $^{3}J_{C-F}$ = 1.9 Hz), 128.81, 126.42 (q, $^{3}J_{C-F}$ = 5.3 Hz), 125.25 (q, $^{1}J_{C-F}$ = 273.1 Hz), 70.96, 60.48, 57.48, 43.39, 39.63, 22.44, 15.34; ^{19}F NMR (CD_3OD, 470 MHz) δ: -60.75 (CF_3).

(2*S*,4*R*)-1-acetyl-N-(4-bromo-2-(trifluoromethyl)benzyl)-4-hydroxypyrrolidine-2-carboxamide (S4c - spy molecule 10):

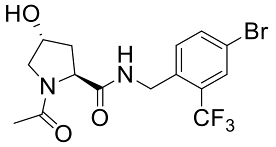

Prepared from 29 mg (0.072 mmol) of amine **S3c**, resulting in 22 mg (0.054 mmol, 75%) of the desired product as a white solid. HRMS (ESI) [M+H]$^{+}$ (*m/z*): Calculated for $C_{15}H_{16}BrF_3N_2O_3$: 409.0369; Observed: 409.0377; ^{1}H NMR (CD_3OD, 500 MHz) δ: 7.89-7.80 (m, 2H), 7.66-7.47 (d, J_{H-H} = 8.4 Hz, 1H), 4.66-4.43 (m, 4H), 3.83-3.79 (dd, J_{H-H} = 4.1, 11.0 Hz, 1H), 3.61-3.57 (dd, J_{H-H} = 1.7, 11.1 Hz, 1H), 2.45-2.26 (dddd, J_{H-H} = 1.7, 2.8, 7.8, 13.2 Hz, 1H), 2.21-2.07 (ddd, J_{H-H} = 4.6, 8.5, 13.2 Hz, 1H), 2.15-1.97 (s, 3H); ^{13}C NMR (CD_3OD, 125 MHz) δ: 175.33, 172.81, 137.72, 136.75, 132.12, 130.26 (q, $^{2}J_{C-F}$ = 31.3 Hz), 129.81 (q, $^{3}J_{C-F}$ = 6.1 Hz), 125.05 (q, $^{1}J_{C-F}$ = 273.5 Hz), 121.81, 70.98, 60.49, 57.52, 40.29 (q, $^{4}J_{C-F}$ = 3.4 Hz), 39.57, 22.44; ^{19}F NMR (CD_3OD, 470 MHz) δ: -62.14 (CF_3).

(2*S*,4*R*)-1-acetyl-4-hydroxy-N-(4-(4-methylthiazol-5-yl)-2-(trifluoromethyl)benzyl)pyrrolidine-2-carboxamide (S4d - spy molecule 14):

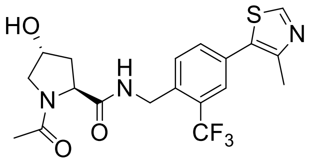

Prepared from 39 mg (0.090 mmol) of amine **S3d**, resulting in 28 mg (0.066 mmol, 72%) of the desired product as a white solid. HRMS (ESI) [M+H]$^{+}$ (*m/z*): Calculated for $C_{19}H_{20}F_3N_3O_3S$: 428.1250; Observed: 428.1265; ^{1}H NMR (CD_3OD, 500 MHz) δ: 8.98 (s, 1H), 7.86-7.76 (m, 3H), 4.75-4.51 (m, 4H), 3.84-3.80 (dd, J_{H-H} = 4.2, 11.1 Hz, 1H), 3.63-3.56 (dt, J_{H-H} = 1.7, 11.1 Hz, 1H), 2.53 (s, 3H), 2.47-2.27 (dddd, J_{H-H} = 1.6, 2.9, 7.9, 13.2 Hz, 1H), 2.24-2.10 (m, 1H), 2.15-2.00 (s, 3H); ^{13}C NMR (CD_3OD, 125 MHz) δ: 175.35, 172.81, 153.75, 150.23, 138.38, 134.32, 132.30, 131.83, 130.80, 129.08 (q, $^{2}J_{C-F}$ = 30.9 Hz), 127.43 (q, $^{3}J_{C-F}$ = 5.9 Hz), 125.70 (q, $^{1}J_{C-F}$ = 273.6 Hz), 70.98, 60.52, 57.53, 40.50 (q, $^{4}J_{C-F}$ = 3.2 Hz), 39.61, 22.47, 15.90; ^{19}F NMR (CD_3OD, 470 MHz) δ: -60.45 (CF_3).

(2*S*,4*R*)-1-((*S*)-2-acetamido-3,3-dimethylbutanoyl)-*N*-(4-bromo-3-(trifluoromethyl)benzyl)-4-hydroxypyrrolidine-2-carboxamide (S6a - spy molecule 4):

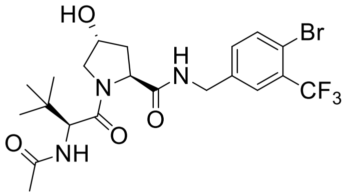

Overall yield of 93% (90 mg, 0.17 mmol), starting from amine **S3a** (77 mg, 0.18 mmol). Yield includes also step (*vi*). HRMS (ESI) $[M+H]^+$ (*m/z*): Calculated for $C_{19}H_{20}F_3N_3O_3S$: 428.1250; Observed: 428.1265; ^1H NMR (CD_3OD, 500 MHz) δ: 7.82-7.58 (m, 3H), 4.63 (s, 1H), 4.60-4.45 (m, 3H), 4.34 (d, J_{H-H} = 15.5 Hz, 1H), 3.92 (d, J_{H-H} = 11.0 Hz, 1H), 3.81 (dd, J_{H-H} = 3.8, 11.0 Hz, 1H), 2.42-2.19 (m, 1H), 2.16-1.99 (m, 4H), 1.04 (s, 9H); ^{13}C NMR (CD_3OD, 125 MHz) δ: 174.75, 173.22, 172.39, 140.68, 136.32, 133.85, 131.02 (q, $^2J_{C-F}$ = 30.9 Hz), 128.15 (q, $^3J_{C-F}$ = 5.4 Hz), 124.56 (q, $^1J_{C-F}$ = 272.5 Hz), 119.03, 71.17, 60.83, 59.25, 58.06, 43.21, 39.00, 36.53, 27.03, 22.39; ^{19}F NMR (CD_3OD, 470 MHz) δ: -62.30 (CF_3).

(2*S*,4*R*)-1-((*S*)-2-acetamido-3,3-dimethylbutanoyl)-4-hydroxy-*N*-(4-(4-methylthiazol-5-yl)-3-(trifluoromethyl)benzyl)pyrrolidine-2-carboxamide (S6b - spy molecule 8):

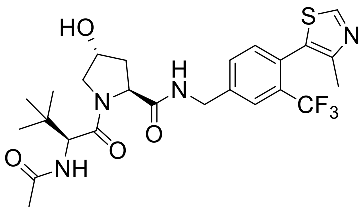

Overall yield of 84% (66 mg, 0.12 mmol), starting from amine **S3b** (63 mg, 0.15 mmol). Yield includes also step (*vi*). HRMS (ESI) $[M+H]^+$ (*m/z*): Calculated for $C_{25}H_{31}F_3N_4O_4S$: 541.2091; Observed: 541.2105; ^1H NMR (CD_3OD, 500 MHz) δ: 9.03 (s, 1H), 7.87-7.84 (s, 1H), 7.78-7.69 (d, J_{H-H} = 7.9 Hz, 1H), 7.48-7.40 (d, J_{H-H} = 7.9 Hz, 1H), 4.82-4.43 (m, 5H), 3.96-3.92 (d, J_{H-H} = 11.0 Hz, 1H), 3.85-3.81 (dd, J_{H-H} = 3.9, 11.0 Hz, 1H), 2.42-2.23 (m, 1H), 2.23-2.19 (s, 3H), 2.18-2.06 (ddd, J_{H-H} = 4.5, 9.2, 13.1 Hz, 1H), 2.05-1.99 (s, 3H), 1.08-1.02 (s, 9H); ^{13}C NMR (CD_3OD, 125 MHz) δ: 174.83, 173.22, 172.39, 154.46, 152.09, 142.49, 135.13, 132.28, 131.57 (q, $^2J_{C-F}$ = 29.7 Hz), 129.69 (q, $^3J_{C-F}$ = 1.9 Hz), 128.81, 126.59 (q, $^3J_{C-F}$ = 5.3 Hz), 125.24 (q, $^1J_{C-F}$ = 273.3 Hz), 71.19, 60.87, 59.26, 58.08, 43.51, 39.05, 36.54, 27.06, 22.39, 15.35; ^{19}F NMR (CD_3OD, 470 MHz) δ: -60.65 (CF_3).

(2*S*,4*R*)-1-((*S*)-2-acetamido-3,3-dimethylbutanoyl)-*N*-(4-bromo-3-(trifluoromethyl)benzyl)-4-hydroxypyrrolidine-2-carboxamide (S6c - spy molecule 12):

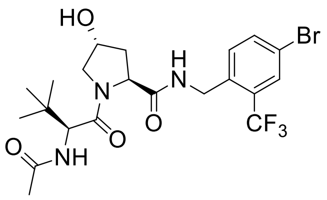

Prepared from 17 mg (0.042 mmol) of amine **S3c**, resulting in 18 mg (0.034 mmol, 82%) of the desired product as a white solid. Yield includes also step (*vi*). HRMS (ESI) $[M+H]^+$ (*m/z*): $C_{21}H_{27}BrF_3N_3O_4$: 522.1210; Observed: 522.1180; ^1H NMR (CD_3OD, 500 MHz) δ: 7.87-7.83 (d, J_{H-H} = 1.1 Hz, 1H), 7.77-7.72 (m, 2H), 4.64 (s, 1H),

4.71-4.41 (m, 4H), 3.96-3.93 (d, $J_{H\text{-}H}$ = 11.1 Hz, 1H), 3.84-3.80 (dd, $J_{H\text{-}H}$ = 3.9, 11.1 Hz, 1H), 2.39-2.22 (m, 1H), 2.14-2.07 (ddd, $J_{H\text{-}H}$ = 4.4, 9.4, 13.1 Hz, 1H), 2.05-2.00 (s, 3H), 1.09-1.02 (s, 9H); ^{13}C NMR (CD_3OD, 125 MHz) δ: 175.02, 173.24, 172.47, 137.74, 136.54, 132.45, 130.32 (q, $^2J_{C\text{-}F}$ = 31.3 Hz), 129.80 (q, $^3J_{C\text{-}F}$ = 6.1 Hz), 125.05 (q, $^1J_{C\text{-}F}$ = 273.7 Hz), 121.79, 71.23, 60.90, 59.30, 58.11, 40.40 (q, $^4J_{C\text{-}F}$ = 3.3 Hz), 38.94, 36.58, 27.04, 22.39; ^{19}F NMR (CD_3OD, 470 MHz) δ: -62.12 (CF_3).

(2*S*,4*R*)-1-((*S*)-2-acetamido-3,3-dimethylbutanoyl)-4-hydroxy-*N*-(4-(4-methylthiazol-5-yl)-3-(trifluoromethyl)benzyl)pyrrolidine-2-carboxamide (S6d - spy molecule 16):

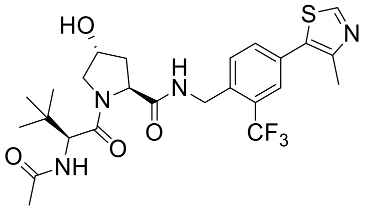

Prepared from 53 mg (0.123 mmol) of amine **S3d**, resulting in 51 mg (0.094 mmol, 77%) of the desired product as a white solid. Yield includes also step (*vi*). HRMS (ESI) $[M+H]^+$ (*m/z*): $C_{25}H_{31}F_3N_4O_4S$: 541.2091; Observed: 541.2091; ^{1}H NMR (CD_3OD, 500 MHz) δ: 8.98 (s, 1H), 7.97-7.93 (d, $J_{H\text{-}H}$ = 8.1 Hz, 1H), 7.80-7.76 (d, $J_{H\text{-}H}$ = 1.3 Hz, 1H), 7.76-7.69 (dd, $J_{H\text{-}H}$ = 1.3, 8.1 Hz, 1H), 4.81-4.75 (d, $J_{H\text{-}H}$ = 16.2 Hz, 1H), 4.67-4.51 (m, 4H), 3.97-3.93 (d, $J_{H\text{-}H}$ = 11.0 Hz, 1H), 3.86-3.81 (dd, $J_{H\text{-}H}$ = 3.9, 11.0 Hz, 1H), 2.54-2.51 (s, 3H), 2.31-2.24 (m, 1H), 2.17-2.10 (ddd, $J_{H\text{-}H}$ = 4.5, 9.2, 13.1 Hz, 1H), 2.06-2.02 (s, 3H), 1.09-1.03 (s, 9H); ^{13}C NMR (CD_3OD, 125 MHz) δ: 175.06, 173.25, 172.49, 153.78, 150.21, 138.42, 134.11, 132.30, 131.83, 131.14, 129.17 (q, $^2J_{C\text{-}F}$ = 30.9 Hz), 127.47 (q, $^3J_{C\text{-}F}$ = 5.9 Hz), 125.70 (q, $^1J_{C\text{-}F}$ = 273.4 Hz), 71.25, 60.93, 59.33, 58.13, 40.64 (q, $^4J_{C\text{-}F}$ = 3.3 Hz), 38.97, 36.61, 27.06, 22.39, 15.89; ^{19}F NMR (CD_3OD, 470 MHz) δ: -61.96.

General procedure v. Amide coupling with 3,3-dimethylbutyric acid – Synthesis of S5a-d

To a solution of amine (1 equiv.) in DMF, 3,3-dimethylbutyric acid (1 equiv.) was added and the mixture was stirred at room temperature. DIPEA (2 equiv.) was added dropwise and the mixture was stirred for 5 minutes at room temperature. HATU (1.1 equiv.) was added and the mixture was stirred at room temperature for 60 minutes (LCMS monitoring). Water was added and the mixture was extracted with ethyl acetate. The combined organic phases were washed with brine, dried over $MgSO_4$ and evaporated under reduced pressure to give the corresponding crude, which was purified in the acidic Gilson preparative HPLC.

(2*S*,4*R*)-*N*-(4-bromo-3-(trifluoromethyl)benzyl)-1-(3,3-dimethylbutanoyl)-4-hydroxypyrrolidine-2-carboxamide (S5a - spy molecule 3):

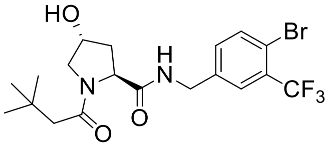

Prepared from 50.6 mg (0.125 mmol) of hydrochloride salt of the amine **S3a** resulting in 49.8 mg (0.107 mmol, 86%) of the desired product as a white solid. HRMS (ESI) $[M+H]^+$ (*m/z*): Calculated for $C_{19}H_{24}BrF_3N_2O_3$:

465.0995; Observed: 465.1013; ^{1}H NMR (CD_3OD, 400 MHz) δ: 7.82-7.75 (d, J_{H-H} = 8.2 Hz, 1H), 7.76-7.73 (m, 1H), 7.56-7.47 (dd, J_{H-H} = 1.6, 8.2 Hz, 1H), 4.58-4.36 (m, 4H), 3.80-3.75 (dd, J_{H-H} = 4.1, 11.0 Hz, 1H), 3.74-3.63 (dt, J_{H-H} = 1.7, 11.0 Hz, 1H), 2.37-1.81 (m, 4H), 1.10-0.97 (s, 9H); ^{13}C NMR (CD_3OD, 100 MHz) δ: 175.29, 173.80, 140.67, 136.36, 133.75, 131.03 (q, $^{2}J_{C-F}$ = 31.8 Hz), 128.08 (q, $^{3}J_{C-F}$ = 5.5 Hz), 124.56 (q, $^{1}J_{C-F}$ = 272.7 Hz), 119.01, 71.10, 60.54, 57.82, 47.76, 43.17, 39.24, 32.74, 30.47; ^{19}F NMR (CD_3OD, 470 MHz) δ: -63.81 (CF_3).

(2*S*,4*R*)-1-(3,3-dimethylbutanoyl)-4-hydroxy-*N*-(4-(4-methylthiazol-5-yl)-3-(trifluoromethyl)benzyl)pyrrolidine-2-carboxamide (S5b - spy molecule 7):

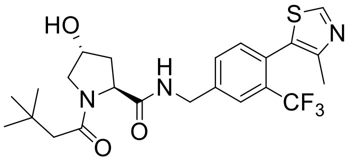

Prepared with 0.023 g (0.053 mmol) of amine **S3b**, resulting in 0.020 mg (0.042 mmol, 80%) of product as a white solid. HRMS (ESI) $[M+H]^+$ (*m/z*): Calculated for $C_{23}H_{28}F_3N_3O_3S$: 484.1876; Observed: 484.1891; ^{1}H NMR (CD_3OD, 500 MHz) δ: 9.04-9.02 (s, 1H), 7.87-7.84 (m, 1H), 7.75-7.68 (m, 1H), 7.48-7.41 (d, J_{H-H} = 7.8 Hz, 1H), 4.65-4.43 (m, 4H), 3.82-3.78 (dd, J_{H-H} = 4.2, 11.1 Hz, 1H), 3.75-3.65 (dt, J_{H-H} = 2.0, 11.1 Hz, 1H), 2.38-2.24 (m, 3H), 2.22-2.20 (s, 3H), 2.19-2.06 (ddd, J_{H-H} = 4.4, 8.4, 13.3 Hz, 1H), 1.10-0.97 (s, 9H); ^{13}C NMR (CD_3OD, 125 MHz) δ: 175.37, 173.82, 154.44, 152.09, 142.49, 135.18, 132.21, 131.59 (q, $^{2}J_{C-F}$ = 30.5 Hz), 129.69 (q, $^{3}J_{C-F}$ = 1.9 Hz), 128.83, 126.55 (q, $^{3}J_{C-F}$ = 5.5 Hz), 125.25 (q, $^{1}J_{C-F}$ = 273.1 Hz), 71.13, 60.58, 57.85, 47.77, 43.65, 39.28, 32.77, 30.48, 15.89; ^{19}F NMR (CD_3OD, 470 MHz) δ: -60.68 (CF_3).

(2*S*,4*R*)-*N*-(4-bromo-2-(trifluoromethyl)benzyl)-1-(3,3-dimethylbutanoyl)-4-hydroxypyrrolidine-2-carboxamide (S5c - spy molecule 11):

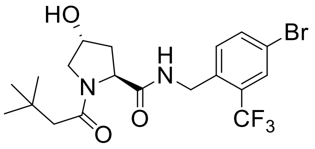

Prepared from 17 mg (0.042 mmol) of amine **S3c**, resulting in 15 mg (0.032 mmol, 76%) of the desired product as a white solid. HRMS (ESI) $[M+H]^+$ (*m/z*): Calculated for $C_{19}H_{24}BrF_3N_2O_3$: 465.0995; Observed: 465.0997; ^{1}H NMR (CD_3OD, 500 MHz) δ: 7.90-7.83 (d, J_{H-H} = 1.8 Hz, 1H), 7.83-7.60 (dd, J_{H-H} = 1.8, 8.3 Hz, 1H), 7.72-7.50 (d, J_{H-H} = 8.3 Hz, 1H), 4.66-4.42 (m, 4H), 3.81-3.77 (dd, J_{H-H} = 4.1, 11.1 Hz, 1H), 3.74-3.66 (dd, J_{H-H} = 1.9, 11.1 Hz, 1H), 2.40-2.16 (m, 3H), 2.14-2.07 (ddd, J_{H-H} = 4.6, 8.5, 13.2 Hz, 1H), 1.12-1.02 (s, 9H); ^{13}C NMR (CD_3OD, 125 MHz) δ: 175.51, 173.92, 137.74 (q, $^{3}J_{C-F}$ = 1.2 Hz), 136.65, 132.32, 130.32 (q, $^{2}J_{C-F}$ = 31.3 Hz), 129.80 (q, $^{3}J_{C-F}$ = 6.1 Hz), 125.05 (q, $^{1}J_{C-F}$ = 273.8 Hz), 121.79, 71.14, 60.58, 57.91, 47.82, 40.36 (q, $^{4}J_{C-F}$ = 3.3 Hz), 39.21, 32.87, 30.50; ^{19}F NMR (CD_3OD, 470 MHz) δ: -62.11 (CF_3).

(2*S*,4*R*)-1-(3,3-dimethylbutanoyl)-4-hydroxy-*N*-(4-(4-methylthiazol-5-yl)-2-(trifluoromethyl)benzyl)pyrrolidine-2-carboxamide (S5d - spy molecule 15):

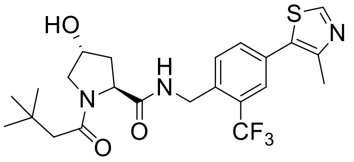

Prepared from 24 mg (0.056 mmol) of amine **S3d**, resulting in 22 mg (0.045 mmol, 82%) of the desired product as a white solid. HRMS (ESI) $[M+H]^+$ (*m/z*): Calculated for $C_{23}H_{28}F_3N_3O_3S$: 484.1876; Observed: 484.1894; ^1H NMR (CD_3OD, 500 MHz) δ: 9.00-8.98 (s, 1H), 7.91-7.87 (d, $J_{H\text{-}H}$ = 7.9 Hz, 1H), 4.76-4.51 (m, 4H), 3.83-3.78 (dd, $J_{H\text{-}H}$ = 4.1, 11.1 Hz, 1H), 3.74-3.67 (dt, $J_{H\text{-}H}$ = 1.5, 11.1 Hz, 1H), 2.53 (s, 3H), 2.41-2.11 (m, 4H), 1.12-1.02 (s, 9H); ^{13}C NMR (CD_3OD, 125 MHz) δ: 175.55, 173.95, 154.03, 149.74, 138.59, 134.26, 132.13, 132.03, 131.06, 129.16 (q, $^2J_{C\text{-}F}$ = 30.9 Hz), 127.48 (q, $^3J_{C\text{-}F}$ = 5.8 Hz), 125.69 (q, $^1J_{C\text{-}F}$ = 273.5 Hz), 71.16, 60.61, 57.94, 47.84, 40.59 (q, $^4J_{C\text{-}F}$ = 3.1 Hz), 39.25, 32.89, 30.51, 15.68; ^{19}F NMR (CD_3OD, 470 MHz) δ: -61.95.

General procedure vi. Amide coupling with Boc-L-*tert*-leucine and deprotection - Synthesis of intermediates of compounds S6a-d and S12a-b

Same as "general procedure iii", just replacing the Boc-L-hydroxyproline with Boc-L-*tert*-leucine. All the crude intermediates prepared at this step were directly used in the next steps without further purification and characterization after deprotection of the Boc group.

General procedure vii. Amine trifluoroacetylation – Synthesis of S10a-b and S12a-b

To a solution of the amine (1 equiv.) in dry MeOH (1 ml per mmol of amine) was added triethylamine (2 equiv.). Ethyltrifluoroacetate (1.25 equiv.) was added and the reaction was stirred at room temperature for approximately 24 hours (LCMS monitoring). The solvent was evaporated under reduced pressure and the crude mixture was extracted with ethyl acetate and 1.0 M HCl solution. Combined organic phases were dried over $MgSO_4$, concentrated and purified in the acidic Gilson preparative HPLC system, yielding the desired product.

(2*S*,4*R*)-*N*-(4-bromobenzyl)-4-hydroxy-1-(2,2,2-trifluoroacetyl)pyrrolidine-2-carboxamide (S10a - spy molecule 17):

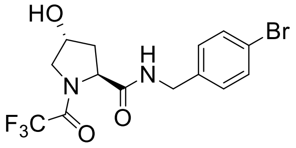

Prepared from 32 mg (0.093 mmol) of amine **S9a**, resulting in 25 mg (0.063 mmol, 68%) of the desired product as a white solid. HRMS (ESI) $[M+H]^+$ (*m/z*): Calculated for $C_{14}H_{14}BrF_3N_2O_3$: 395.0213; Observed: 395.0230; ^1H NMR (CD_3OD, 500 MHz) δ: 7.52-7.48 (m, 2H), 7.29-7.22 (m, 2H), 4.66-4.61 (t, $J_{H\text{-}H}$ = 8.6Hz, 1H), 4.57-4.54 (m, 1H), 4.50-4.30 (m, 2H), 3.88-3.84 (dd, $J_{H\text{-}H}$ = 3.5, 11.5 Hz, 1H), 3.82-3.78 (dd, $J_{H\text{-}H}$ = 1.5, 11.5 Hz, 1H), 2.48-2.29 (ddt, $J_{H\text{-}H}$ = 1.8, 7.8, 13.2, 1H), 2.24-2.04 (ddd, $J_{H\text{-}H}$ = 4.3, 9.3, 13.2 Hz, 1H); ^{13}C NMR (CD_3OD, 125

MHz) δ: 173.19, 157.56 (q, $^{2}J_{C\text{-}F}$ = 37.11 Hz), 139.11, 132.69, 130.44, 121.98, 117.73 (q, $^{1}J_{C\text{-}F}$ = 286.8 Hz), 71.08, 61.89, 57.21 (q, $^{4}J_{C\text{-}F}$ = 3.0 Hz), 43.53, 38.57; ^{19}F NMR (CD_3OD, 470 MHz) δ: -72.35 (CF_3).

(2*S*,4*R*)-4-hydroxy-*N*-(4-(4-methylthiazol-5-yl)benzyl)-1-(2,2,2-trifluoroacetyl)pyrrolidine-2-carboxamide (S10b - spy molecule 18):

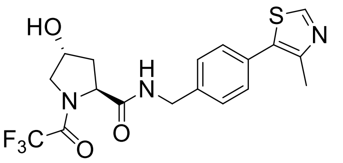

Prepared from 28 mg (0.079 mmol) of amine **S9b**, resulting in 19 mg (0.046 mmol, 58%) of the desired product as a white solid. HRMS (ESI) $[M+H]^+$ (*m/z*): Calculated for $C_{18}H_{19}F_3N_3O_3S$: 414.1094; Observed: 414.1117; ^{1}H NMR (CD_3OD, 500 MHz) δ: 8.91 (s, 1H), 7.50-7.41 (m, 4H), 4.69-4.64 (d, $J_{H\text{-}H}$ = 8.5 Hz, 1H), 4.59-4.40 (m, 3H), 3.89-3.85 (dd, $J_{H\text{-}H}$ = 3.6, 11.5 Hz, 1H), 3.83-3.79 (dd, $J_{H\text{-}H}$ = 1.4, 11.5 Hz, 1H), 2.54-2.50 (s, 3H), 2.37-2.31 (ddt, $J_{H\text{-}H}$ = 1.8, 7.8, 13.3 Hz, 1H), 2.14-2.07 (ddd, $J_{H\text{-}H}$ = 4.3, 9.3, 13.3 Hz, 1H); ^{13}C NMR (CD_3OD, MHz) δ: 173.23, 157.58 (q, $^{2}J_{C\text{-}F}$ = 36.8 Hz), 153.00, 149.20, 140.12, 133.48, 131.79, 130.59, 129.03, 117.75 (q, $^{1}J_{C\text{-}F}$ = 286.8 Hz), 71.10, 61.93, 57.23 (q, $^{4}J_{C\text{-}F}$ = 3.0 Hz), 43.82, 38.61, 15.89; ^{19}F NMR (CD_3OD, 470 MHz) δ: -73.74 (CF_3).

(2*S*,4*R*)-*N*-(4-bromobenzyl)-1-((*S*)-3,3-dimethyl-2-(2,2,2-trifluoroacetamido)butanoyl)-4-hydroxypyrrolidine-2-carboxamide (S12a - spy molecule 21):

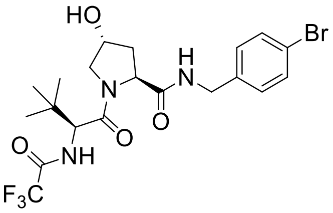

Prepared from 24 mg (0.070 mmol) of amine **S9a**, resulting in 23 mg (0.045 mmol, 65%) of the desired product as a white solid. HRMS (ESI) $[M+H]^+$ (*m/z*): Calculated for $C_{20}H_{25}BrF_3N_3O_4$: 508.1053; Observed: 508.1046; ^{1}H NMR (CD_3OD, 500 MHz) δ: 8.58 (s, NH), 7.53-7.29 (m, 4H), 4.77-4.75 (s, 1H), 4.61-4.26 (m, 4H), 3.88-3.81 (m, 2H), 2.42-2.22 (m, 1H), 2.12-2.06 (ddd, $J_{H\text{-}H}$ = 4.4, 9.4, 13.1 Hz, 1H), 1.10-1.04 (s, 9H); ^{13}C NMR (CD_3OD, 125 MHz) δ: 174.38, 170.74, 158.81 (q, $^{2}J_{C\text{-}F}$ = 37.6 Hz), 139.34, 132.57, 130.46, 121.82, 117.58 (q, $^{1}J_{C\text{-}F}$ = 286.3 Hz), 71.17, 60.95, 59.62, 58.30, 43.50, 39.03, 37.38, 26.93; ^{19}F NMR (CD_3OD, 470 MHz) δ: -76.65 (CF_3).

(2*S*,4*R*)-1-((*S*)-3,3-dimethyl-2-(2,2,2-trifluoroacetamido)butanoyl)-4-hydroxy-*N*-(4-(4-methylthiazol-5-yl)benzyl)pyrrolidine-2-carboxamide (S12b - spy molecule 22):

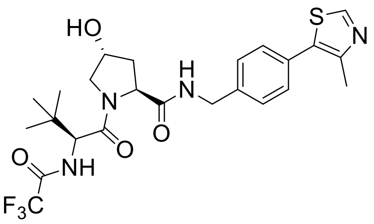

Prepared from 60 mg (0.17 mmol) of amine **S9b**, resulting in 63 mg (0.12 mmol, 71%) of the desired product as a white solid. HRMS (ESI) $[M+H]^+$ (*m/z*): Calculated for $C_{24}H_{29}F_3N_4O_4S$: 527.1934; Observed: 527.1901; ^{1}H NMR (CD_3OD, 500 MHz) δ: 8.91-8.90 (s, 1H), 7.51-7.43 (m, 4H), 4.77 (s, 1H), 4.81-4.36 (m, 4H), 3.89-3.62 (m, 2H), 2.52-2.49 (s, 3H), 2.45-2.24 (m, 1H), 2.18-2.08 (ddd, $J_{H\text{-}H}$ = 4.4, 9.3, 13.1 Hz, 1H), 1.11-1.05 (s, 9H); ^{13}C NMR (CD_3OD, 100 MHz) δ: 174.38, 170.77, 158.80 (q, $^2J_{C\text{-}F}$ = 38.4 Hz), 152.94, 149.15, 140.34, 133.50, 131.64, 130.47, 129.04, 117.58 (q, $^1J_{C\text{-}F}$ = 285.9 Hz), 71.18, 60.98, 59.64, 58.31, 43.81, 39.05, 37.40, 26.96, 15.89 ; ^{19}F NMR (CD_3OD, 470 MHz) δ: -75.18.

General procedure viii. Amide coupling with 3,3,3-fluoropropanoic acid – Synthesis of S11a-b

To a solution of amine (1 equiv.) in DMF, 3,3,3-Trifluoropropanoic acid (1 equiv.) was added and the mixture was stirred at room temperature. DIPEA (2 equiv.) was added dropwise and the mixture was stirred for 5 minutes at room temperature. HATU (1.1 equiv.) was added and the mixture was stirred at room temperature for 30-90 minutes (TLC monitoring). Water was added and the mixture was extracted with ethyl acetate. The combined organic phases were washed with brine, dried over $MgSO_4$ and evaporated under reduced pressure to give the corresponding crude, which was purified by flash column chromatography with an increasing gradient of DCM and 20% MeOH in DCM to yield the desired product.

(2*S*,4*R*)-*N*-(4-bromobenzyl)-4-hydroxy-1-(3,3,3-trifluoropropanoyl)pyrrolidine-2-carboxamide (S11a - spy molecule 19):

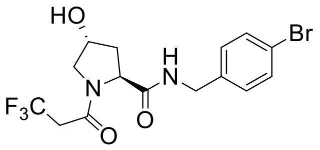

Prepared from 37 mg (0.107 mmol) of amine **S9a**, resulting in 33 mg (0.081 mmol, 75%) of the desired product as a white solid. HRMS (ESI) $[M+H]^+$ (*m/z*): Calculated for $C_{15}H_{17}BrF_3N_2O_3$: 409.0369; Observed: 409.0389; ^{1}H NMR (CD_3OD, 500 MHz) δ: 7.52-7.47 (m, 2H), 7.29-7.25 (m, 2H), 4.59-4.33 (m, 4H), 3.81-3.77 (dd, $J_{H\text{-}H}$ = 4.3, 11.0 Hz, 1H), 3.61-3.58 (m, 1H), 3.58-2.93 (m, 2H), 2.45-2.24 (m, 1H), 2.20-2.05 (ddd, $J_{H\text{-}H}$ = 4.7, 8.2, 13.2 Hz, 1H); ^{13}C NMR (CD_3OD, 125 MHz) δ: 174.37, 165.27 (q, $^3J_{C\text{-}F}$ = 3.5 Hz), 139.20, 132.66, 130.43, 125.90 (q, $^1J_{C\text{-}F}$ = 275.3 Hz), 121.89, 70.83, 60.70, 56.98, 43.48, 39.75 (q, $^1J_{C\text{-}F}$ = 28.9 Hz), 39.39; ^{19}F NMR (CD_3OD, 470 MHz) δ: -64.04 (CF_3).

(2*S*,4*R*)-4-hydroxy-*N*-(4-(4-methylthiazol-5-yl)benzyl)-1-(3,3,3-trifluoropropanoyl)pyrrolidine-2-carboxamide (S11b - spy molecule 20):

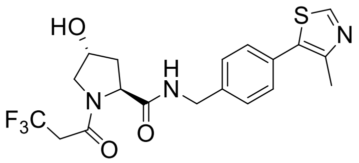

Prepared from 22 mg (0.062 mmol) of amine **S9b**, resulting in 22 mg (0.051 mmol, 83%) of the desired product as a white solid. HRMS (ESI) $[M+H]^+$ (*m/z*): Calculated for $C_{19}H_{20}F_3N_3O_3S$: 428.1250; Observed: 428.1262; ^{1}H

NMR (CD_3OD, 500 MHz) δ: 8.93-8.89 (s, 1H), 7.50-7.44 (m, 4H), 4.65-4.44 (m, 4H), 3.83-3.79 (dd, $J_{H\text{-}H}$ = 4.3, 10.9 Hz, 1H), 3.78-3.58 (dd, $J_{H\text{-}H}$ = 2.0, 10.9 Hz, 1H), 3.58-2.93 (m, 2H), 2.52-2.50 (s, 3H), 2.47-2.27 (m, 1H), 2.24-2.09 (ddd, $J_{H\text{-}H}$ = 4.7, 8.2, 13.2 Hz, 1H); ^{13}C NMR (CD_3OD, 125 MHz) δ: 174.41, 165.29 (q, $^3J_{C\text{-}F}$ = 3.3 Hz), 152.98, 149.17, 140.23, 133.51, 131.70, 130.56, 129.03, 125.92 (q, $^1J_{C\text{-}F}$ = 275.3 Hz), 70.85, 60.74, 57.00, 43.78, 39.77 (q, $^2J_{C\text{-}F}$ = 29.0 Hz), 39.43, 15.88; ^{19}F NMR (CD_3OD, 470 MHz) δ: -64.04 (CF_3).

3. Protein expression, purification and biotinylation

The VHL E3 ligase is a multi-protein complex composed of five proteins: VHL protein (pVHL), elongin B (eloB), elongin C (eloC), Cullin-2 (Cul2) and Ring-box protein 1 (Rbx1).[3] Since the compounds developed in this work bind solely to the VHL protein, the VBC complex (equimolar complex of $pVHL_{54-213}$, $eloB_{1-104}$ and $eloC_{17-112}$) was used in all experiments, as it can be readily expressed in *E. coli* with high yields,[4] while the full E3 ligase would require baculovirus-insect cells expression system.[5] The expression and purification of VBC was performed as described previously by our group[2] and employed directly in all NMR experiments.

For the surface plasmon resonance (SPR) experiments, a VBC complex containing an AviTag^{TM} in the N-terminus of eloB (AviVBC) was purified using the same procedure described for VBC. The modified eloB/eloC expression plasmid was previously developed in-house by Dr. Michael Roy and kindly shared. The AviVBC complex was site-specifically biotinylated in the AviTag using the GST-BirA method previously described by Fairhead and Howarth.[6]

4. Surface plasmon resonance experiments

The SPR experiments were performed with a Biacore T200 instrument (GE Healthcare). All measurements were performed at 20 °C with buffer containing 10 mM HEPES, pH 7.5, 150 mM NaCl, 1 mM TCEP, 0.005% (v/v) Tween® 20 and 2% (v/v) dimethyl sulfoxide (DMSO). Biotinylated AviVBC (~0.5 μM) was immobilised at 22 °C onto a Series S sensor chip SA (GE Heathcare) to levels of approximately 3500-4000 response units (RU).

Solutions of each spy molecule were prepared in buffer at concentrations based on previous structure-activity relationship studies of VHL ligands (K_D expected in the nanomolar range for structures like spy molecules **16** and **22**, or in the milimolar range for spy molecules similar to **1** and **9**). From this first screen, the binding affinities were roughly estimated using the Biacore T200 evaluation software (GE Healthcare), then measurements were repeated using concentrations above and below the K_D obtained in the first round to generate better curves for fitting the data accurately. Contact and dissociation times varied across the different compounds tested, but in general fast binding kinetics were observed for all compounds, fully reaching steady-state or being completely dissociated from the surface in less than sixty seconds.

Data analysis was performed using the steady state responses of the double-referenced sensorgrams (raw data subtracted from blank and reference surface injections) obtained for each concentration tested. These responses were plotted against the respective concentrations and the data fitted to a 1:1 binding model using the Biacore T200 evaluation software and the following equation:

$$R_{eq} = \frac{C \times R_{MAX}}{K_D + C} + offset$$

Where R_{eq} is the steady-state response at a given concentration C. Deviations in R_{eq} were corrected by adding an 'offset' term to the equation. K_D is the dissociation constant to be determined and R_{MAX} is the maximum response expected for a given compound according to the equation below:

$$R_{MAX} = n \times R_{Protein} \frac{MW_{Compound}}{MW_{Protein}}$$

Where n is the stoichiometry of the interaction (in this case, n = 1) and $R_{Protein}$ is the immobilization level of protein. $MW_{Compound}$ and $MW_{Protein}$ are the molecular weights of compound and protein, respectively. Sensorgrams and fitting parameters for all spy molecules can be found in section 7.

5. Measurement of the transverse relaxation rates by ^{19}F CPMG NMR

All the NMR experiments were performed in a 500 MHz Bruker AVANCE NMR spectrometer equipped with a CPQCI-F cryoprobe. To measure the transverse relaxation rates (R_2), a solution of each spy molecule at 100 µM was prepared in 50 mM potassium phosphate monobasic (KH_2PO_4), pH 7.5, 100 mM NaCl, 1 mM TCEP, 2% (v/v) DMSO, 20% D_2O and 10 µM trifluoroacetic acid (TFA). For each solution, ^{19}F CPMG experiments (decoupled) were performed varying the total CPMG filter (50, 100, 200, 400, 800, 1200, 1600 and 3200 ms). Due to the fast relaxation of molecules **21** and **22**, these experiments were repeated with shorter CPMG filters (50, 100, 150, 200, 300, 400, 700 and 1000 ms). The fluorine peaks were integrated and plotted against the respective CPMG filters. The R_2 relaxation rates were obtained from fitting the data as an exponential decay (GraphPad Prism 6) according to the equation below: [7]

$$I(t) = I(0) \times e^{-R_2 t}$$

Where I(t) is the ^{19}F signal intensity or integral, t is the total CPMG filter in seconds, I(0) is the signal intensity when t = 0. To obtain the R_2 upon addition of protein, all the experiments above were repeated in presence of VBC at 1 µM. Experiments were performed as triplicates and the R_2 contrasts (C_2) were obtained according to the equation below: [8]

$$C_2 = \frac{{R_2}^{Observed} - {R_2}^{Free}}{{R_2}^{Observed}}$$

Where ${R_2}^{Free}$ is the R_2 obtained for the spy molecule free in solution and ${R_2}^{Observed}$ is the R_2 obtained with the spy molecule in presence of VBC. The raw data, fitting, R_2 and C_2 values for each spy molecule can be found in section 8.1. For the R_2 measurements using molecule **19** at varied concentrations of spy molecule and protein, see section 8.2. For the R_2 measurements using spy molecules **6** and **11** (used to generate figures S2, S3 and S4) see section 8.3.

6. Competition experiments by ^{19}F NMR

As full measurements of R_2 would be very time consuming, the competition experiments were performed using a single ^{19}F CPMG experiment (decoupled) per sample with a fixed CPMG delay. To determine the best CPMG delay for a given spy molecule and assay condition, the procedure described in Figure S5 was developed based on the transverse relaxation rate equations.[7] By knowing the relaxation rates of the spy molecule free in solution and in presence of protein, the CPMG delay where the difference between the NMR peaks is maximum is hereon referred as d_{max}. The d_{max} for all the conditions tested for each spy molecule can be found in section 8, together with the respective values of R_2 and C_2.

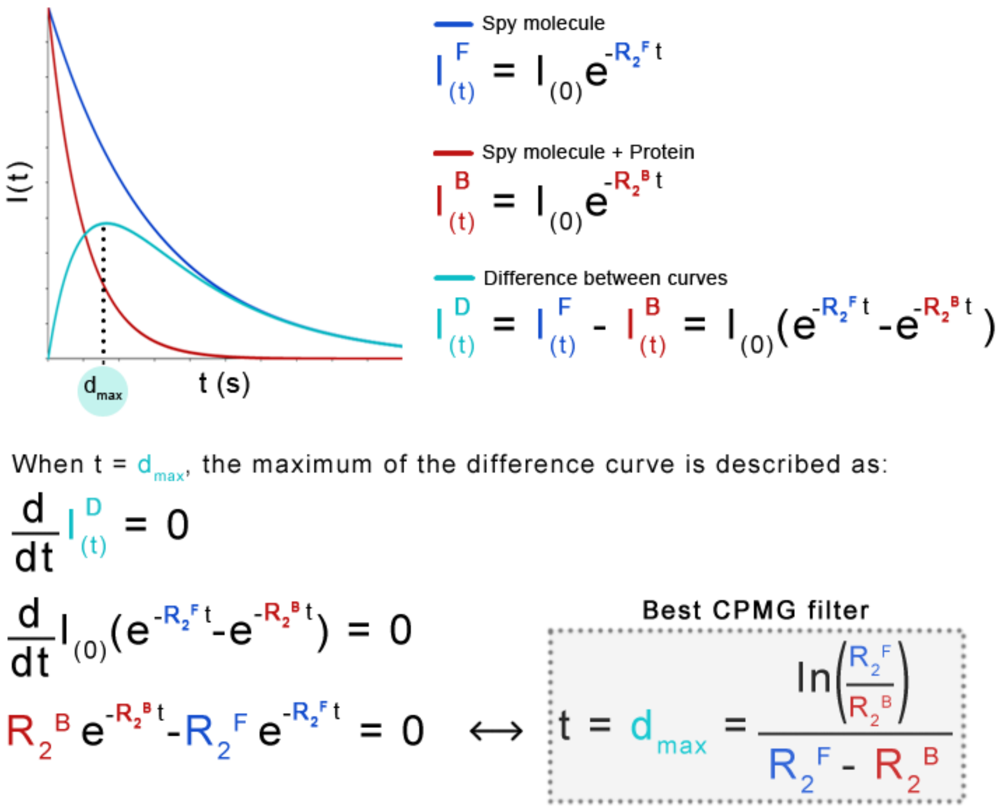

Method for selecting the best CPMG delay for competition experiments. With the transverse relaxation rates of the spy molecule free in solution (R_2^F, resulting in the blue plot) and bound to protein (R_2^B, resulting in the red plot), the difference between the two curves is described by $I(t)^D$ (cyan curve). To obtain d_{max}, the delay where the difference curve reaches its maximum, the first derivative of $I(t)^D$ was obtained and equalled to 0, subsequently isolating t (CPMG filter).

After the d_{max} for each condition was established, the competition experiments with spy molecules **6** (Figure S3), **11** (Figure S4) and **19** (Figure 5 and S5) were performed with solutions containing 50 mM potassium phosphate monobasic (KH_2PO_4), pH 7.5, 100 mM NaCl, 1 mM TCEP, 2% (v/v) DMSO, 20% D_2O and 10 µM trifluoroacetic acid (TFA). Each assay consisted of two controls:

1) Spy molecule free in solution
2) Spy molecule in presence of protein

Samples containing different concentrations of competitors were prepared in presence of spy molecule and protein and ^{19}F CPMG spectra at the respective d_{max} were collected. The displacement of the spy molecule was obtained from the equation below:

$$Displacement = \frac{I_C - I_P}{I_F - I_P} \times 100\%$$

Where I_F is the integral of the fluorine peak of the spy molecule free in solution, I_P is the integral of the fluorine peak of the spy molecule in presence of protein and I_C is the integral of the fluorine peak of the spy molecule in presence of protein and a competitor at a given concentration.

To determine the dissociation constant of a competitor, the displacement of the spy molecule was plotted against the concentration of the competitors, and then fitted to a "$\log_{10}$[Inhibitor] versus Normalised response" model using GraphPad Prism 6.0, resulting in the plots observed in Figures 5, S3 and S4. By knowing the concentrations of spy molecule, protein and K_D of the spy molecule (K_S), the IC_{50} values obtained from the fitting were converted into the K_D of competitor (K_i) using the Nikolovska-Coleska relationship.[9]

$$K_i = \frac{[C]_{50}}{\frac{[S]_{50}}{K_S} + \frac{[P]_0}{K_S} + 1}$$

Where $[C]_{50}$ and $[S]_{50}$ are, respectively, the free concentrations of competitor and spy molecule at 50% inhibition (concentration of competitor equals the IC_{50}), and $[P]_0$ is the free concentration of protein in presence of just the spy molecule. As the total concentrations of protein (P_t) and spy molecule (S_t) are known, these values can be obtained from the equations below:[9a]

$$P_0 = \frac{[P]_t - K_s - [S]_t - \sqrt[2]{(K_s + [S]_t - [P]_t)^2 + 4[P]_t K_s}}{2}$$

$$[S]_{50} = [S]_t - \frac{([P]_t - [P]_0)}{2}$$

$$[C]_{50} = IC_{50} - [P]_0 + \frac{([P]_t - [P]_0)}{2} \times \left(1 + \frac{K_S}{[S]_{50}}\right)$$

7. Surface plasmon resonance – Sensorgrams and data fitting

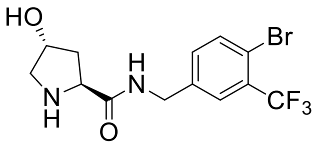

Spy molecule 1 (Compound S3a)

Blank and reference surface subtracted responses according to the concentration of spy molecule. **Theoretical R_{MAX} = 25.4**

[C] (μM)	log_{10}[C]	Response (RU)
14.0625	-4.85	2.6
28.125	-4.55	2.9
56.25	-4.25	3.3
112.5	-3.95	3.5
225	-3.65	4
450	-3.35	4.8

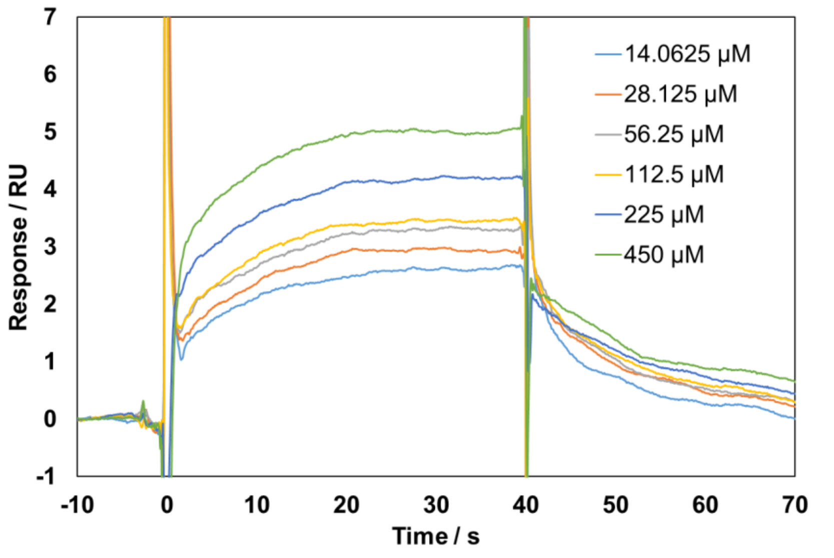

Data could not be fitted. Responses increase with concentration, but too far from the theoretical R_{MAX}. K_D >>> 1.0 mM.

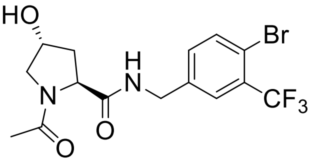

Spy molecule 2 (Compound S4a)

Blank and reference surface subtracted responses according to the concentration of spy molecule. **Theoretical R_{MAX} = 29.7**

[C] (μM)	log_{10}[C]	Response (RU)
12.5	4.90	0.1
25.0	4.60	0.8
50.0	4.30	1.5
100.0	4.00	3.0
200.0	3.70	5.5
400.0	3.40	10.6
800.0	3.10	16.3

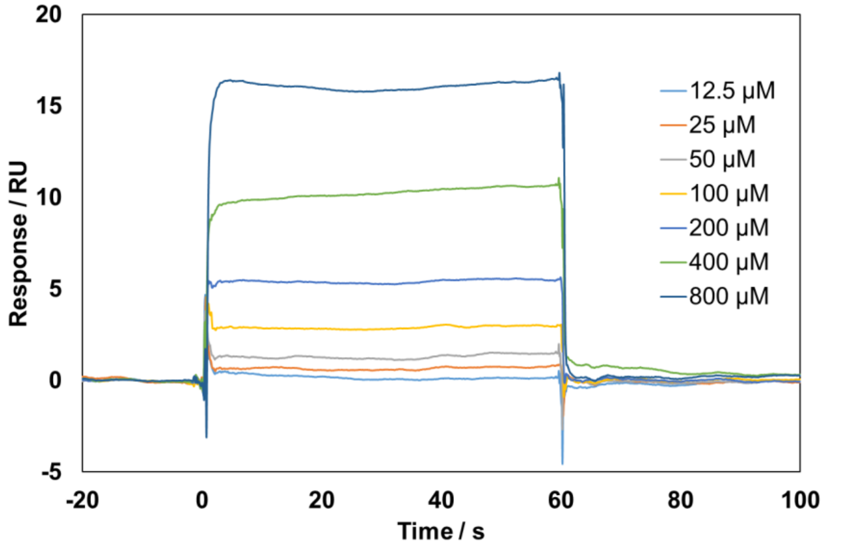

Data fitting using the Biacore T200 evaluation software. As responses were lower than the theoretical R_{MAX}, fitting was performed with a fixed R_{MAX}.

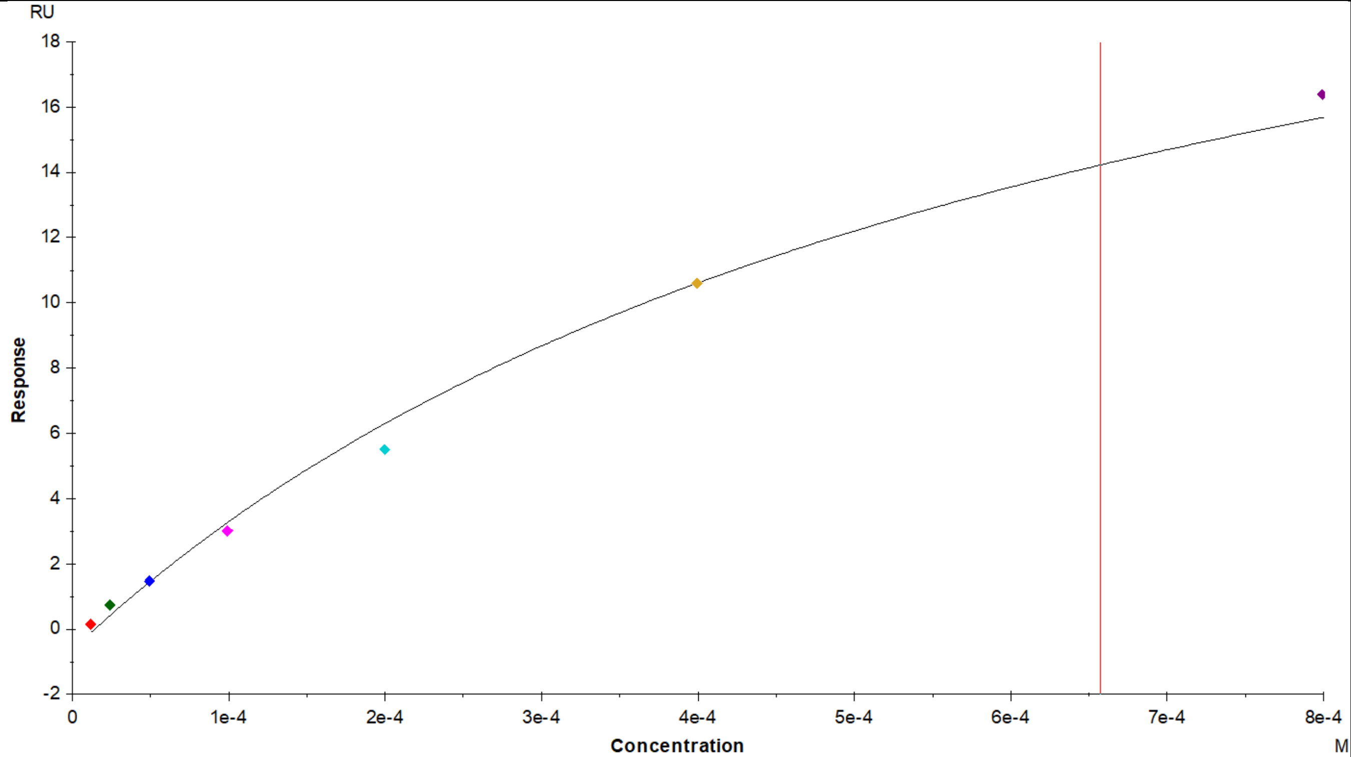

K_D (μM)	SE (K_D)	R_{max} (RU)	SE(R_{max})	offset (RU)	SE(offset)	Chi^2 (RU^2)
657.3	49.0	29.7	-	-0.62	0.34	0.262

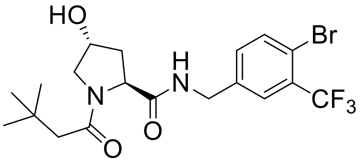

Spy molecule 3 (Compound S5a)

Blank and reference surface subtracted responses according to the concentration of spy molecule. **Theoretical R_{MAX} = 34.0**

[C] (µM)	log_{10}[C]	Response (RU)
12.5	4.90	1.6
25.0	4.60	3.0
50.0	4.30	5.3
100.0	4.00	8.8
200.0	3.70	13.0
400.0	3.40	18.1
800.0	3.10	21.9

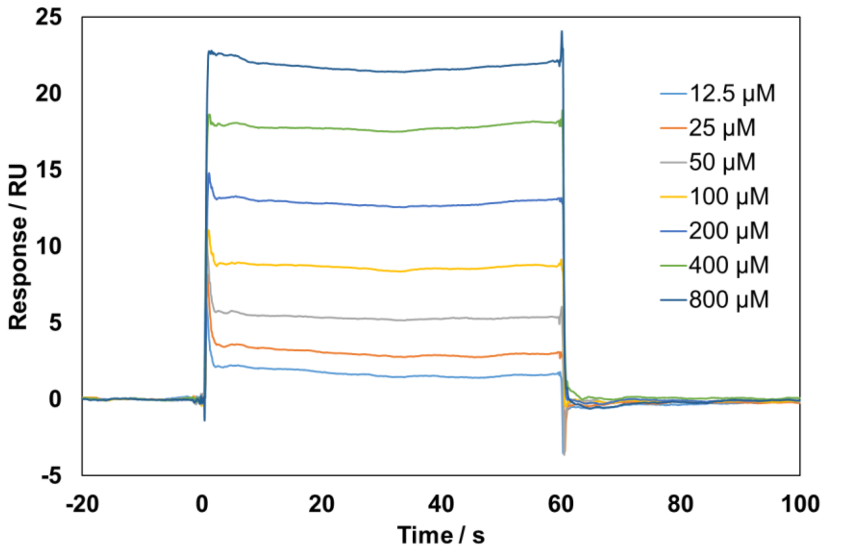

Data fitting using the Biacore T200 evaluation software. As responses were lower than the theoretical R_{MAX}, fitting was performed with a fixed R_{MAX}.

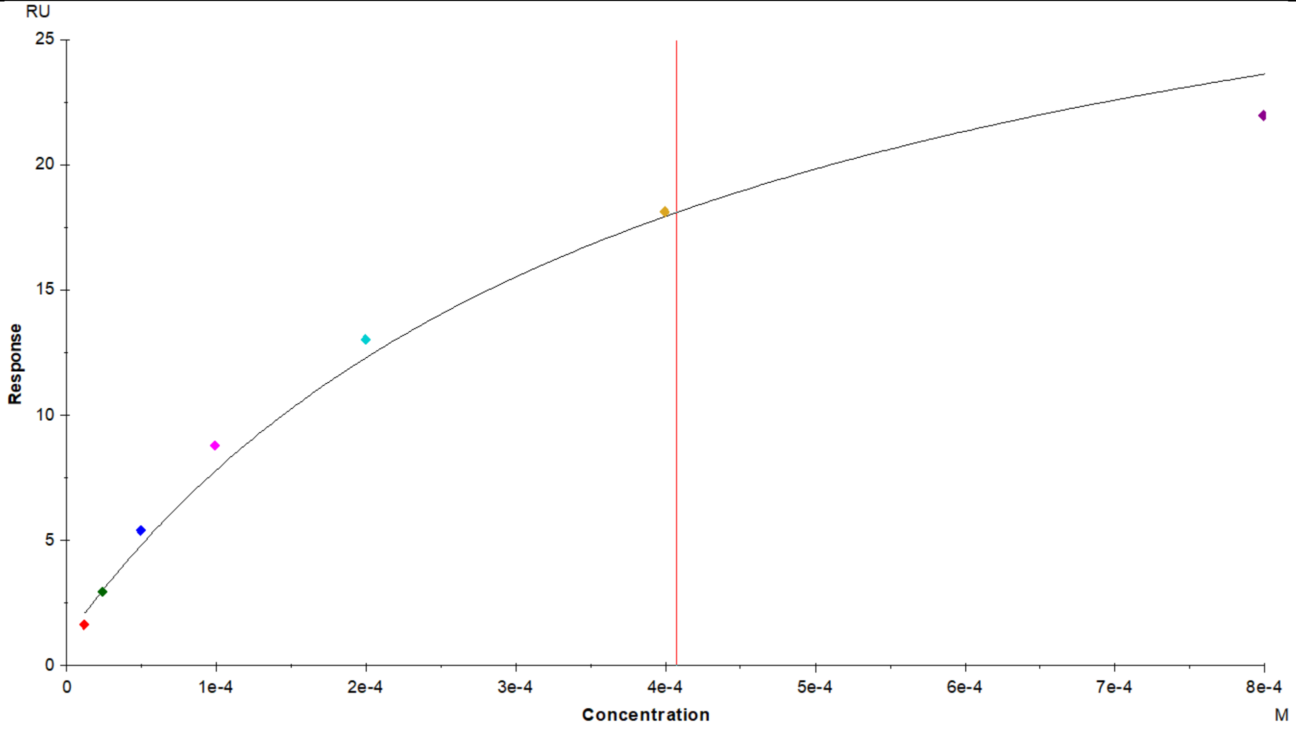

K_D (µM)	SE (K_D)	R_{max} (RU)	SE(R_{max})	offset (RU)	SE(offset)	Chi^2 (RU^2)
407.2	54.0	34.0	-	1.13	0.76	0.961

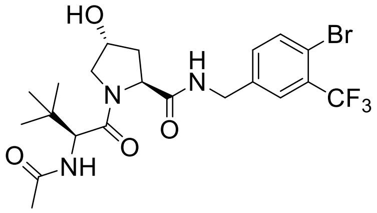

Spy molecule 4 (Compound S6a)

Blank and reference surface subtracted responses according to the concentration of spy molecule.

Theoretical R_{MAX} = 35.3

[C] (μM)	log_{10}[C]	Response (RU)
0.023	-7.64	2.9
0.069	-7.16	3.4
0.206	-6.69	4.6
0.617	-6.21	7.2
1.852	-5.73	12.2
5.556	-5.26	20.5
16.667	-4.78	27.9
50.000	-4.30	32.3

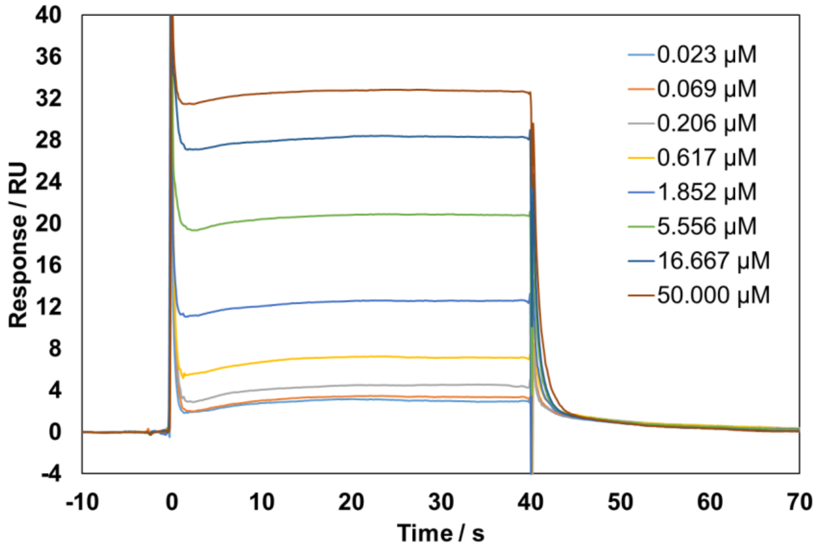

Data fitting using the Biacore T200 evaluation software:

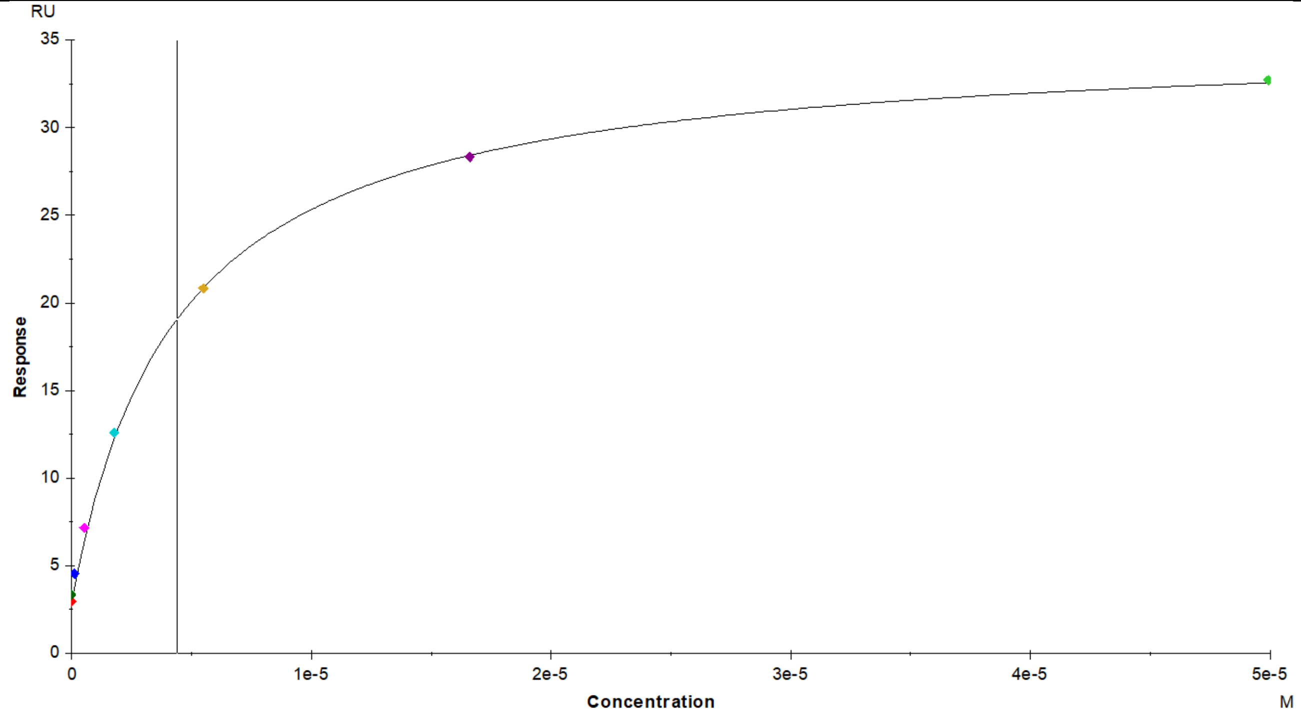

K_D (μM)	SE (K_D)	R_{max} (RU)	SE(R_{max})	offset (RU)	SE(offset)	Chi² (RU²)
4.388	0.120	32.2	0.22	3	0.11	0.0344

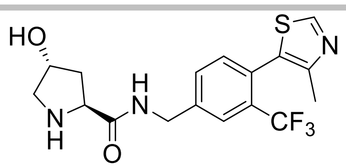

Spy molecule 5 (Compound S3b)

Blank and reference surface subtracted responses according to the concentration of spy molecule:

Theoretical R_{MAX} = 26.0

[C] (µM)	$\log_{10}$[C]	Response (RU)
14.0625	-4.85	2.7
28.125	-4.55	2.8
56.25	-4.25	3.0
112.5	-3.95	4.0
225	-3.65	5.0
450	-3.35	6.8

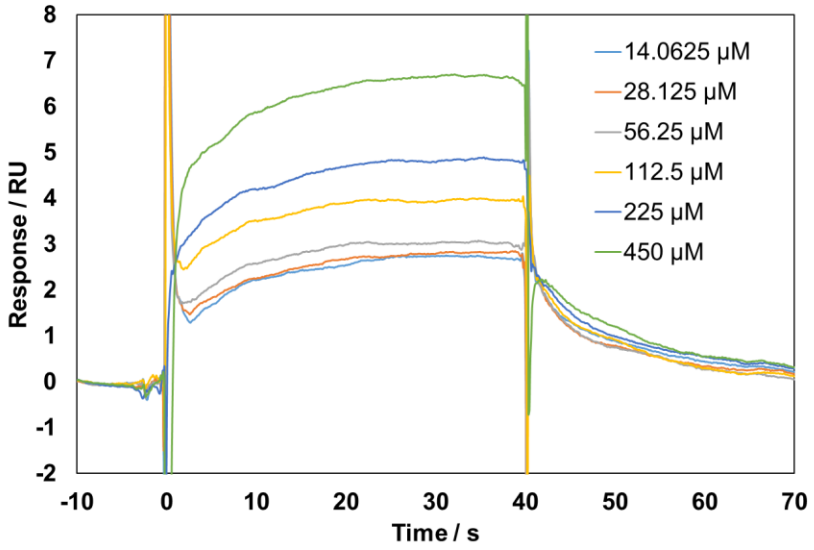

Data could not be fitted. Responses increase with concentration, but too far from the theoretical R_{MAX}. K_D >>> 1.0 mM.

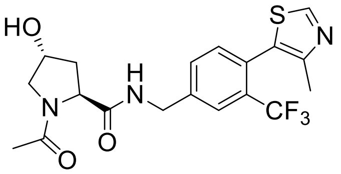

Spy molecule 6 (Compound S4b)

Blank and reference surface subtracted responses according to the concentration of spy molecule:

Theoretical R_{MAX} = 28.5

[C] (μM)	log_{10}[C]	Response (RU)
14.0625	-4.85	3.0
28.125	-4.55	4.0
56.25	-4.25	4.6
225	-3.65	9.5
450	-3.35	11.8
900	-3.05	18.4

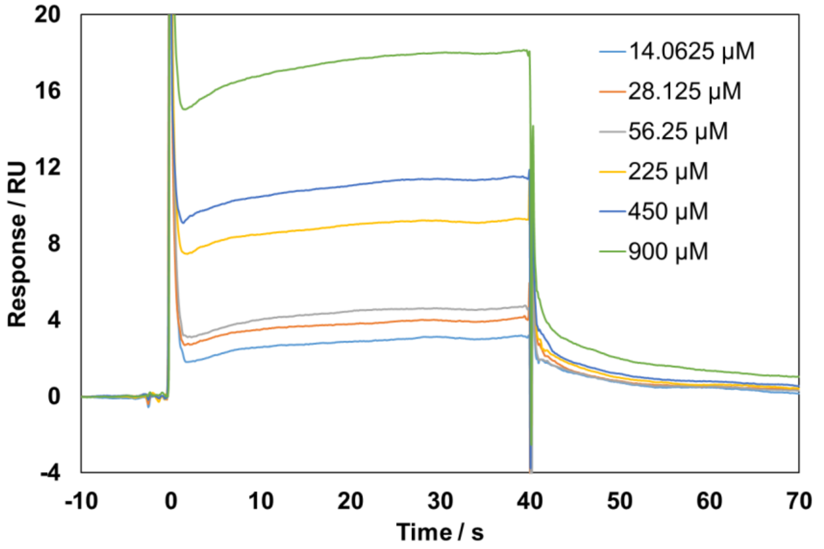

Data fitting using the Biacore T200 evaluation software. As responses were much lower than the theoretical R_{MAX}, fitting was performed with a fixed R_{MAX}:

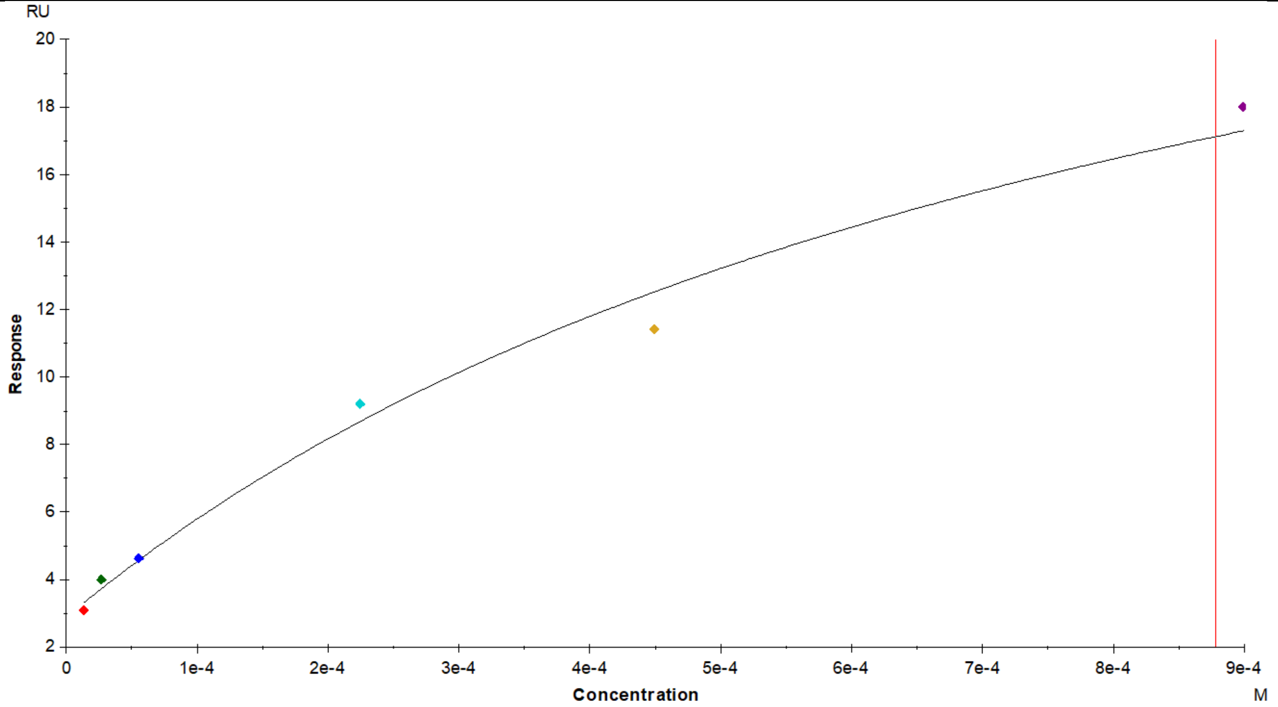

K_D (μM)	SE (K_D)	R_{max} (RU)	SE(R_{max})	offset (RU)	SE(offset)	Chi^2 (RU^2)
877.7	98.0	28.5	-	2.9	0.49	0.526

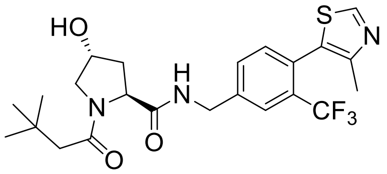

Spy molecule 7 (Compound S5b)

Blank and reference surface subtracted responses according to the concentration of spy molecule.

Theoretical R_{MAX} = 32.0

[C] (μM)	log_{10}[C]	Response (RU)
3.125	-5.51	7.2
6.250	-5.20	7.8
12.500	-4.90	10.5
25.000	-4.60	15.4
50.000	-4.30	18.8
100.000	-4.00	23.5
200.000	-3.70	29.8

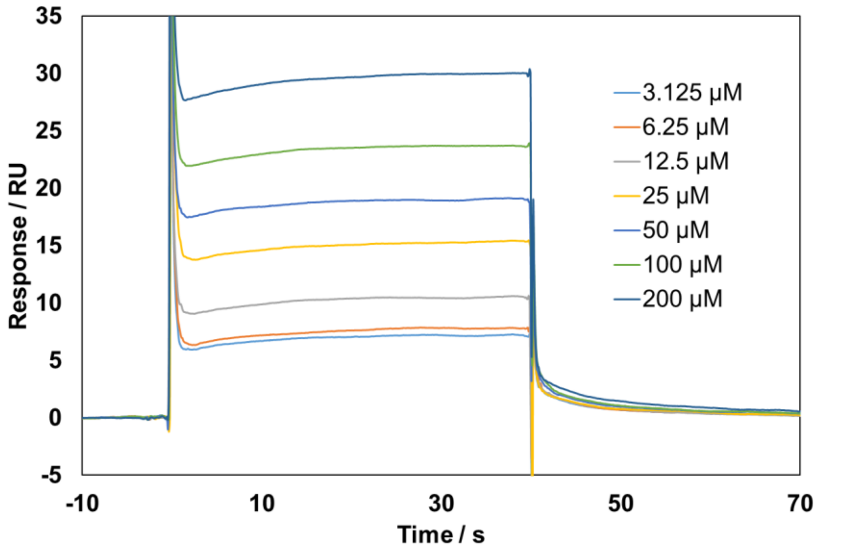

Data fitting using the Biacore T200 evaluation software:

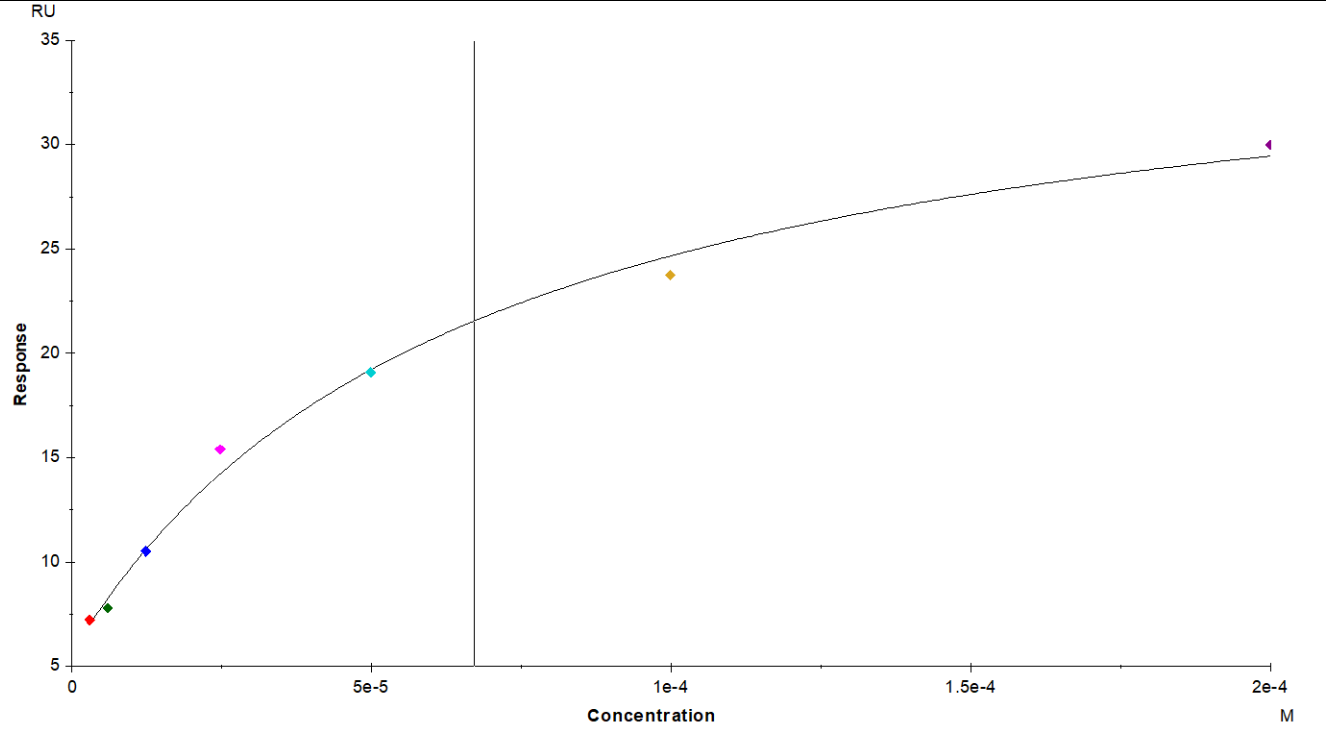

K_D (μM)	SE (K_D)	R_{max} (RU)	SE(R_{max})	offset (RU)	SE(offset)	Chi² (RU²)
67.12	14.00	31.8	2	5.6	0.79	0.704

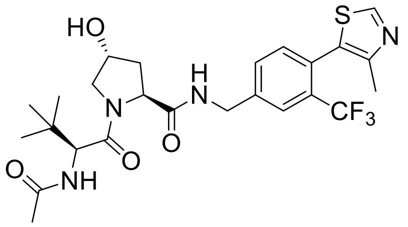

Spy molecule 8 (Compound S6b)

Blank and reference surface subtracted responses according to the concentration of spy molecule.

Theoretical R_{MAX} = 35.5

[C] (μM)	log_{10}[C]	Response (RU)
0.781	-6.11	12.6
1.563	-5.81	17.8
3.125	-5.51	24.0
6.250	-5.20	27.4
12.500	-4.90	32.0
25.000	-4.60	34.3
50.000	-4.30	38.0

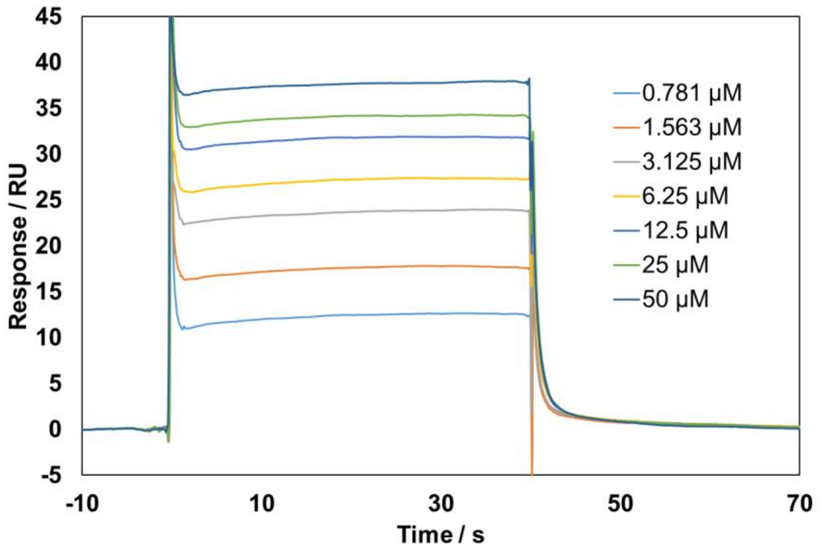

Data fitting using the Biacore T200 evaluation software:

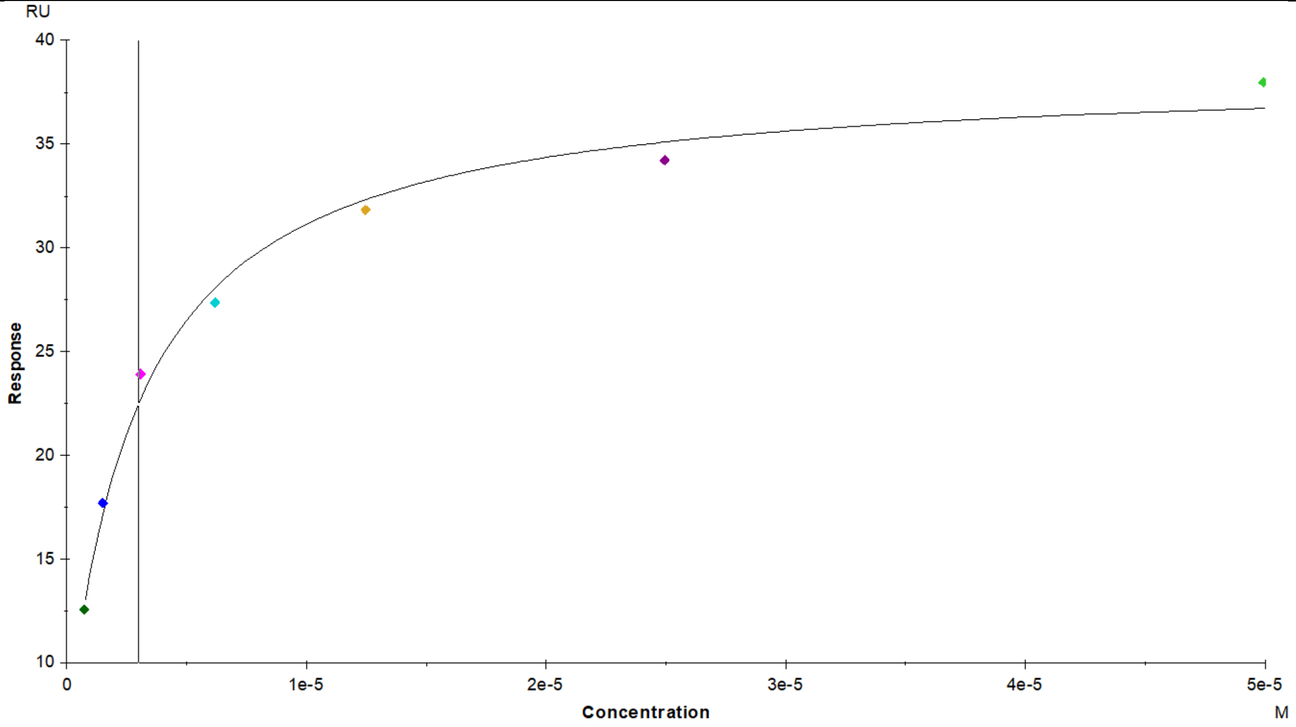

K_D (μM)	SE (K_D)	R_{max} (RU)	SE(R_{max})	offset (RU)	SE(offset)	Chi^2 (RU^2)
2.991	0.750	32.1	2.1	6.4	2.4	1.16

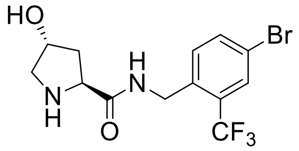

Spy molecule 9 (Compound S3c)

Blank and reference surface subtracted responses according to the concentration of spy molecule.

Theoretical R_{MAX} = 24.0

[C] (μM)	log_{10}[C]	Response (RU)
14.063	-4.85	2.5
28.125	-4.55	2.6
56.250	-4.25	3.1
112.500	-3.95	3.0
225.000	-3.65	3.8
450.000	-3.35	4.5
900.000	-3.05	7.2

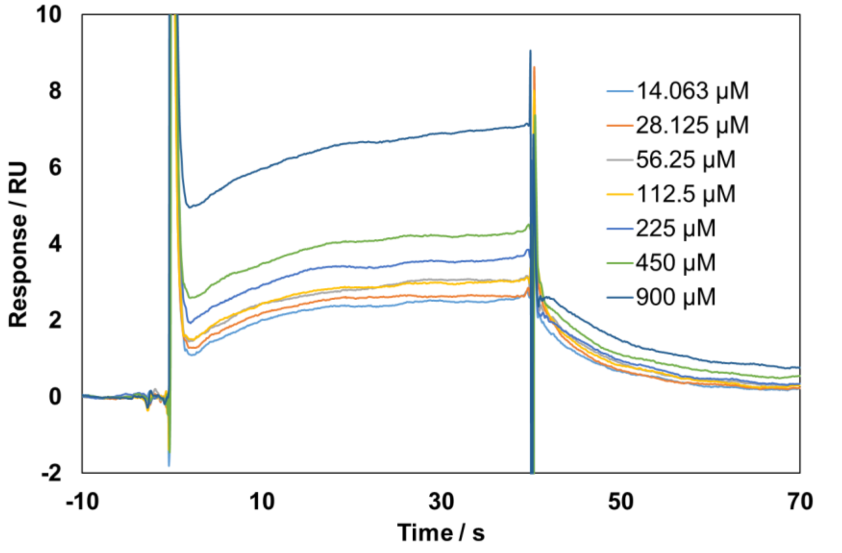

Data could not be fitted. Responses increase with concentration, but too far from the theoretical R_{MAX}. K_D >>> 1.0 mM.

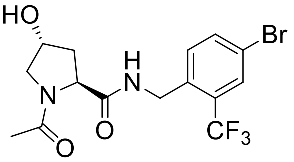

Spy molecule 10 (Compound S4c)

Blank and reference surface subtracted responses according to the concentration of spy molecule.

Theoretical R_{MAX} = 26.6

[C] (µM)	log_{10}[C]	Response (RU)
14.063	-4.85	3.2
28.125	-4.55	3.3
56.250	-4.25	4.3
112.500	-3.95	5.3
225.000	-3.65	6.3
450.000	-3.35	8.9
900.000	-3.05	13.9

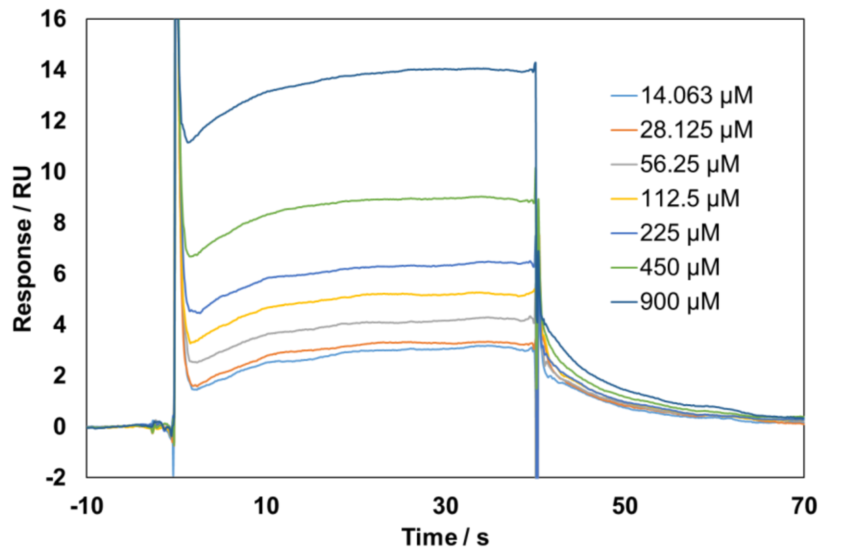

Data fitting using the Biacore T200 evaluation software. As responses were lower than the theoretical R_{MAX}, fitting was performed with a fixed R_{MAX}.

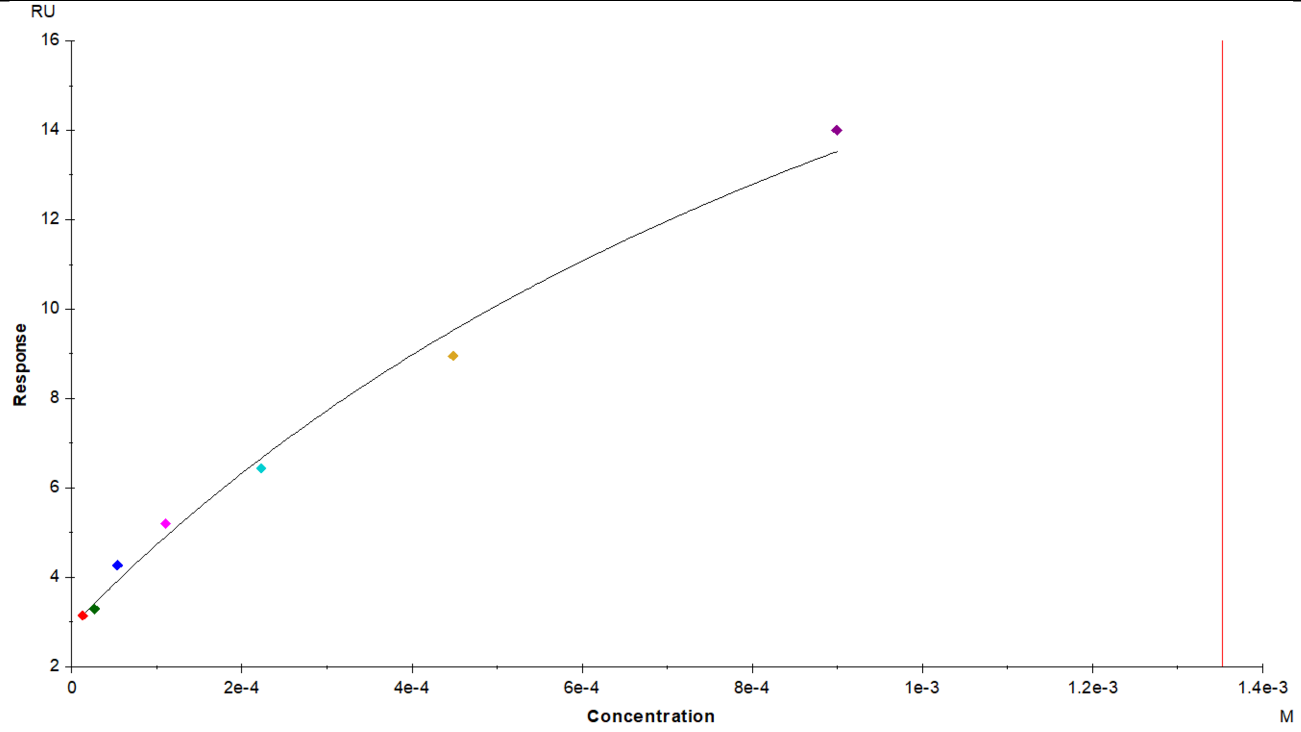

K_D (µM)	SE (K_D)	R_{max} (RU)	SE(R_{max})	offset (RU)	SE(offset)	Chi^2 (RU^2)
1352	94	26.6	-	2.9	0.24	0.159

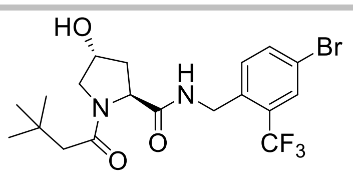

Spy molecule 11 (Compound S5c)

Blank and reference surface subtracted responses according to the concentration of spy molecule.

Theoretical R_{MAX} = 29.8

[C] (µM)	log_{10}[C]	Response (RU)
6.250	-5.20	5.5
12.500	-4.90	6.2
25.000	-4.60	8.2
50.000	-4.30	11.9
100.000	-4.00	14.4
200.000	-3.70	18.0
400.000	-3.40	21.5
800.000	-3.10	24.0

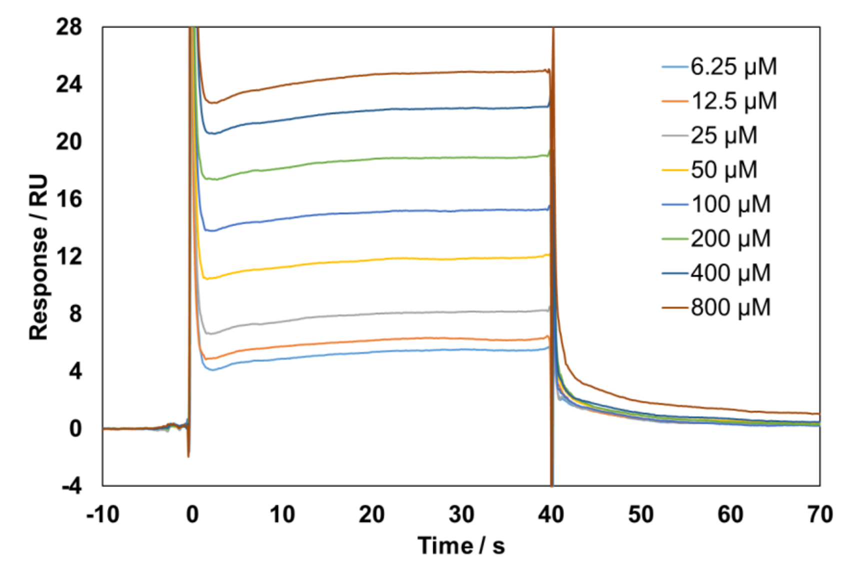

Data fitting using the Biacore T200 evaluation software:

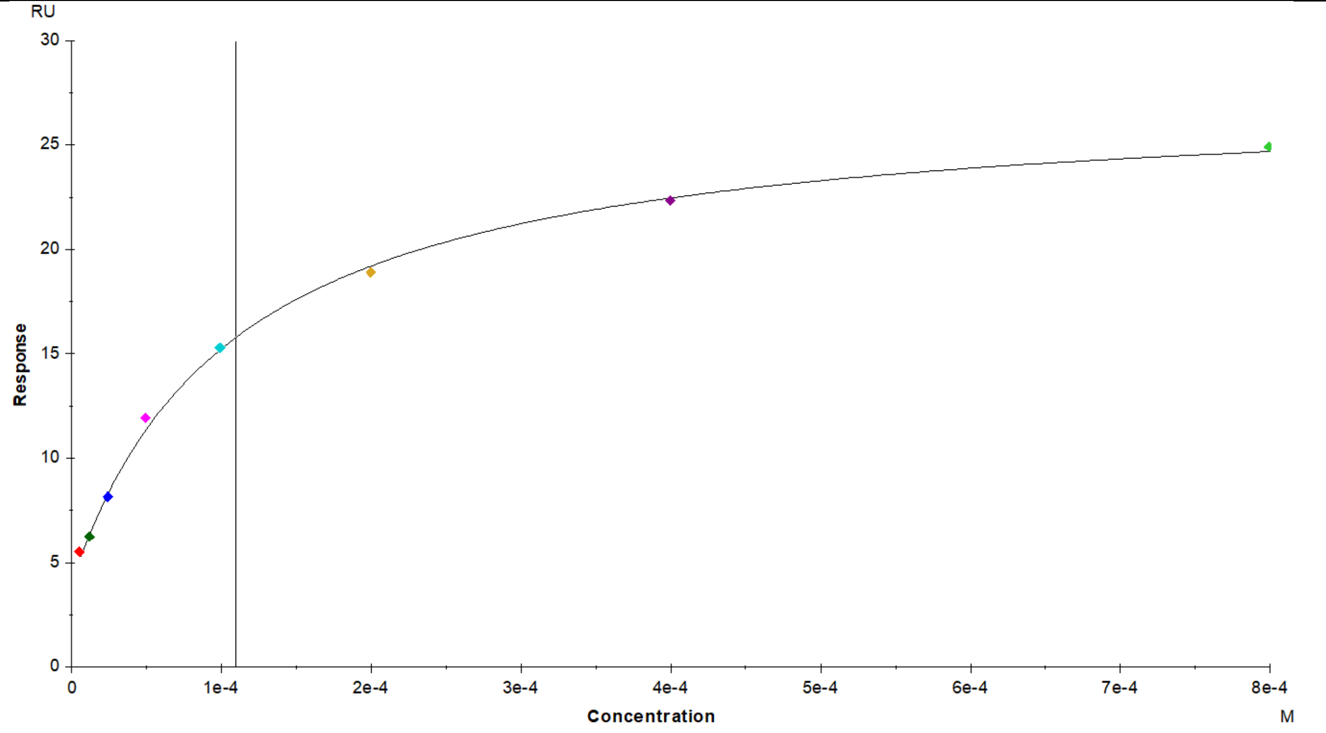

K_D (µM)	SE (K_D)	R_{max} (RU)	SE(R_{max})	offset (RU)	SE(offset)	Chi^2 (RU^2)
109.7	8.9	23.5	0.43	4	0.31	0.11

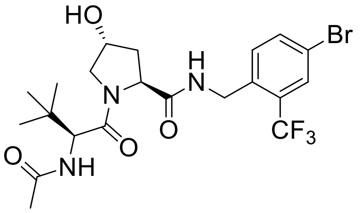

Spy molecule 12 (Compound S6c)

Blank and reference surface subtracted responses according to the concentration of spy molecule.

Theoretical R_{MAX} = 33.2

[C] (µM)	log_{10}[C]	Response (RU)
0.078	-7.11	6.2
0.156	-6.81	8.4
0.313	-6.51	10.7
0.625	-6.20	14.9
1.250	-5.90	19.2
2.500	-5.60	24.0
5.000	-5.30	26.8
10.000	-5.00	30.1

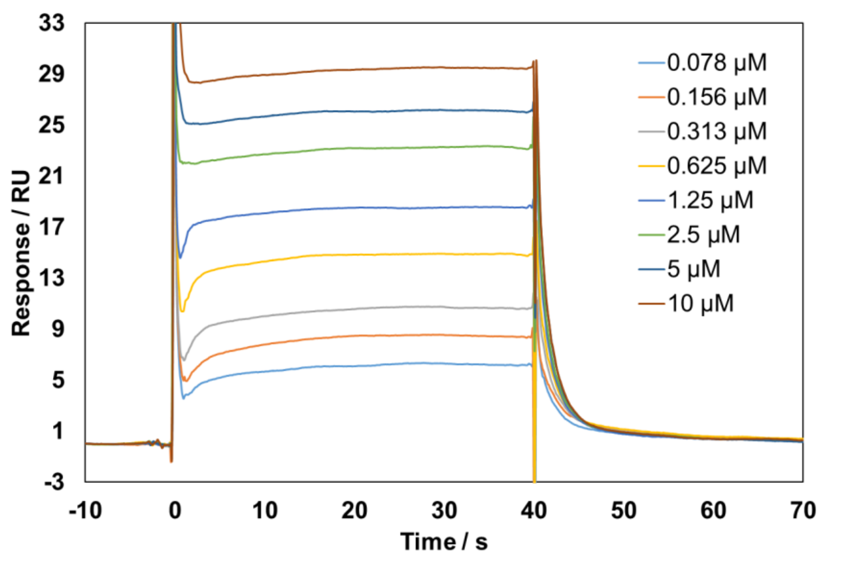

Data fitting using the Biacore T200 evaluation software:

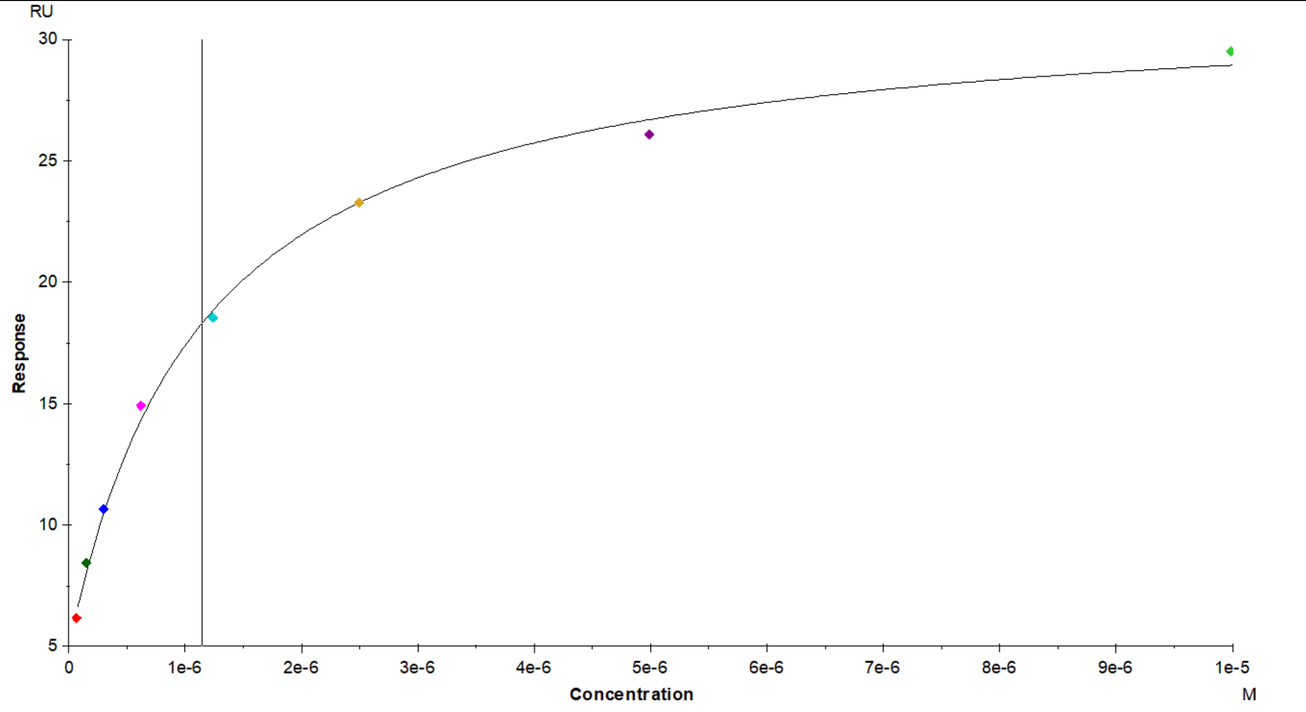

K_D (µM)	SE (K_D)	R_{max} (RU)	SE(R_{max})	offset (RU)	SE(offset)	Chi^2 (RU^2)
1.142	0.120	26.8	0.61	4.9	0.51	0.255

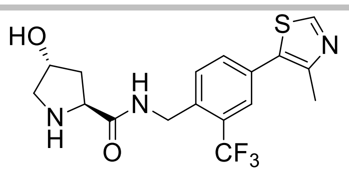

Spy molecule 13 (Compound S3d)

Blank and reference surface subtracted responses according to the concentration of spy molecule.

Theoretical R_{MAX} = 24.3

[C] (μM)	log_{10}[C]	Response (RU)
14.063	-4.85	3.8
28.125	-4.55	4.9
56.250	-4.25	6.4
112.500	-3.95	8.4
225.000	-3.65	11.1
450.000	-3.35	12.8
900.000	-3.05	22.2

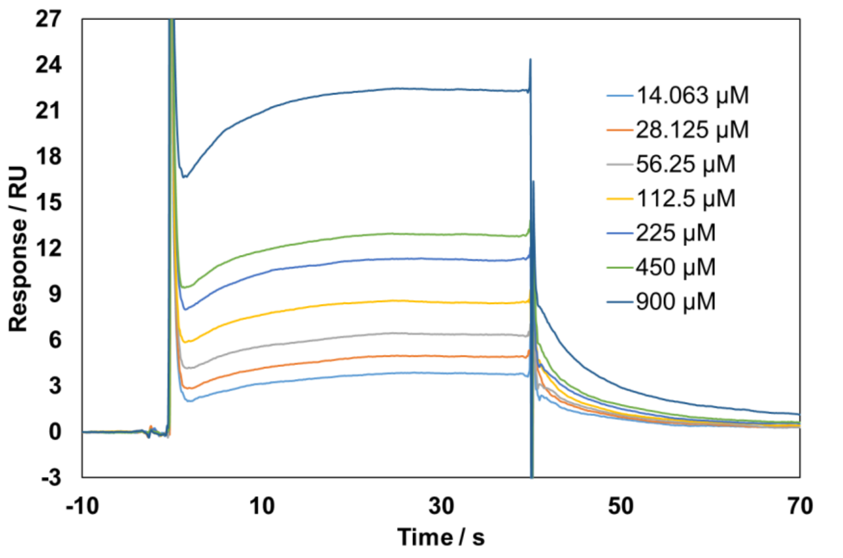

Data fitting using the Biacore T200 evaluation software: The fitted K_D presented a very large error and high R_{MAX}. The results not significant even using different fitting methods (fixing R_{MAX} or the offset). Large difference in response between concentrations of 450 and 900 μM might indicate unspecific / promiscuous binding to the protein surface. Similar result obtained from different repeats of different stocks of the compound.

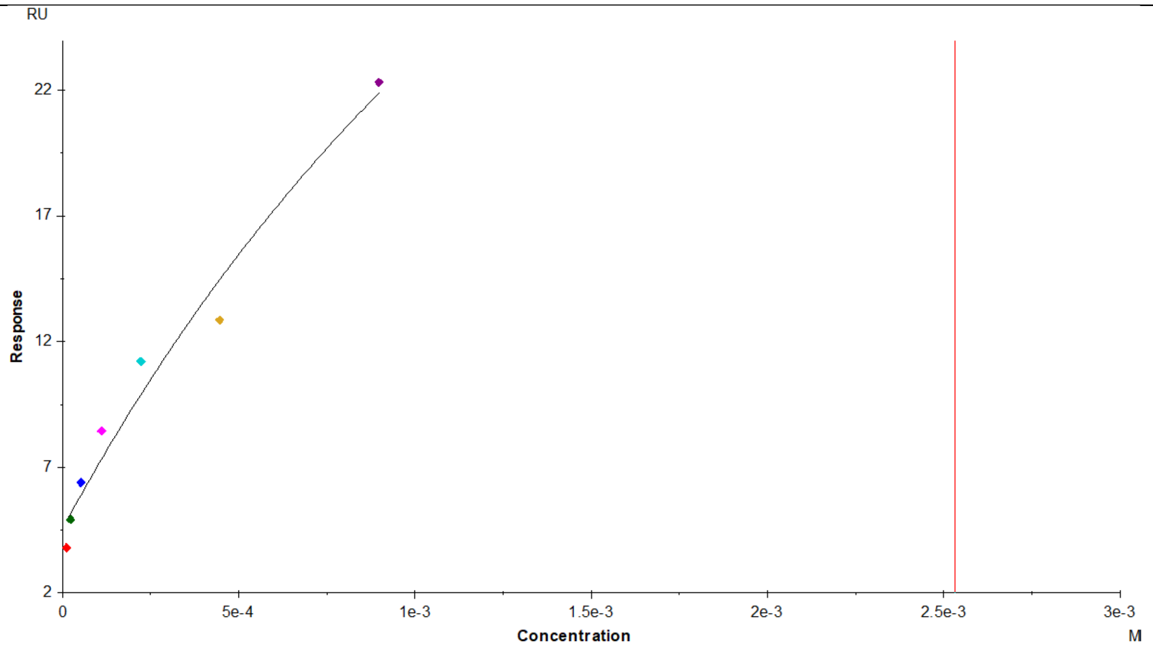

K_D (μM)	SE (K_D)	R_{max} (RU)	SE(R_{max})	offset (RU)	SE(offset)	Chi^2 (RU^2)
2531	3200	66.3	62	4.5	0.91	1.83

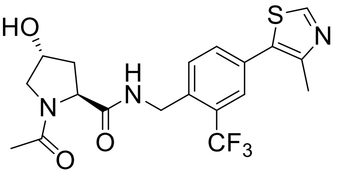

Spy molecule 14 (Compound S4d)

Blank and reference surface subtracted responses according to the concentration of spy molecule.

Theoretical R_{MAX} = 26.8

[C] (μM)	log_{10}[C]	Response (RU)
6.250	-5.20	5.5
12.500	-4.90	6.7
25.000	-4.60	10.1
50.000	-4.30	14.2
100.000	-4.00	19.8
200.000	-3.70	25.1
400.000	-3.40	31.0
800.000	-3.10	40.3

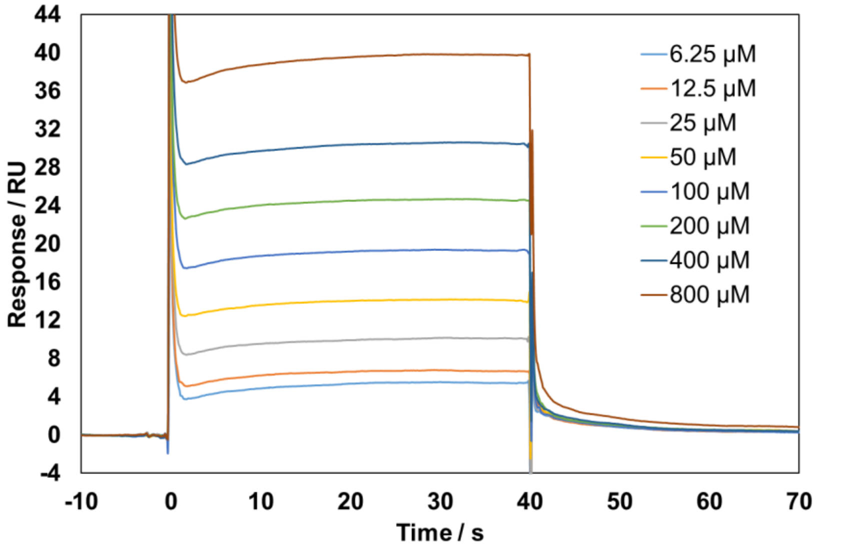

Data fitting using the Biacore T200 evaluation software: The only acceptable fitting resulted in a very large R_{MAX}. (1.6 times larger than the theoretical). Fitting with fixed theoretical R_{MAX} presented a large error in the K_D. Compound might bind to the VHL-HIF site, but also bind unspecifically / promiscuously to the protein surface.

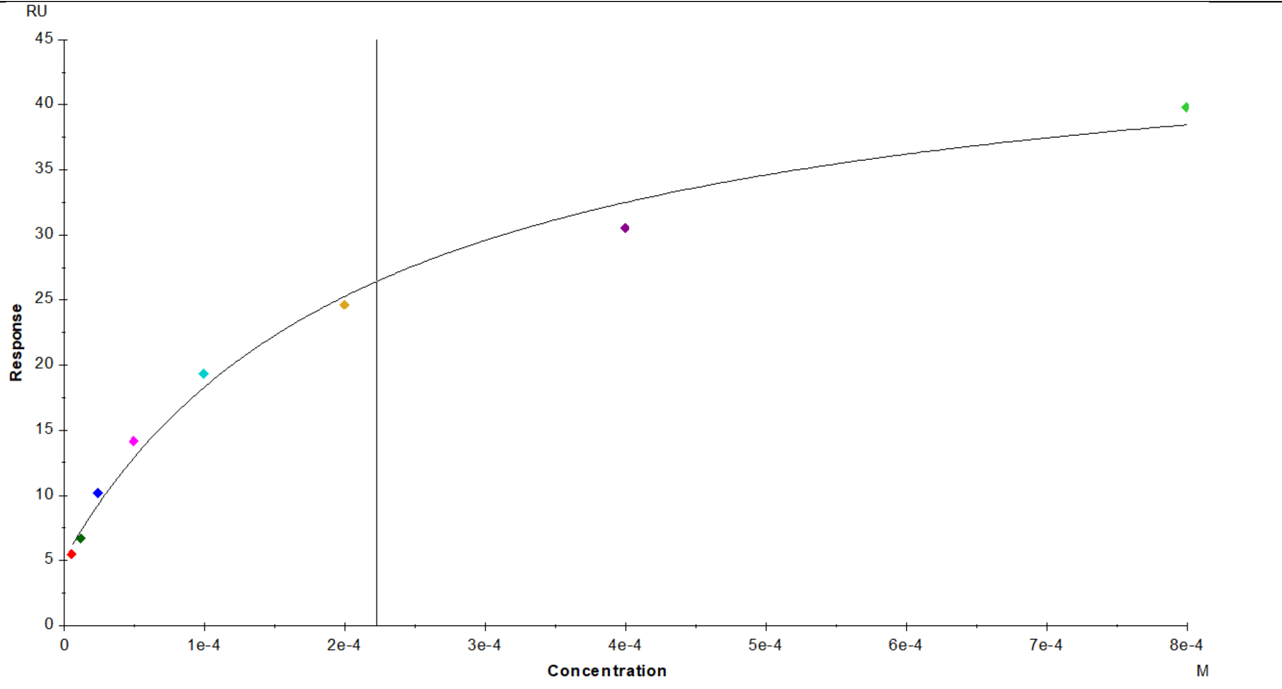

K_D (μM)	SE (K_D)	R_{max} (RU)	SE(R_{max})	offset (RU)	SE(offset)	Chi² (RU²)
222.7	51.0	42.6	2.9	5.1	1.1	2.03

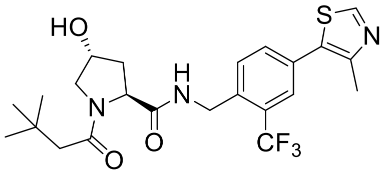

Spy molecule 15 (Compound S5d)

Blank and reference surface subtracted responses according to the concentration of spy molecule.

Theoretical R_{MAX} = 30.0

[C] (μM)	log_{10}[C]	Response (RU)
1.563	-5.81	6.3
3.125	-5.51	8.5
6.250	-5.20	10.4
12.500	-4.90	15.7
25.000	-4.60	19.0
50.000	-4.30	23.3
100.000	-4.00	28.7
200.000	-3.70	33.7

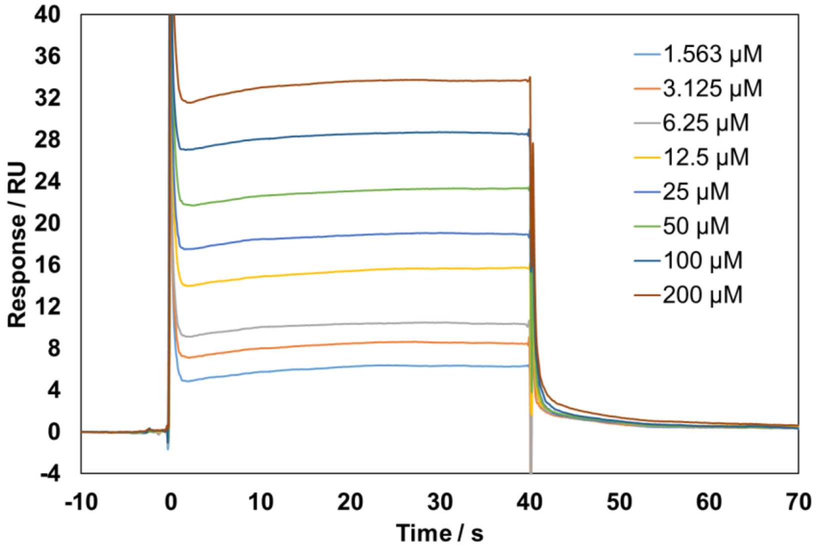

Data fitting using the Biacore T200 evaluation software:

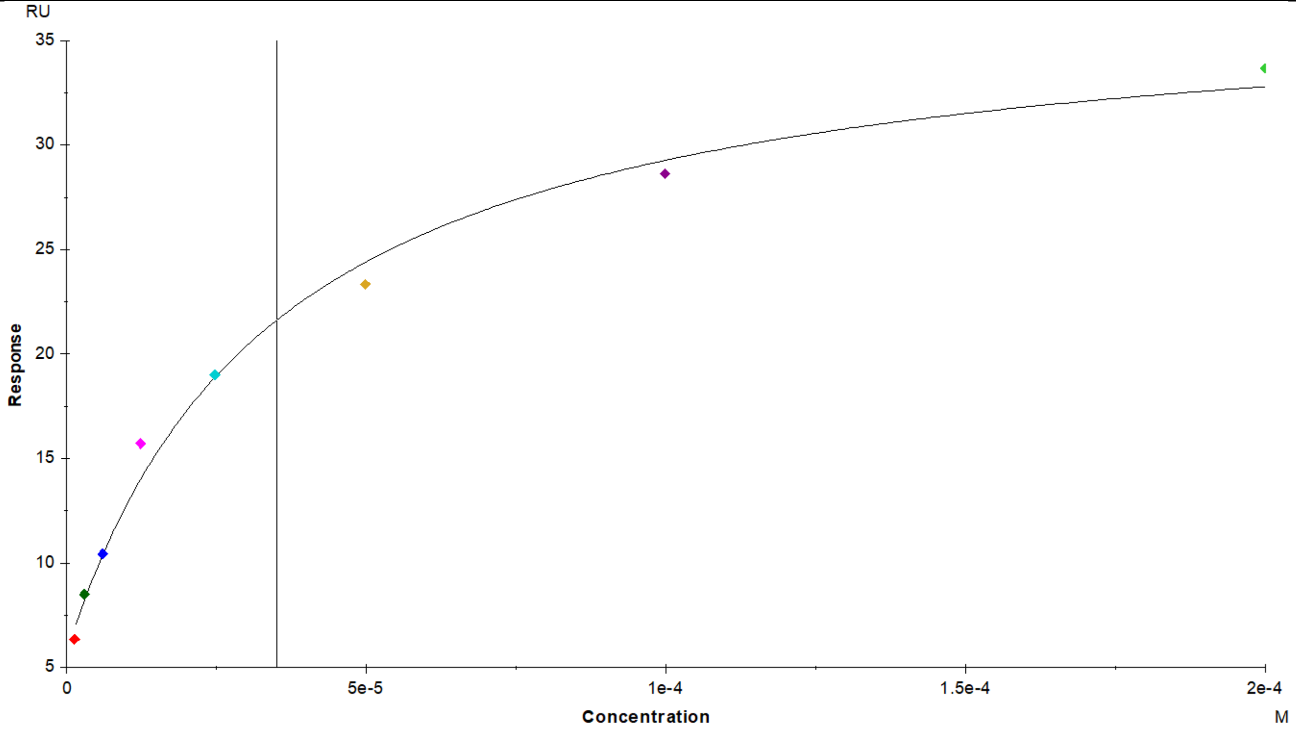

K_D (μM)	SE (K_D)	R_{max} (RU)	SE(R_{max})	offset (RU)	SE(offset)	Chi² (RU²)
35.16	7.00	31.8	1.5	5.7	0.92	1.13

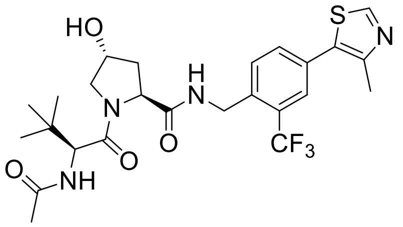

Spy molecule 16 (Compound S6d)

Blank and reference surface subtracted responses according to the concentration of spy molecule.

Theoretical R_{MAX} = 33.3

[C] (μM)	log_{10}[C]	Response (RU)
0.039	-7.41	10.4
0.078	-7.11	13.6
0.156	-6.81	18.2
0.313	-6.51	23.1
0.625	-6.20	27.2
1.250	-5.90	30.8
2.500	-5.60	33.2
5.000	-5.30	35.8

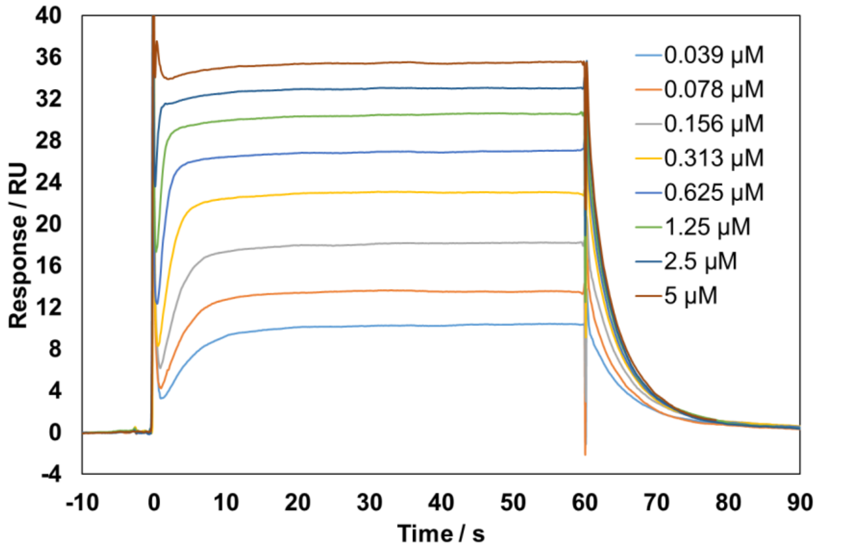

Data fitting using the Biacore T200 evaluation software:

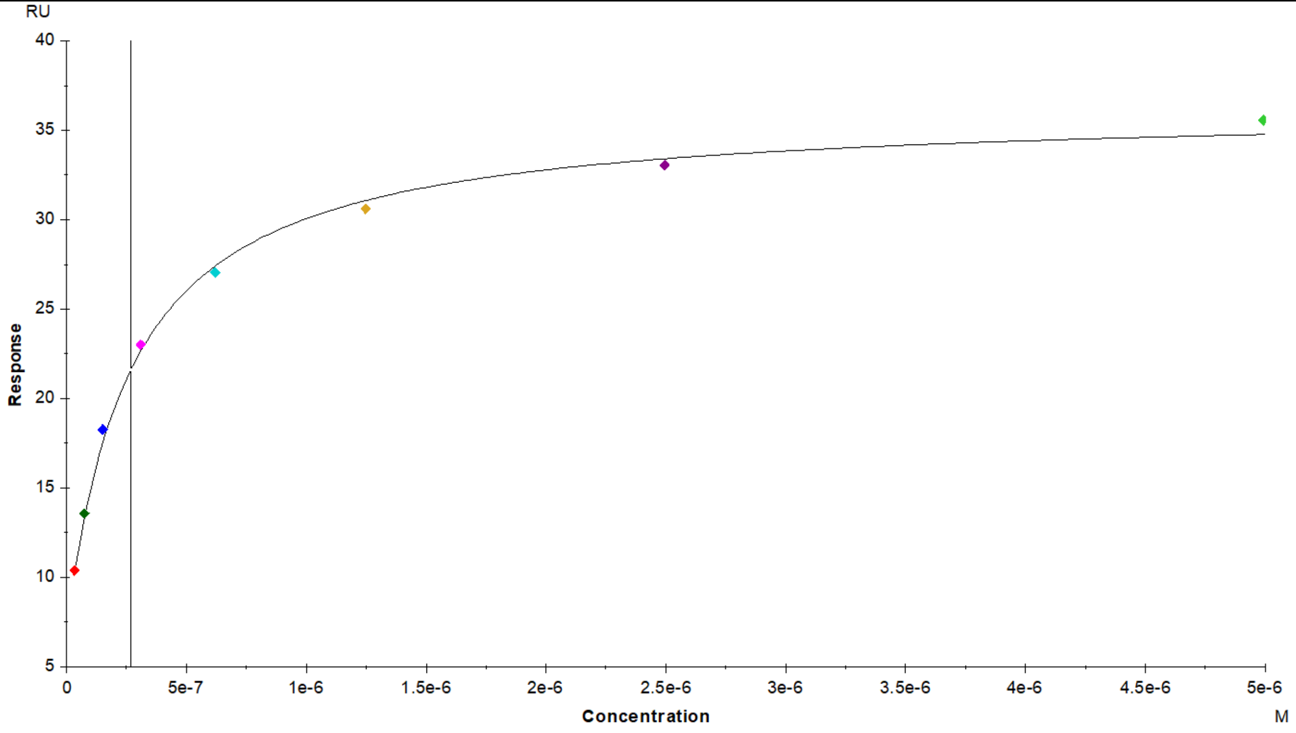

K_D (μM)	SE (K_D)	R_{max} (RU)	SE(R_{max})	offset (RU)	SE(offset)	Chi² (RU²)
0.2675	0.0300	29.3	0.74	6.9	0.82	0.319

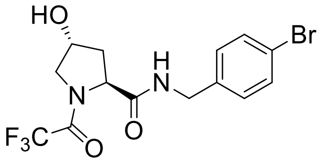

Spy molecule 17 (Compound S10a)

Blank and reference surface subtracted responses according to the concentration of spy molecule.

Theoretical R_{MAX} = 24.1

[C] (μM)	log_{10}[C]	Response (RU)
7.031	-5.15	3.9
14.063	-4.85	4.6
28.125	-4.55	5.2
56.250	-4.25	7.7
112.500	-3.95	8.7
225.000	-3.65	10.3
450.000	-3.35	13.2
900.000	-3.05	19.6

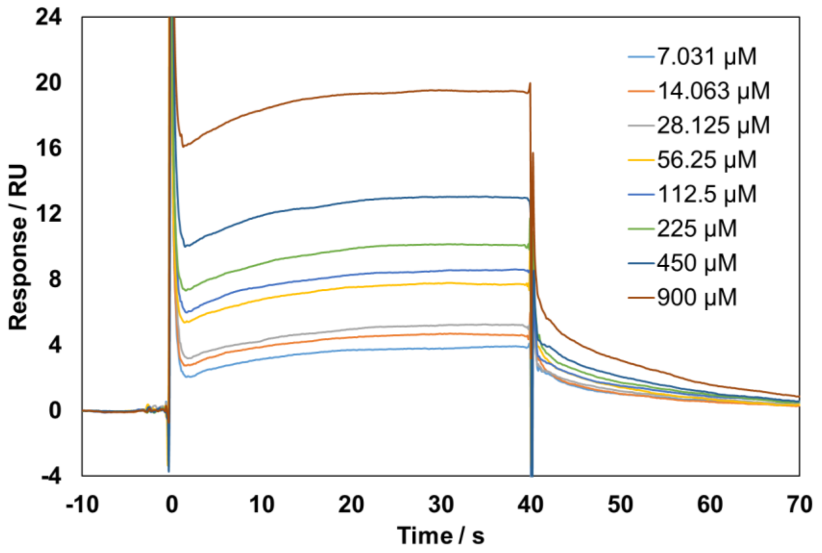

Data fitting using the Biacore T200 evaluation software. As responses were lower than the theoretical R_{MAX}, fitting was performed with a fixed R_{MAX}.

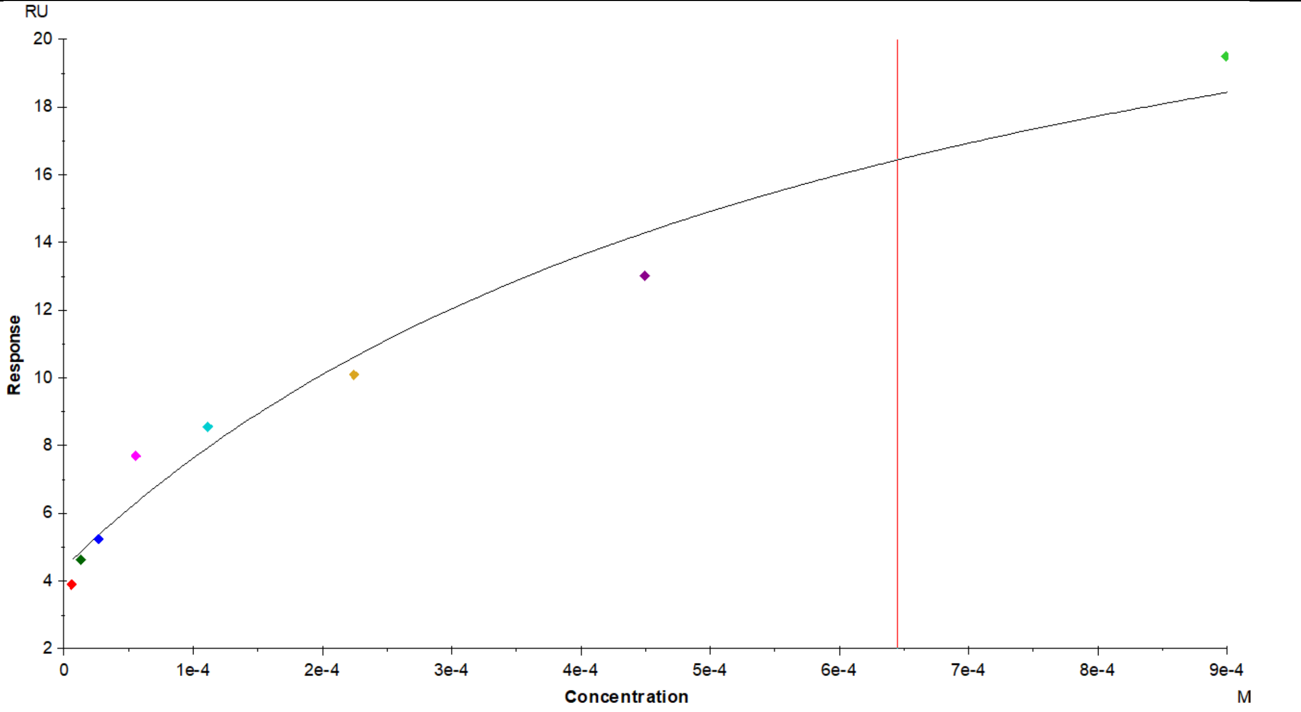

K_D (μM)	SE (K_D)	R_{max} (RU)	SE(R_{max})	offset (RU)	SE(offset)	Chi² (RU²)
645.0	100.0	24.1	-	4.4	0.58	0.985

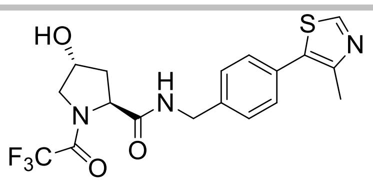

Spy molecule 18 (Compound S10b)

Blank and reference surface subtracted responses according to the concentration of spy molecule.

Theoretical R_{MAX} = 24.7

[C] (µM)	log_{10}[C]	Response (RU)
1.172	-5.93	7.1
2.344	-5.63	7.9
4.688	-5.33	11.3
9.375	-5.03	13.5
18.750	-4.73	18.3
37.500	-4.43	22.6
75.000	-4.12	26.1
150.000	-3.82	30.5

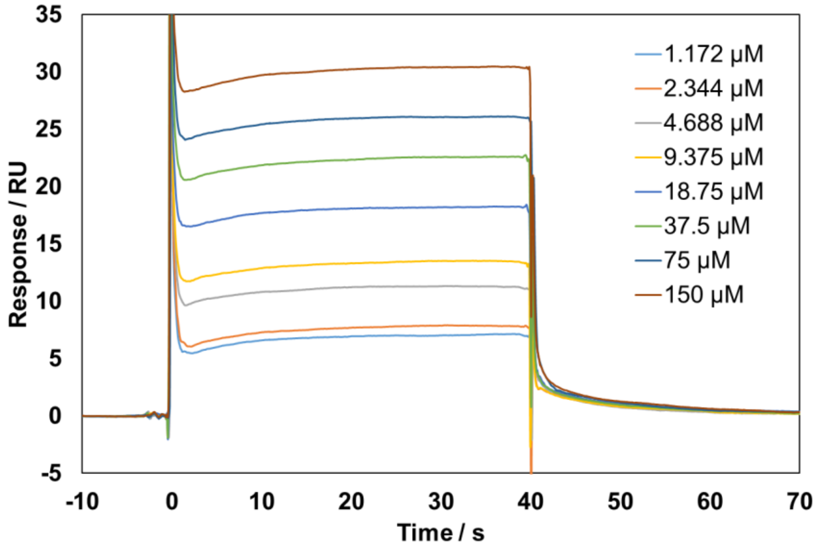

Data fitting using the Biacore T200 evaluation software:

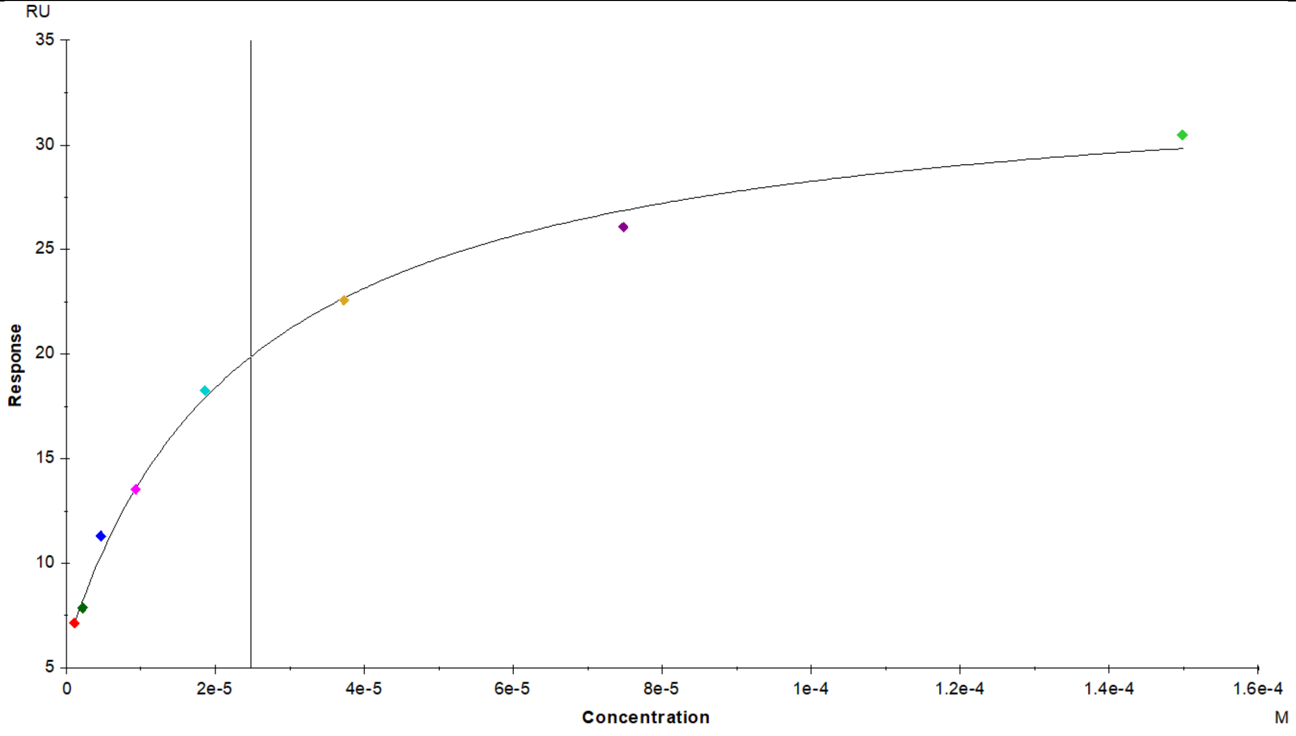

K_D (µM)	SE (K_D)	R_{max} (RU)	SE(R_{max})	offset (RU)	SE(offset)	Chi² (RU²)
24.76	3.40	27.8	0.92	6.00	0.58	0.43

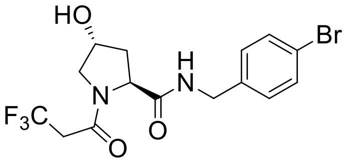

Spy molecule 19 (Compound S11a)

Blank and reference surface subtracted responses according to the concentration of spy molecule.

Theoretical R_{MAX} = 24.7

[C] (μM)	log_{10}[C]	Response (RU)
6.250	-5.20	5.3
12.500	-4.90	6.6
25.000	-4.60	8.8
50.000	-4.30	11.6
100.000	-4.00	15.3
200.000	-3.70	19.4
400.000	-3.40	21.9
800.000	-3.10	27.2

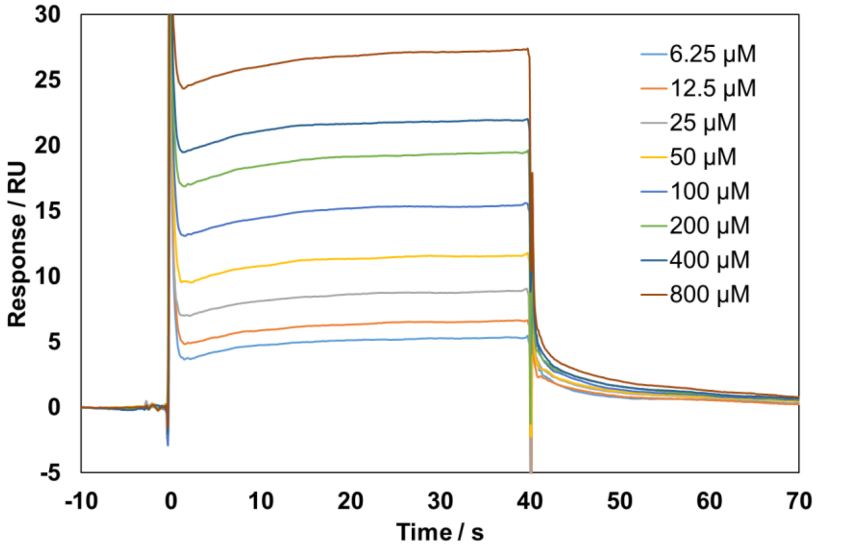

Data fitting using the Biacore T200 evaluation software:

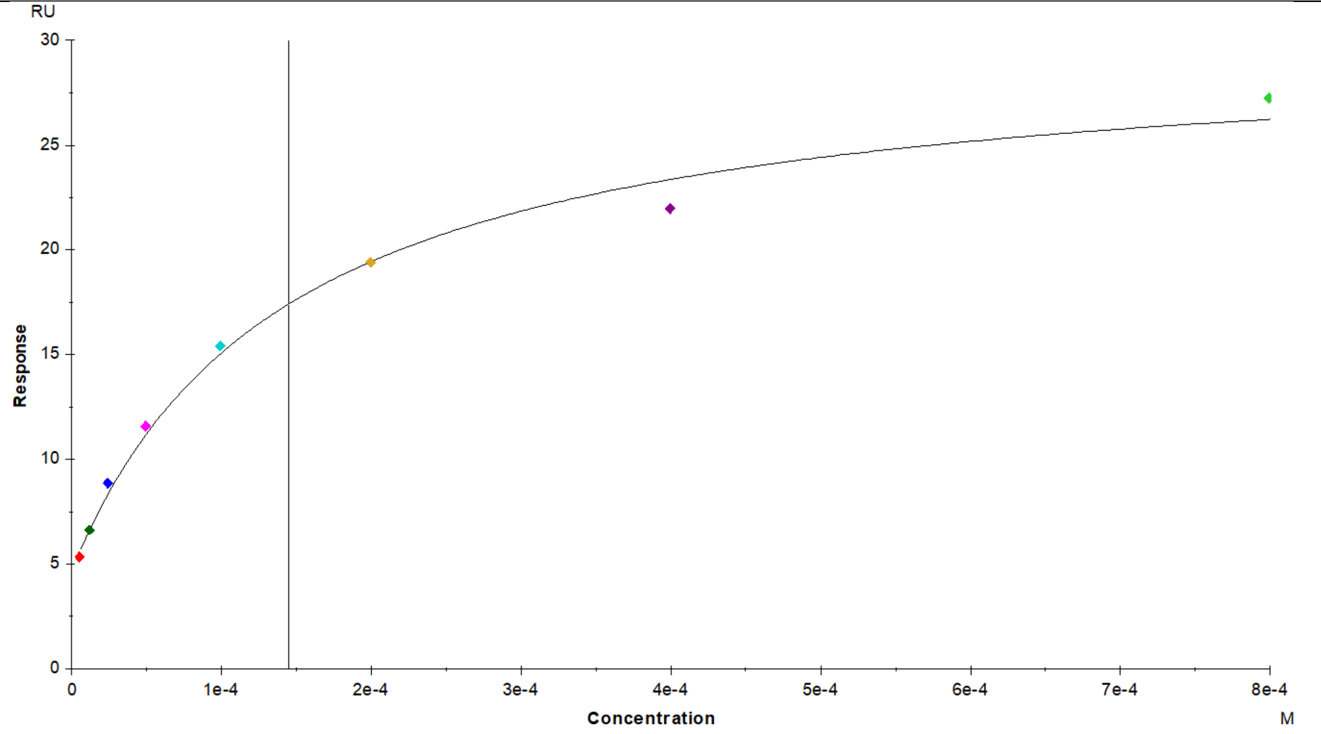

K_D (μM)	SE (K_D)	R_{max} (RU)	SE(R_{max})	offset (RU)	SE(offset)	Chi² (RU²)
144.8	29.0	25.4	1.3	4.7	0.73	0.727

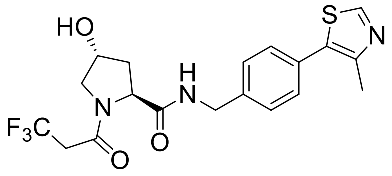

Spy molecule 20 (Compound S11b)

Blank and reference surface subtracted responses according to the concentration of spy molecule.

Theoretical R_{MAX} = 25.4

[C] (µM)	log_{10}[C]	Response (RU)
0.977	-6.01	6.4
1.953	-5.71	9.1
3.906	-5.41	11.7
7.813	-5.11	16.1
15.625	-4.81	20.0
31.250	-4.51	23.6
62.500	-4.20	27.0
125.000	-3.90	30.5

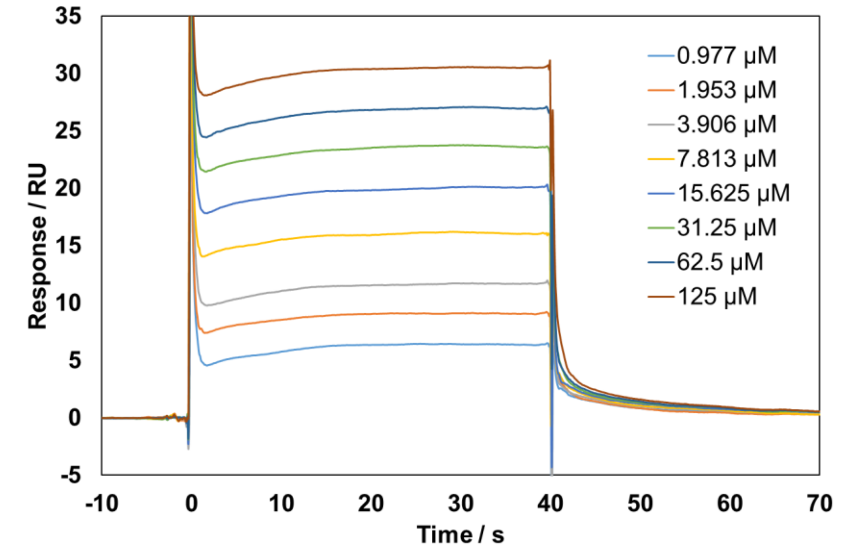

Data fitting using the Biacore T200 evaluation software:

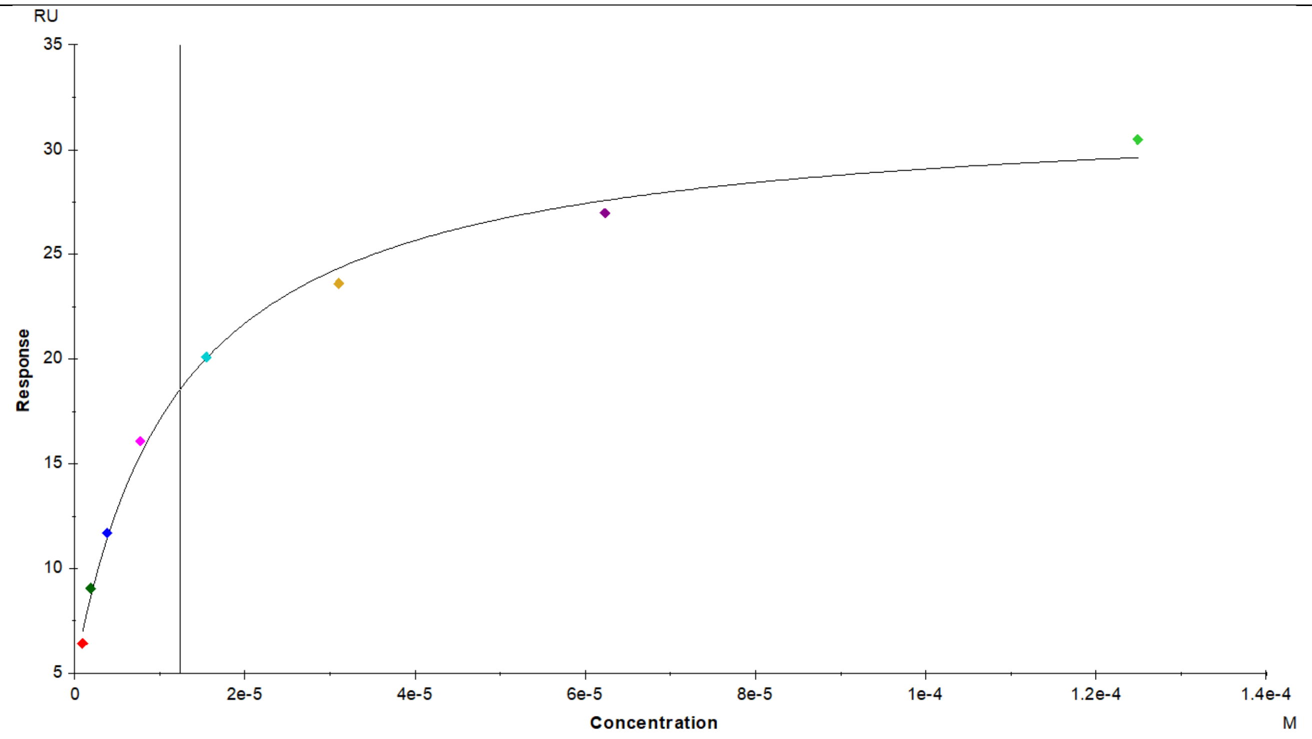

K_D (µM)	SE (K_D)	R_{max} (RU)	SE(R_{max})	offset (RU)	SE(offset)	Chi^2 (RU^2)
12.41	1.90	27.1	0.86	5.00	0.77	0.53

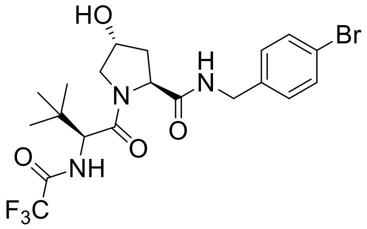

Spy molecule 21 (Compound S12a)

Blank and reference surface subtracted responses according to the concentration of spy molecule.

Theoretical R_{MAX} = 30.5

[C] (µM)	log_{10}[C]	Response (RU)
0.023	-7.63	5.9
0.047	-7.33	7.9
0.094	-7.03	10.8
0.188	-6.73	13.7
0.375	-6.43	16.9
0.750	-6.12	20.5
1.500	-5.82	24.6
3.000	-5.52	28.3

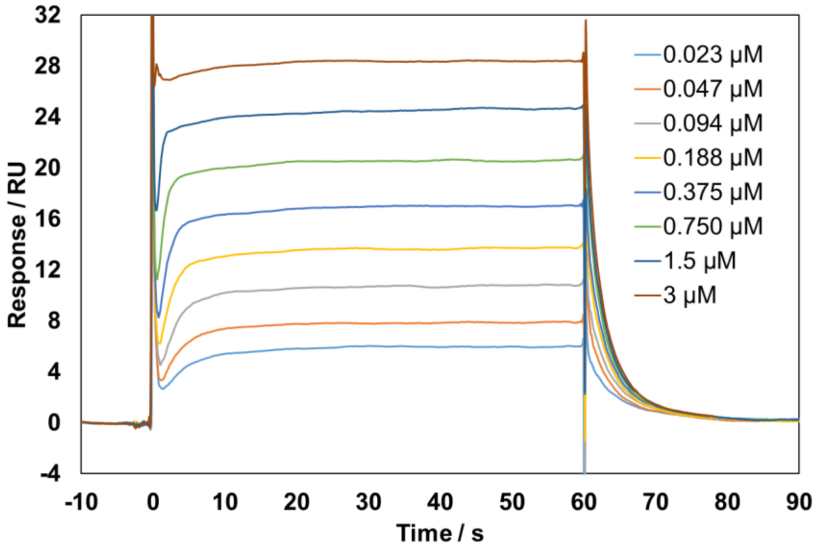

Data fitting using the Biacore T200 evaluation software:

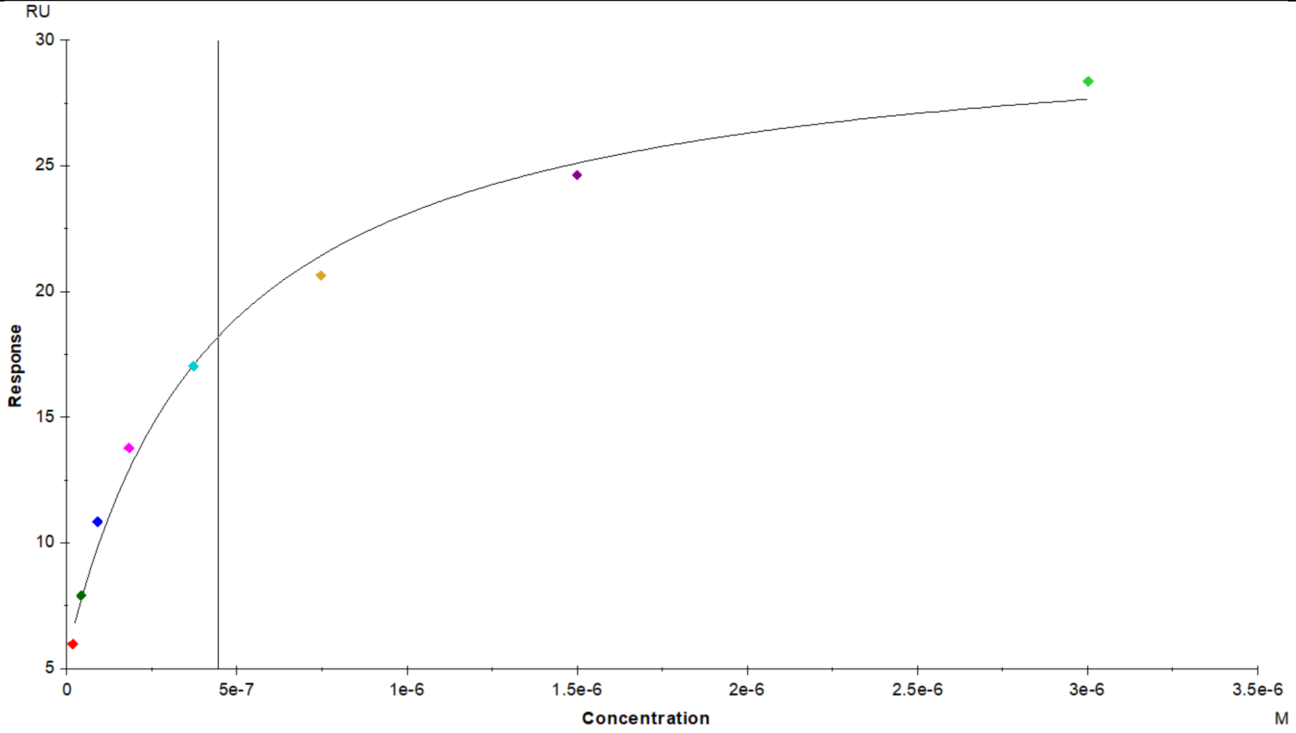

K_D (µM)	SE (K_D)	R_{max} (RU)	SE(R_{max})	offset (RU)	SE(offset)	Chi² (RU²)
0.4468	0.0850	25.4	1.1	5.5	0.76	0.692

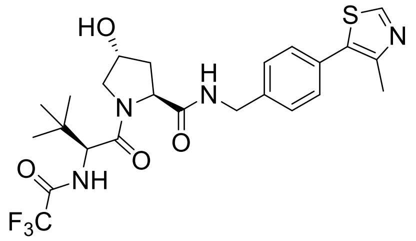

Spy molecule 22 (Compound S12b)

Blank and reference surface subtracted responses according to the concentration of spy molecule.

Theoretical R_{MAX} = 36.6

[C] (μM)	log_{10}[C]	Response (RU)
0.0039	-8.41	6.1
0.0078	-8.11	7.1
0.0156	-7.81	9.9
0.0313	-7.51	13.5
0.0625	-7.20	18.9
0.1250	-6.90	25.0
0.2500	-6.60	30.0
0.5000	-6.30	35.4

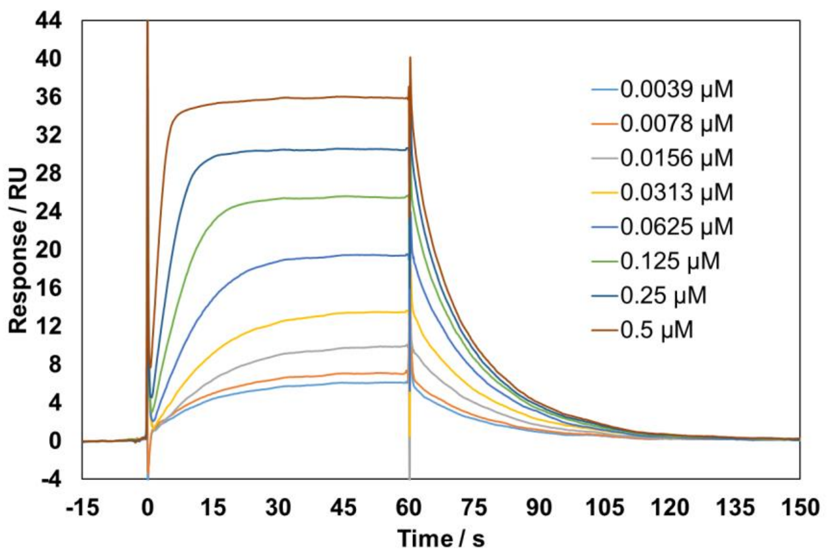

Data fitting using the Biacore T200 evaluation software:

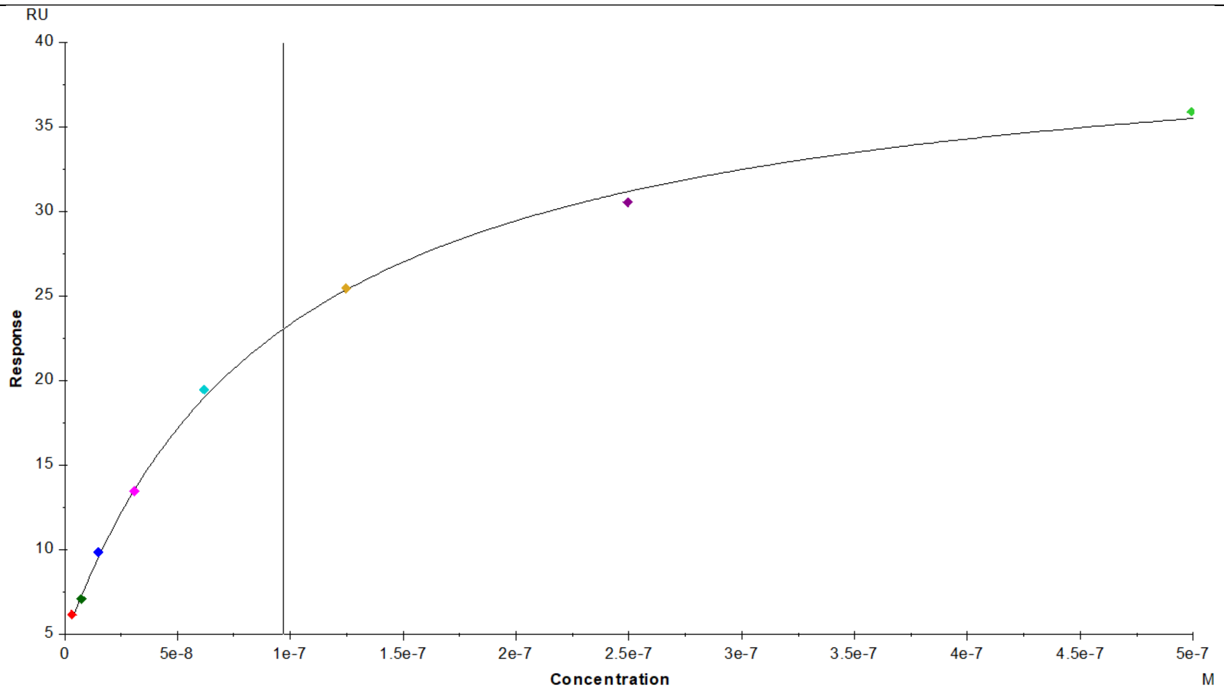

K_D (μM)	SE (K_D)	R_{max} (RU)	SE(R_{max})	offset (RU)	SE(offset)	Chi^2 (RU^2)
0.09693	0.00680	36.9	0.65	4.6	0.35	0.178

8. [19]F CPMG signal intensity versus CPMG filter

8.1 Spy molecules at 100 µM in absence or in presence of VBC 1 µM

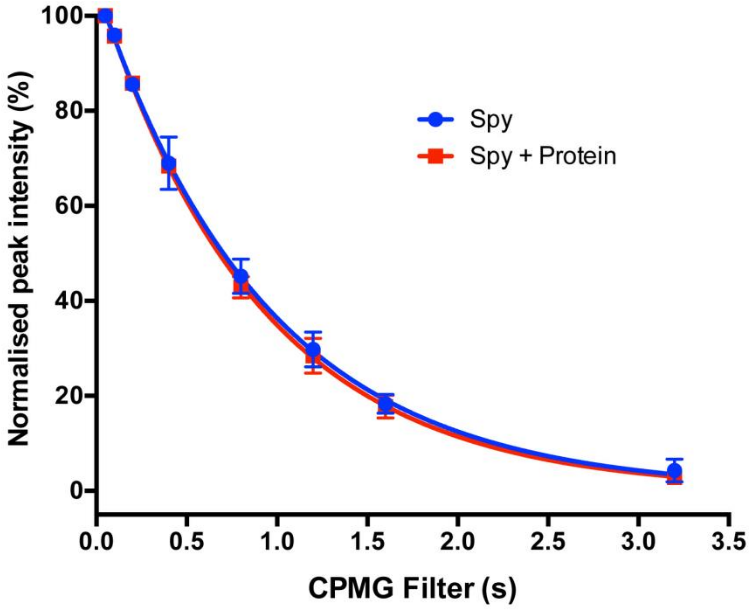

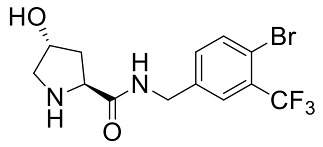

1

Spy molecule 100 µM	
R_2 (s^{-1})	1.069 ± 0.028
R-square	0.995
**Spy molecule 100 µM + VBC 1 µM**	
R_2 (s^{-1})	1.115 ± 0.020
R-square	0.998
**Contrast**	
R_2 contrast – C_2 (%)	4.1 ± 3.1
d_{max} (s)	0.916

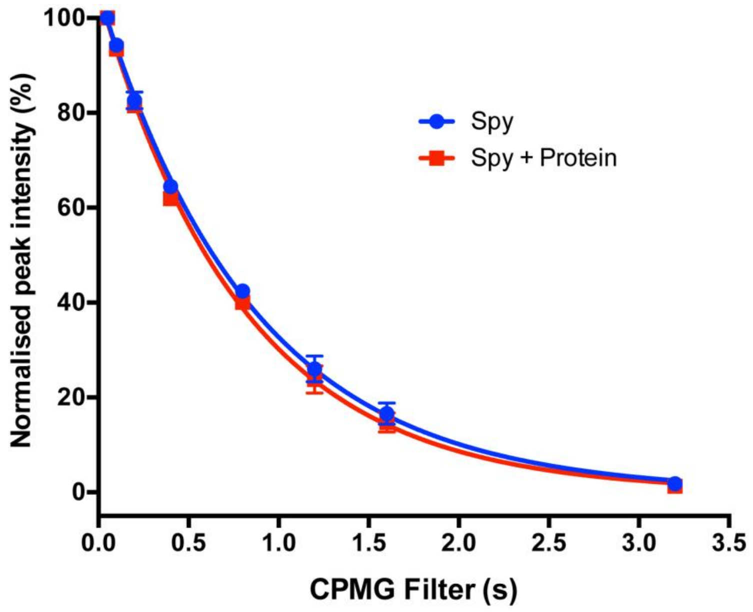

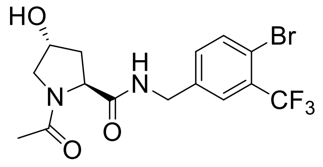

2

Spy molecule 100 µM	
R_2 (s^{-1})	1.170 ± 0.019
R-square	0.998
**Spy molecule 100 µM + VBC 1 µM**	
R_2 (s^{-1})	1.251 ± 0.021
R-square	0.998
**Contrast**	
R_2 contrast – C_2 (%)	6.5 ± 2.2
d_{max} (s)	0.826

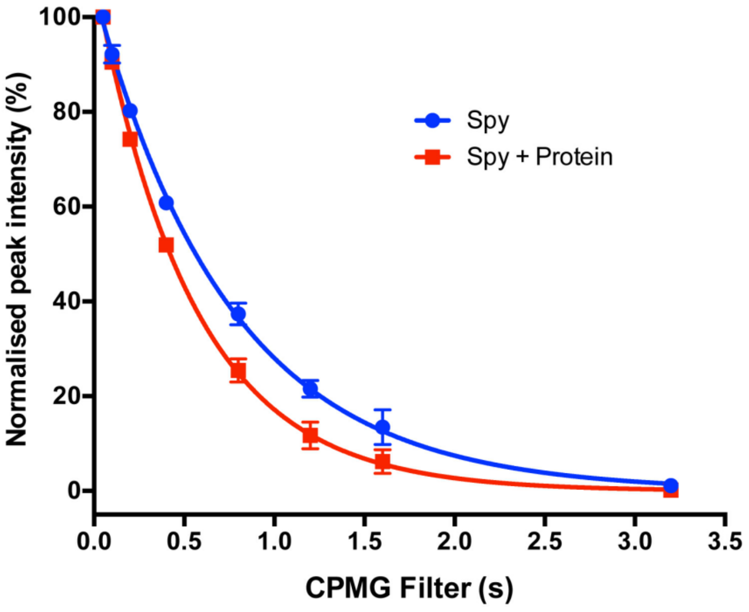

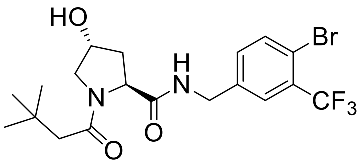

3

Spy molecule 100 µM	
R_2 (s^{-1})	1.326 ± 0.025
R-square	0.998
**Spy molecule 100 µM + VBC 1 µM**	
R_2 (s^{-1})	1.847 ± 0.033
R-square	0.998
**Contrast**	
R_2 contrast – C_2 (%)	28.2 ± 2.3
d_{max} (s)	0.636

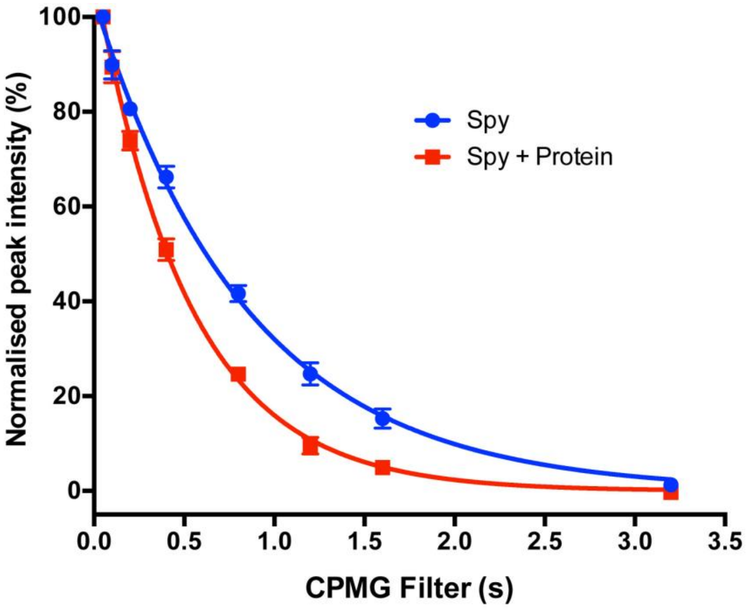

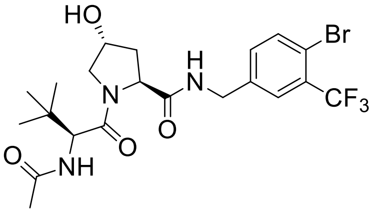

4

Spy molecule 100 µM	
R_2 (s^{-1})	1.175 ± 0.027
R-square	0.996
**Spy molecule 100 µM + VBC 1 µM**	
R_2 (s^{-1})	1.924 ± 0.037
R-square	0.998
**Contrast**	
R_2 contrast – C_2 (%)	38.9 ± 2.5
d_{max} (s)	0.658

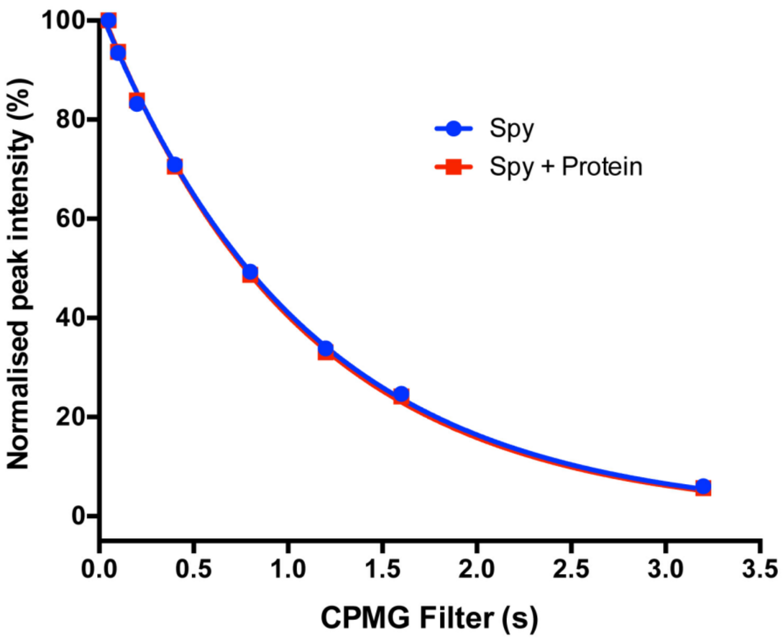

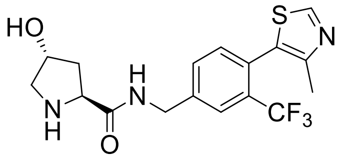

5

**Spy molecule 100 µM**	
R_2 (s^{-1})	0.915 ± 0.012
R-square	0.999
**Spy molecule 100 µM + VBC 1 µM**	
R_2 (s^{-1})	0.936 ± 0.011
R-square	0.999
**Contrast**	
R_2 contrast – C_2 (%)	2.2 ± 1.8
d_{max} (s)	1.080

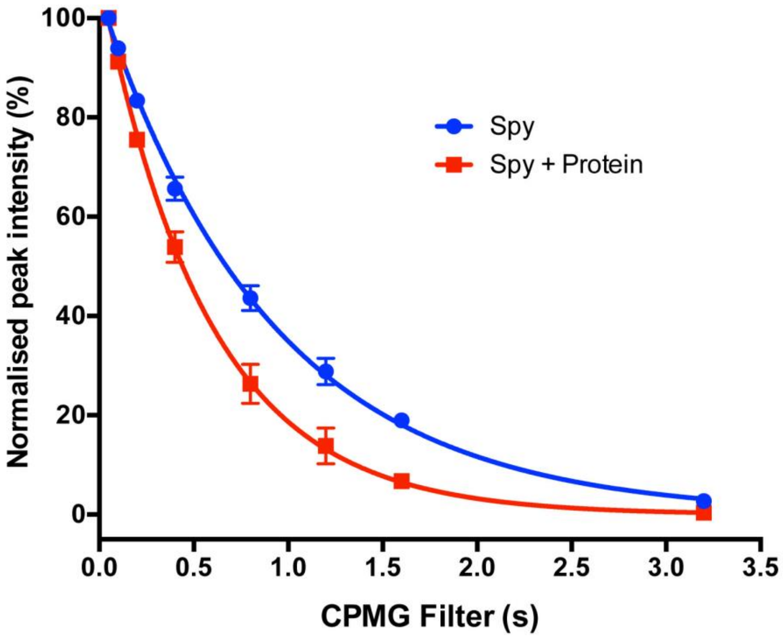

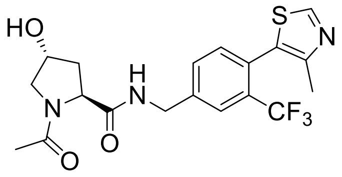

6

**Spy molecule 100 µM**	
R_2 (s^{-1})	1.096 ± 0.020
R-square	0.998
**Spy molecule 100 µM + VBC 1 µM**	
R_2 (s^{-1})	1.760 ± 0.039
R-square	0.997
**Contrast**	
R_2 contrast – C_2 (%)	37.7 ± 2.6
d_{max} (s)	0.713

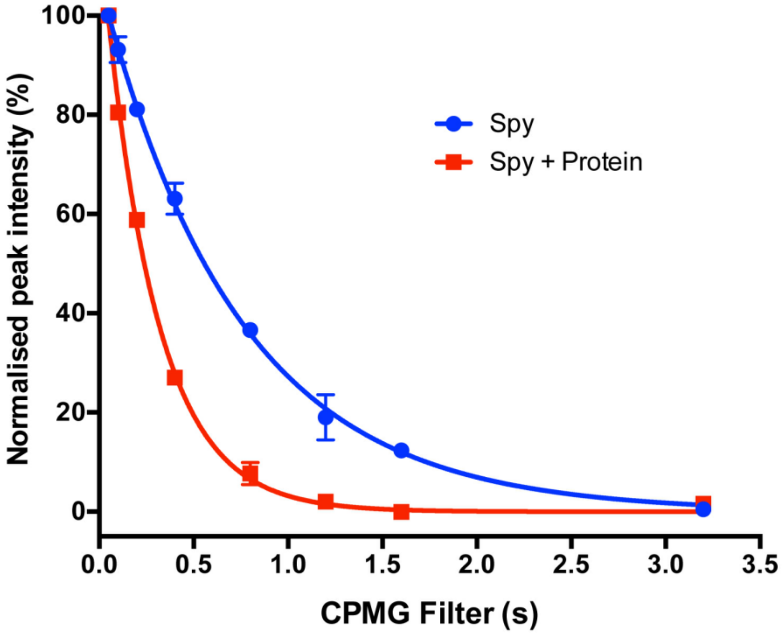

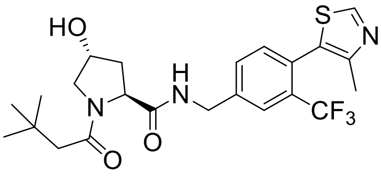

7

Spy molecule 100 µM	
R_2 (s^{-1})	1.368 ± 0.033
R-square	0.998
**Spy molecule 100 µM + VBC 1 µM**	
R_2 (s^{-1})	3.614 ± 0.088
R-square	0.999
**Contrast**	
R_2 contrast – C_2 (%)	62.1 ± 3.0
d_{max} (s)	0.433

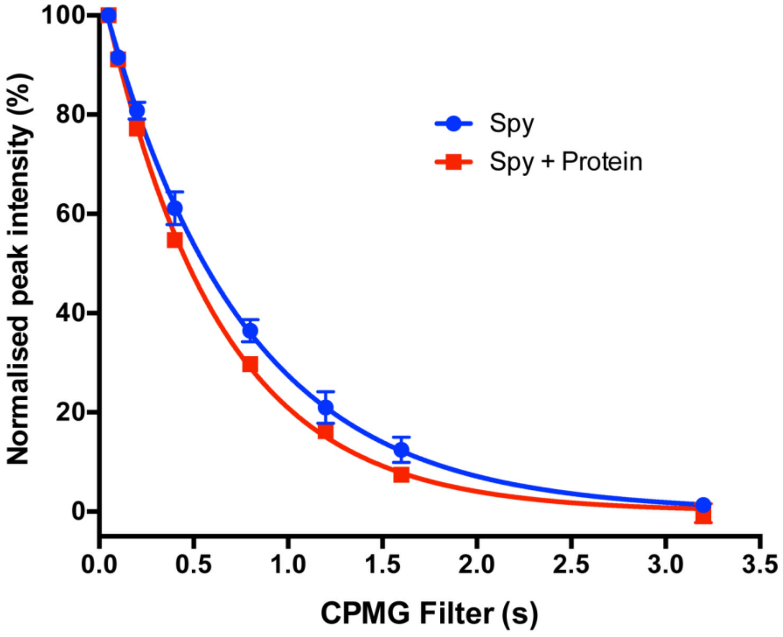

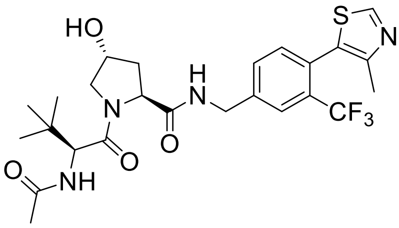

8

Spy molecule 100 µM	
R_2 (s^{-1})	1.349 ± 0.027
R-square	0.997
**Spy molecule 100 µM + VBC 1 µM**	
R_2 (s^{-1})	1.639 ± 0.022
R-square	0.999
**Contrast**	
R_2 contrast – C_2 (%)	17.7 ± 2.2
d_{max} (s)	0.671

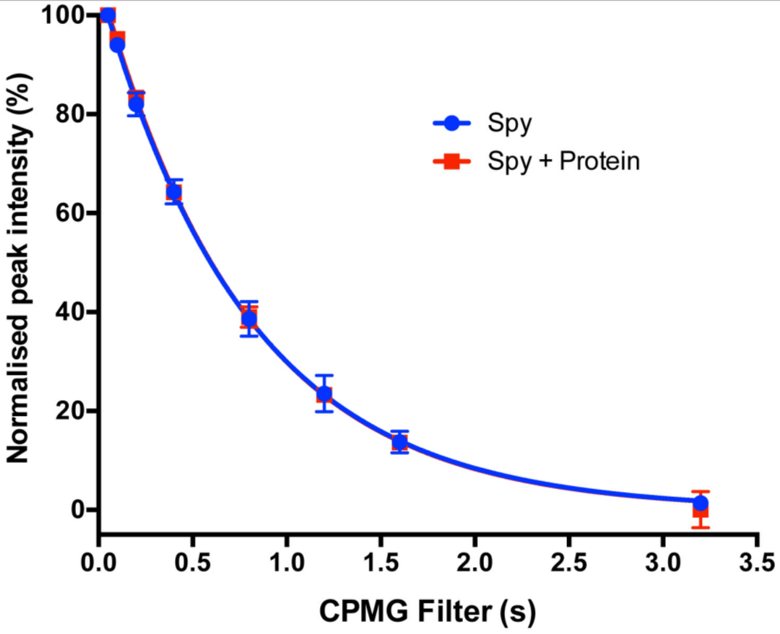

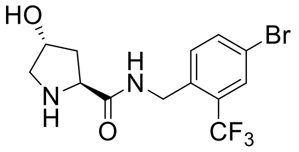

9

Spy molecule 100 µM	
R_2 (s^{-1})	1.268 ± 0.026
R-square	0.997
**Spy molecule 100 µM + VBC 1 µM**	
R_2 (s^{-1})	1.279 ± 0.022
R-square	0.998
**Contrast**	
R_2 contrast – C_2 (%)	0.9 ± 2.7
d_{max} (s)	0.785

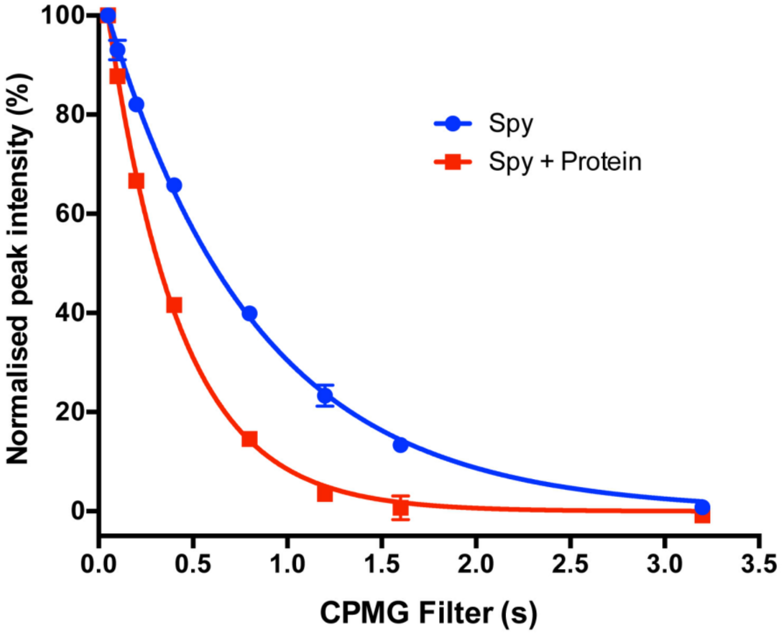

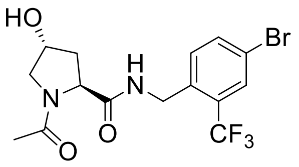

10

Spy molecule 100 µM	
R_2 (s^{-1})	1.249 ± 0.018
R-square	0.999
**Spy molecule 100 µM + VBC 1 µM**	
R_2 (s^{-1})	2.596 ± 0.046
R-square	0.999
**Contrast**	
R_2 contrast – C_2 (%)	51.9 ± 2.1
d_{max} (s)	0.543

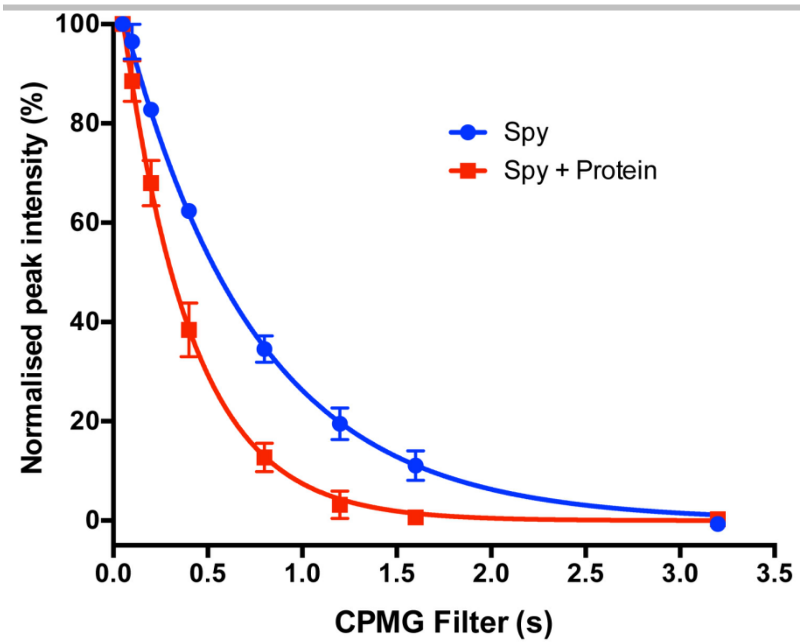

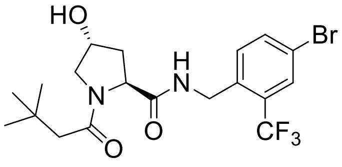

11

Spy molecule 100 µM	
R_2 (s^{-1})	1.427 ± 0.033
R-square	0.997
**Spy molecule 100 µM + VBC 1 µM**	
R_2 (s^{-1})	2.740 ± 0.096
R-square	0.995
**Contrast**	
R_2 contrast – C_2 (%)	47.9 ± 4.1
d_{max} (s)	0.497

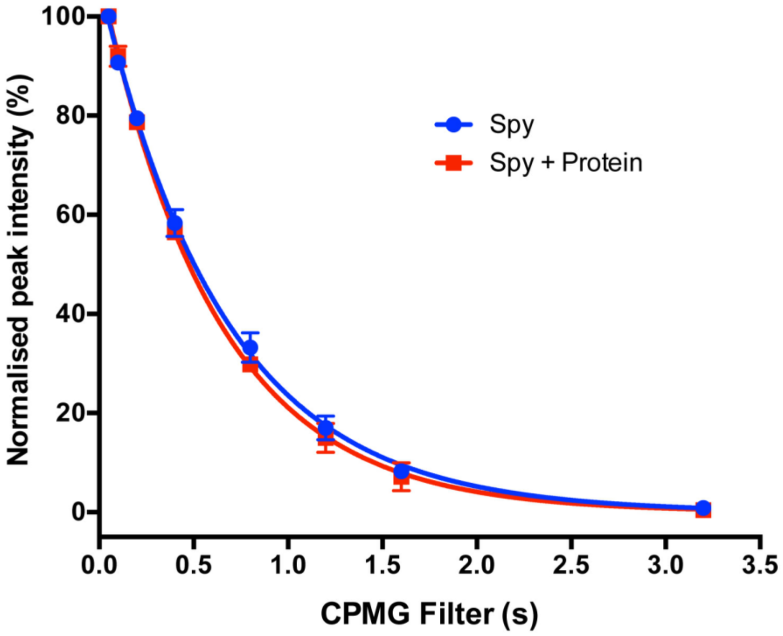

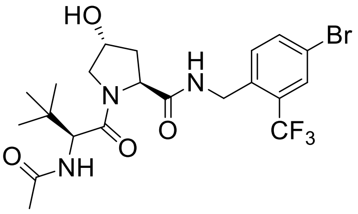

12

Spy molecule 100 µM	
R_2 (s^{-1})	1.512 ± 0.029
R-square	0.998
**Spy molecule 100 µM + VBC 1 µM**	
R_2 (s^{-1})	1.637 ± 0.028
R-square	0.998
**Contrast**	
R_2 contrast – C_2 (%)	7.6 ± 2.4
d_{max} (s)	0.635

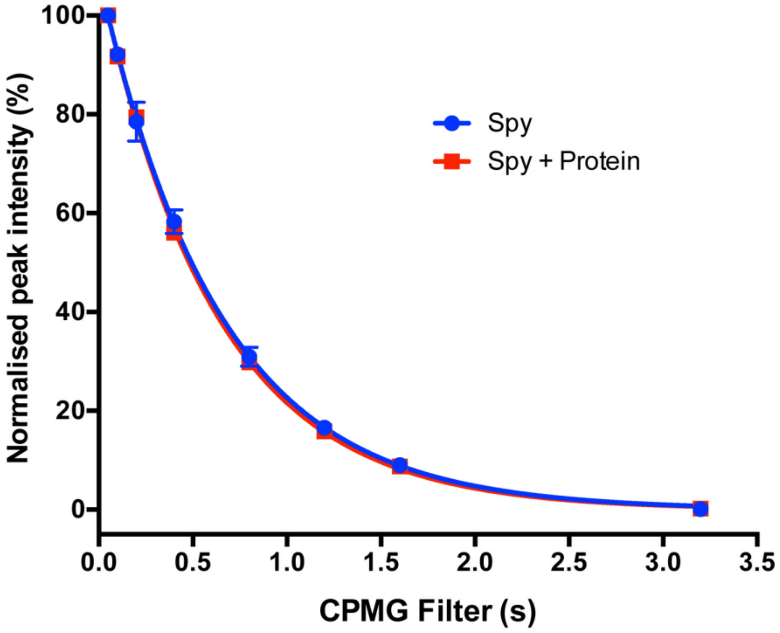

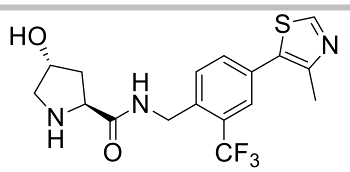

13

Spy molecule 100 µM	
R_2 (s^{-1})	1.558 ± 0.028
R-square	0.998
**Spy molecule 100 µM + VBC 1 µM**	
R_2 (s^{-1})	1.609 ± 0.017
R-square	0.999
**Contrast**	
R_2 contrast – C_2 (%)	3.2 ± 2.0
d_{max} (s)	0.632

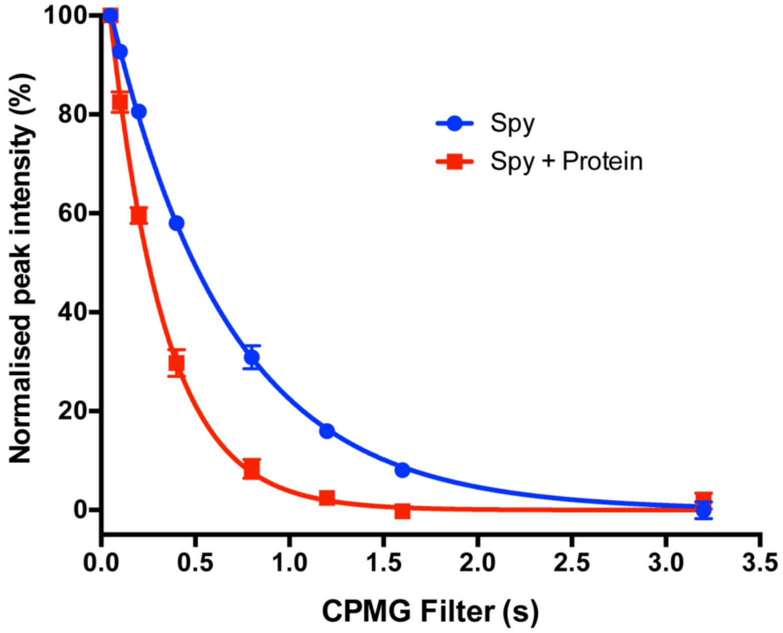

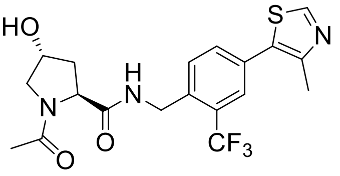

14

Spy molecule 100 µM	
R_2 (s^{-1})	1.579 ± 0.022
R-square	0.999
**Spy molecule 100 µM + VBC 1 µM**	
R_2 (s^{-1})	3.425 ± 0.073
R-square	0.998
**Contrast**	
R_2 contrast – C_2 (%)	53.9 ± 2.5
d_{max} (s)	0.419

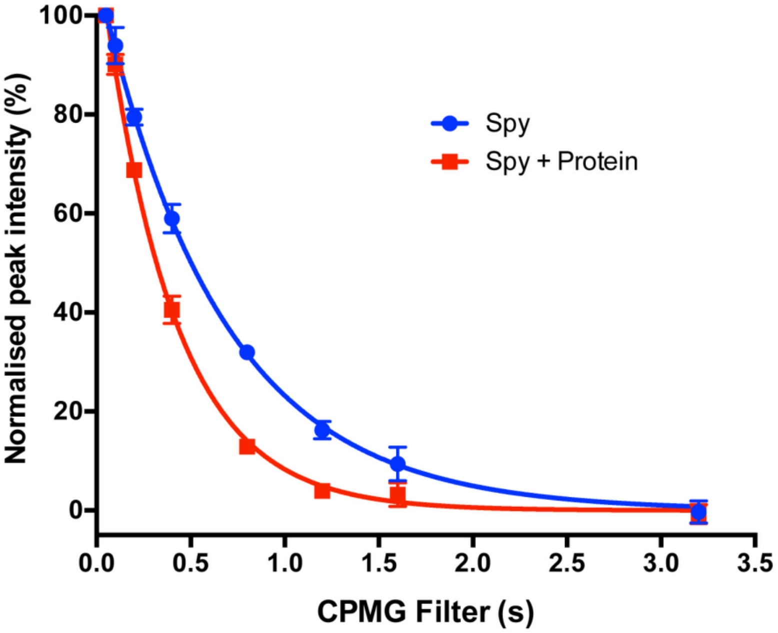

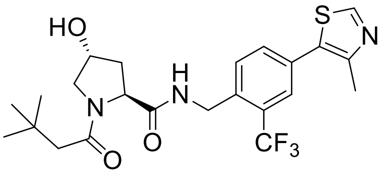

15

Spy molecule 100 µM	
R_2 (s^{-1})	1.545 ± 0.035
R-square	0.997
**Spy molecule 100 µM + VBC 1 µM**	
R_2 (s^{-1})	2.634 ± 0.058
R-square	0.998
**Contrast**	
R_2 contrast – C_2 (%)	41.3 ± 2.7
d_{max} (s)	0.490

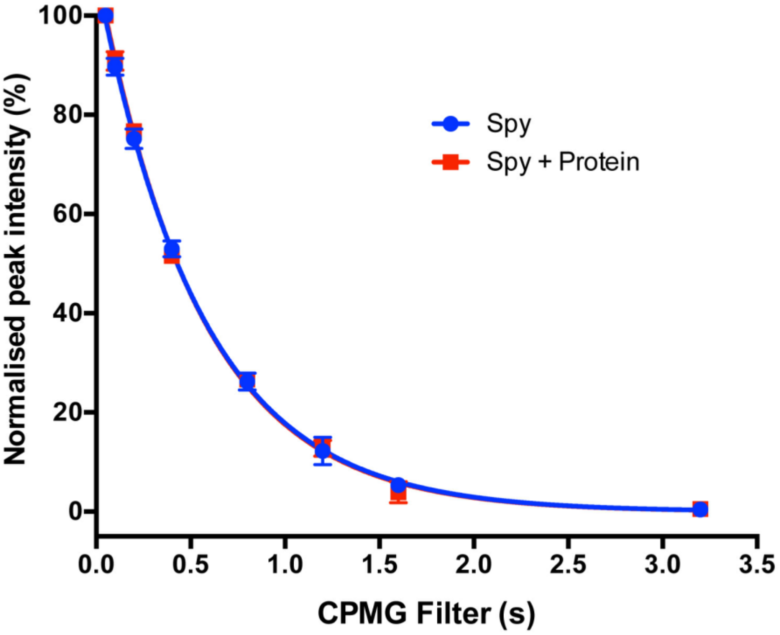

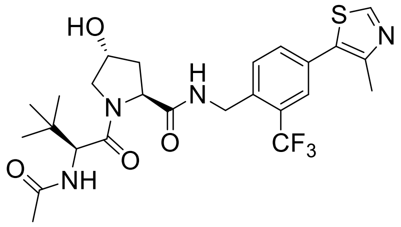

16

Spy molecule 100 µM	
R_2 (s^{-1})	1.805 ± 0.032
R-square	0.998
**Spy molecule 100 µM + VBC 1 µM**	
R_2 (s^{-1})	1.828 ± 0.031
R-square	0.999
**Contrast**	
R_2 contrast – C_2 (%)	1.3 ± 2.4
d_{max} (s)	0.551

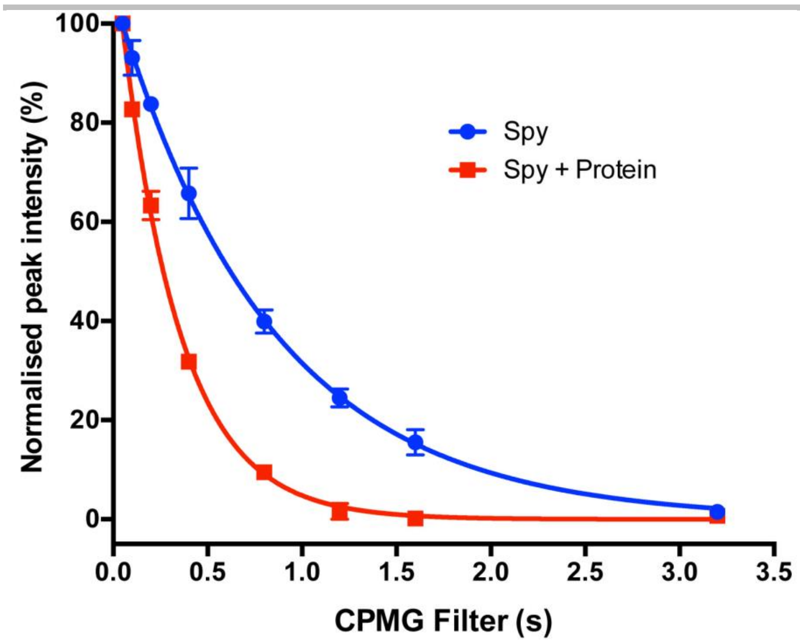

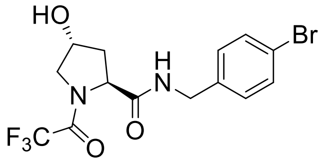

17

Spy molecule 100 µM	
R_2 (s^{-1})	1.213 ± 0.028
R-square	0.996
**Spy molecule 100 µM + VBC 1 µM**	
R_2 (s^{-1})	3.189 ± 0.066
R-square	0.998
**Contrast**	
R_2 contrast – C_2 (%)	62.0 ± 2.6
d_{max} (s)	0.489

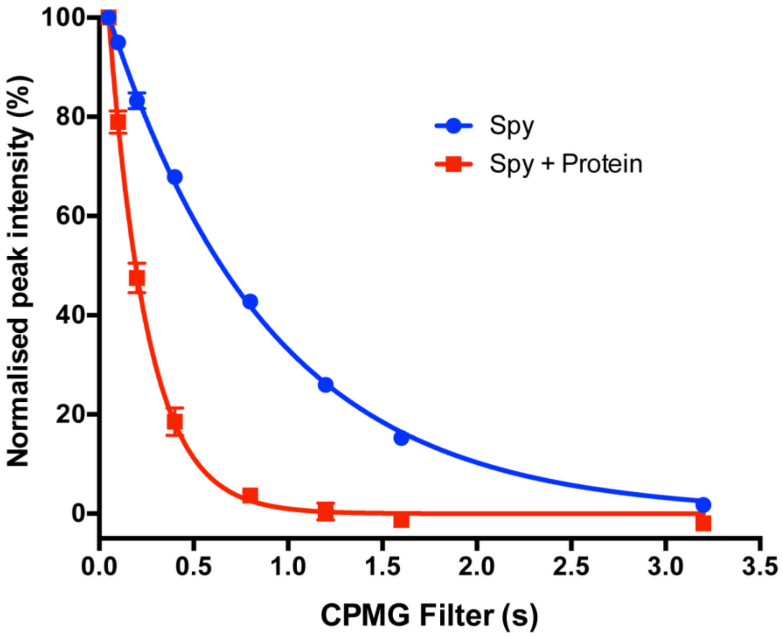

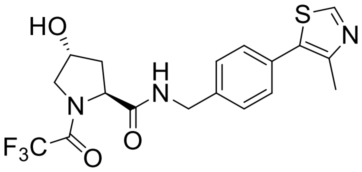

18

Spy molecule 100 µM	
R_2 (s^{-1})	1.167 ± 0.014
R-square	0.999
**Spy molecule 100 µM + VBC 1 µM**	
R_2 (s^{-1})	4.858 ± 0.123
R-square	0.998
**Contrast**	
R_2 contrast – C_2 (%)	76.0 ± 3.2
d_{max} (s)	0.386

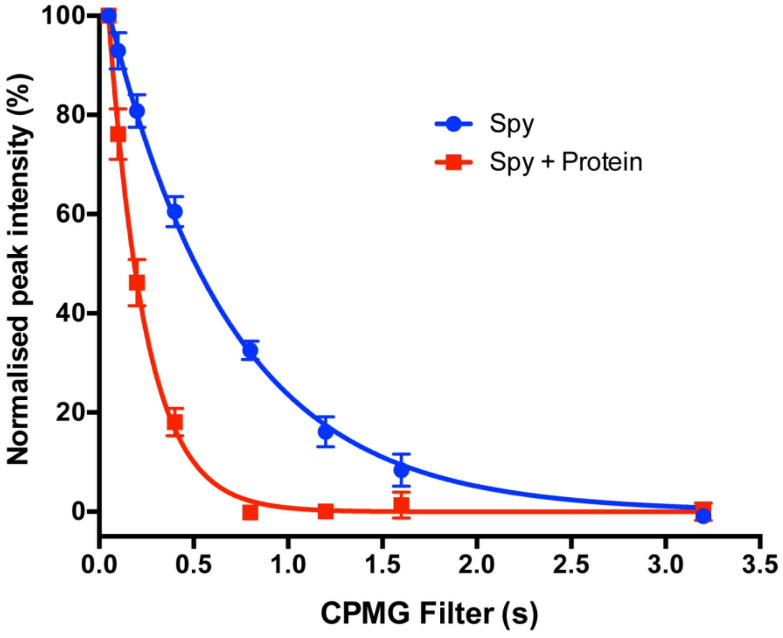

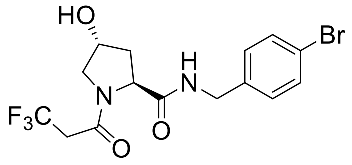

19

Spy molecule 100 µM	
R_2 (s^{-1})	1.526 ± 0.041
R-square	0.996
**Spy molecule 100 µM + VBC 1 µM**	
R_2 (s^{-1})	5.096 ± 0.186
R-square	0.995
**Contrast**	
R_2 contrast – C_2 (%)	70.1 ± 4.5
d_{max} (s)	0.338

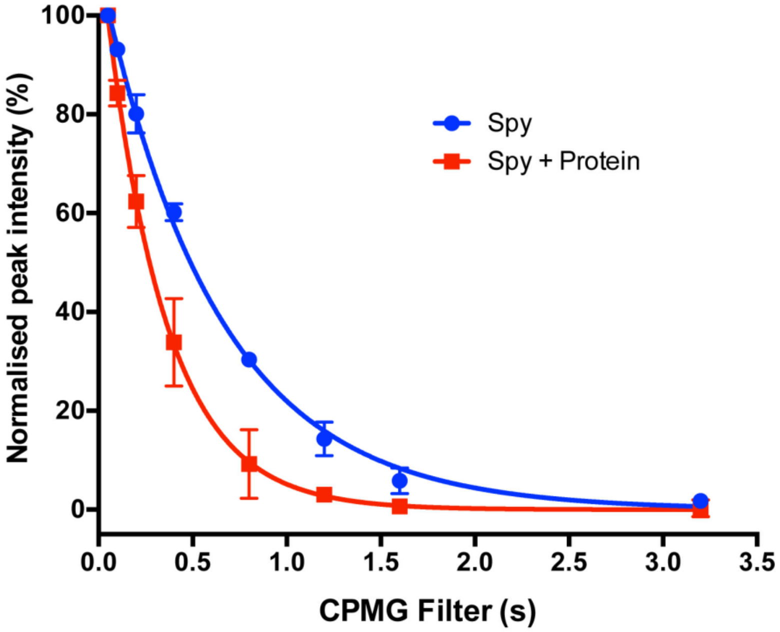

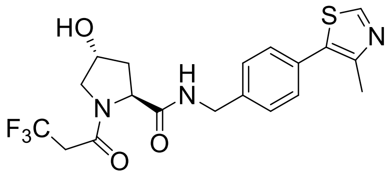

20

Spy molecule 100 µM	
R_2 (s^{-1})	1.609 ± 0.042
R-square	0.996
**Spy molecule 100 µM + VBC 1 µM**	
R_2 (s^{-1})	3.116 ± 0.153
R-square	0.990
**Contrast**	
R_2 contrast – C_2 (%)	48.4 ± 5.6
d_{max} (s)	0.439

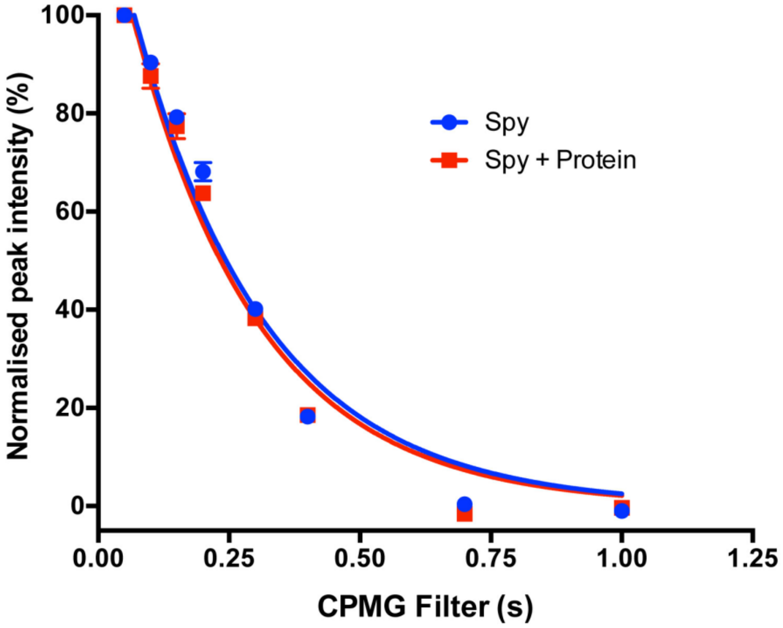

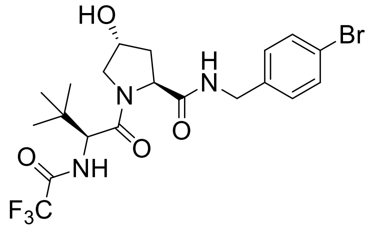

21

Spy molecule 100 µM	
R_2 (s^{-1})	3.959 ± 0.262
R-square	0.970
**Spy molecule 100 µM + VBC 1 µM**	
R_2 (s^{-1})	4.109 ± 0.247
R-square	0.976
**Contrast**	
R_2 contrast – C_2 (%)	3.7 ± 8.8
d_{max} (s)	0.248

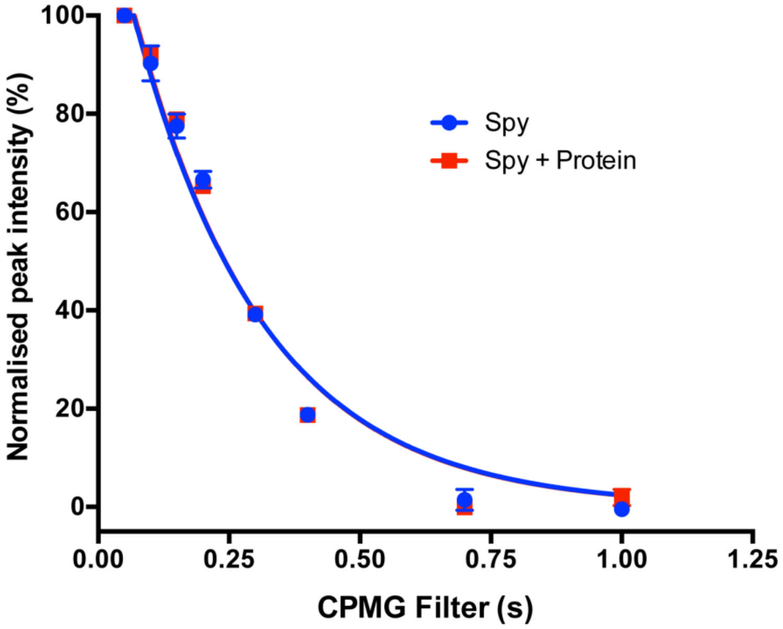

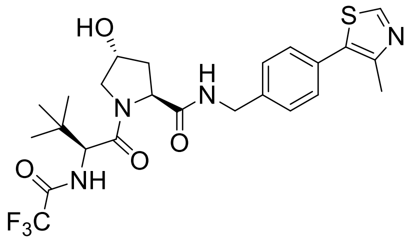

22

Spy molecule 100 µM	
R_2 (s^{-1})	3.987 ± 0.241
R-square	0.975
**Spy molecule 100 µM + VBC 1 µM**	
R_2 (s^{-1})	4.018 ± 0.245
R-square	0.974
**Contrast**	
R_2 contrast – C_2 (%)	0.8 ± 8.6
d_{max} (s)	0.250

8.2 Spy molecule **19** and VBC at different concentrations

Spy molecule 19 at 50 µM

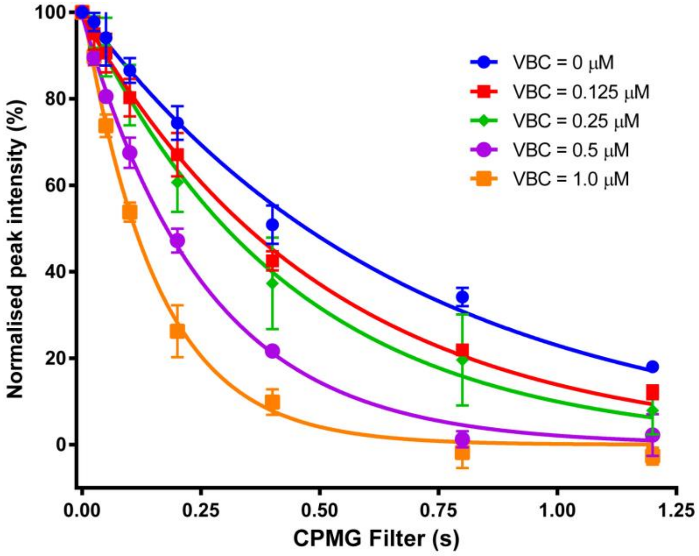

Parameter	[VBC] µM				
	0.000	0.125	0.250	0.500	1.000
R_2 (s^{-1})	1.475 ± 0.059	1.969 ± 0.071	2.316 ± 0.168	3.857 ± 0.121	6.424 ± 0.262
R-square	0.986	0.991	0.967	0.995	0.993
R_2 contrast – C_2 (%)	-	22.5 ± 4.2	34.1 ± 7.9	60.4 ± 3.8	76.2 ± 5.2
d_{max} (s)	-	0.575	0.528	0.398	0.293

Spy molecule 19 at 25 µM

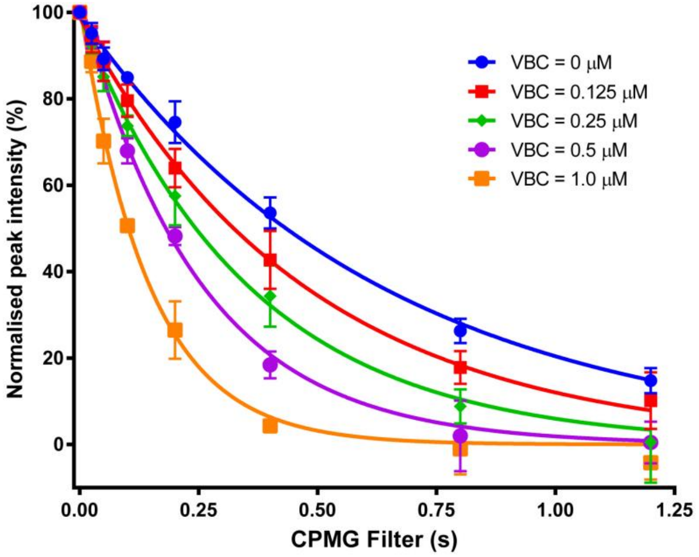

Parameter	[VBC] µM				
	0.000	0.125	0.250	0.500	1.000
R_2 (s^{-1})	1.582 ± 0.049	2.107 ± 0.095	2.811 ± 0.156	4.008 ± 0.200	6.934 ± 0.348
R-square	0.992	0.986	0.984	0.989	0.989
R_2 contrast – C_2 (%)	-	27.6 ± 5.1	45.7 ± 6.3	61.9 ± 5.9	78.0 ± 6.4
d_{max} (s)	-	0.555	0.475	0.389	0.280

Spy molecule 19 at 10 µM

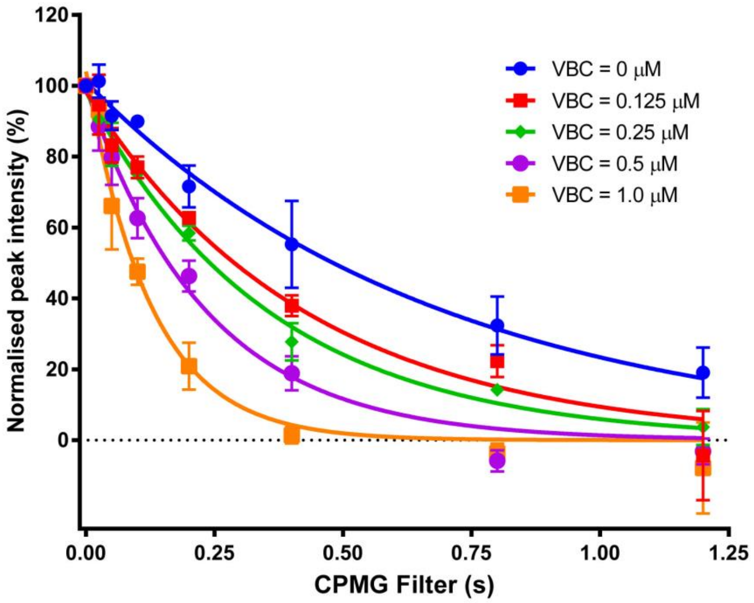

Parameter	[VBC] µM				
	0.000	0.125	0.250	0.500	1.000
**R_2 (s^{-1})**	1.464 ± 0.096	2.346 ± 0.193	2.807 ± 0.131	4.288 ± 0.304	7.974 ± 0.727
**R-square**	0.962	0.961	0.988	0.979	0.967
**R_2 contrast – C_2 (%)**	-	35.0 ± 8.9	45.6 ± 5.3	64.4 ± 8.5	80.9 ± 11.7
**d_{max} (s)**	-	0.524	0.476	0.374	0.256

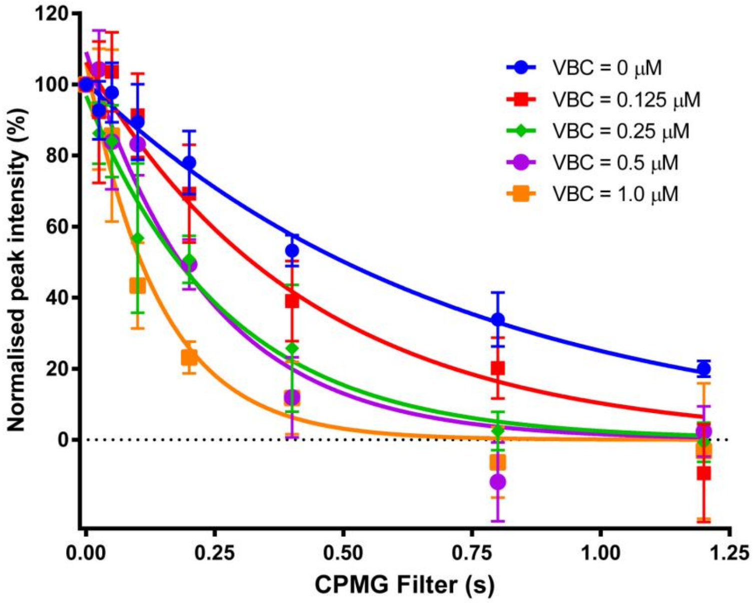

Parameter	[VBC] µM				
	0.000	0.125	0.250	0.500	1.000
**R_2 (s^{-1})**	1.392 ± 0.101	2.337 ± 0.335	3.678 ± 0.489	4.255 ± 0.566	7.063 ± 1.128
**R-square**	0.952	0.898	0.922	0.931	0.903
**R_2 contrast – C_2 (%)**	-	34.7 ± 15.3	58.5 ± 15.5	64.1 ± 15.8	78.4 ± 20.3
**d_{max} (s)**	-	0.526	0.409	0.376	0.277

8.3 Spy molecules **6** and **11** at 50 µM in absence or in presence of VBC 1 µM

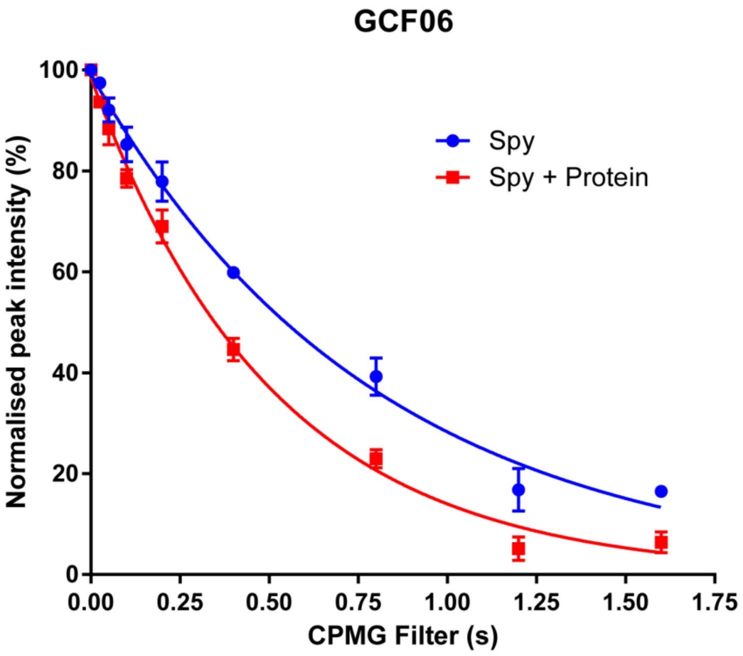

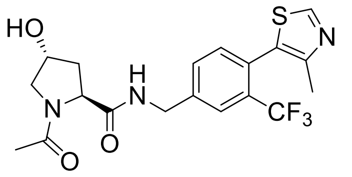

6

Spy molecule 100 µM	
R_2 (s^{-1})	1.255 ± 0.043
R-square	0.989
**Spy molecule 100 µM + VBC 1 µM**	
R_2 (s^{-1})	1.951 ± 0.062
R-square	0.994
**Contrast**	
R_2 contrast – C_2 (%)	35.7 ± 4.0
d_{max} (s)	0.634

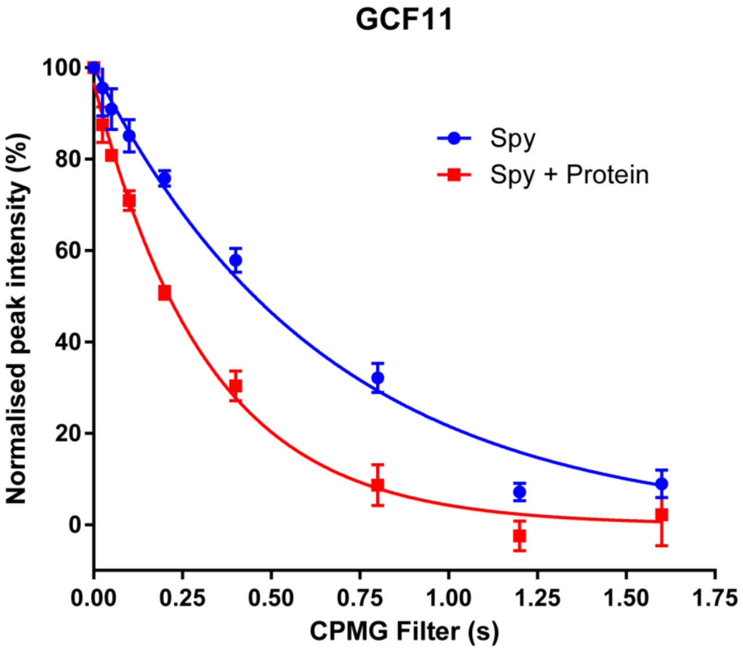

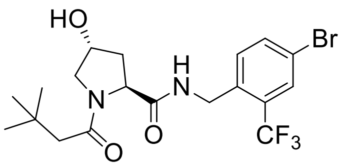

11

Spy molecule 100 µM	
R_2 (s^{-1})	1.534 ± 0.071
R-square	0.984
**Spy molecule 100 µM + VBC 1 µM**	
R_2 (s^{-1})	3.122 ± 0.145
R-square	0.990
**Contrast**	
R_2 contrast – C_2 (%)	50.9 ± 5.7
d_{max} (s)	0.447

9. NMR spectra of synthesized compounds

Compound S1b

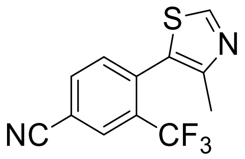

S1b

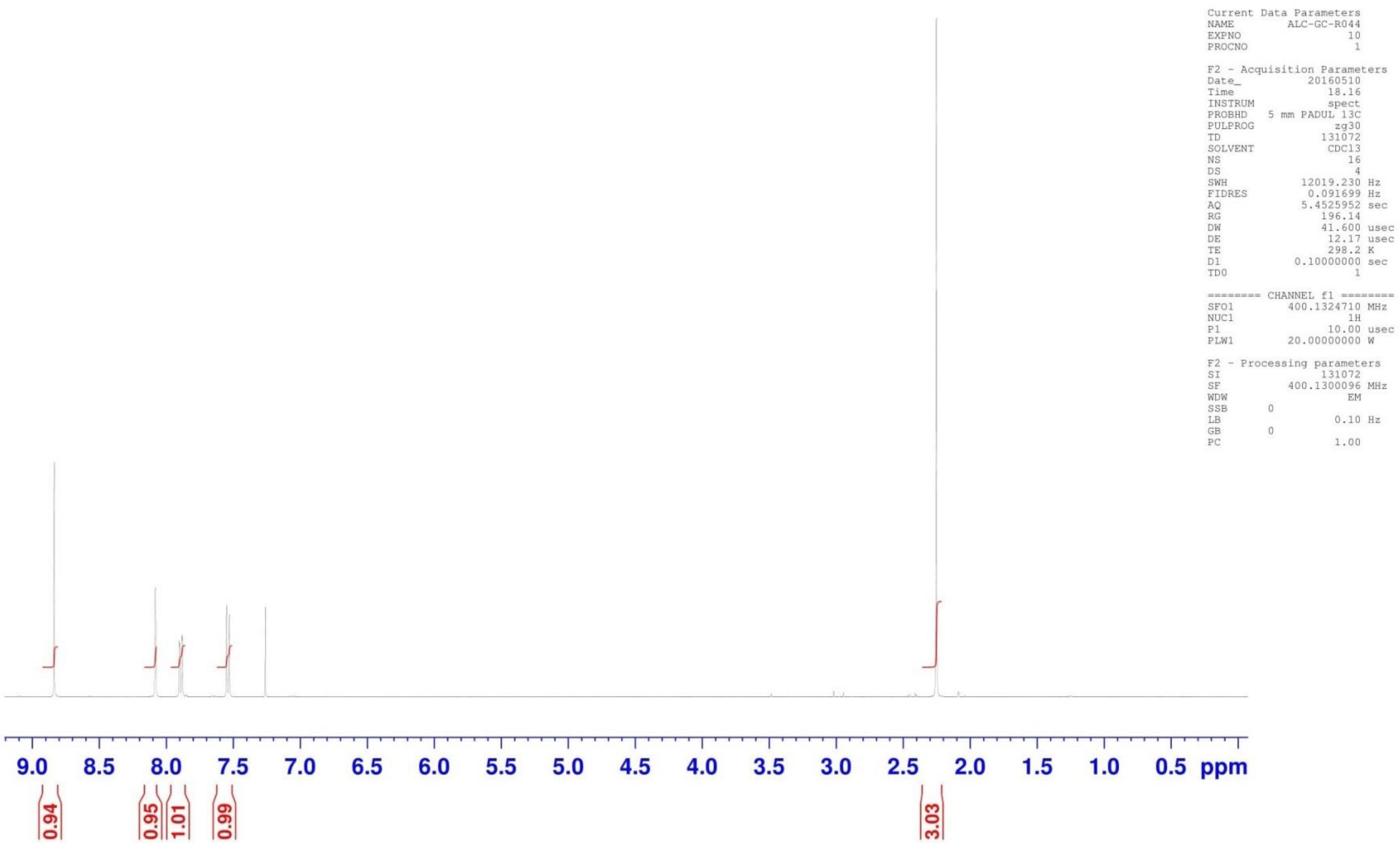

Compound S1b

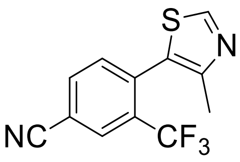

S1b

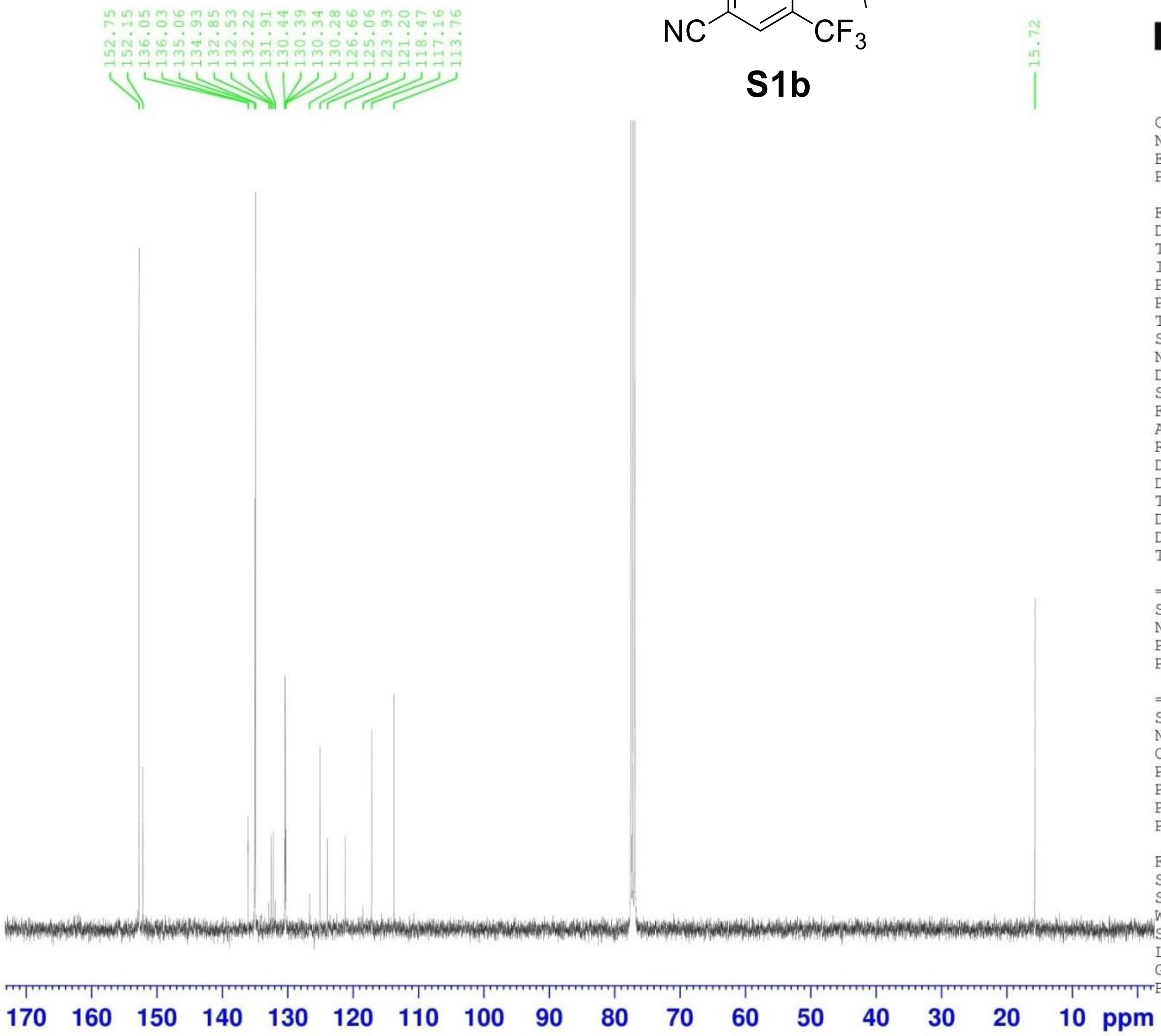


```
Current Data Parameters
NAME          ALC-GC-R044
EXPNO                  14
PROCNO                  1

F2 - Acquisition Parameters
Date_            20160511
Time                19.04
INSTRUM             spect
PROBHD    5 mm PADUL 13C
PULPROG            zgpg30
TD                 119044
SOLVENT             CDCl3
NS                    512
DS                      4
SWH             25000.000 Hz
FIDRES           0.210006 Hz
AQ              2.3808801 sec
RG                 196.14
DW                 20.000 usec
DE                   7.92 usec
TE                  298.2 K
D1             1.00000000 sec
D11            0.03000000 sec
TD0                     1

======== CHANNEL f1 ========
SFO1         100.6238351 MHz
NUC1                  13C
P1                  10.00 usec
PLW1          36.00000000 W

======== CHANNEL f2 ========
SFO2         400.1316005 MHz
NUC2                   1H
CPDPRG[2          waltz64
PCPD2               90.00 usec
PLW2          20.00000000 W
PLW12          0.24691001 W
PLW13          0.20000000 W

F2 - Processing parameters
SI                 131072
SF            100.6127525 MHz
WDW                    EM
SSB      0
LB                   1.00 Hz
GB       0
PC                   1.40
```

Compound S1b

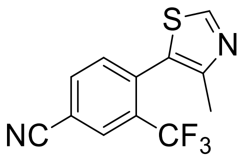

S1b

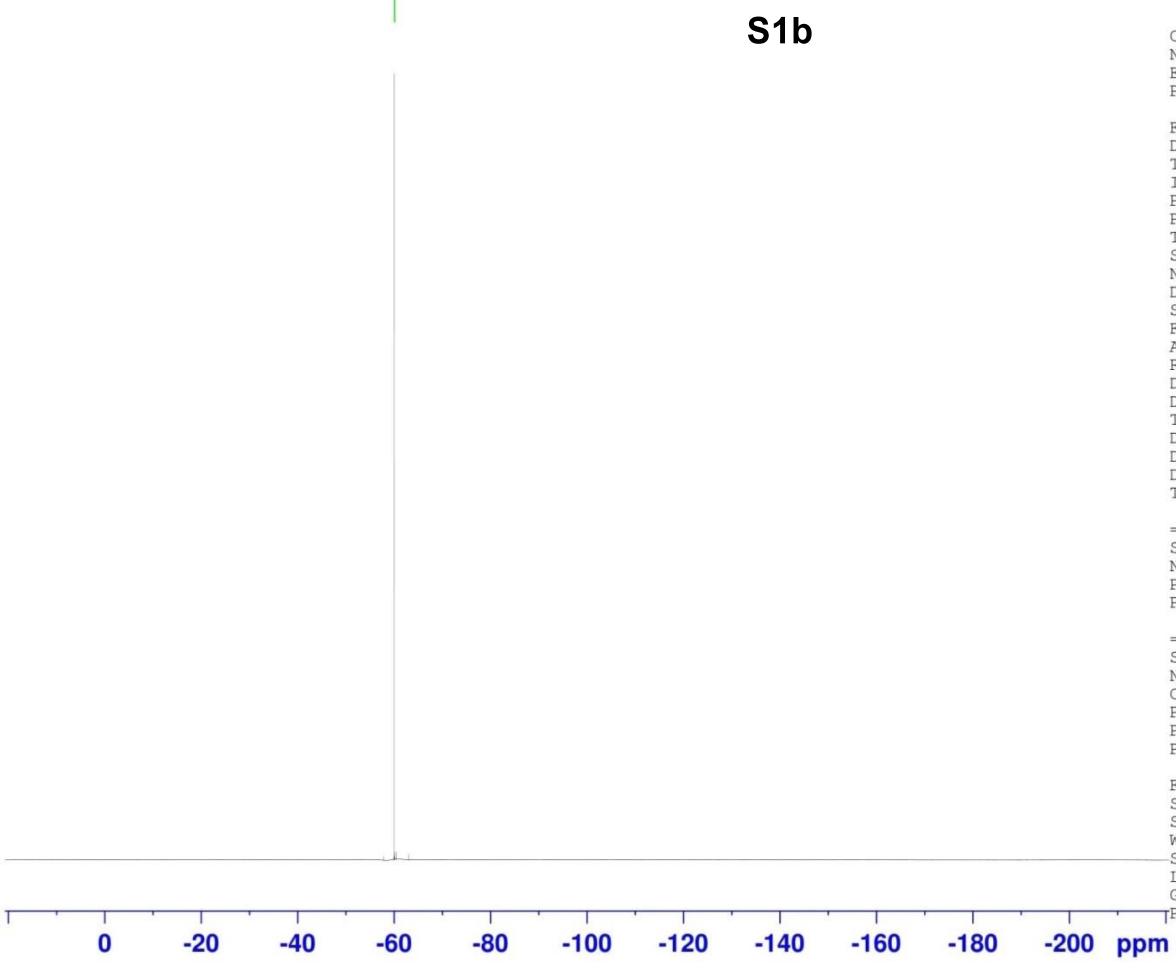


```
Current Data Parameters
NAME              GC-R044
EXPNO                   2
PROCNO                  1

F2 - Acquisition Parameters
Date_            20160517
Time                10.37
INSTRUM             spect
PROBHD    5 mm QNP 1H/13
PULPROG       zgfhigqn.2
TD                 131072
SOLVENT             CDCl3
NS                     16
DS                      4
SWH            113636.367 Hz
FIDRES           0.866977 Hz
AQ              0.5767168 sec
RG                    724
DW                  4.400 usec
DE                   6.50 usec
TE                  298.2 K
D1             1.00000000 sec
D11            0.03000000 sec
D12            0.00002000 sec
TD0                     1

======== CHANNEL f1 ========
SFO1          470.5453180 MHz
NUC1                  19F
P1                  13.50 usec
PLW1          25.00000000 W

======== CHANNEL f2 ========
SFO2          500.1320005 MHz
NUC2                   1H
CPDPRG[2          waltz16
PCPD2               80.00 usec
PLW2          25.00000000 W
PLW12          0.39063001 W

F2 - Processing parameters
SI                  65536
SF            470.5923772 MHz
WDW                    EM
SSB      0
LB                   0.30 Hz
GB       0
PC                   1.00
```

Compound S1d

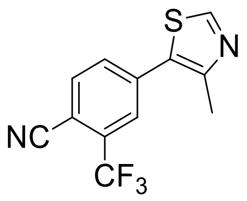

S1d

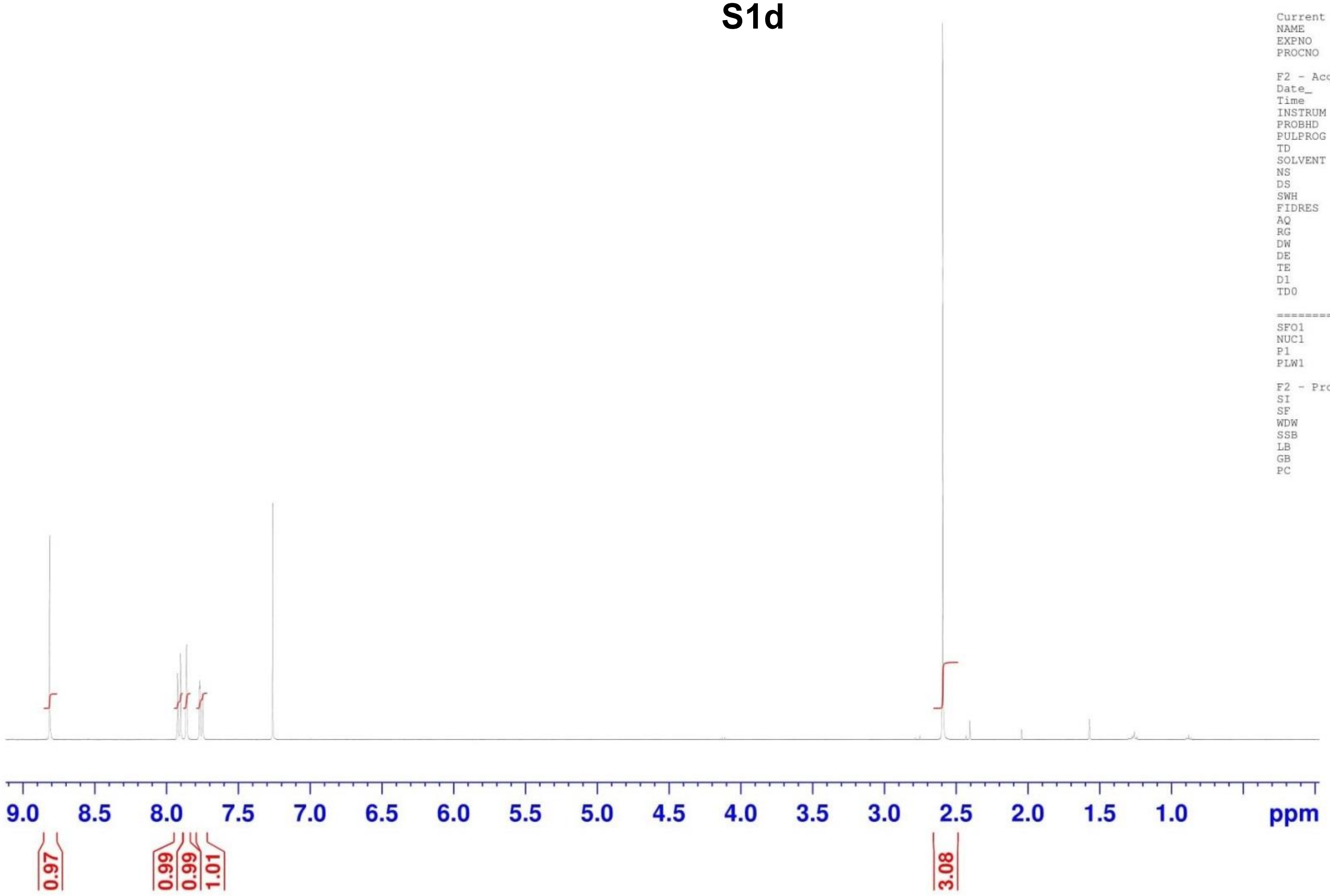


```
Current Data Parameters
NAME          ALC-GC-R045
EXPNO                  10
PROCNO                  1

F2 - Acquisition Parameters
Date_            20160511
Time                17.29
INSTRUM             spect
PROBHD   5 mm PADUL 13C
PULPROG              zg30
TD                 131072
SOLVENT             CDCl3
NS                     16
DS                      4
SWH             12019.230 Hz
FIDRES           0.091699 Hz
AQ              5.4525952 sec
RG                 196.14
DW                 41.600 usec
DE                  12.17 usec
TE                  298.2 K
D1             0.10000000 sec
TD0                     1

======== CHANNEL f1 ========
SFO1          400.1324710 MHz
NUC1                   1H
P1                  10.00 usec
PLW1          20.00000000 W

F2 - Processing parameters
SI                 131072
SF            400.1300097 MHz
WDW                    EM
SSB      0
LB                   0.10 Hz
GB       0
PC                   1.00
```

Compound S1d

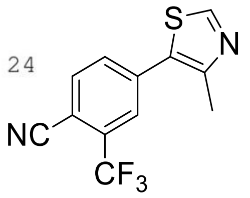

S1d

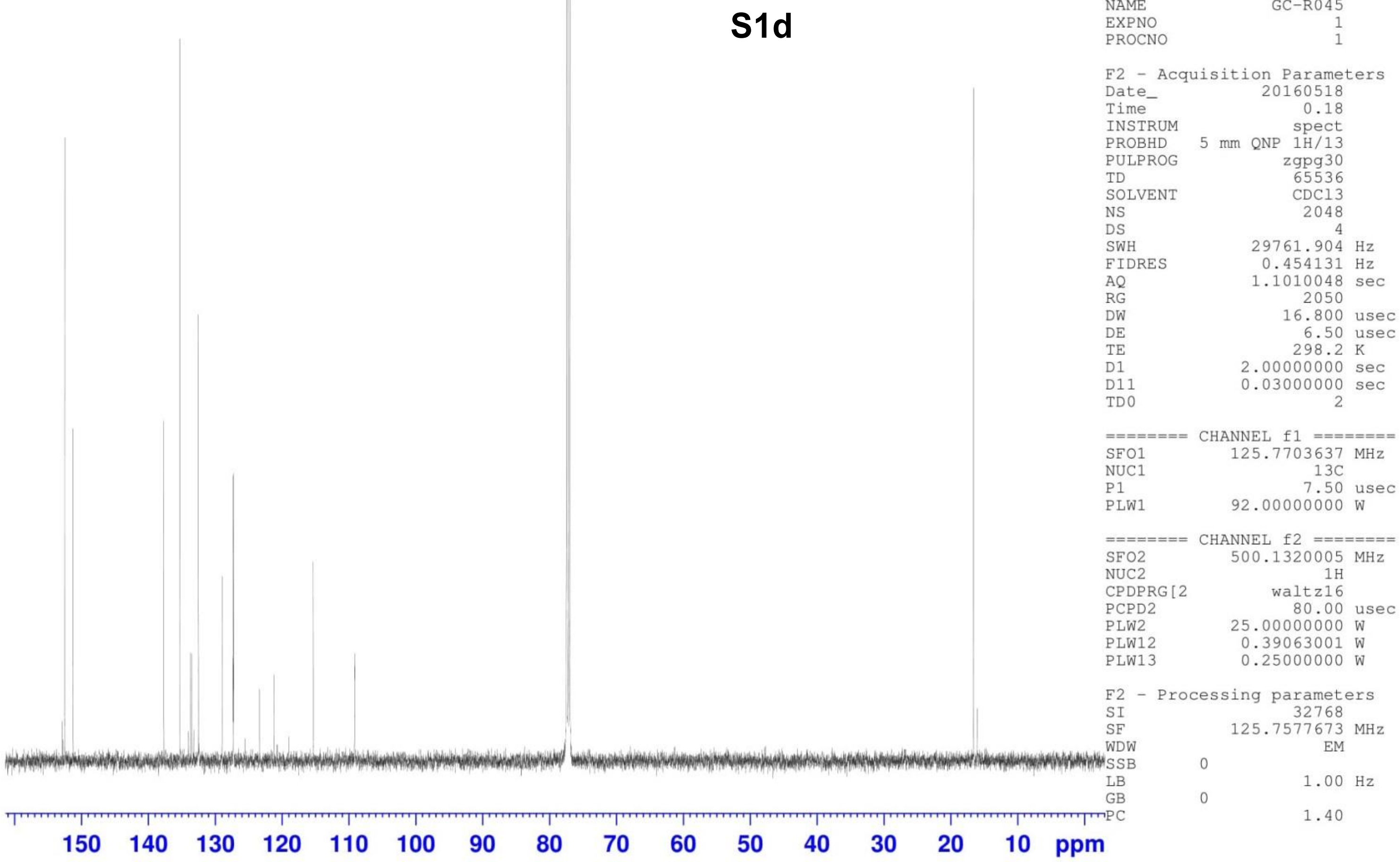


```
Current Data Parameters
NAME              GC-R045
EXPNO                   1
PROCNO                  1

F2 - Acquisition Parameters
Date_            20160518
Time                 0.18
INSTRUM             spect
PROBHD    5 mm QNP 1H/13
PULPROG           zgpg30
TD                  65536
SOLVENT             CDCl3
NS                   2048
DS                      4
SWH            29761.904 Hz
FIDRES          0.454131 Hz
AQ             1.1010048 sec
RG                   2050
DW                 16.800 usec
DE                   6.50 usec
TE                  298.2 K
D1             2.00000000 sec
D11            0.03000000 sec
TD0                     2

======== CHANNEL f1 ========
SFO1         125.7703637 MHz
NUC1                  13C
P1                   7.50 usec
PLW1          92.00000000 W

======== CHANNEL f2 ========
SFO2         500.1320005 MHz
NUC2                   1H
CPDPRG[2           waltz16
PCPD2               80.00 usec
PLW2          25.00000000 W
PLW12          0.39063001 W
PLW13          0.25000000 W

F2 - Processing parameters
SI                  32768
SF           125.7577673 MHz
WDW                    EM
SSB      0
LB                   1.00 Hz
GB       0
PC                   1.40
```

Compound S1d

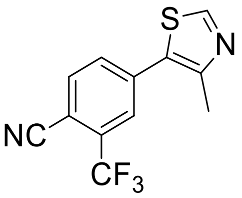

S1d

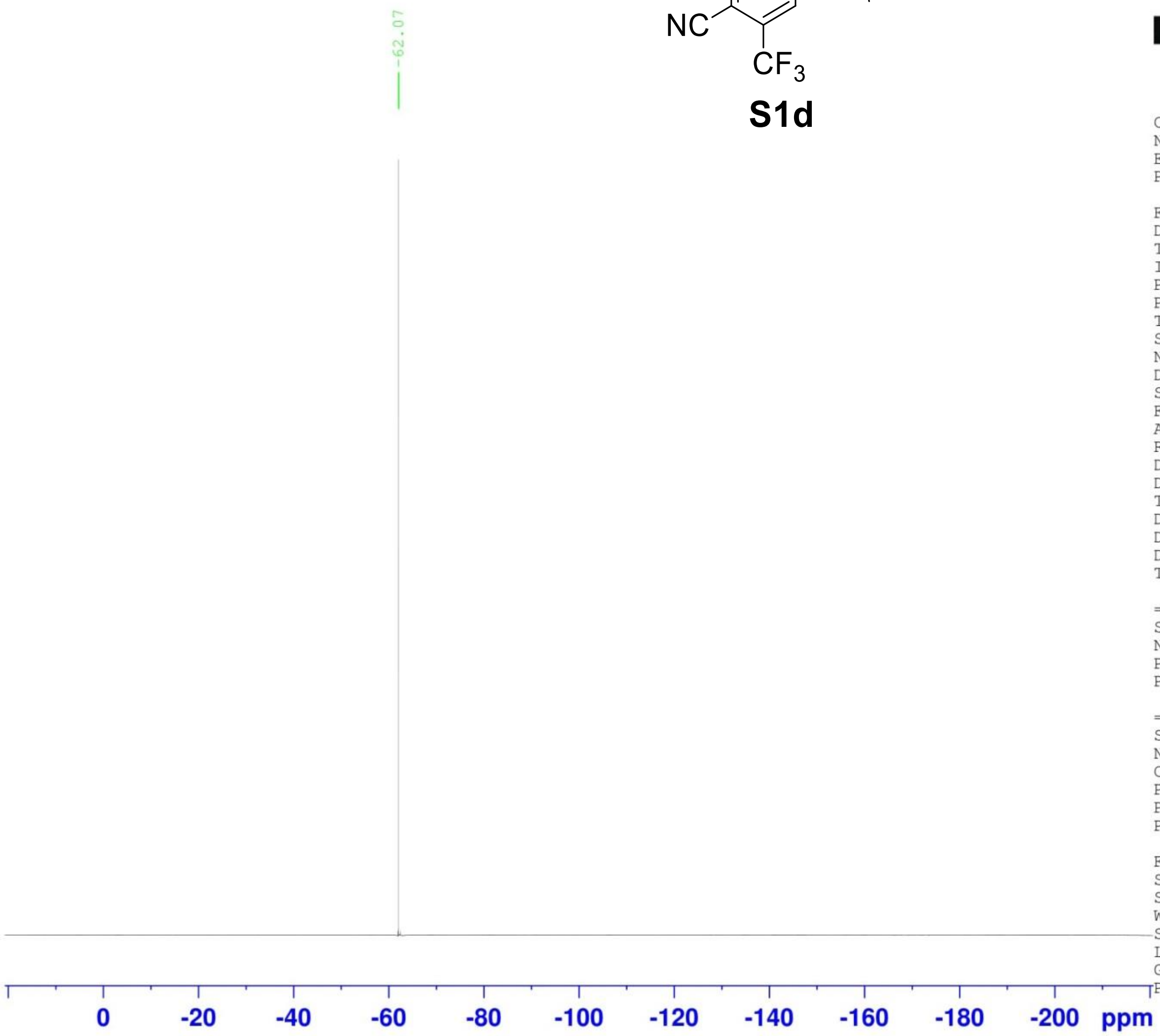


```
Current Data Parameters
NAME               GC-R045
EXPNO                    9
PROCNO                   1

F2 - Acquisition Parameters
Date_             20160517
Time                 10.50
INSTRUM              spect
PROBHD    5 mm QNP 1H/13
PULPROG        zgfhigqn.2
TD                  131072
SOLVENT              CDCl3
NS                      16
DS                       4
SWH             113636.367 Hz
FIDRES            0.866977 Hz
AQ               0.5767168 sec
RG                     724
DW                   4.400 usec
DE                    6.50 usec
TE                   298.2 K
D1              1.00000000 sec
D11             0.03000000 sec
D12             0.00002000 sec
TD0                      1

======== CHANNEL f1 ========
SFO1           470.5453180 MHz
NUC1                   19F
P1                   13.50 usec
PLW1           25.00000000 W

======== CHANNEL f2 ========
SFO2           500.1320005 MHz
NUC2                    1H
CPDPRG[2           waltz16
PCPD2                80.00 usec
PLW2           25.00000000 W
PLW12           0.39063001 W

F2 - Processing parameters
SI                   65536
SF             470.5923772 MHz
WDW                     EM
SSB      0
LB                    0.30 Hz
GB       0
PC                    1.00
```

Compound S2a

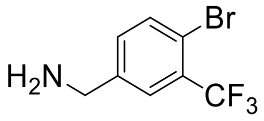

S2a

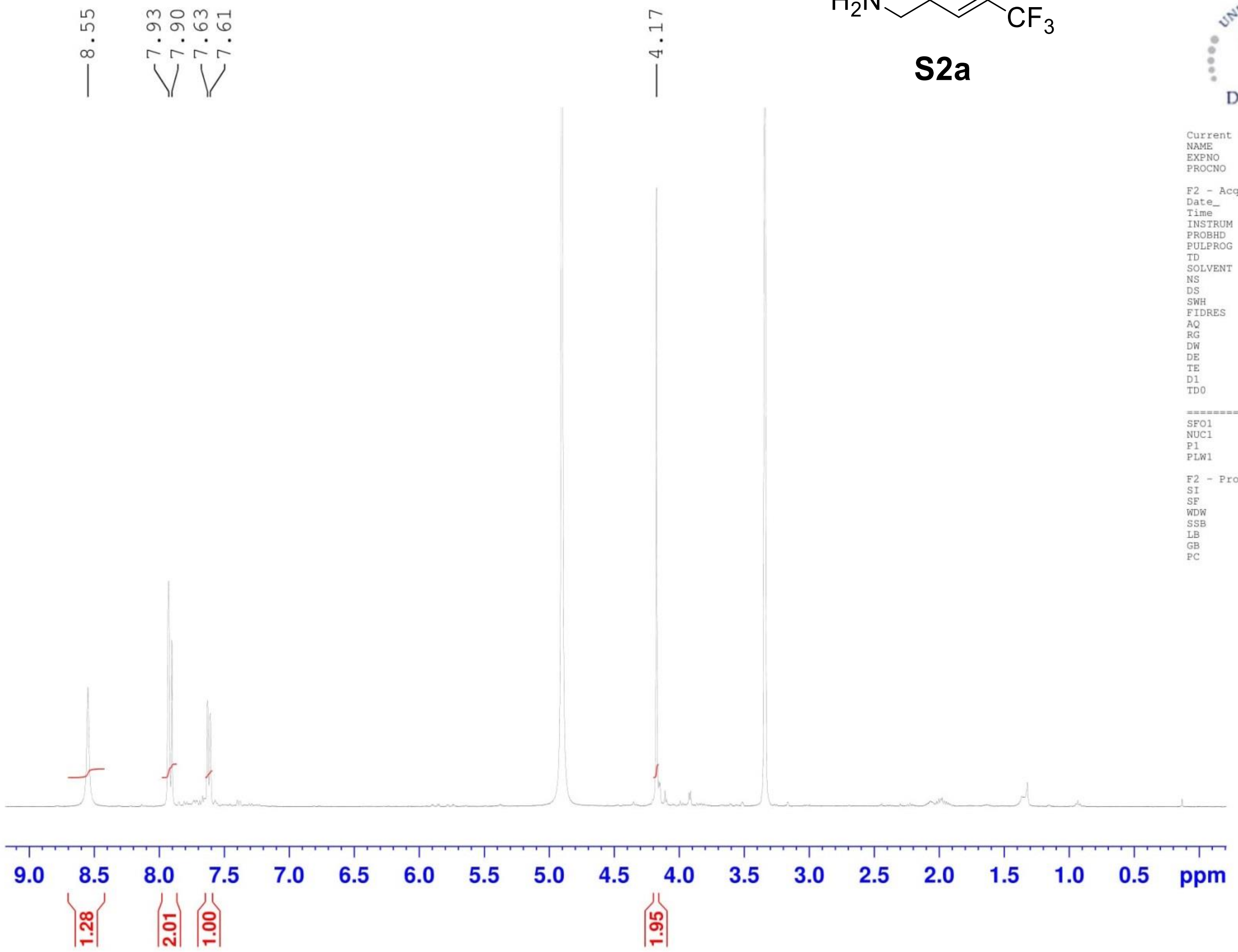

UNIVERSITY OF DUNDEE

```
Current Data Parameters
NAME          ALC-GC-S3A-3
EXPNO                   20
PROCNO                   1

F2 - Acquisition Parameters
Date_             20181123
Time                 21.31
INSTRUM              spect
PROBHD   5 mm PADUL 13C
PULPROG               zg30
TD                   65536
SOLVENT               MeOD
NS                      64
DS                       2
SWH               8000.000 Hz
FIDRES            0.122070 Hz
AQ               4.0960002 sec
RG                  196.14
DW                  62.500 usec
DE                   11.07 usec
TE                   294.6 K
D1              1.00000000 sec
TD0                      1

======== CHANNEL f1 ========
SFO1           400.1324710 MHz
NUC1                    1H
P1                   10.00 usec
PLW1           20.00000000 W

F2 - Processing parameters
SI                   65536
SF             400.1299954 MHz
WDW                     EM
SSB      0
LB                    0.30 Hz
GB       0
PC                    1.00
```

Compound S2a

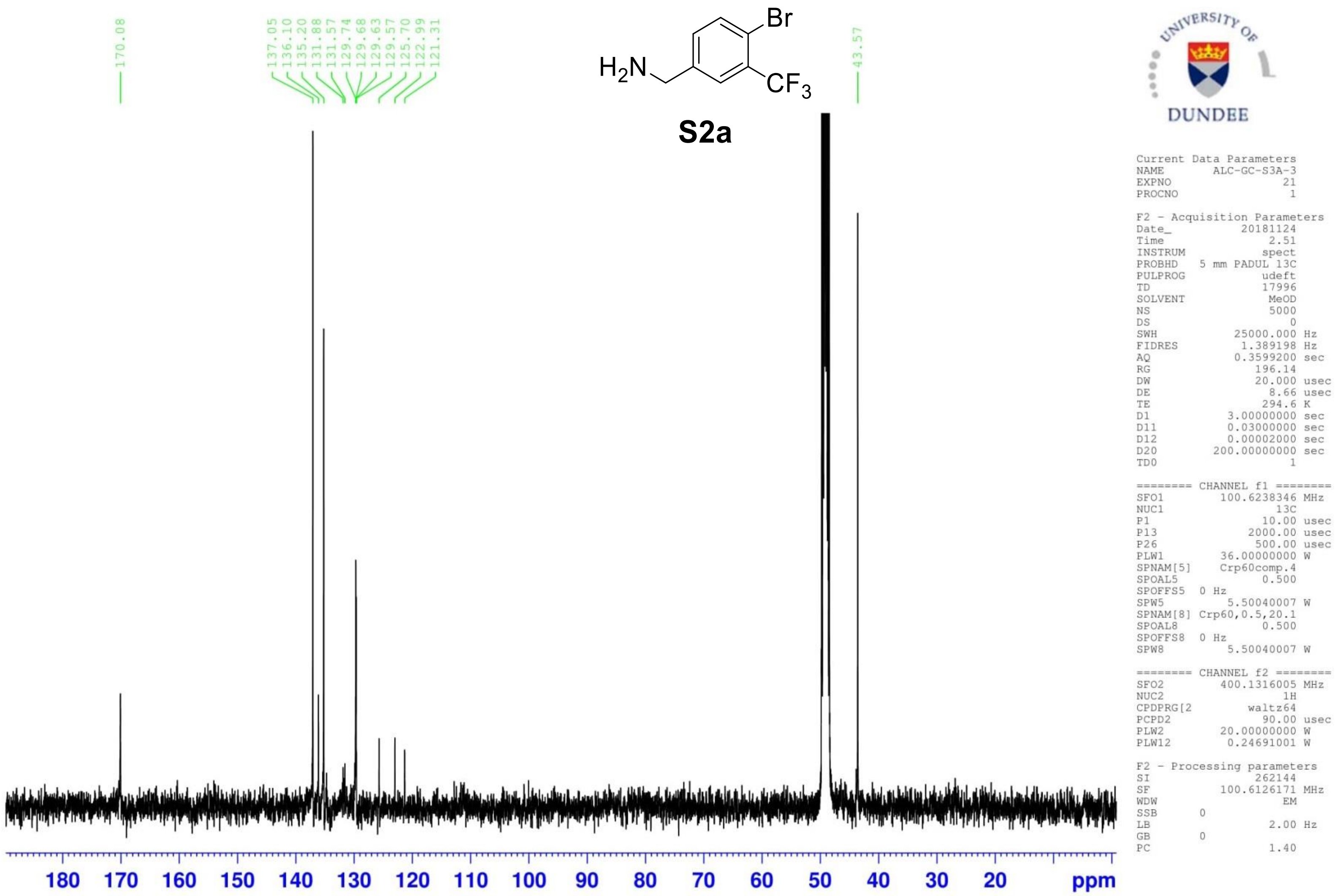

Compound S2a

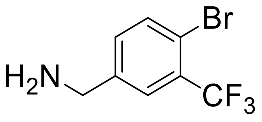

S2a

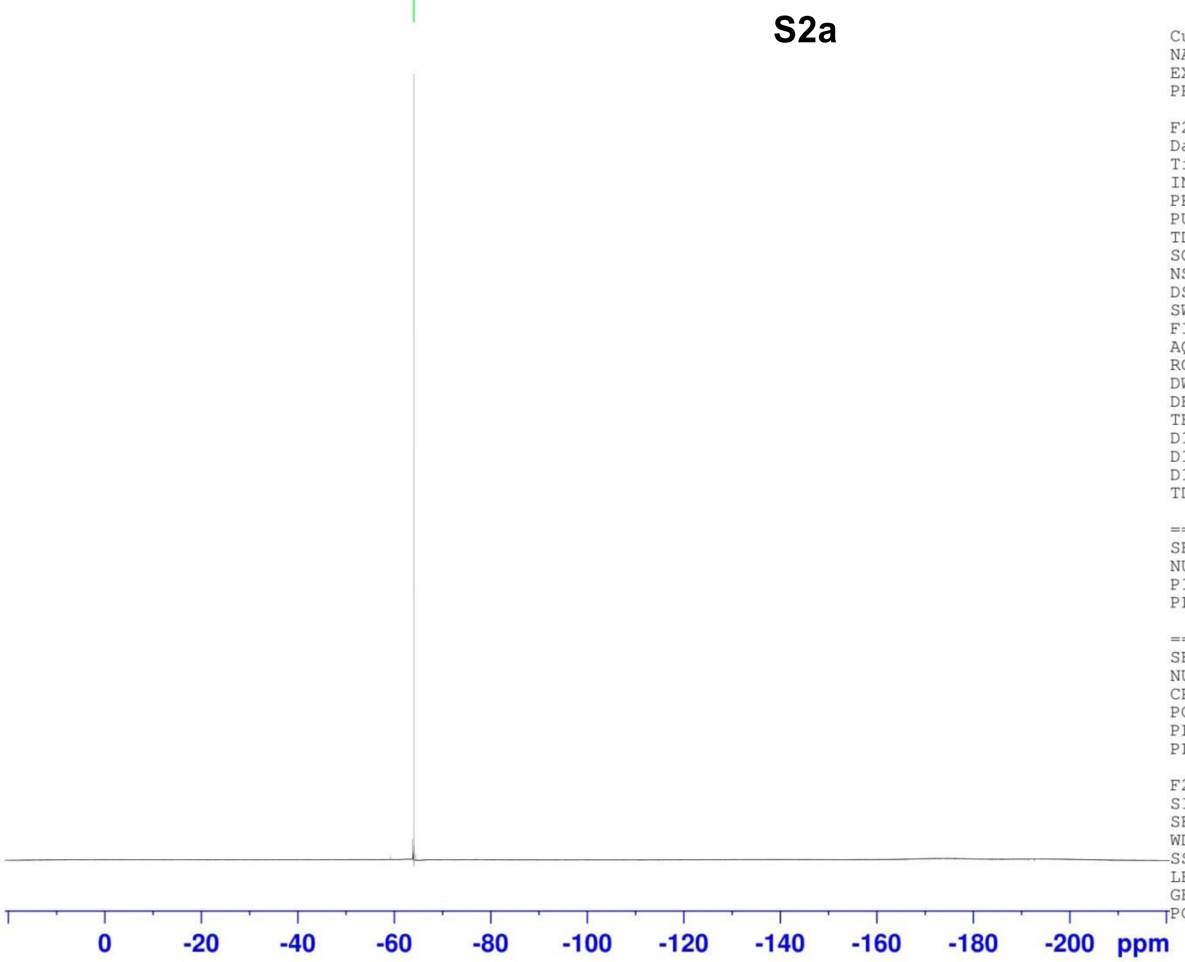


```
Current Data Parameters
NAME            GC-S2A-F3
EXPNO                  10
PROCNO                  1

F2 - Acquisition Parameters
Date_            20181126
Time                 9.55
INSTRUM             spect
PROBHD    5 mm PABBO BB/
PULPROG       zgfhigqn.2
TD                 131072
SOLVENT              MeOD
NS                     16
DS                      4
SWH            113636.367 Hz
FIDRES           0.866977 Hz
AQ              0.5767168 sec
RG                    322
DW                  4.400 usec
DE                   6.50 usec
TE                  303.4 K
D1             1.00000000 sec
D11            0.03000000 sec
D12            0.00002000 sec
TD0                     1

======== CHANNEL f1 ========
SFO1         470.5453180 MHz
NUC1                 19F
P1                  14.50 usec
PLW1          45.00000000 W

======== CHANNEL f2 ========
SFO2         500.1320005 MHz
NUC2                  1H
CPDPRG[2          waltz16
PCPD2               80.00 usec
PLW2          20.85000038 W
PLW12          0.35916999 W

F2 - Processing parameters
SI                  65536
SF            470.5923772 MHz
WDW                    EM
SSB      0
LB                   0.30 Hz
GB       0
PC                   1.00
```

Compound S2b

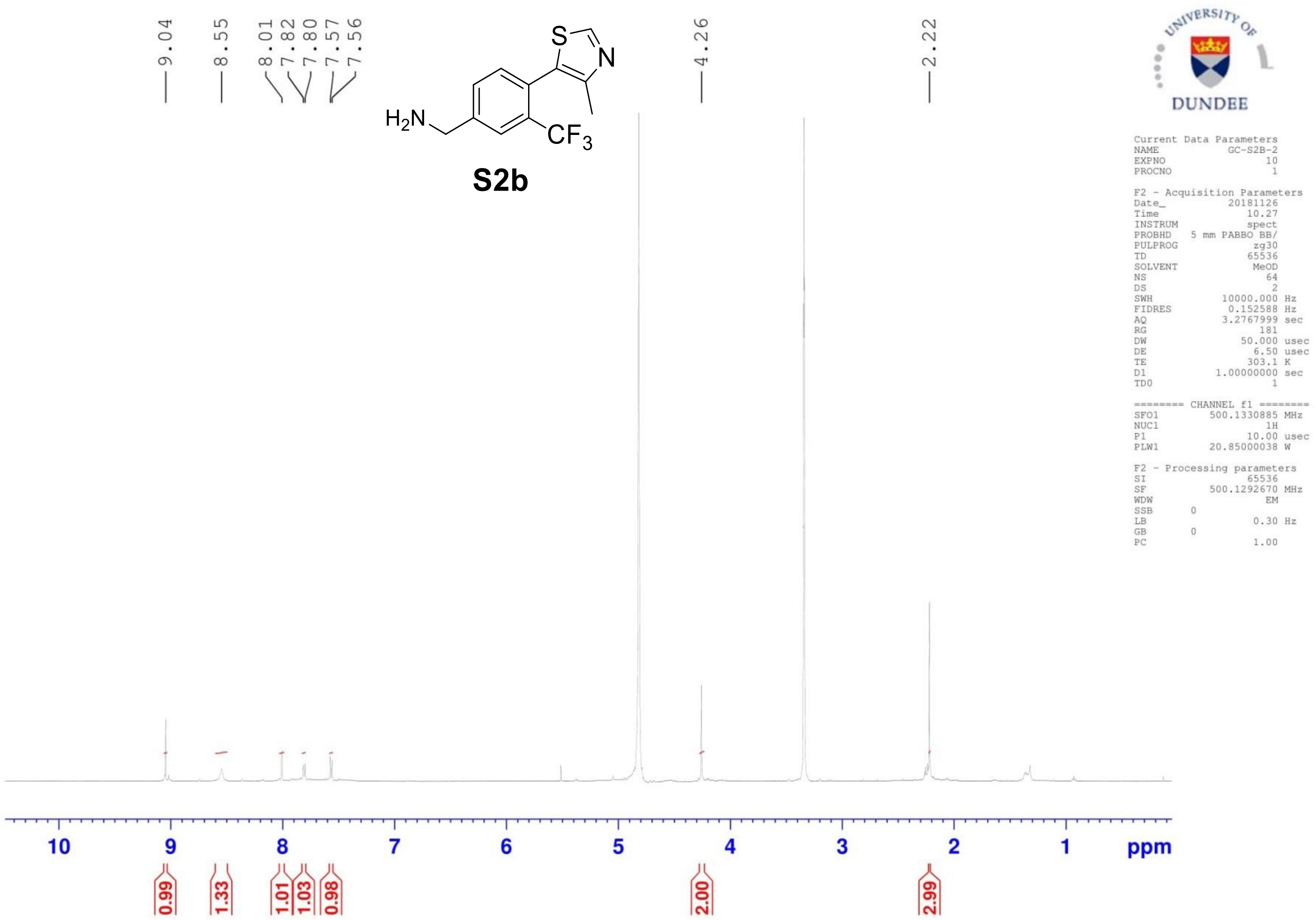

Compound S2b

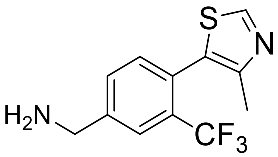

S2b

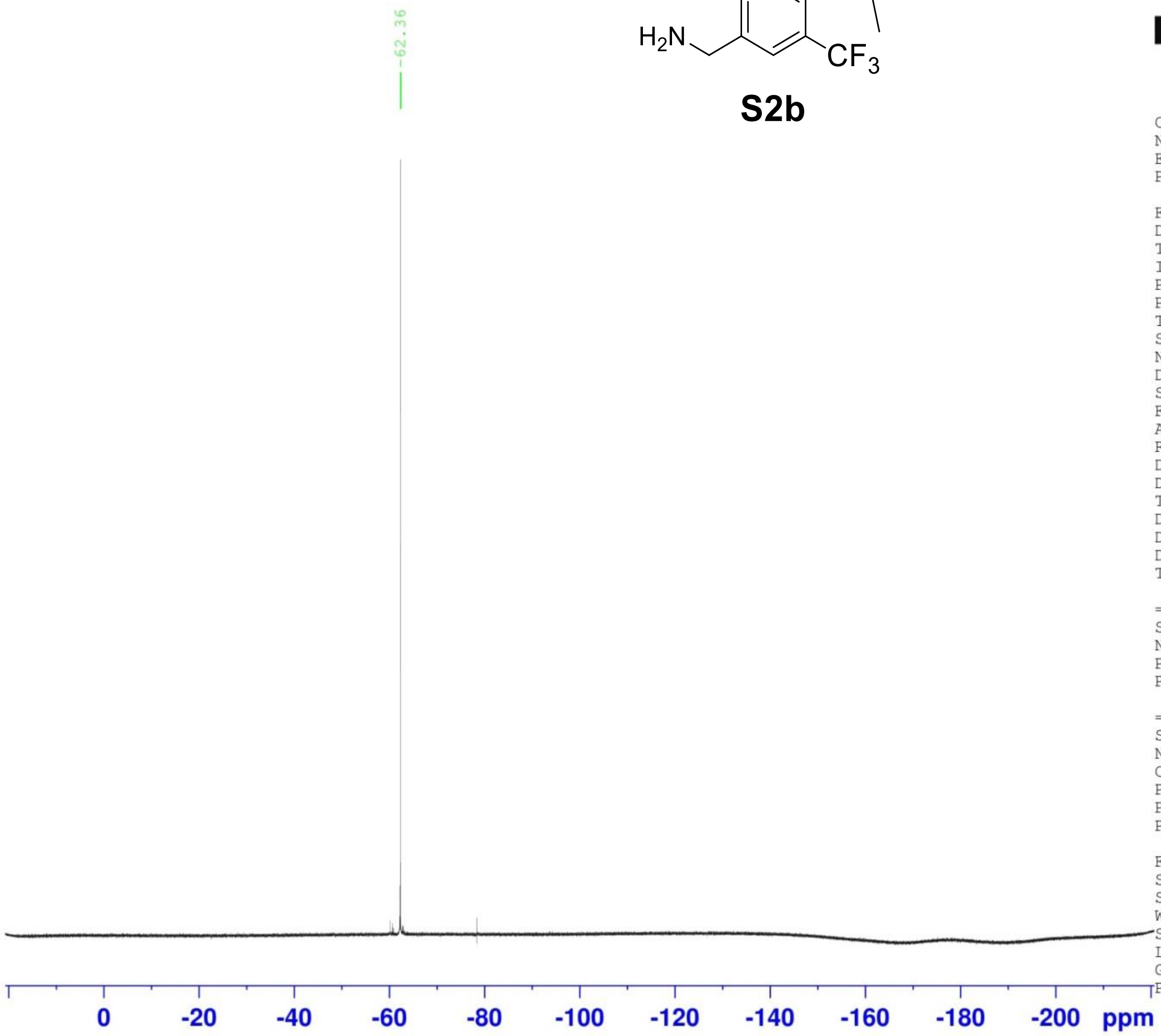


```
Current Data Parameters
NAME              GC-S2B-2
EXPNO                   11
PROCNO                   1

F2 - Acquisition Parameters
Date_             20181126
Time                 10.29
INSTRUM              spect
PROBHD    5 mm PABBO BB/
PULPROG        zgfhigqn.2
TD                  131072
SOLVENT               MeOD
NS                      16
DS                       4
SWH             113636.367 Hz
FIDRES            0.866977 Hz
AQ               0.5767168 sec
RG                     322
DW                   4.400 usec
DE                    6.50 usec
TE                   303.4 K
D1              1.00000000 sec
D11             0.03000000 sec
D12             0.00002000 sec
TD0                      1

======== CHANNEL f1 ========
SFO1           470.5453180 MHz
NUC1                   19F
P1                   14.50 usec
PLW1           45.00000000 W

======== CHANNEL f2 ========
SFO2           500.1320005 MHz
NUC2                    1H
CPDPRG[2           waltz16
PCPD2                80.00 usec
PLW2           20.85000038 W
PLW12           0.35916999 W

F2 - Processing parameters
SI                   65536
SF             470.5923772 MHz
WDW                     EM
SSB       0
LB                    0.30 Hz
GB        0
PC                    1.00
```

Compound S2c

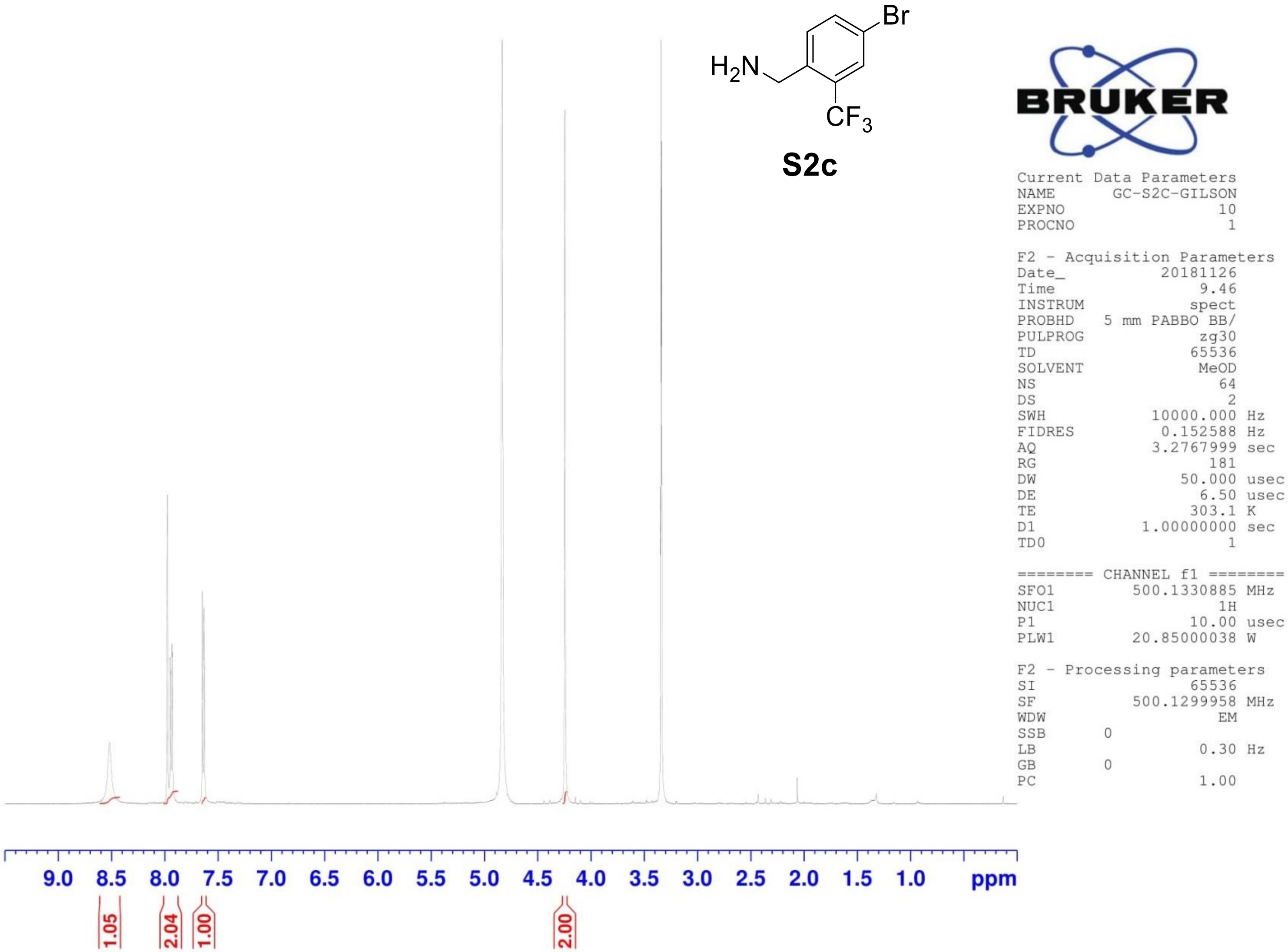

Compound S2c

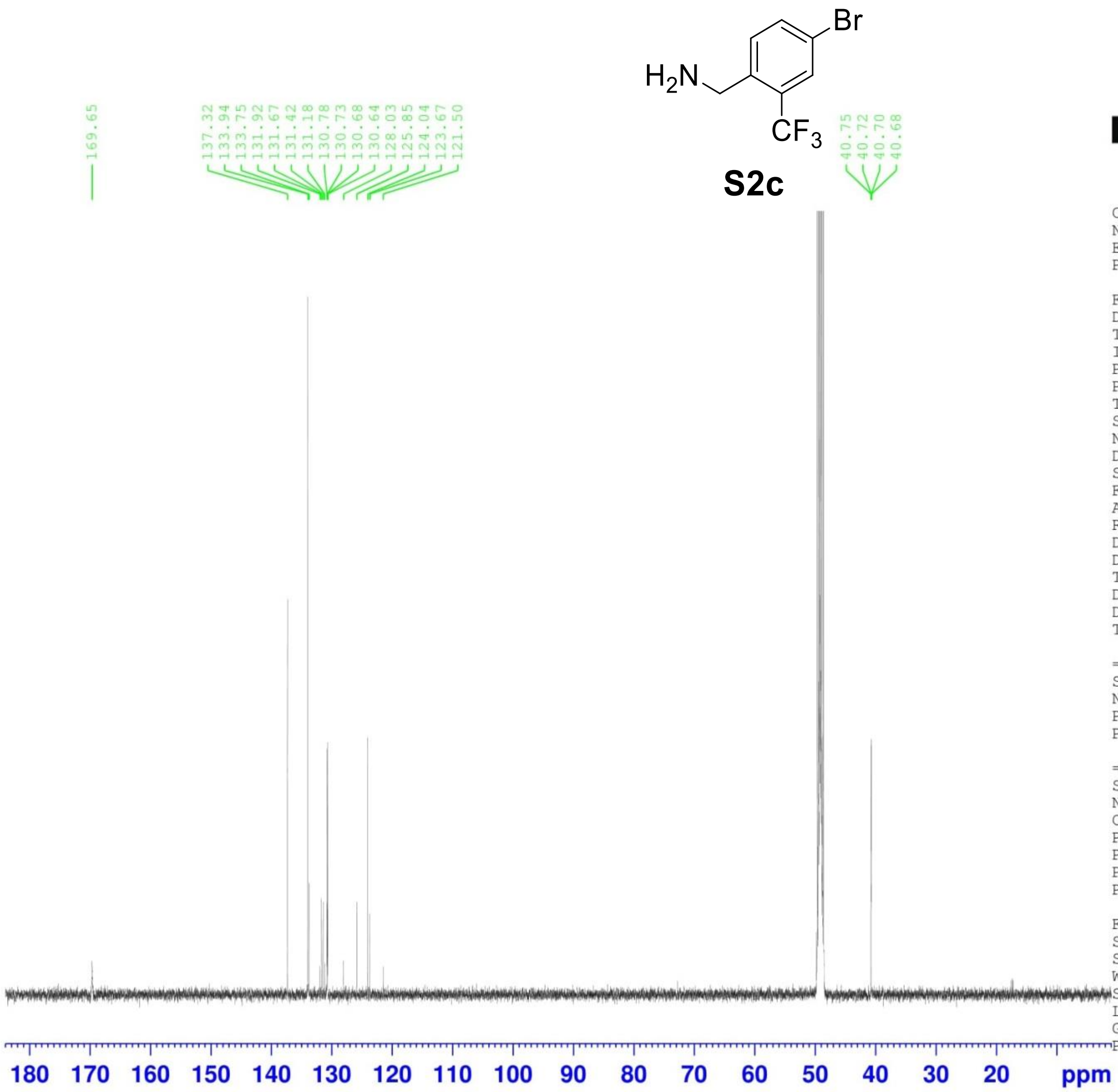


```
Current Data Parameters
NAME            GC-S2C+
EXPNO                11
PROCNO                1

F2 - Acquisition Parameters
Date_          20181126
Time              11.50
INSTRUM           spect
PROBHD   5 mm CPQCI 1H/
PULPROG         zgpg30
TD                65400
SOLVENT            MeOD
NS                 2048
DS                    4
SWH           29761.904 Hz
FIDRES         0.455075 Hz
AQ            1.0987200 sec
RG               190.78
DW               16.800 usec
DE                18.00 usec
TE                303.1 K
D1           2.00000000 sec
D11          0.03000000 sec
TD0                   1

======== CHANNEL f1 ========
SFO1        125.7703637 MHz
NUC1                13C
P1                12.00 usec
PLW1       133.00000000 W

======== CHANNEL f2 ========
SFO2        500.1320005 MHz
NUC2                 1H
CPDPRG[2        waltz16
PCPD2            100.00 usec
PLW2         5.19999981 W
PLW12        0.06406900 W
PLW13        0.06406900 W

F2 - Processing parameters
SI               131072
SF          125.7575991 MHz
WDW                  EM
SSB      0
LB                 1.00 Hz
GB       0
PC                 1.40
```

Compound S2c

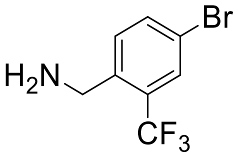

S2c

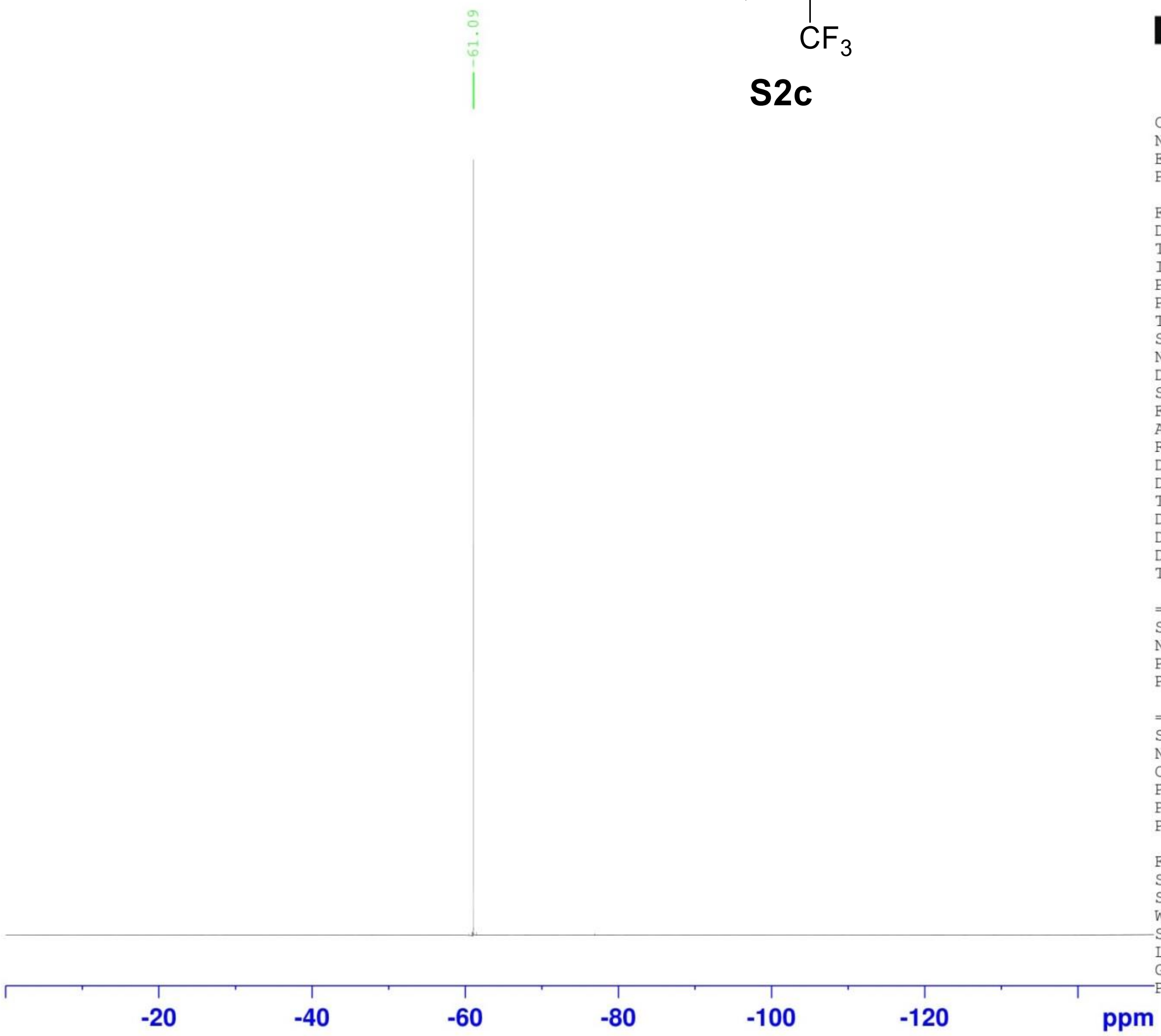

Current Data Parameters
NAME GC-S2C-GILSON
EXPNO 11
PROCNO 1

F2 - Acquisition Parameters
Date_ 20181126
Time 9.49
INSTRUM spect
PROBHD 5 mm PABBO BB/
PULPROG zgfhigqn.2
TD 131072
SOLVENT MeOD
NS 16
DS 4
SWH 113636.367 Hz
FIDRES 0.866977 Hz
AQ 0.5767168 sec
RG 322
DW 4.400 usec
DE 6.50 usec
TE 303.4 K
D1 1.00000000 sec
D11 0.03000000 sec
D12 0.00002000 sec
TD0 1

======== CHANNEL f1 ========
SFO1 470.5453180 MHz
NUC1 19F
P1 14.50 usec
PLW1 45.00000000 W

======== CHANNEL f2 ========
SFO2 500.1320005 MHz
NUC2 1H
CPDPRG[2 waltz16
PCPD2 80.00 usec
PLW2 20.85000038 W
PLW12 0.35916999 W

F2 - Processing parameters
SI 65536
SF 470.5923772 MHz
WDW EM
SSB 0
LB 0.30 Hz
GB 0
PC 1.00

Compound S2d

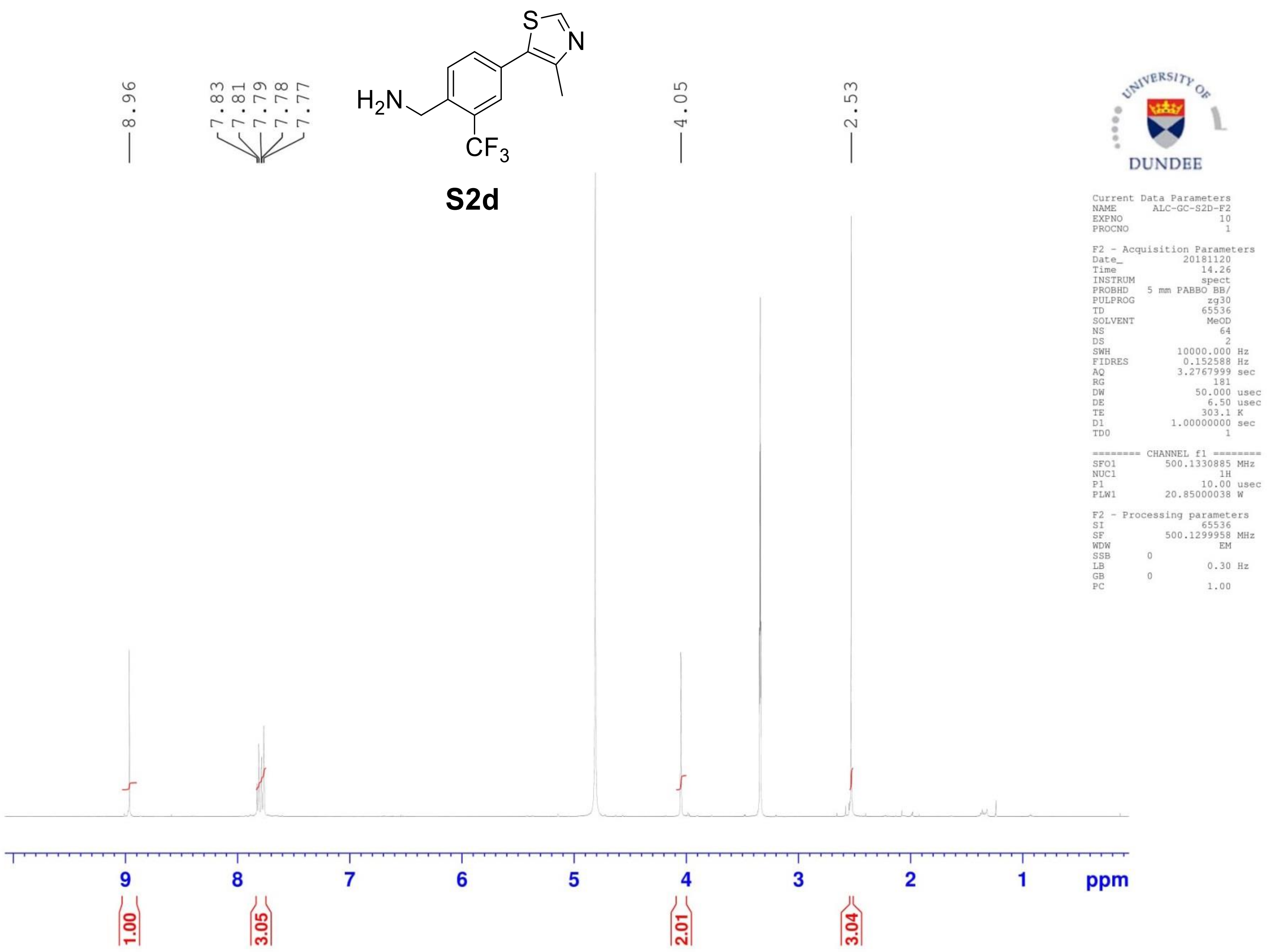

Compound S2d

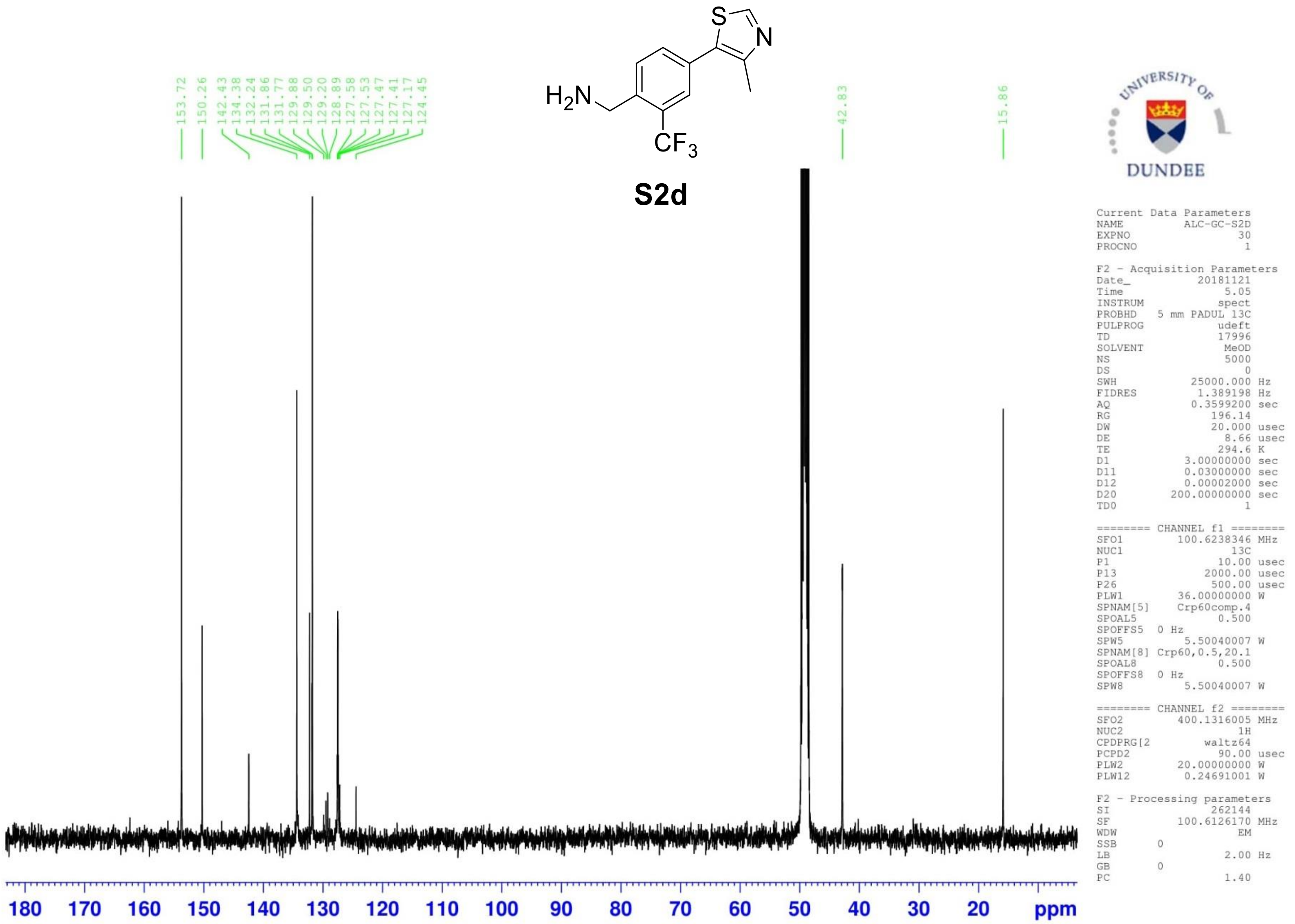

Compound S2d

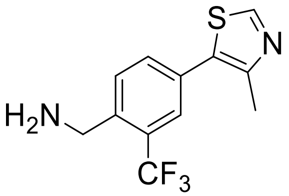

S2d

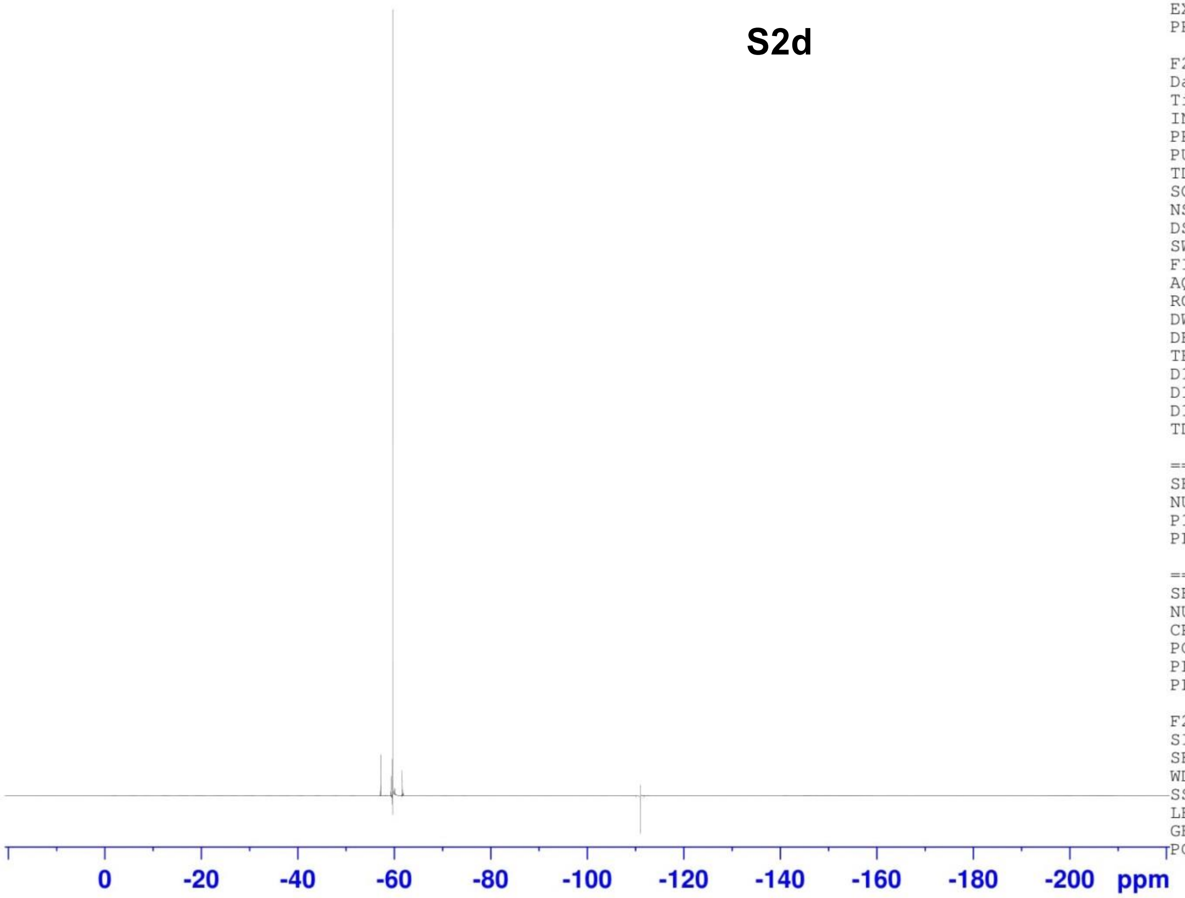


```
Current Data Parameters
NAME              GC-R049
EXPNO                   2
PROCNO                  1

F2 - Acquisition Parameters
Date_            20160519
Time                13.52
INSTRUM             spect
PROBHD   5 mm QNP 1H/13
PULPROG       zgfhigqn.2
TD                 131072
SOLVENT              MeOD
NS                     16
DS                      4
SWH            113636.367 Hz
FIDRES           0.866977 Hz
AQ              0.5767168 sec
RG                    203
DW                  4.400 usec
DE                   6.50 usec
TE                  298.2 K
D1             1.00000000 sec
D11            0.03000000 sec
D12            0.00002000 sec
TD0                     1

======== CHANNEL f1 ========
SFO1         470.5453180 MHz
NUC1                  19F
P1                  13.50 usec
PLW1          25.00000000 W

======== CHANNEL f2 ========
SFO2         500.1320005 MHz
NUC2                   1H
CPDPRG[2          waltz16
PCPD2               80.00 usec
PLW2          25.00000000 W
PLW12          0.39063001 W

F2 - Processing parameters
SI                  65536
SF           470.5923772 MHz
WDW                    EM
SSB       0
LB                   0.30 Hz
GB        0
PC                   1.00
```

Spy molecule 1 (Compound S3a)

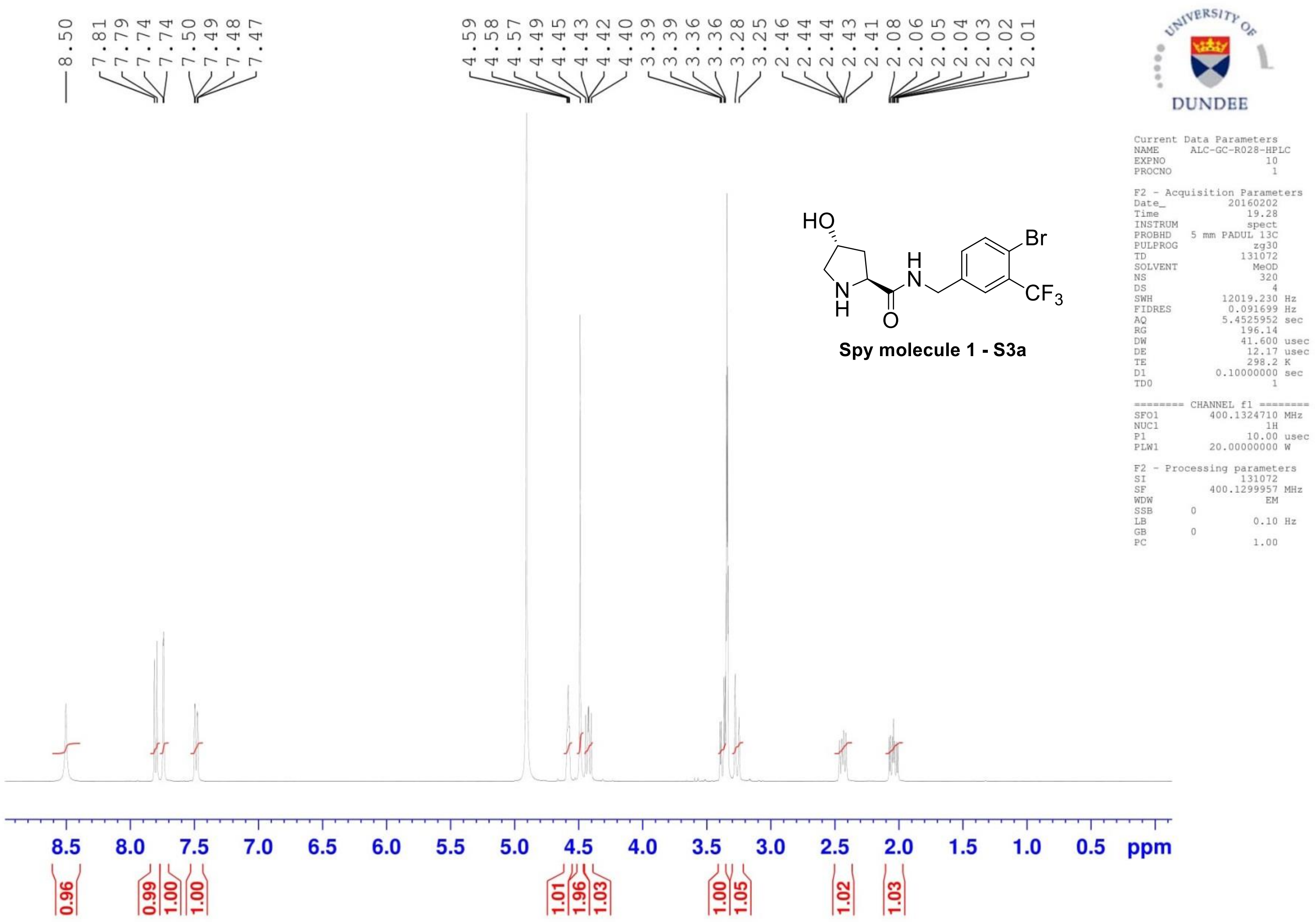

Spy molecule 1 (Compound S3a)

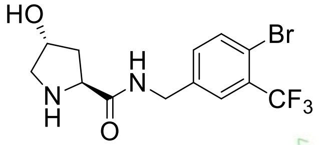

Spy molecule 1 - S3a

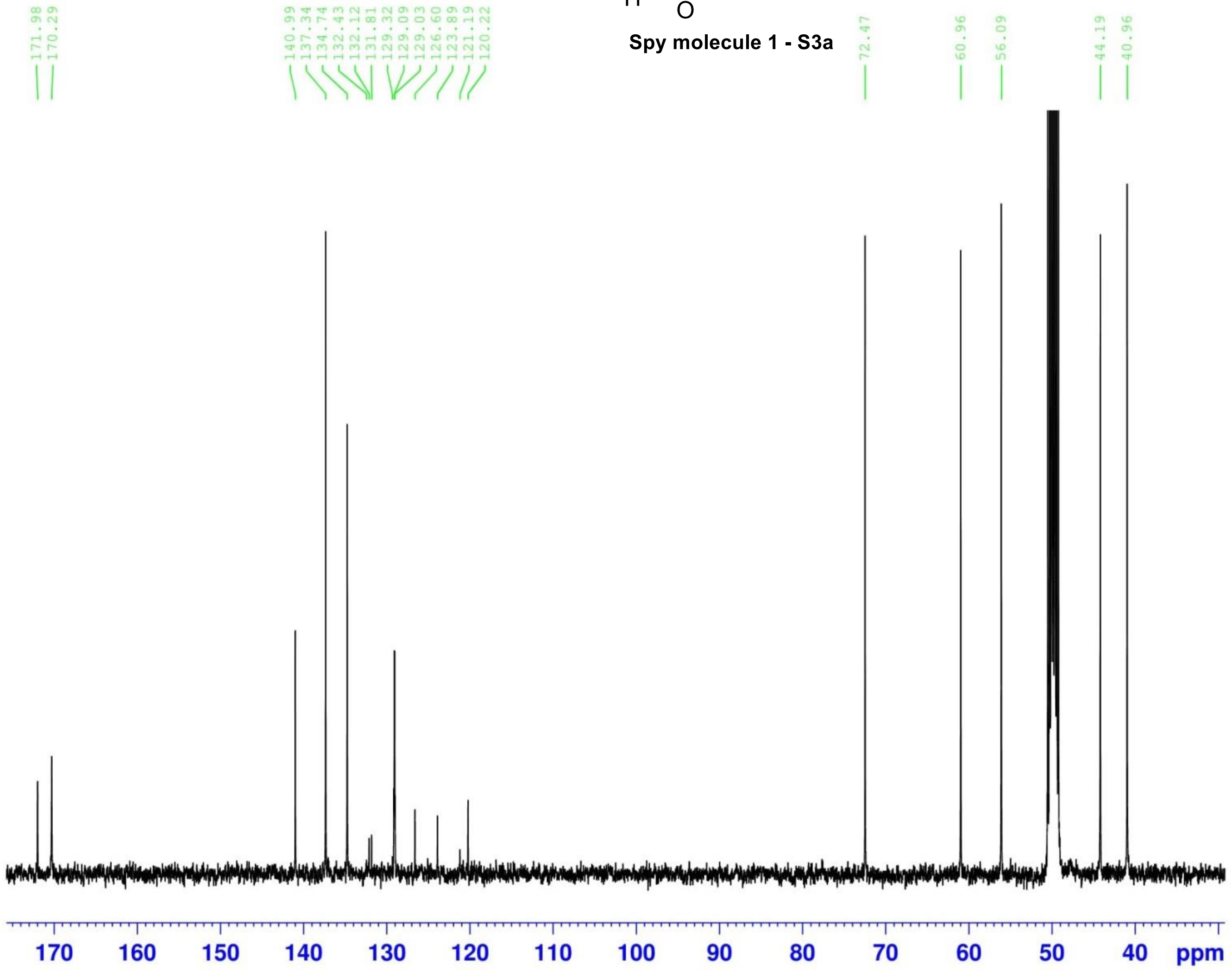

UNIVERSITY OF DUNDEE

```
Current Data Parameters
NAME       ALC-GC-R028-HPLC
EXPNO                    11
PROCNO                    1

F2 - Acquisition Parameters
Date_              20160203
Time                   0.07
INSTRUM               spect
PROBHD     5 mm PADUL 13C
PULPROG               udeft
TD                    17996
SOLVENT                MeOD
NS                     2000
DS                        0
SWH             25000.000 Hz
FIDRES           1.389198 Hz
AQ              0.3599200 sec
RG                   196.14
DW                  20.000 usec
DE                    8.66 usec
TE                   298.2 K
D1              3.00000000 sec
D11             0.03000000 sec
D12             0.00002000 sec
D20           200.00000000 sec
TD0                       1

======== CHANNEL f1 ========
SFO1          100.6238346 MHz
NUC1                    13C
P1                    10.00 usec
P13                 2000.00 usec
P26                  500.00 usec
PLW1            36.00000000 W
SPNAM[5]        Crp60comp.4
SPOAL5                0.500
SPOFFS5    0 Hz
SPW5             5.50040007 W
SPNAM[8]   Crp60,0.5,20.1
SPOAL8                0.500
SPOFFS8    0 Hz
SPW8             5.50040007 W

======== CHANNEL f2 ========
SFO2          400.1316005 MHz
NUC2                     1H
CPDPRG[2            waltz64
PCPD2                 90.00 usec
PLW2            20.00000000 W
PLW12            0.24691001 W

F2 - Processing parameters
SI                   262144
SF            100.6125411 MHz
WDW                      EM
SSB        0
LB                     2.00 Hz
GB         0
PC                     1.40
```

Spy molecule 1 (Compound S3a)

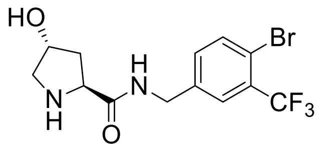

Spy molecule 1 - S3a

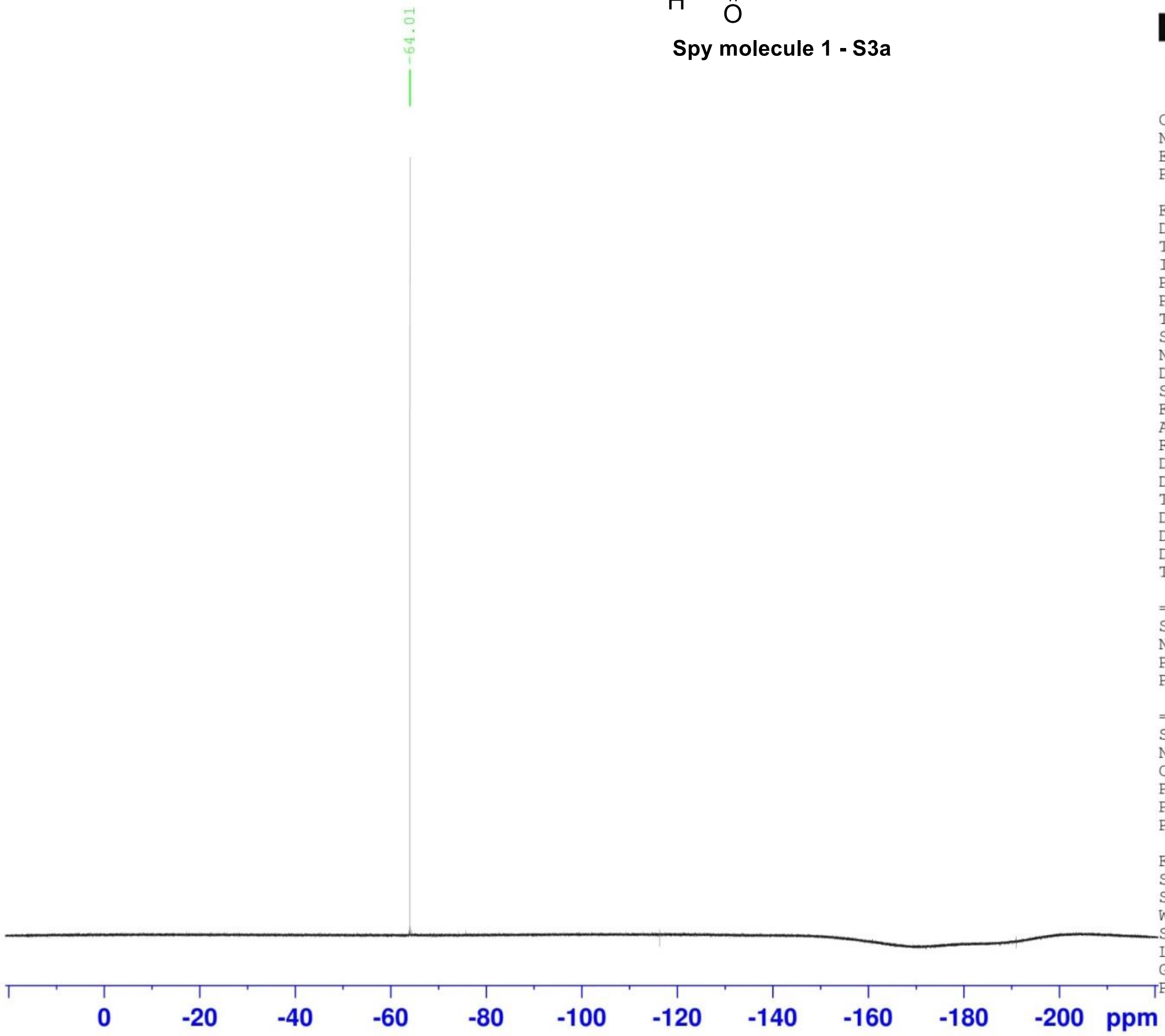


```
Current Data Parameters
NAME              AC-GC-R028
EXPNO                      1
PROCNO                     1

F2 - Acquisition Parameters
Date_               20160125
Time                    9.49
INSTRUM                spect
PROBHD      5 mm QNP 1H/13
PULPROG          zgfhigqn.2
TD                    131072
SOLVENT                 MeOD
NS                        64
DS                         4
SWH               113636.367 Hz
FIDRES              0.866977 Hz
AQ                 0.5767168 sec
RG                       456
DW                     4.400 usec
DE                      6.50 usec
TE                     298.2 K
D1                1.00000000 sec
D11               0.03000000 sec
D12               0.00002000 sec
TD0                        1

======== CHANNEL f1 ========
SFO1             470.5453180 MHz
NUC1                     19F
P1                     11.50 usec
PLW1             25.00000000 W

======== CHANNEL f2 ========
SFO2             500.1320005 MHz
NUC2                      1H
CPDPRG[2             waltz16
PCPD2                  80.00 usec
PLW2             25.00000000 W
PLW12             0.39063001 W

F2 - Processing parameters
SI                     65536
SF               470.5923772 MHz
WDW                       EM
SSB      0
LB                      0.30 Hz
GB       0
PC                      1.00
```

Spy molecule 5 (Compound S3b)

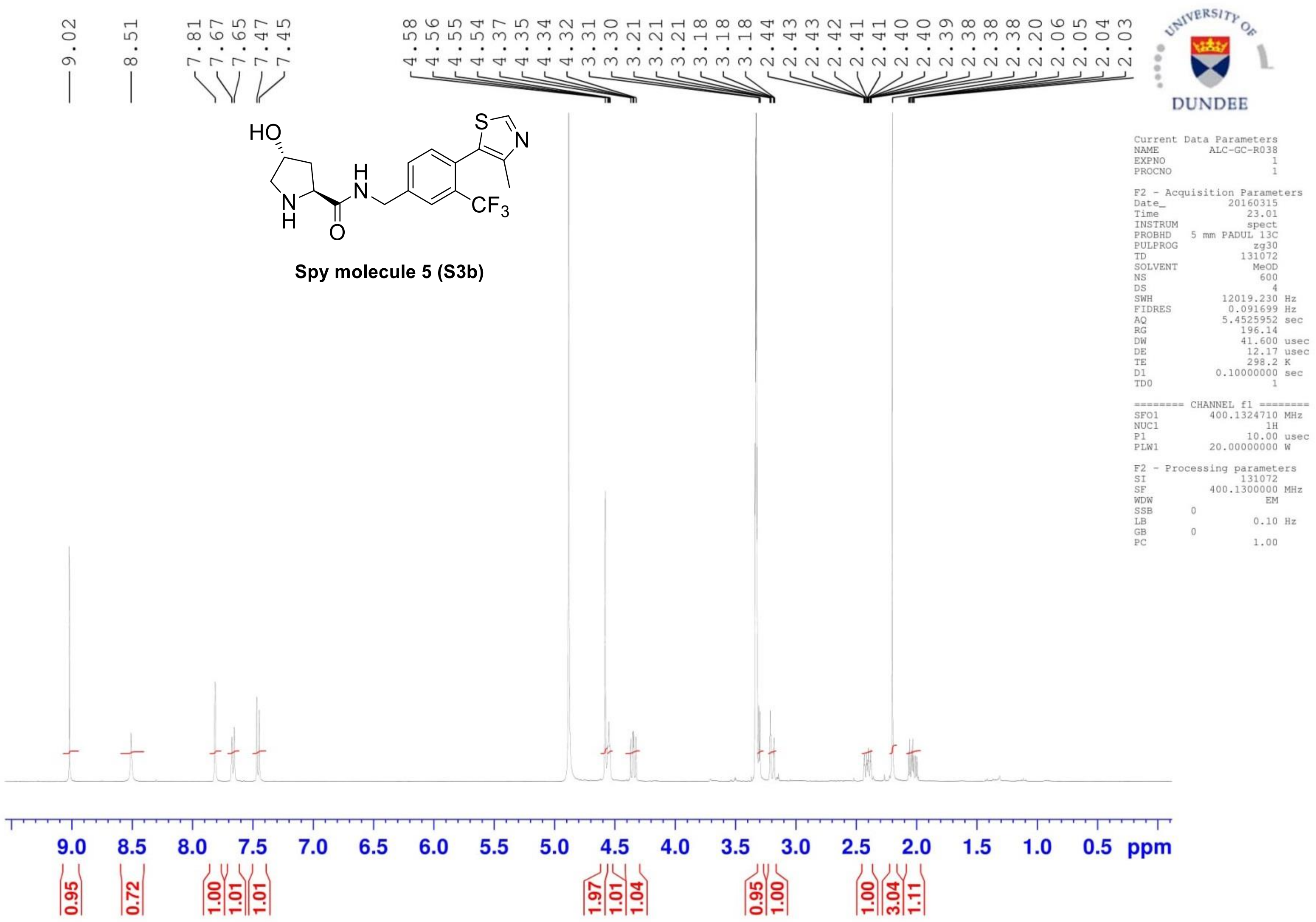

Spy molecule 5 (Compound S3b)

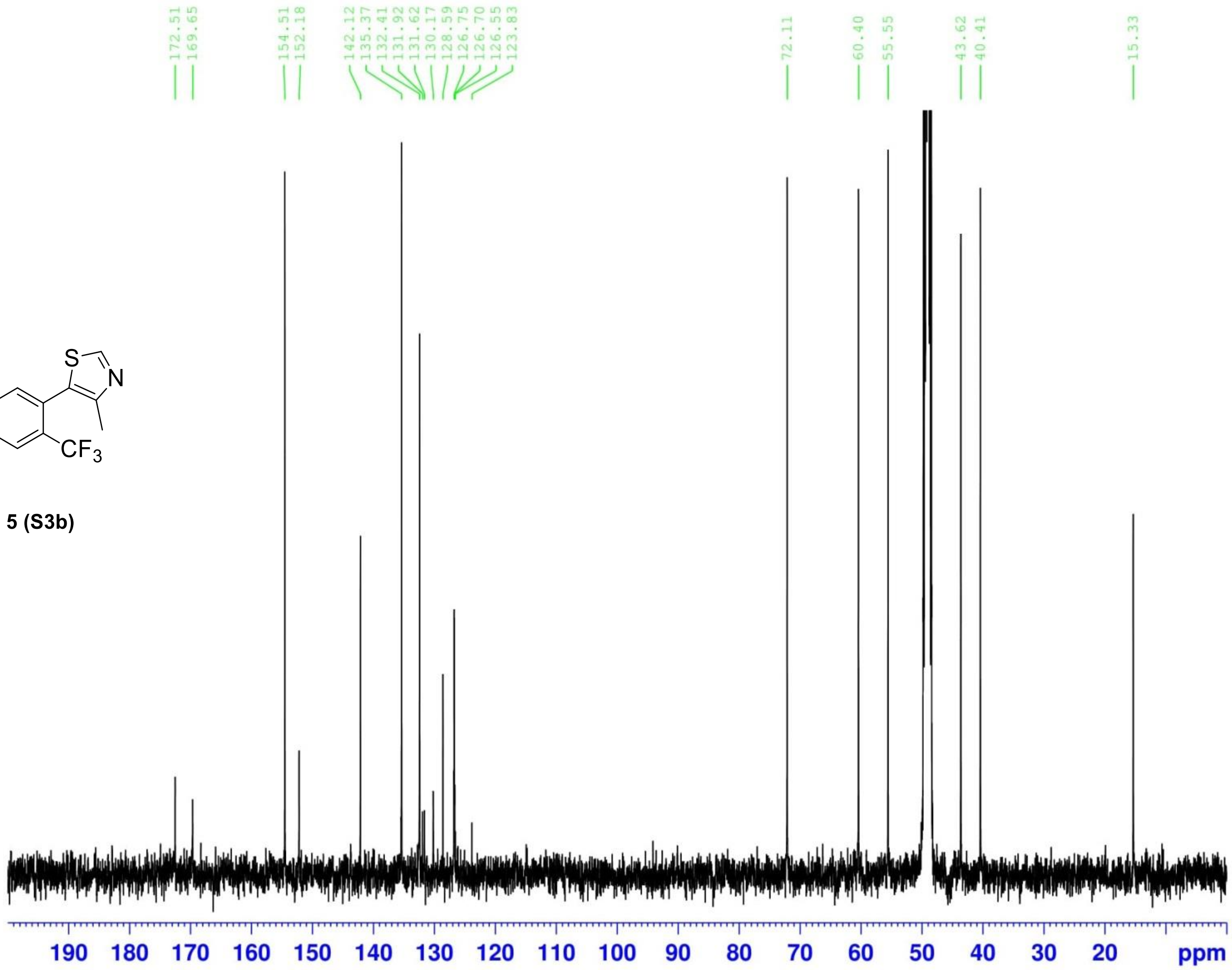

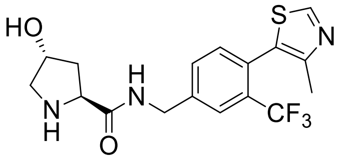

Spy molecule 5 (S3b)

UNIVERSITY OF DUNDEE

Current Data Parameters
NAME ALC-GC-R038
EXPNO 2
PROCNO 1

F2 - Acquisition Parameters
Date_ 20160316
Time 5.10
INSTRUM spect
PROBHD 5 mm PADUL 13C
PULPROG udeft
TD 17996
SOLVENT MeOD
NS 5800
DS 0
SWH 25000.000 Hz
FIDRES 1.389198 Hz
AQ 0.3599200 sec
RG 196.14
DW 20.000 usec
DE 8.66 usec
TE 298.2 K
D1 3.00000000 sec
D11 0.03000000 sec
D12 0.00002000 sec
D20 200.00000000 sec
TD0 1

======== CHANNEL f1 ========
SFO1 100.6238346 MHz
NUC1 13C
P1 10.00 usec
P13 2000.00 usec
P26 500.00 usec
PLW1 36.00000000 W
SPNAM[5] Crp60comp.4
SPOAL5 0.500
SPOFFS5 0 Hz
SPW5 5.50040007 W
SPNAM[8] Crp60,0.5,20.1
SPOAL8 0.500
SPOFFS8 0 Hz
SPW8 5.50040007 W

======== CHANNEL f2 ========
SFO2 400.1316005 MHz
NUC2 1H
CPDPRG[2 waltz64
PCPD2 90.00 usec
PLW2 20.00000000 W
PLW12 0.24691001 W

F2 - Processing parameters
SI 262144
SF 100.6126170 MHz
WDW EM
SSB 0
LB 2.00 Hz
GB 0
PC 1.40

Spy molecule 5 (Compound S3b)

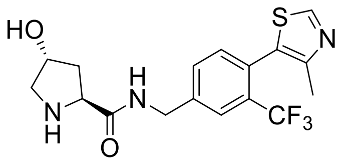

Spy molecule 5 (S3b)

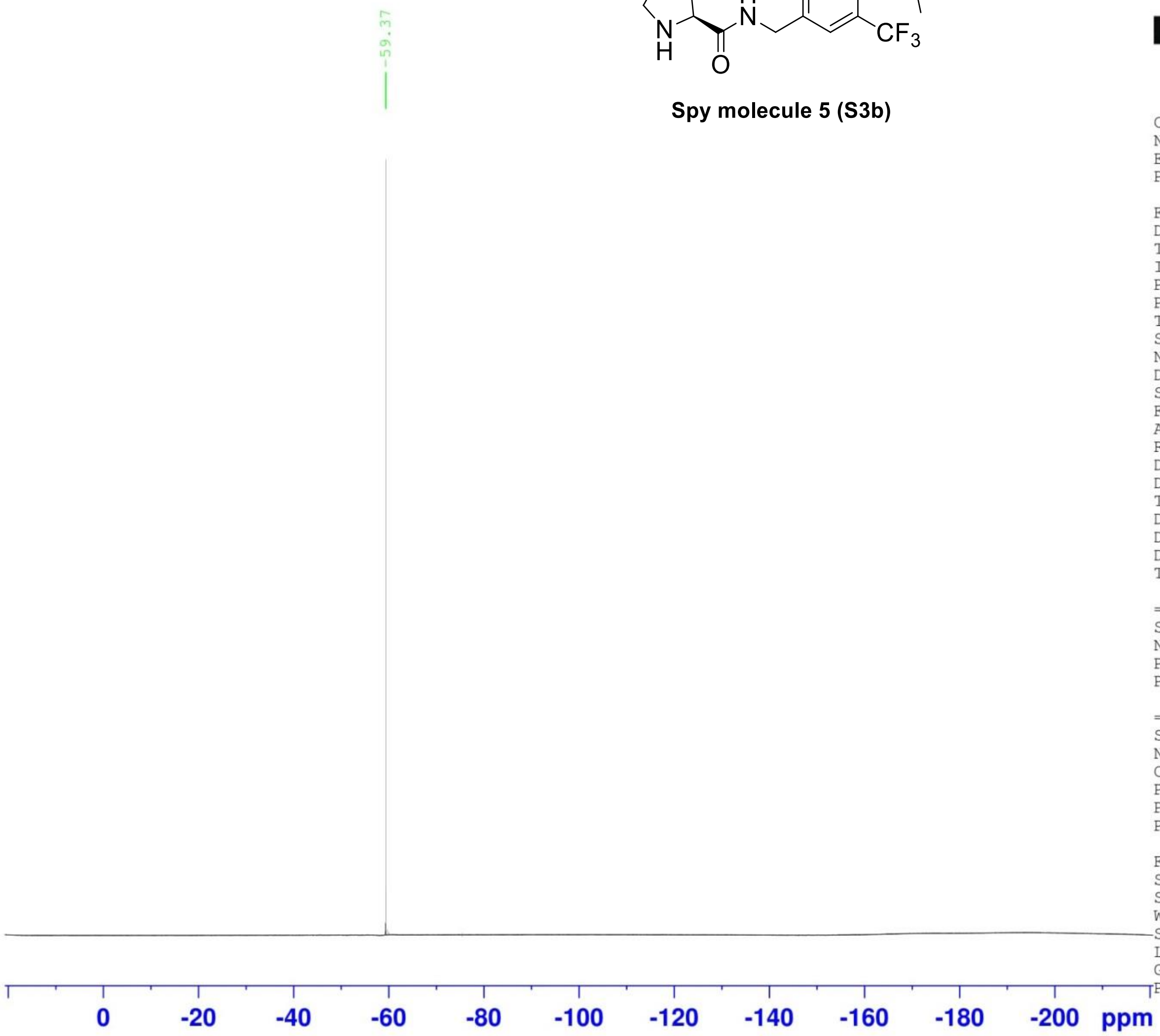


```
Current Data Parameters
NAME            AC-GC-R038
EXPNO                    1
PROCNO                   1

F2 - Acquisition Parameters
Date_             20160316
Time                 14.35
INSTRUM              spect
PROBHD     5 mm QNP 1H/13
PULPROG         zgfhigqn.2
TD                  131072
SOLVENT               MeOD
NS                      64
DS                       4
SWH             113636.367 Hz
FIDRES            0.866977 Hz
AQ               0.5767168 sec
RG                     812
DW                   4.400 usec
DE                    6.50 usec
TE                   295.0 K
D1              1.00000000 sec
D11             0.03000000 sec
D12             0.00002000 sec
TD0                      1

======== CHANNEL f1 ========
SFO1           470.5453180 MHz
NUC1                   19F
P1                   13.50 usec
PLW1           25.00000000 W

======== CHANNEL f2 ========
SFO2           500.1320005 MHz
NUC2                    1H
CPDPRG[2           waltz16
PCPD2                80.00 usec
PLW2           25.00000000 W
PLW12           0.39063001 W

F2 - Processing parameters
SI                   65536
SF             470.5923772 MHz
WDW                     EM
SSB      0
LB                    0.30 Hz
GB       0
PC                    1.00
```

Spy molecule 9 (Compound S3c)

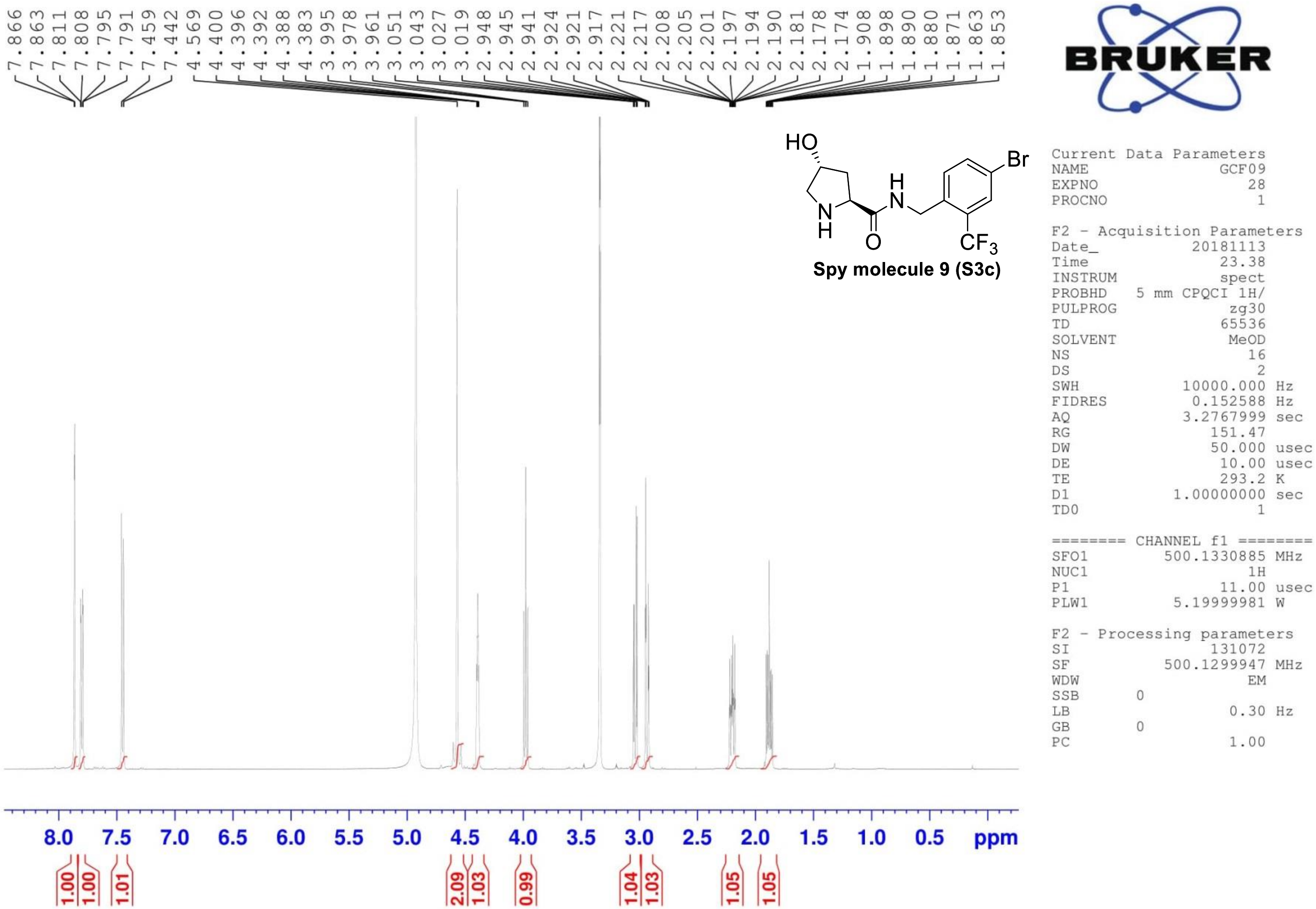

Spy molecule 9 (Compound S3c)

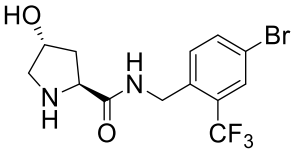

Spy molecule 9 (S3c)

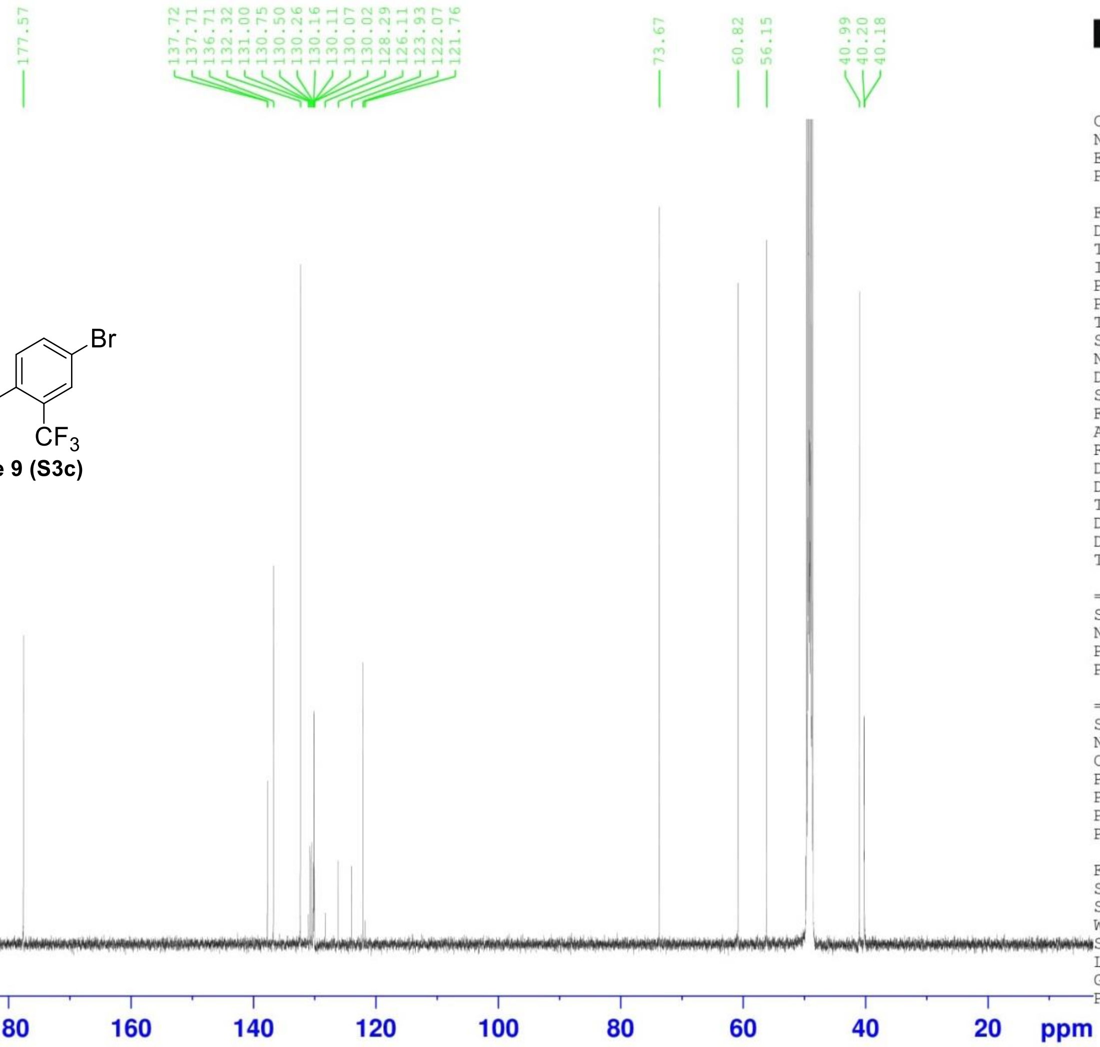


```
Current Data Parameters
NAME              GCF09
EXPNO                31
PROCNO                1

F2 - Acquisition Parameters
Date_          20181116
Time              14.33
INSTRUM           spect
PROBHD   5 mm CPQCI 1H/
PULPROG          zgpg30
TD                65400
SOLVENT            MeOD
NS                 4000
DS                    4
SWH           29761.904 Hz
FIDRES         0.455075 Hz
AQ            1.0987200 sec
RG               190.78
DW               16.800 usec
DE                18.00 usec
TE                293.1 K
D1           2.00000000 sec
D11          0.03000000 sec
TD0                   1

======== CHANNEL f1 ========
SFO1        125.7703637 MHz
NUC1                13C
P1                12.00 usec
PLW1       133.00000000 W

======== CHANNEL f2 ========
SFO2        500.1320005 MHz
NUC2                 1H
CPDPRG[2        waltz16
PCPD2            100.00 usec
PLW2         5.19999981 W
PLW12        0.06406900 W
PLW13        0.06406900 W

F2 - Processing parameters
SI               131072
SF          125.7575994 MHz
WDW                  EM
SSB      0
LB                 1.00 Hz
GB       0
PC                 1.40
```

Spy molecule 9 (Compound S3c)

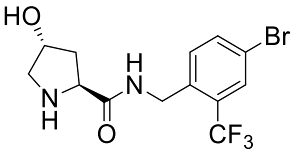

Spy molecule 9 (S3c)

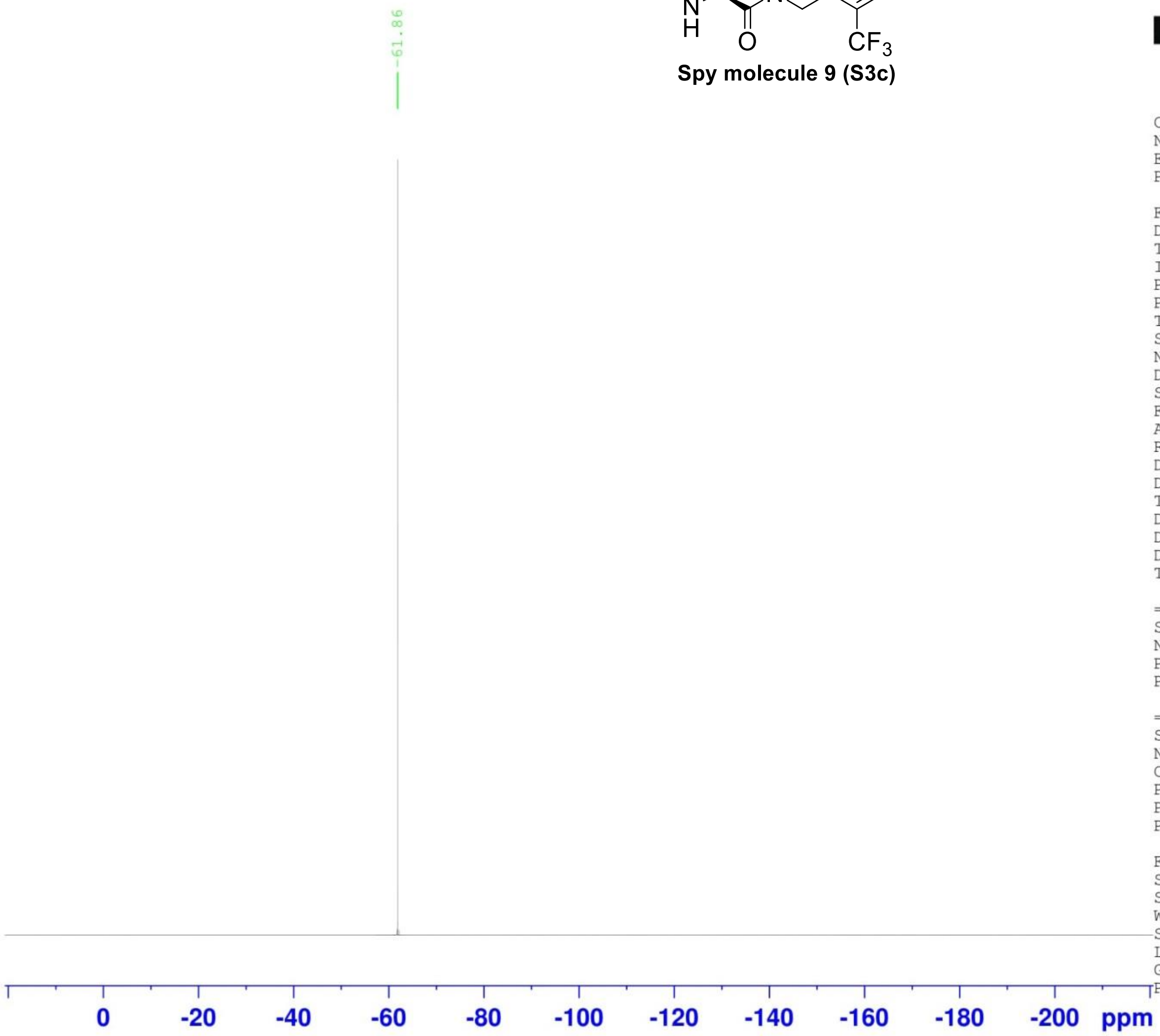

Current Data Parameters

NAME	GCF09
EXPNO	11
PROCNO	1

F2 - Acquisition Parameters

Date_	20181113
Time	22.37
INSTRUM	spect
PROBHD	5 mm CPQCI 1H/
PULPROG	zgfhigqn.2
TD	131072
SOLVENT	MeOD
NS	256
DS	4
SWH	113636.367 Hz
FIDRES	0.866977 Hz
AQ	0.5767168 sec
RG	190.78
DW	4.400 usec
DE	18.00 usec
TE	293.2 K
D1	1.00000000 sec
D11	0.03000000 sec
D12	0.00002000 sec
TD0	1

======== CHANNEL f1 ========

SFO1	470.5453180 MHz
NUC1	19F
P1	12.00 usec
PLW1	5.19999981 W

======== CHANNEL f2 ========

SFO2	500.1320005 MHz
NUC2	1H
CPDPRG[2	waltz16
PCPD2	100.00 usec
PLW2	5.19999981 W
PLW12	0.06406900 W

F2 - Processing parameters

SI	262144
SF	470.5923772 MHz
WDW	EM
SSB	0
LB	1.50 Hz
GB	0
PC	1.00

Spy molecule 13 (Compound S3d)

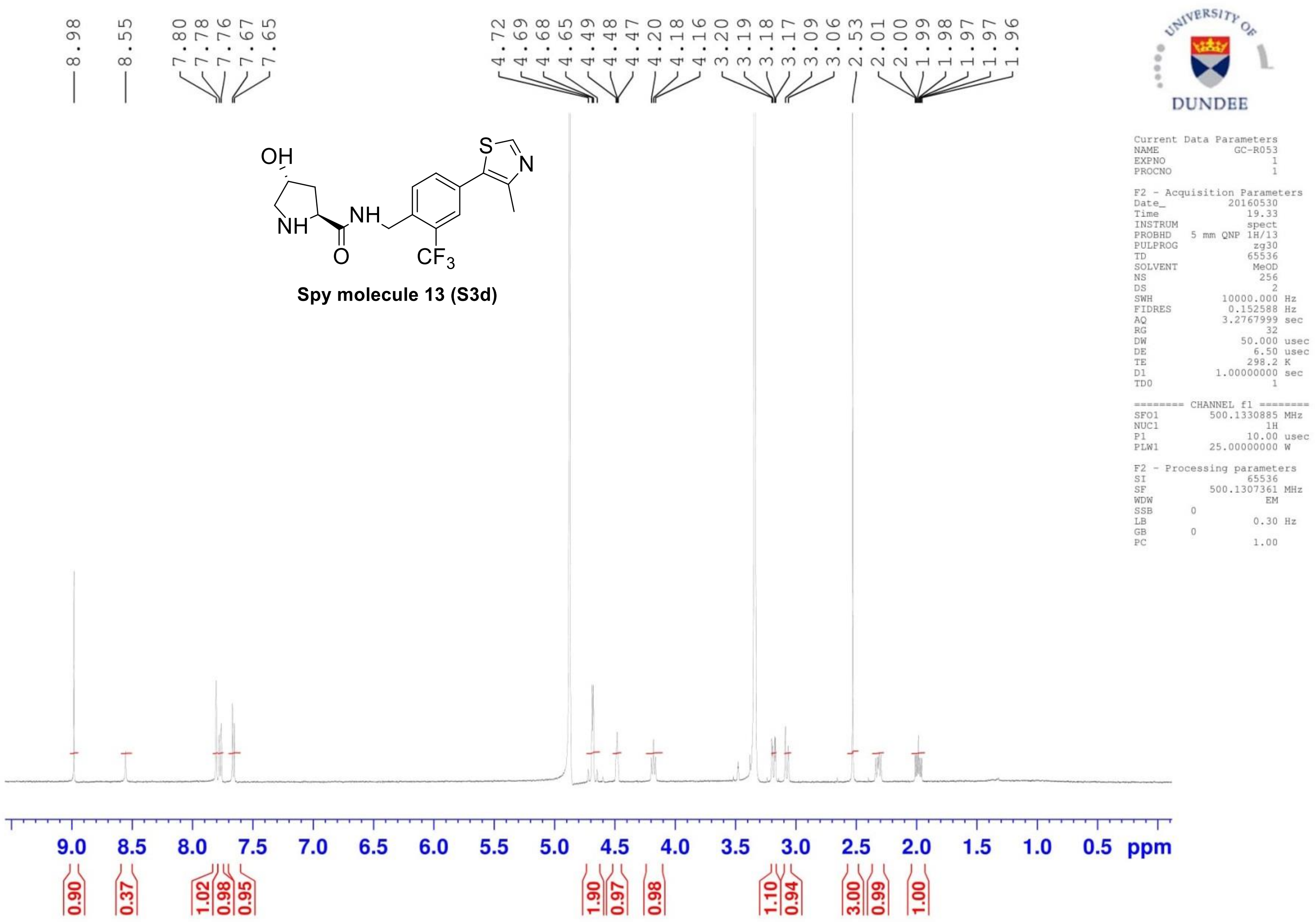

Spy molecule 13 (Compound S3d)

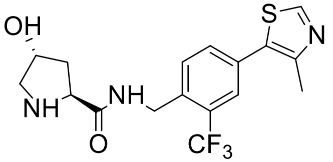

Spy molecule 13 (S3d)

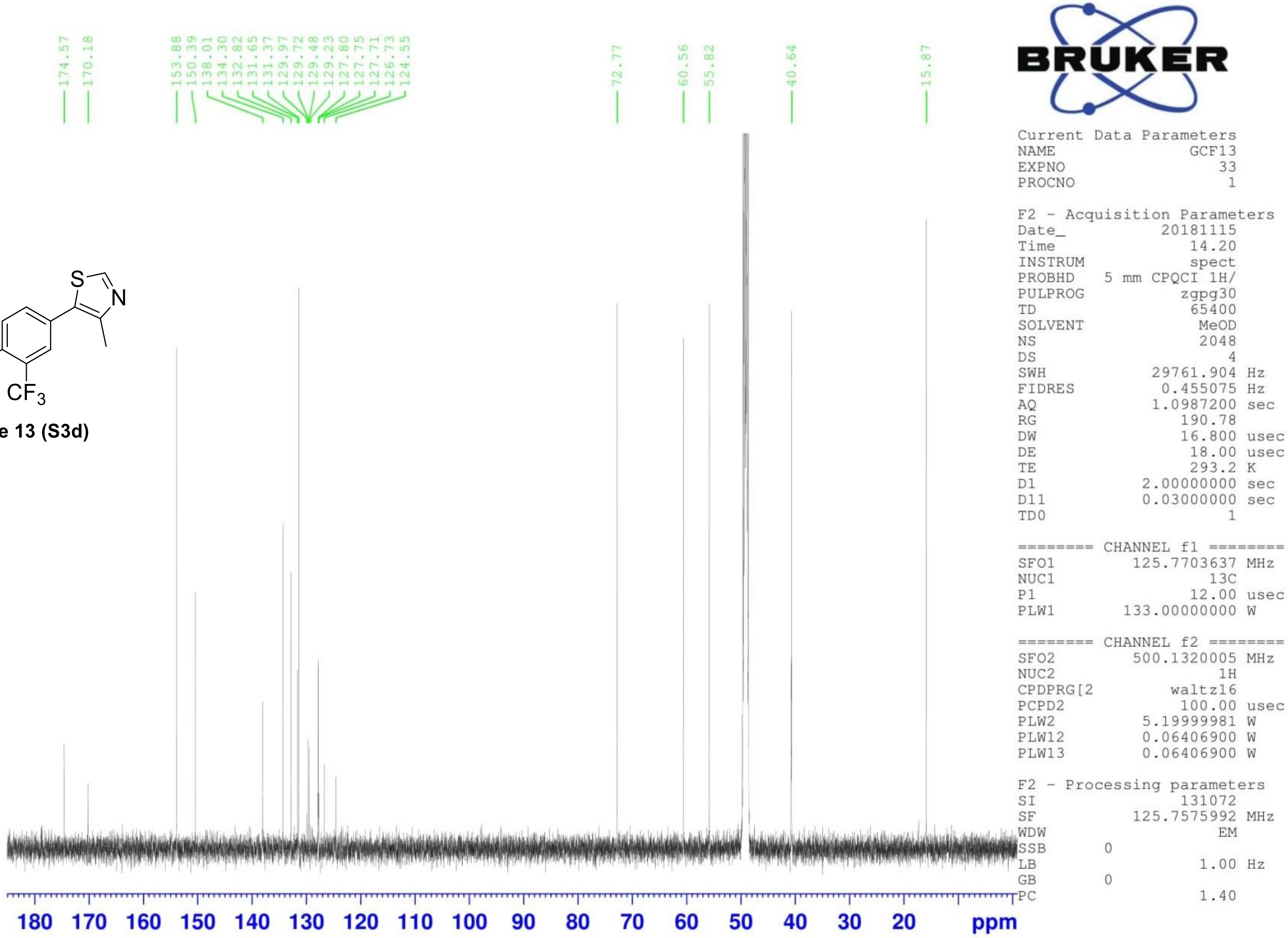

Spy molecule 13 (Compound S3d)

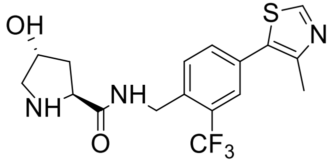

Spy molecule 13 (S3d)

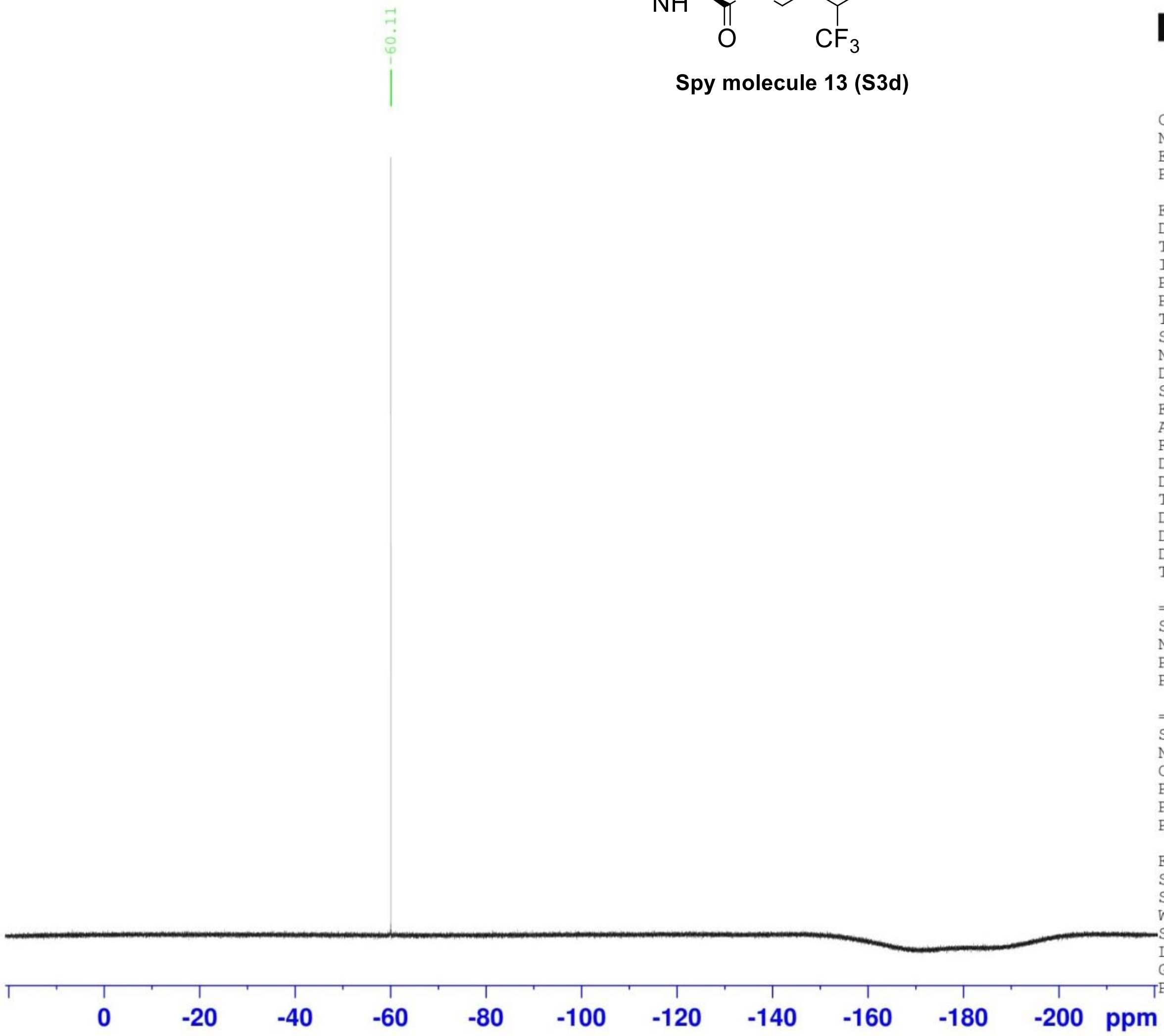


```
Current Data Parameters
NAME              GC-R053
EXPNO                   4
PROCNO                  1

F2 - Acquisition Parameters
Date_            20160530
Time                20.31
INSTRUM             spect
PROBHD     5 mm QNP 1H/13
PULPROG        zgfhigqn.2
TD                 131072
SOLVENT              MeOD
NS                     16
DS                      4
SWH            113636.367 Hz
FIDRES           0.866977 Hz
AQ              0.5767168 sec
RG                    362
DW                  4.400 usec
DE                   6.50 usec
TE                  298.2 K
D1             1.00000000 sec
D11            0.03000000 sec
D12            0.00002000 sec
TD0                     1

======== CHANNEL f1 ========
SFO1          470.5453180 MHz
NUC1                  19F
P1                  13.50 usec
PLW1          25.00000000 W

======== CHANNEL f2 ========
SFO2          500.1320005 MHz
NUC2                   1H
CPDPRG[2          waltz16
PCPD2               80.00 usec
PLW2          25.00000000 W
PLW12          0.39063001 W

F2 - Processing parameters
SI                  65536
SF            470.5923772 MHz
WDW                    EM
SSB      0
LB                   0.30 Hz
GB       0
PC                   1.00
```

Spy molecule 2 (Compound S4a)

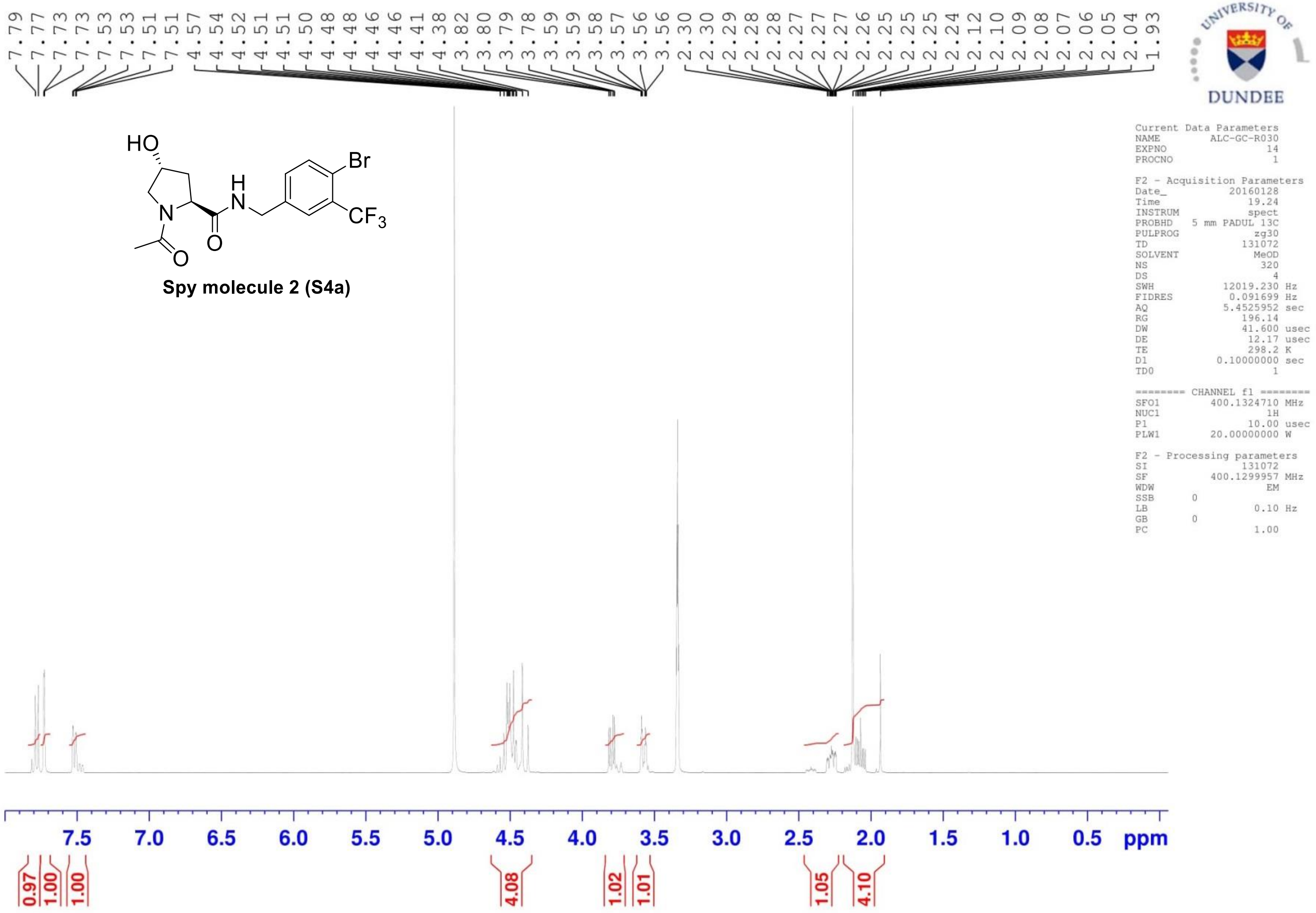

Spy molecule 2 (Compound S4a)

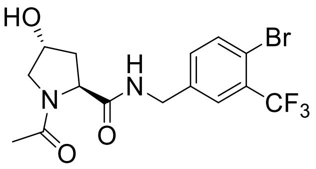

Spy molecule 2 (S4a)

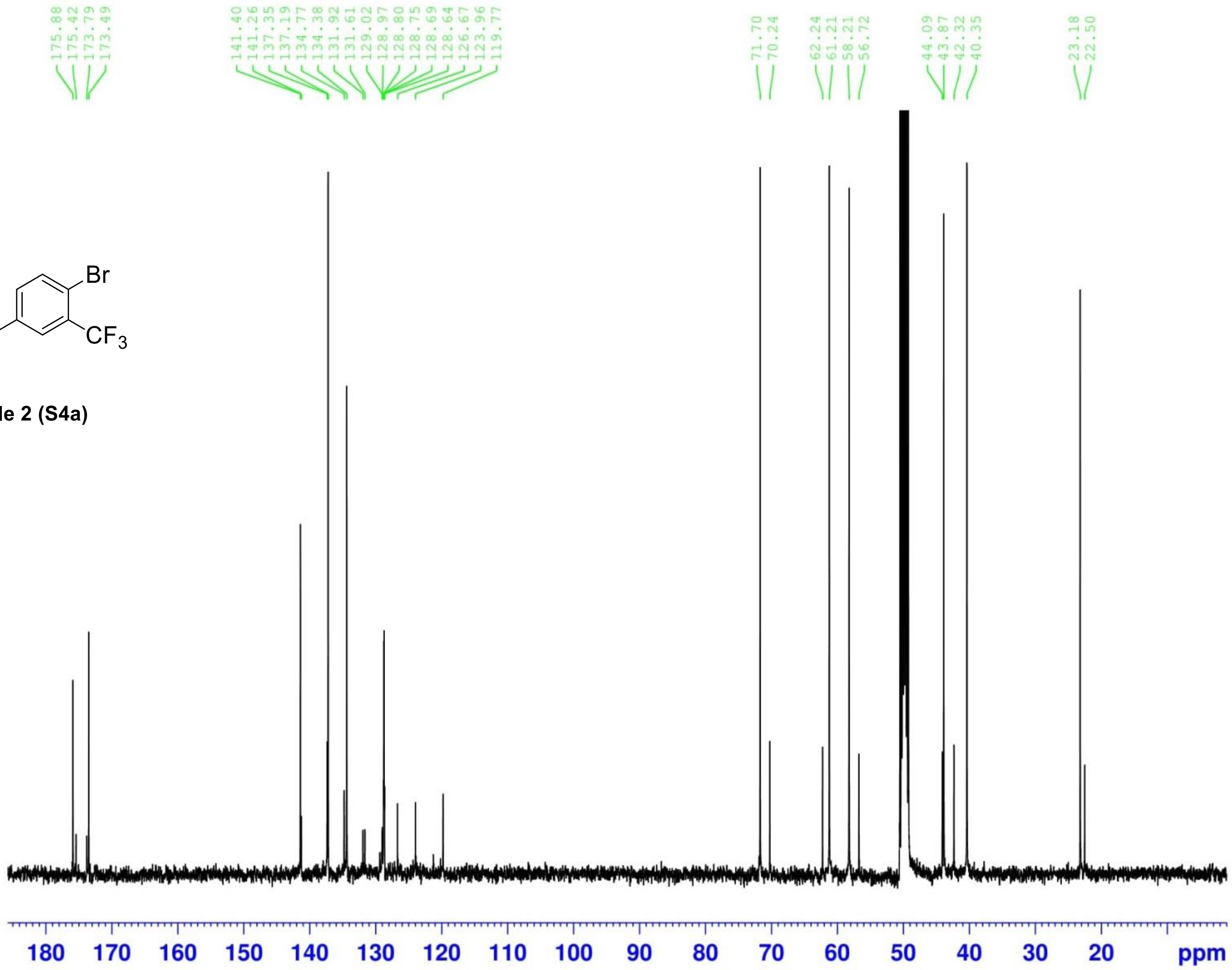

DUNDEE

```
Current Data Parameters
NAME          ALC-GC-R030
EXPNO                  16
PROCNO                  1

F2 - Acquisition Parameters
Date_            20160128
Time                22.27
INSTRUM             spect
PROBHD   5 mm PADUL 13C
PULPROG             udeft
TD                  17996
SOLVENT              MeOD
NS                   2800
DS                      0
SWH             25000.000 Hz
FIDRES           1.389198 Hz
AQ              0.3599200 sec
RG                 196.14
DW                 20.000 usec
DE                   8.66 usec
TE                  298.2 K
D1             3.00000000 sec
D11            0.03000000 sec
D12            0.00002000 sec
D20          200.00000000 sec
TD0                     1

======== CHANNEL f1 ========
SFO1          100.6238346 MHz
NUC1                  13C
P1                  10.00 usec
P13               2000.00 usec
P26                500.00 usec
PLW1          36.00000000 W
SPNAM[5]     Crp60comp.4
SPOAL5              0.500
SPOFFS5  0 Hz
SPW5           5.50040007 W
SPNAM[8] Crp60,0.5,20.1
SPOAL8              0.500
SPOFFS8  0 Hz
SPW8           5.50040007 W

======== CHANNEL f2 ========
SFO2          400.1316005 MHz
NUC2                   1H
CPDPRG[2          waltz64
PCPD2               90.00 usec
PLW2          20.00000000 W
PLW12          0.24691001 W

F2 - Processing parameters
SI                 262144
SF            100.6125410 MHz
WDW                    EM
SSB      0
LB                   2.00 Hz
GB       0
PC                   1.40
```

Spy molecule 2 (Compound S4a)

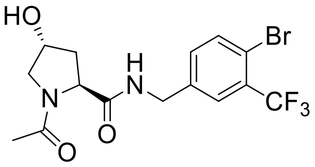

Spy molecule 2 (S4a)

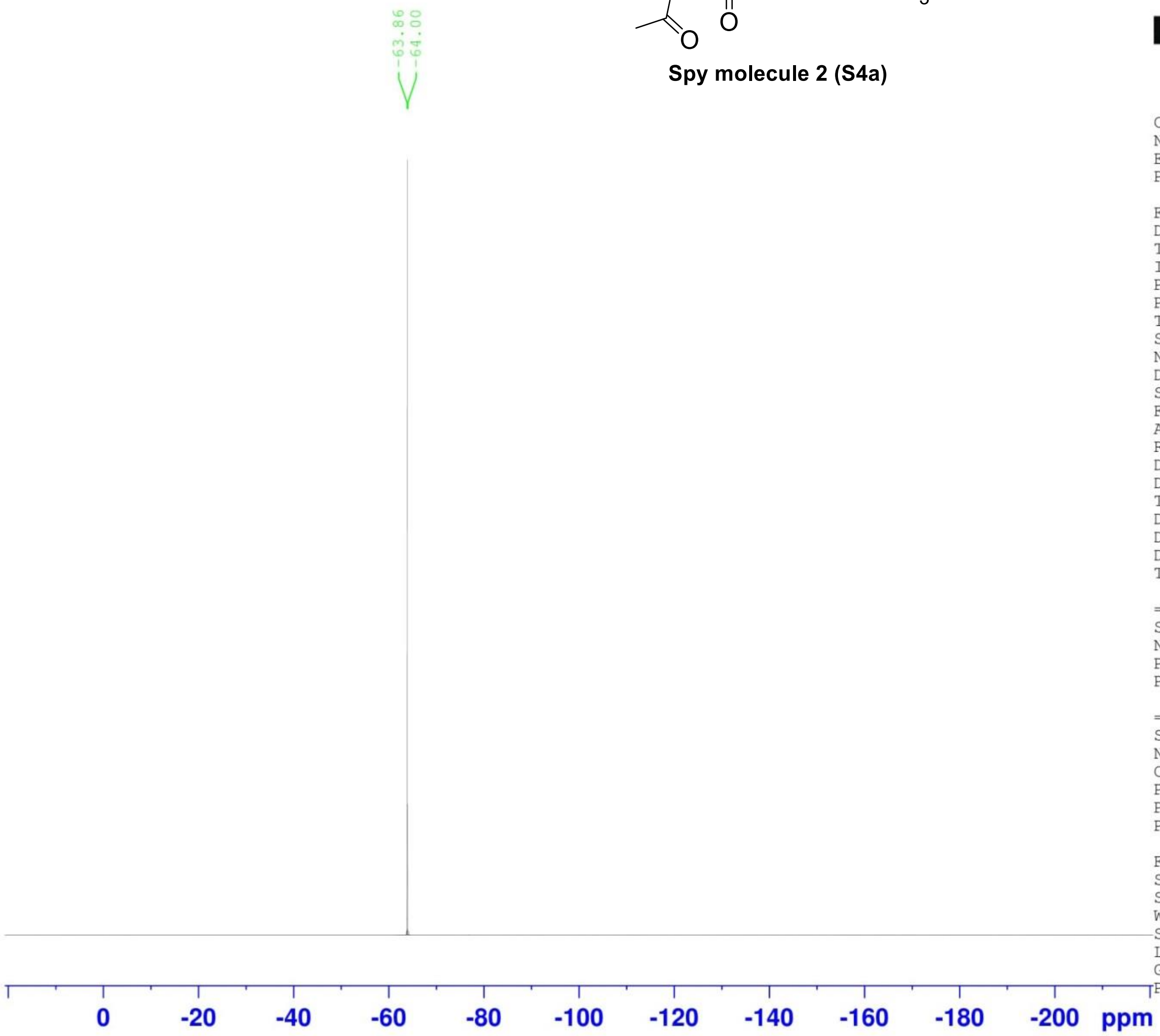


```
Current Data Parameters
NAME      GCF02 (Correct)
EXPNO                 100
PROCNO                  1

F2 - Acquisition Parameters
Date_            20181114
Time                10.35
INSTRUM             spect
PROBHD    5 mm CPQCI 1H/
PULPROG       zgfhigqn.2
TD                 131072
SOLVENT              MeOD
NS                    256
DS                      4
SWH            113636.367 Hz
FIDRES           0.866977 Hz
AQ              0.5767168 sec
RG                 190.78
DW                  4.400 usec
DE                  18.00 usec
TE                  293.1 K
D1             1.00000000 sec
D11            0.03000000 sec
D12            0.00002000 sec
TD0                     1

======== CHANNEL f1 ========
SFO1         470.5453180 MHz
NUC1                 19F
P1                 12.00 usec
PLW1          5.19999981 W

======== CHANNEL f2 ========
SFO2         500.1320005 MHz
NUC2                  1H
CPDPRG[2         waltz16
PCPD2             100.00 usec
PLW2          5.19999981 W
PLW12         0.06406900 W

F2 - Processing parameters
SI                262144
SF           470.5923772 MHz
WDW                   EM
SSB       0
LB                  1.50 Hz
GB        0
PC                  1.00
```

Spy molecule 6 (Compound S4b)

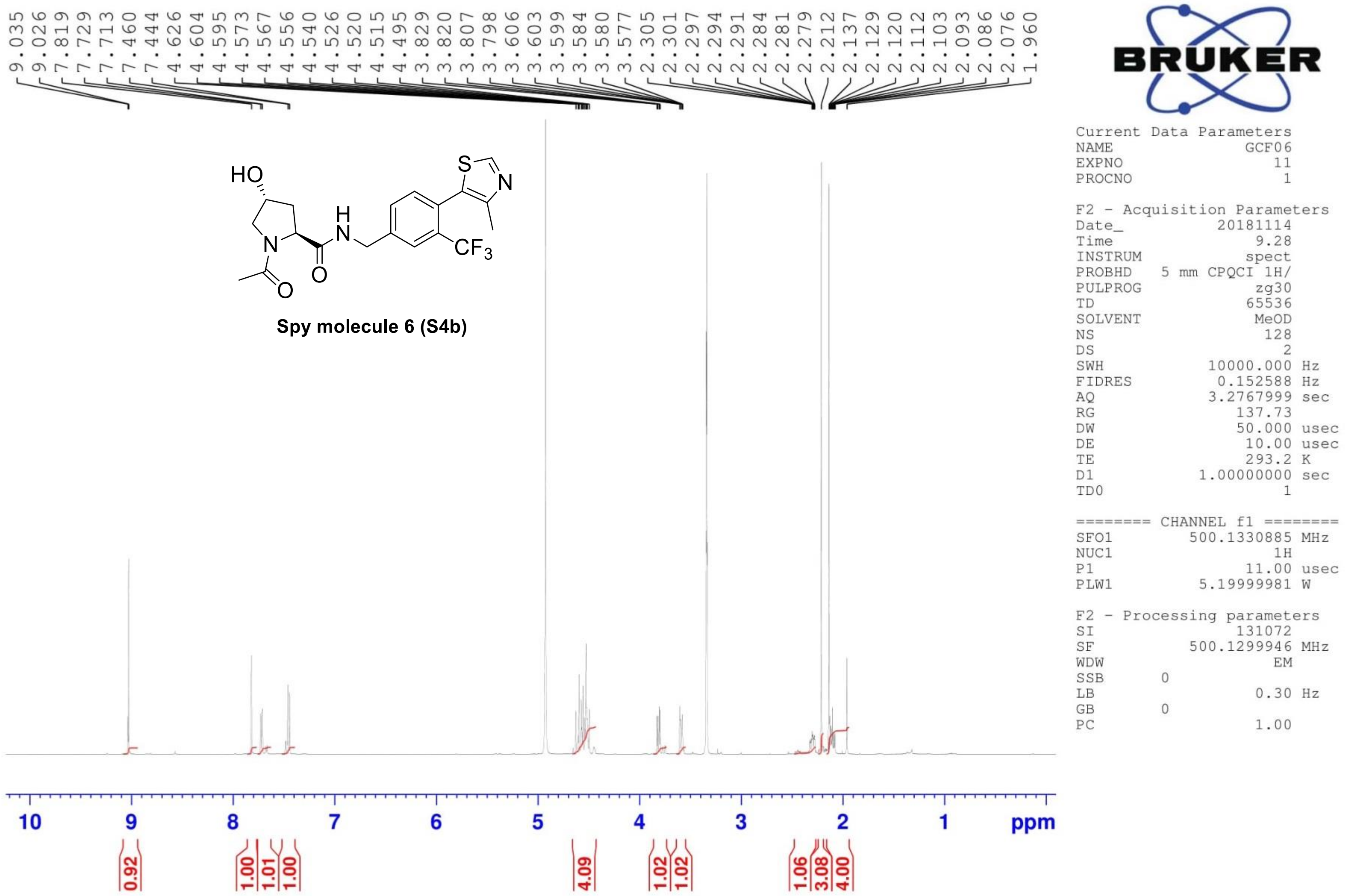

Spy molecule 6 (Compound S4b)

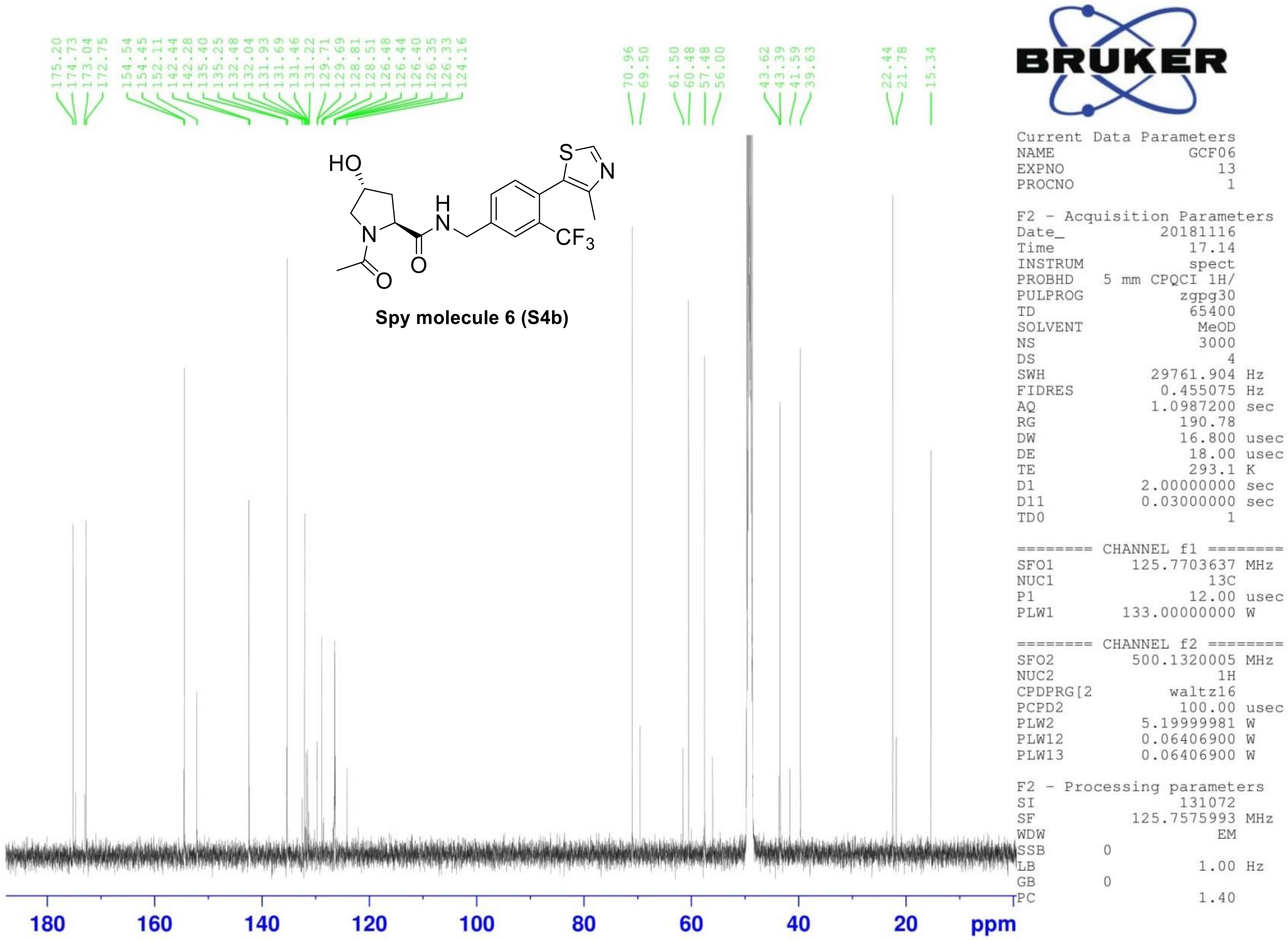

Spy molecule 6 (Compound S4b)

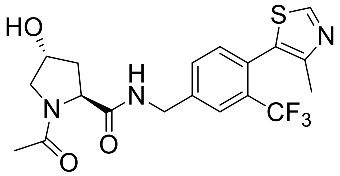

Spy molecule 6 (S4b)

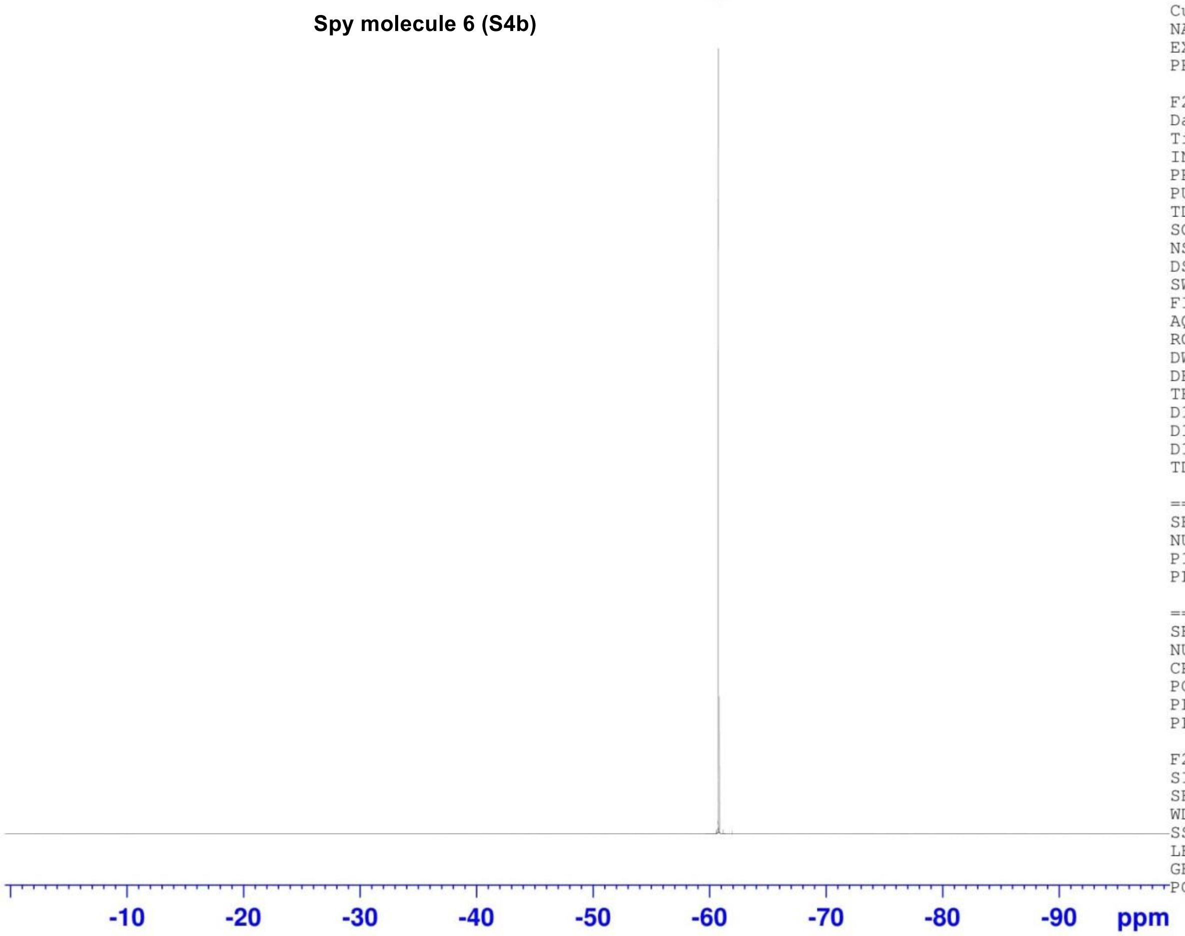


```
Current Data Parameters
NAME                GCF06
EXPNO                  10
PROCNO                  1

F2 - Acquisition Parameters
Date_            20181114
Time                 9.18
INSTRUM             spect
PROBHD    5 mm CPQCI 1H/
PULPROG        zgfhigqn.2
TD                 131072
SOLVENT              MeOD
NS                    256
DS                      4
SWH            113636.367 Hz
FIDRES           0.866977 Hz
AQ              0.5767168 sec
RG                 190.78
DW                  4.400 usec
DE                  18.00 usec
TE                  293.2 K
D1             1.00000000 sec
D11            0.03000000 sec
D12            0.00002000 sec
TD0                     1

======== CHANNEL f1 ========
SFO1          470.5453180 MHz
NUC1                  19F
P1                  12.00 usec
PLW1           5.19999981 W

======== CHANNEL f2 ========
SFO2          500.1320005 MHz
NUC2                   1H
CPDPRG[2          waltz16
PCPD2              100.00 usec
PLW2           5.19999981 W
PLW12          0.06406900 W

F2 - Processing parameters
SI                 262144
SF            470.5923772 MHz
WDW                    EM
SSB      0
LB                   1.50 Hz
GB       0
PC                   1.00
```

Spy molecule 10 (Compound S4c)

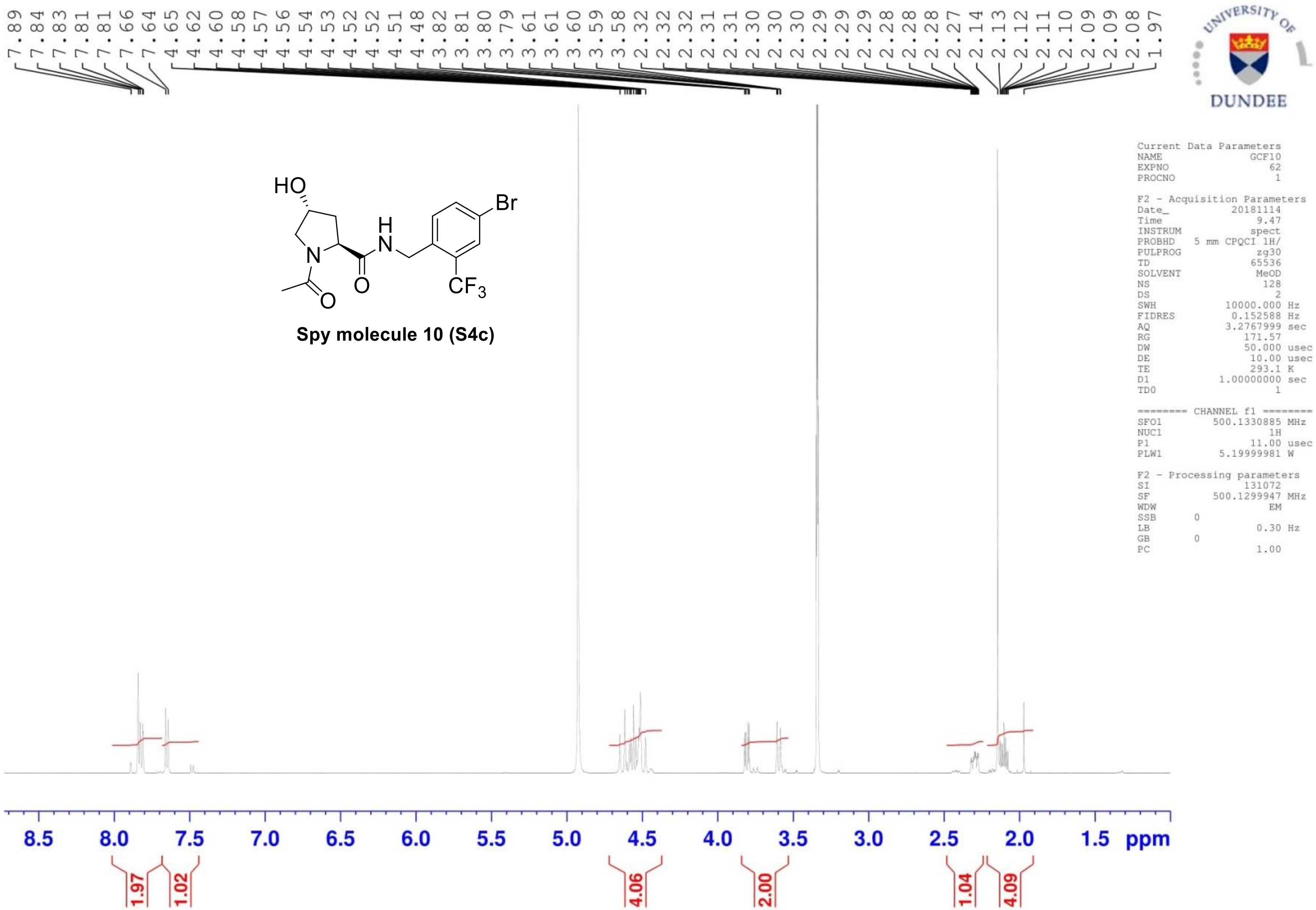

Spy molecule 10 (Compound S4c)

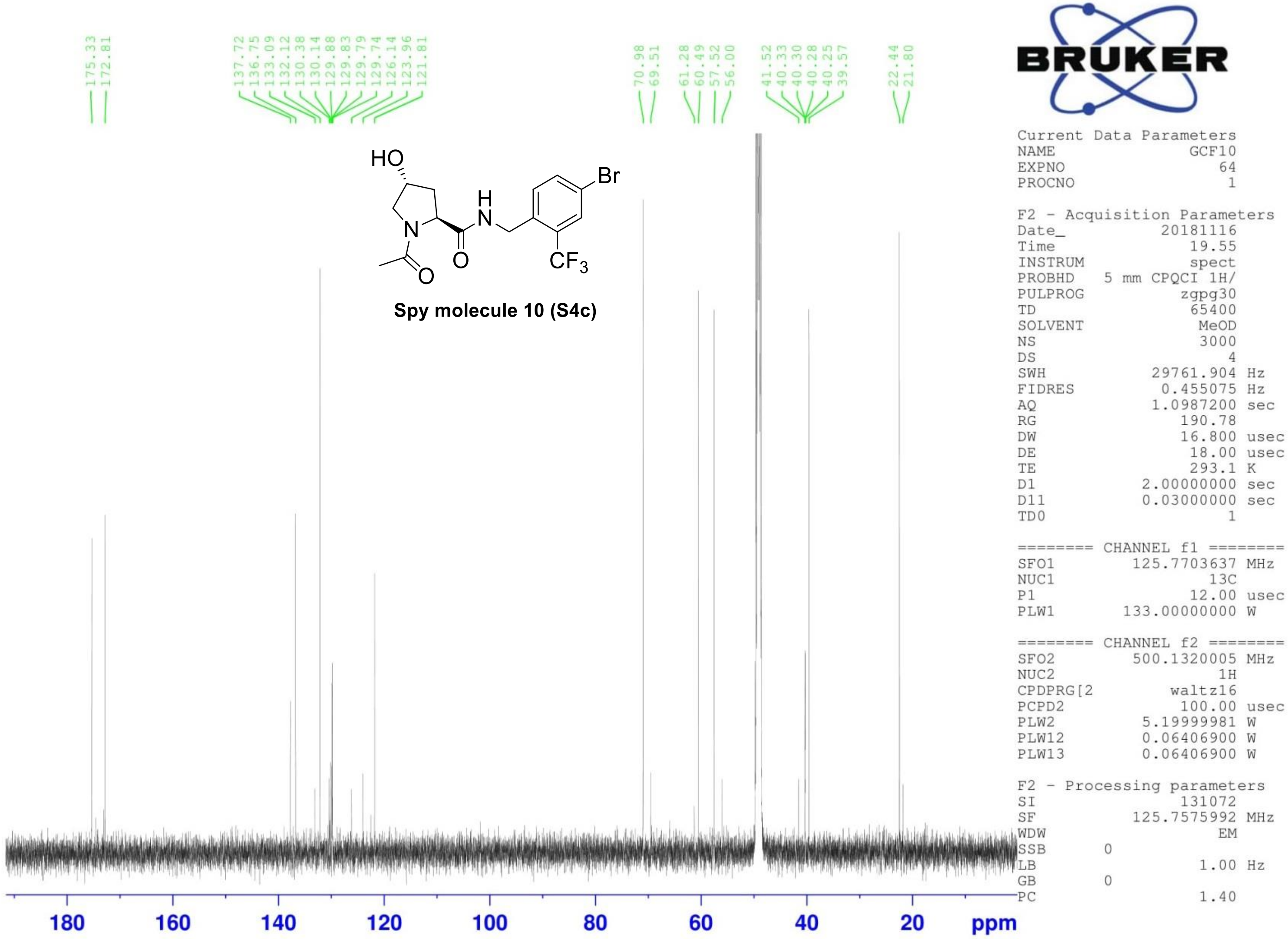

Spy molecule 10 (Compound S4c)

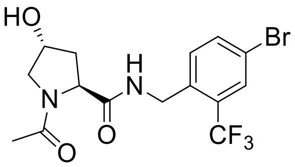

Spy molecule 10 (S4c)

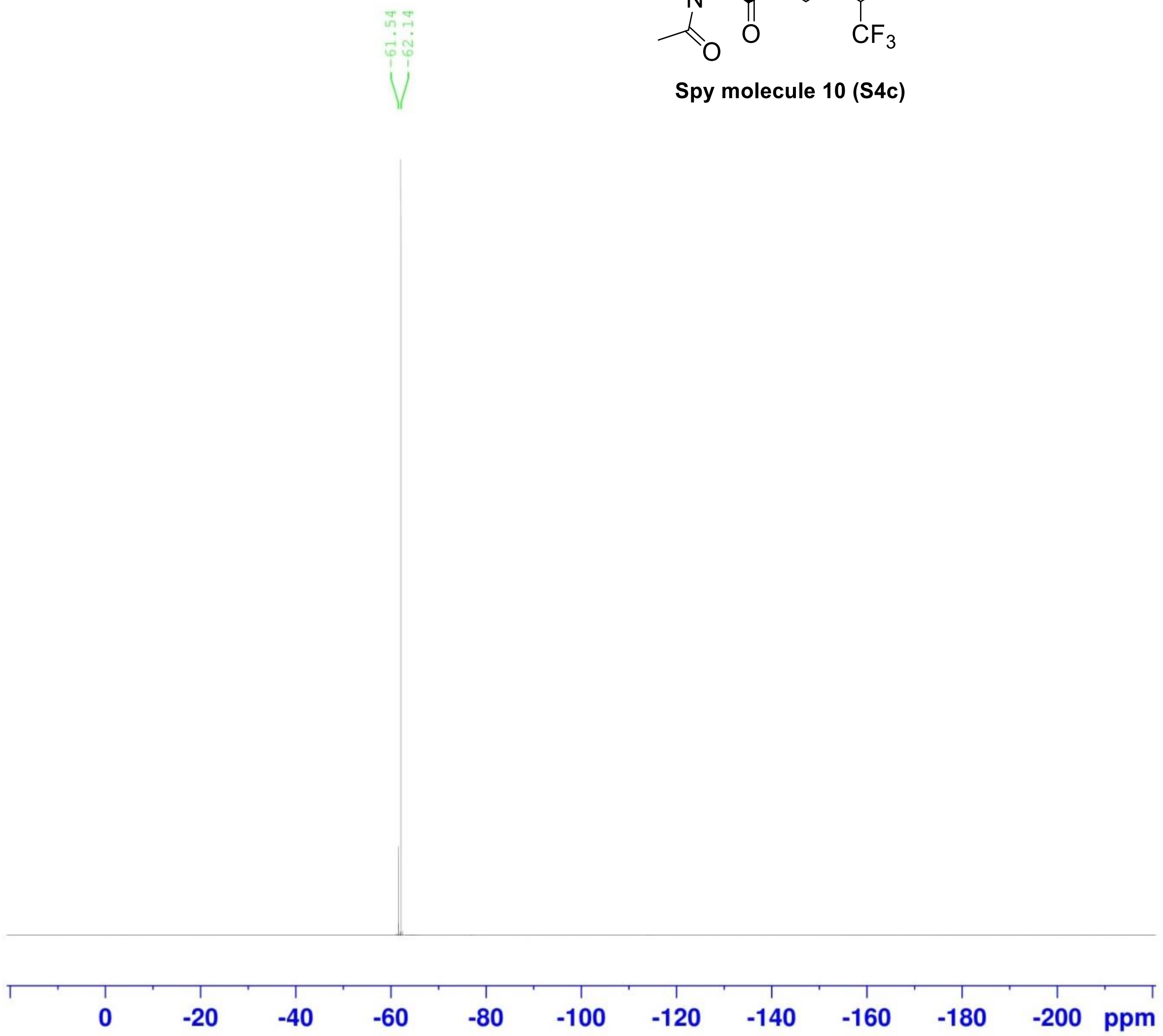


```
Current Data Parameters
NAME              GCF10
EXPNO                60
PROCNO                1

F2 - Acquisition Parameters
Date_          20181114
Time               9.37
INSTRUM           spect
PROBHD   5 mm CPQCI 1H/
PULPROG      zgfhigqn.2
TD               131072
SOLVENT            MeOD
NS                  256
DS                    4
SWH          113636.367 Hz
FIDRES         0.866977 Hz
AQ            0.5767168 sec
RG               190.78
DW                4.400 usec
DE                18.00 usec
TE                293.1 K
D1           1.00000000 sec
D11          0.03000000 sec
D12          0.00002000 sec
TD0                   1

======== CHANNEL f1 ========
SFO1        470.5453180 MHz
NUC1                19F
P1                12.00 usec
PLW1         5.19999981 W

======== CHANNEL f2 ========
SFO2        500.1320005 MHz
NUC2                 1H
CPDPRG[2        waltz16
PCPD2            100.00 usec
PLW2         5.19999981 W
PLW12        0.06406900 W

F2 - Processing parameters
SI               262144
SF          470.5923772 MHz
WDW                  EM
SSB      0
LB                 1.50 Hz
GB       0
PC                 1.00
```

Spy molecule 14 (Compound S4d)

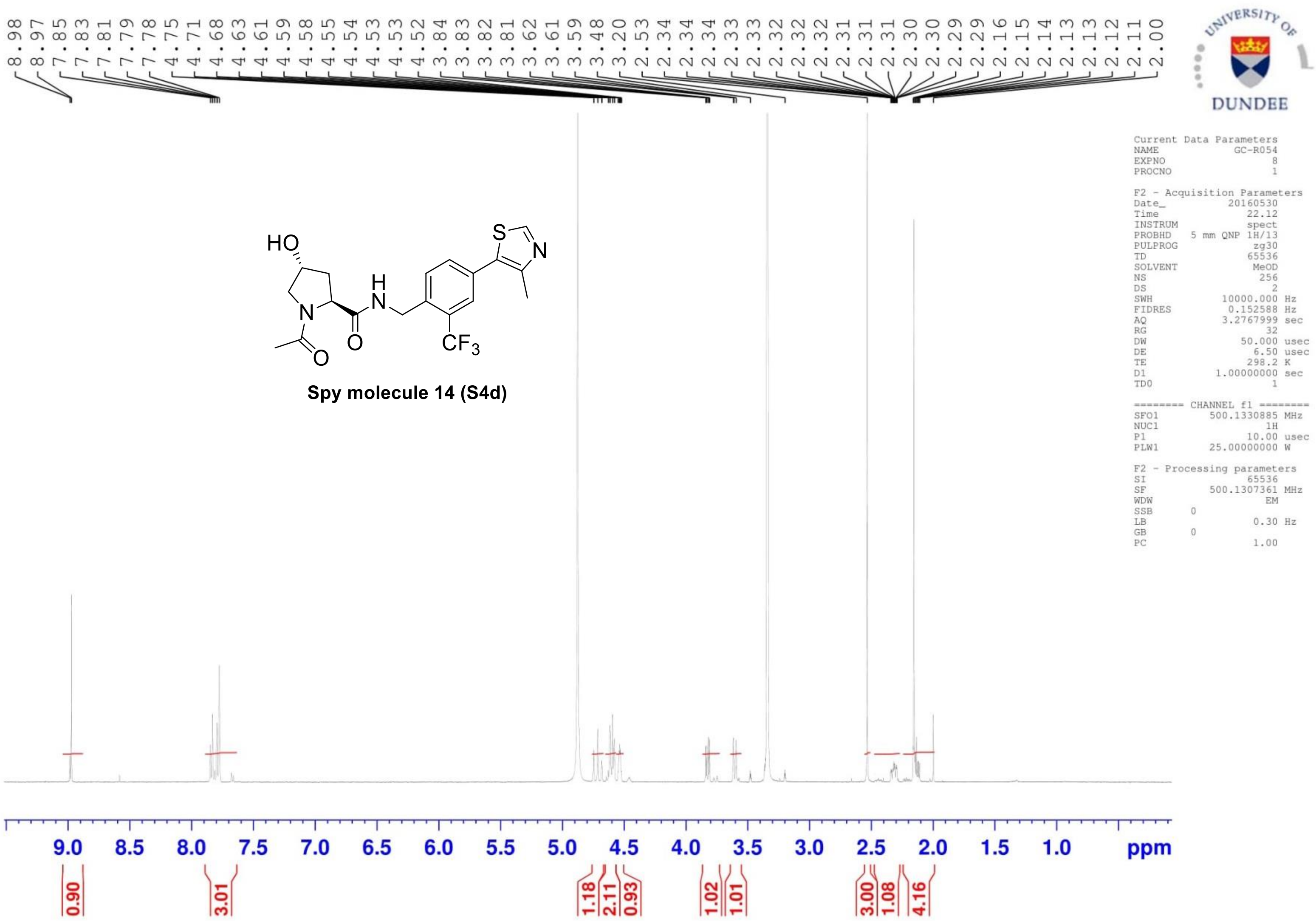

Spy molecule 14 (Compound S4d)

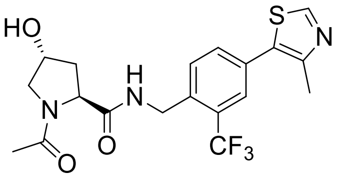

Spy molecule 14 (S4d)

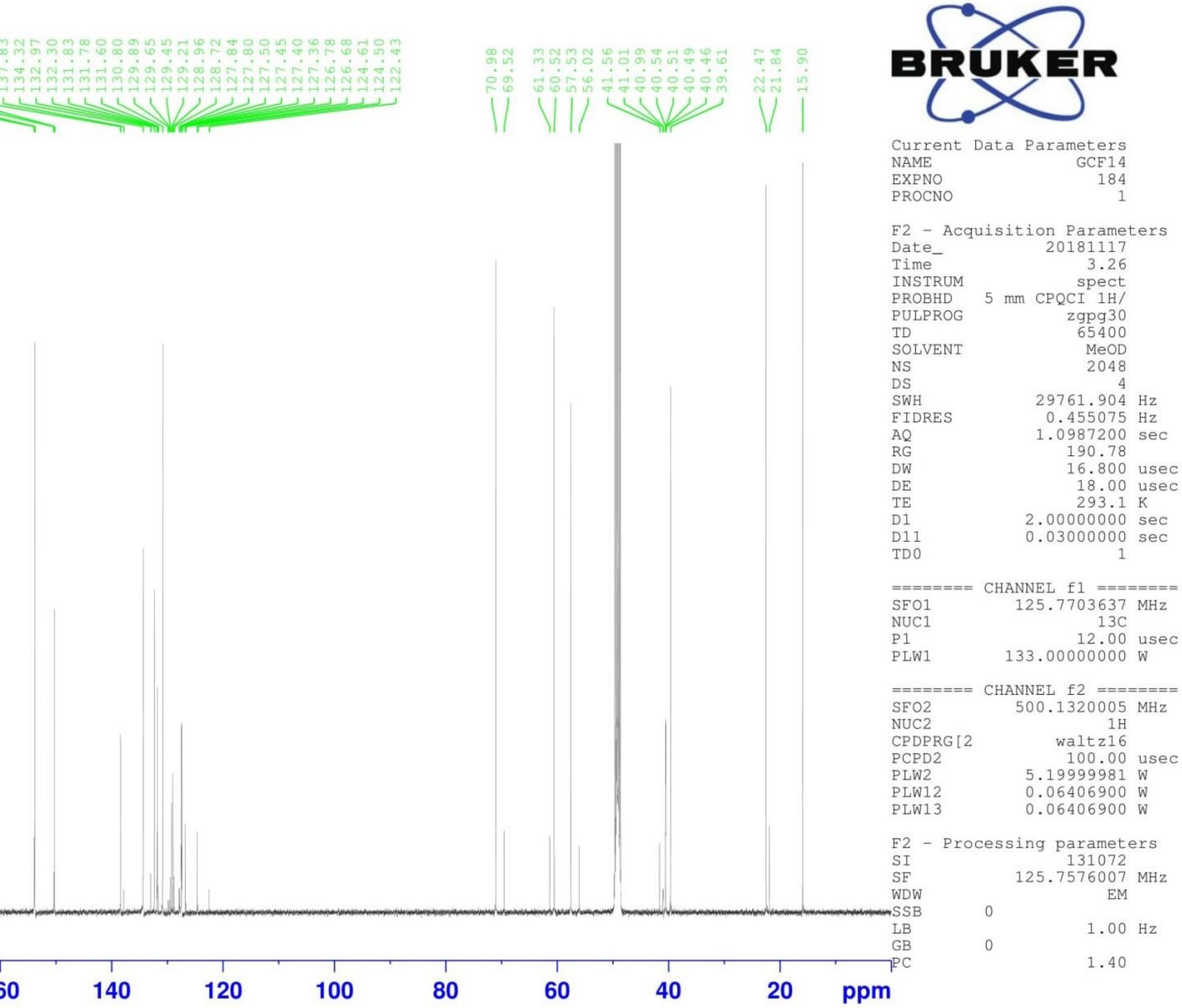

Spy molecule 14 (Compound S4d)

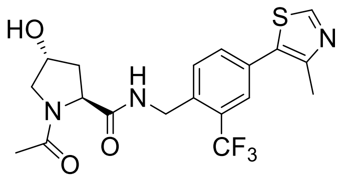

Spy molecule 14 (S4d)

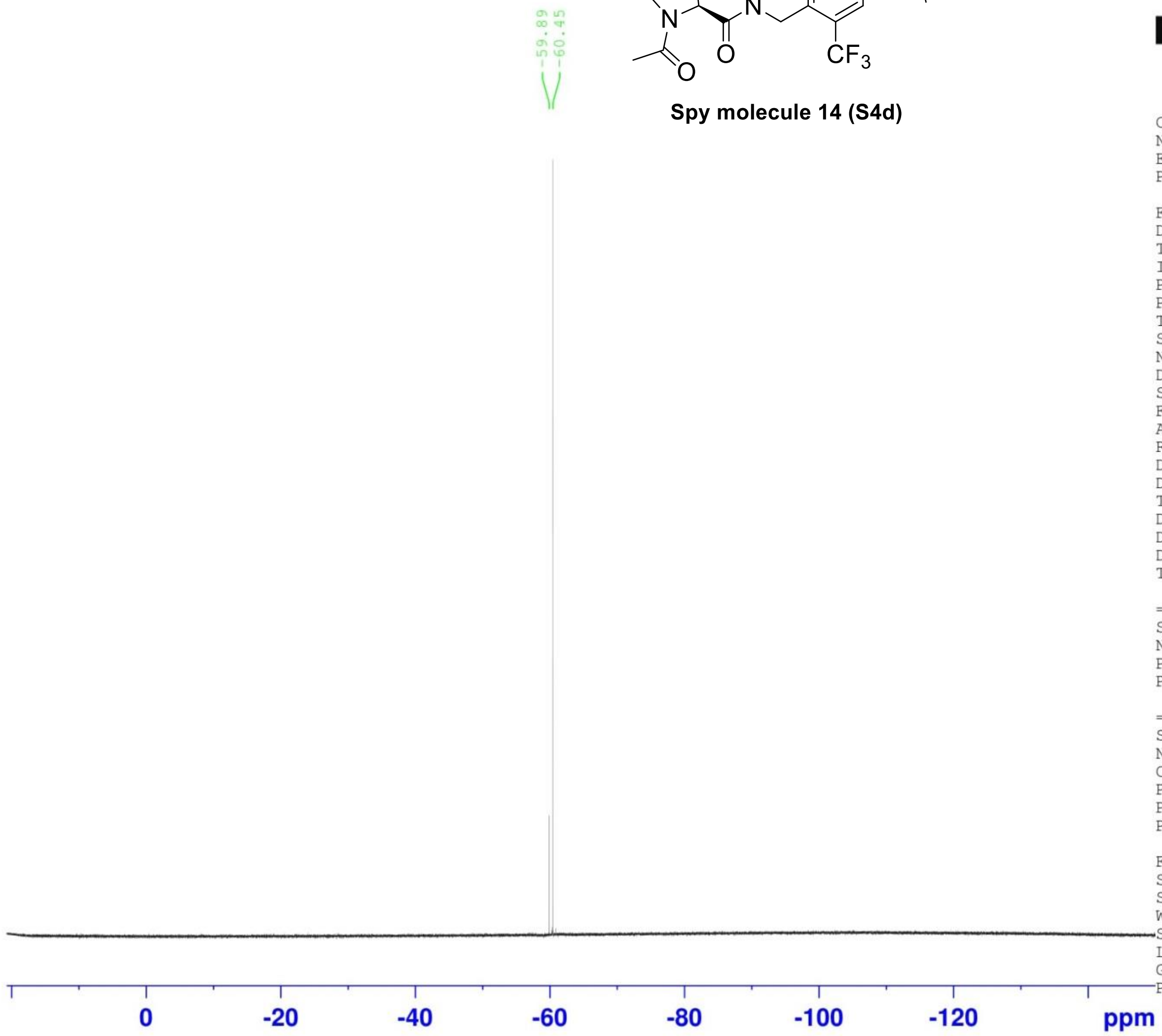


```
Current Data Parameters
NAME            GC-R054
EXPNO                12
PROCNO                1

F2 - Acquisition Parameters
Date_          20160530
Time              23.11
INSTRUM           spect
PROBHD   5 mm QNP 1H/13
PULPROG      zgfhigqn.2
TD               131072
SOLVENT            MeOD
NS                   16
DS                    4
SWH          113636.367 Hz
FIDRES         0.866977 Hz
AQ            0.5767168 sec
RG                  362
DW                4.400 usec
DE                 6.50 usec
TE                298.2 K
D1           1.00000000 sec
D11          0.03000000 sec
D12          0.00002000 sec
TD0                   1

======== CHANNEL f1 ========
SFO1        470.5453180 MHz
NUC1                19F
P1                13.50 usec
PLW1        25.00000000 W

======== CHANNEL f2 ========
SFO2        500.1320005 MHz
NUC2                 1H
CPDPRG[2        waltz16
PCPD2             80.00 usec
PLW2        25.00000000 W
PLW12        0.39063001 W

F2 - Processing parameters
SI                65536
SF          470.5923772 MHz
WDW                  EM
SSB       0
LB                 0.30 Hz
GB        0
PC                 1.00
```

Spy molecule 3 (Compound S5a)

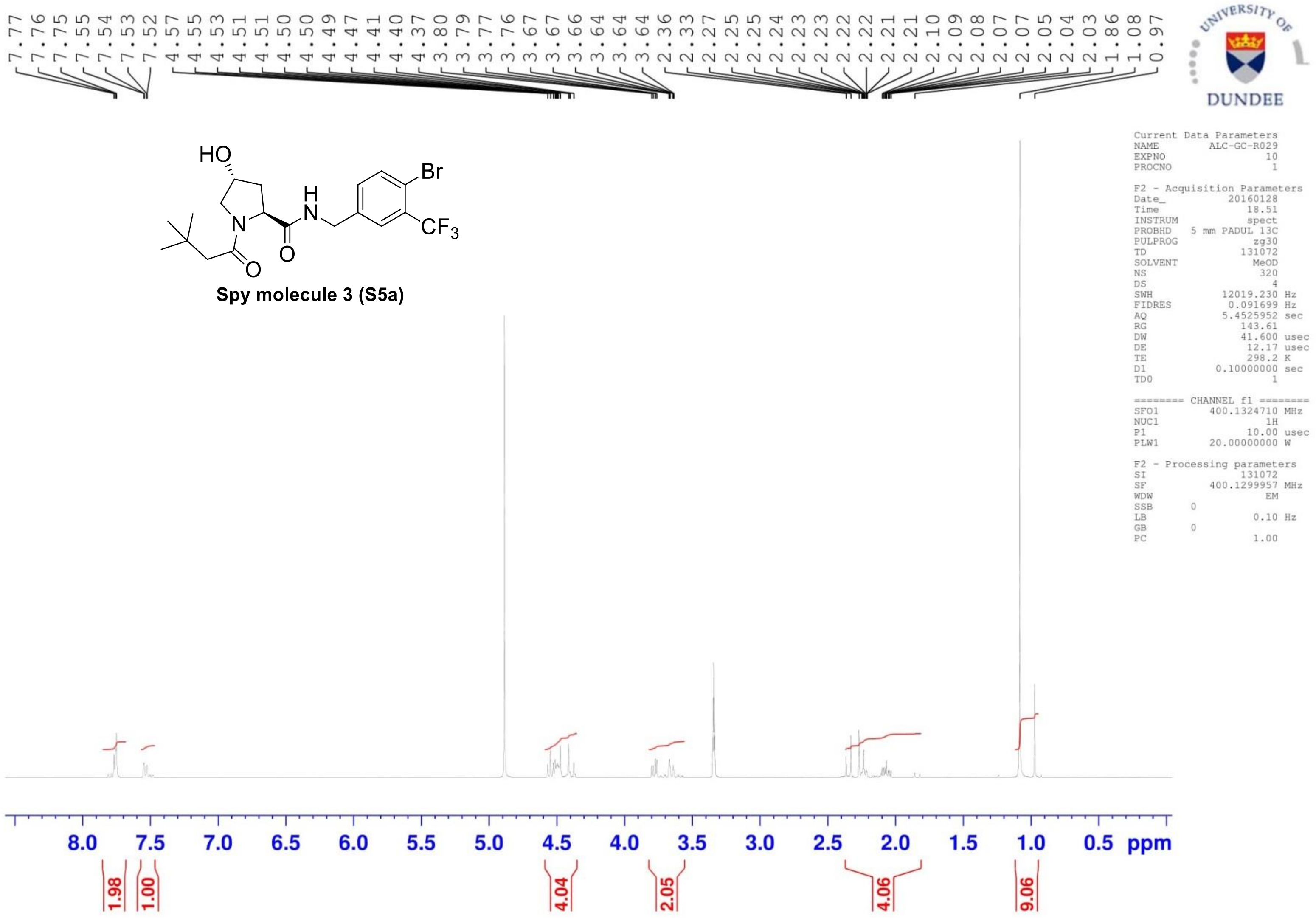

Spy molecule 3 (Compound S5a)

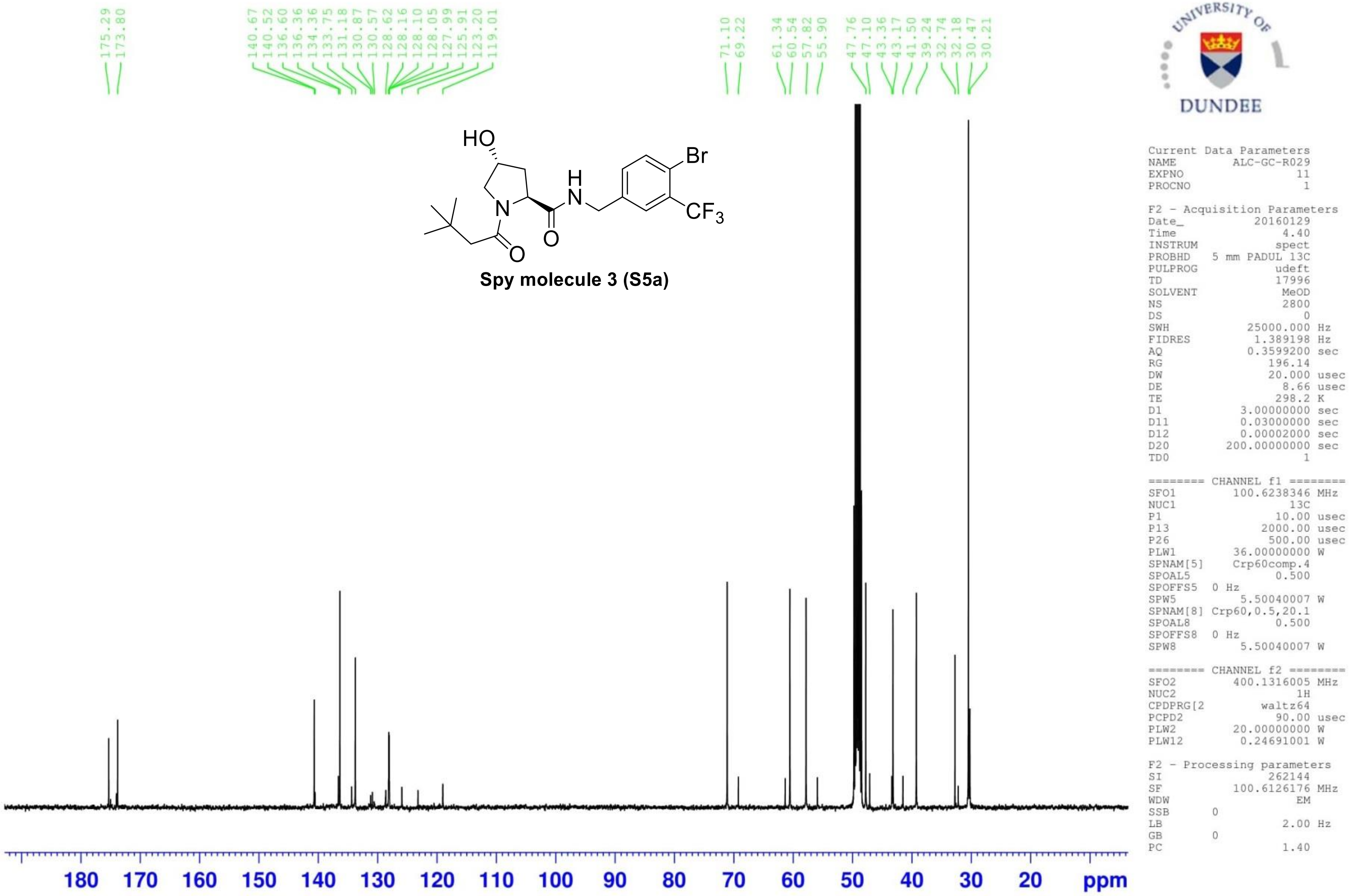

Spy molecule 3 (Compound S5a)

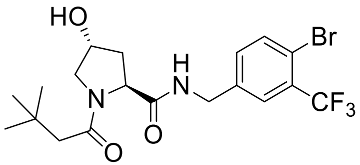

Spy molecule 3 (S5a)

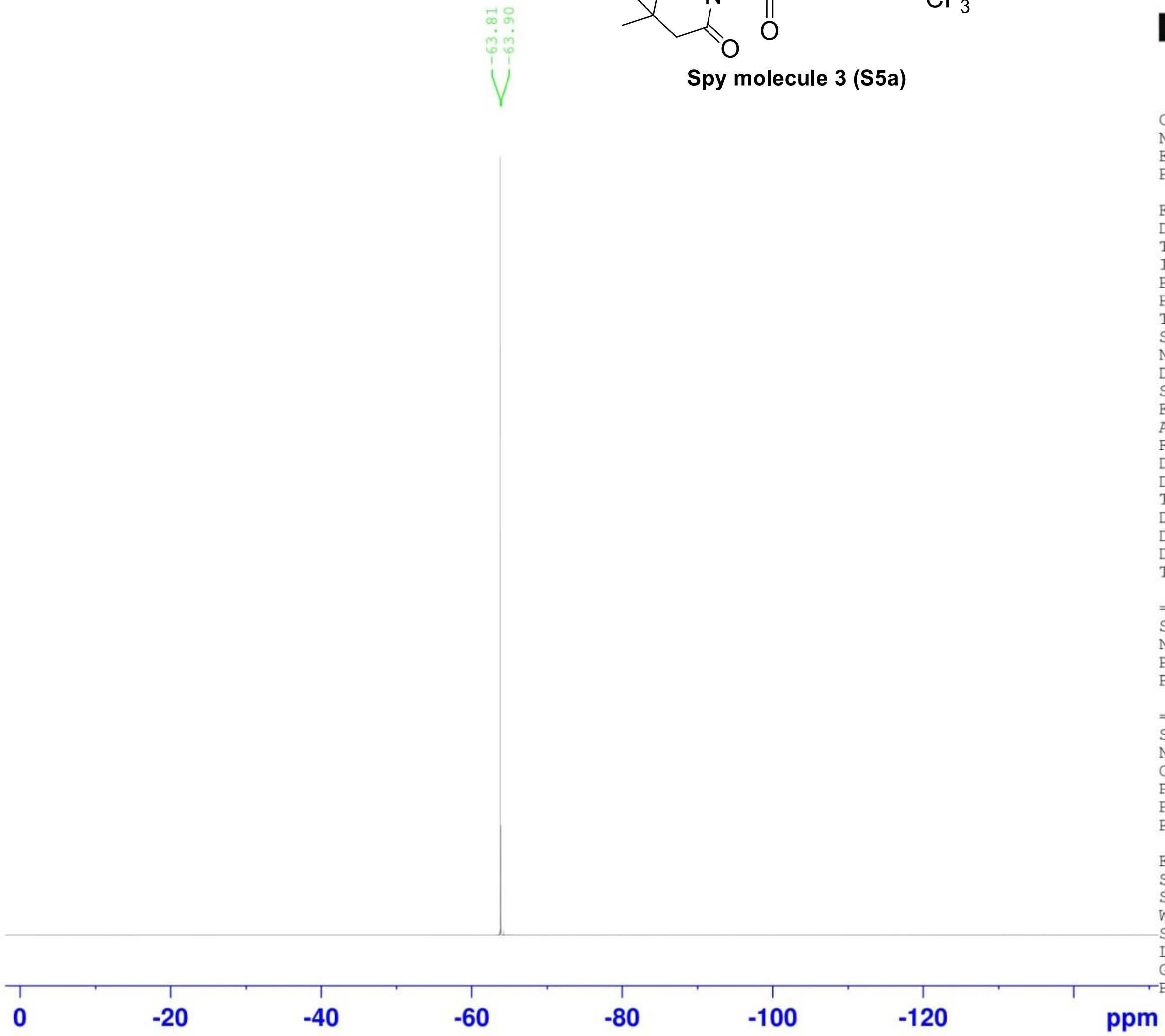

Current Data Parameters
NAME GCF03 (Correct)
EXPNO 10
PROCNO 1

F2 - Acquisition Parameters
Date_ 20181114
Time 8.58
INSTRUM spect
PROBHD 5 mm CPQCI 1H/
PULPROG zgfhigqn.2
TD 131072
SOLVENT MeOD
NS 256
DS 4
SWH 113636.367 Hz
FIDRES 0.866977 Hz
AQ 0.5767168 sec
RG 190.78
DW 4.400 usec
DE 18.00 usec
TE 293.2 K
D1 1.00000000 sec
D11 0.03000000 sec
D12 0.00002000 sec
TD0 1

======== CHANNEL f1 ========
SFO1 470.5453180 MHz
NUC1 19F
P1 12.00 usec
PLW1 5.19999981 W

======== CHANNEL f2 ========
SFO2 500.1320005 MHz
NUC2 1H
CPDPRG[2 waltz16
PCPD2 100.00 usec
PLW2 5.19999981 W
PLW12 0.06406900 W

F2 - Processing parameters
SI 262144
SF 470.5923772 MHz
WDW EM
SSB 0
LB 1.50 Hz
GB 0
PC 1.00

Spy molecule 7 (Compound S5b)

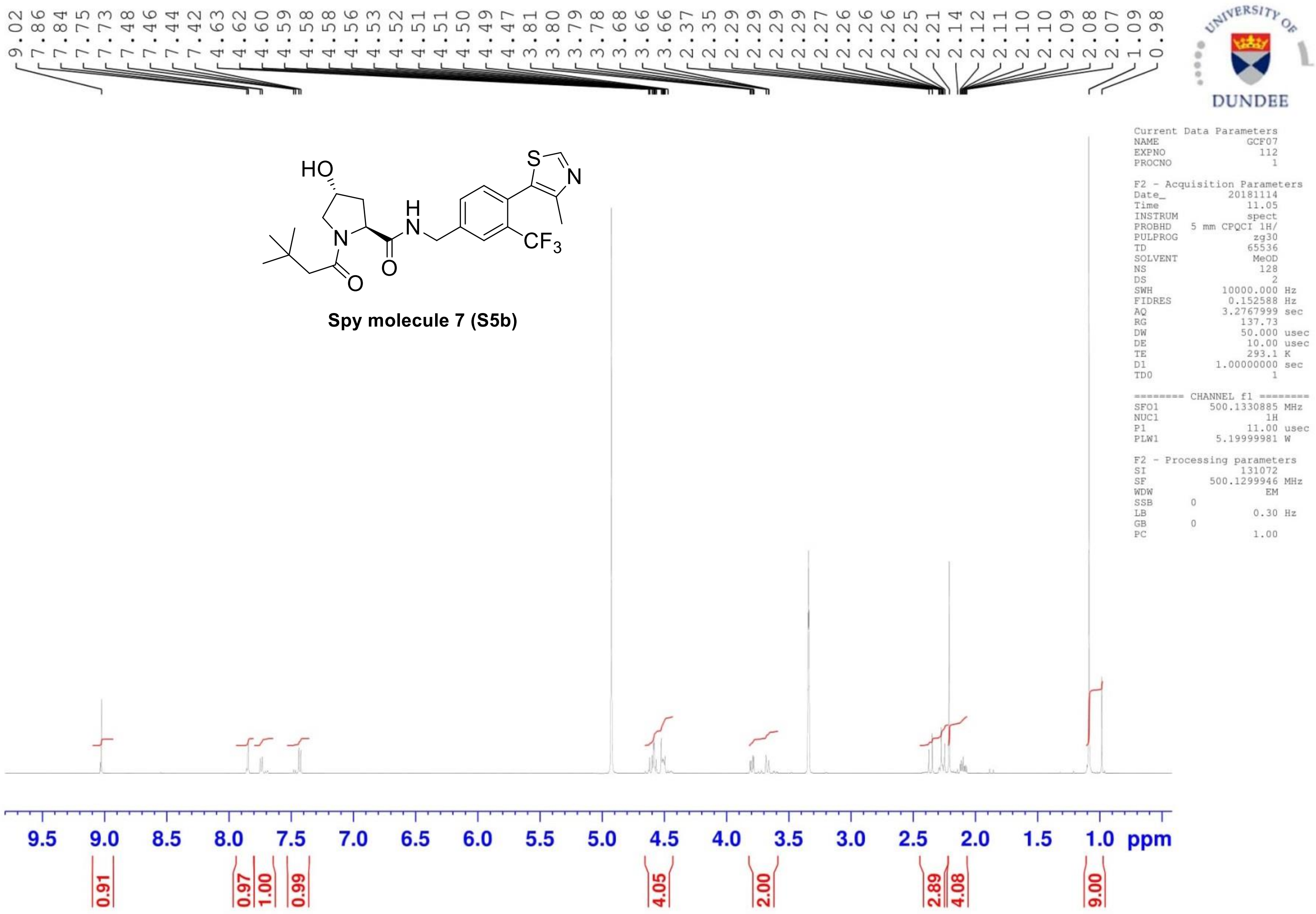

Spy molecule 7 (Compound S5b)

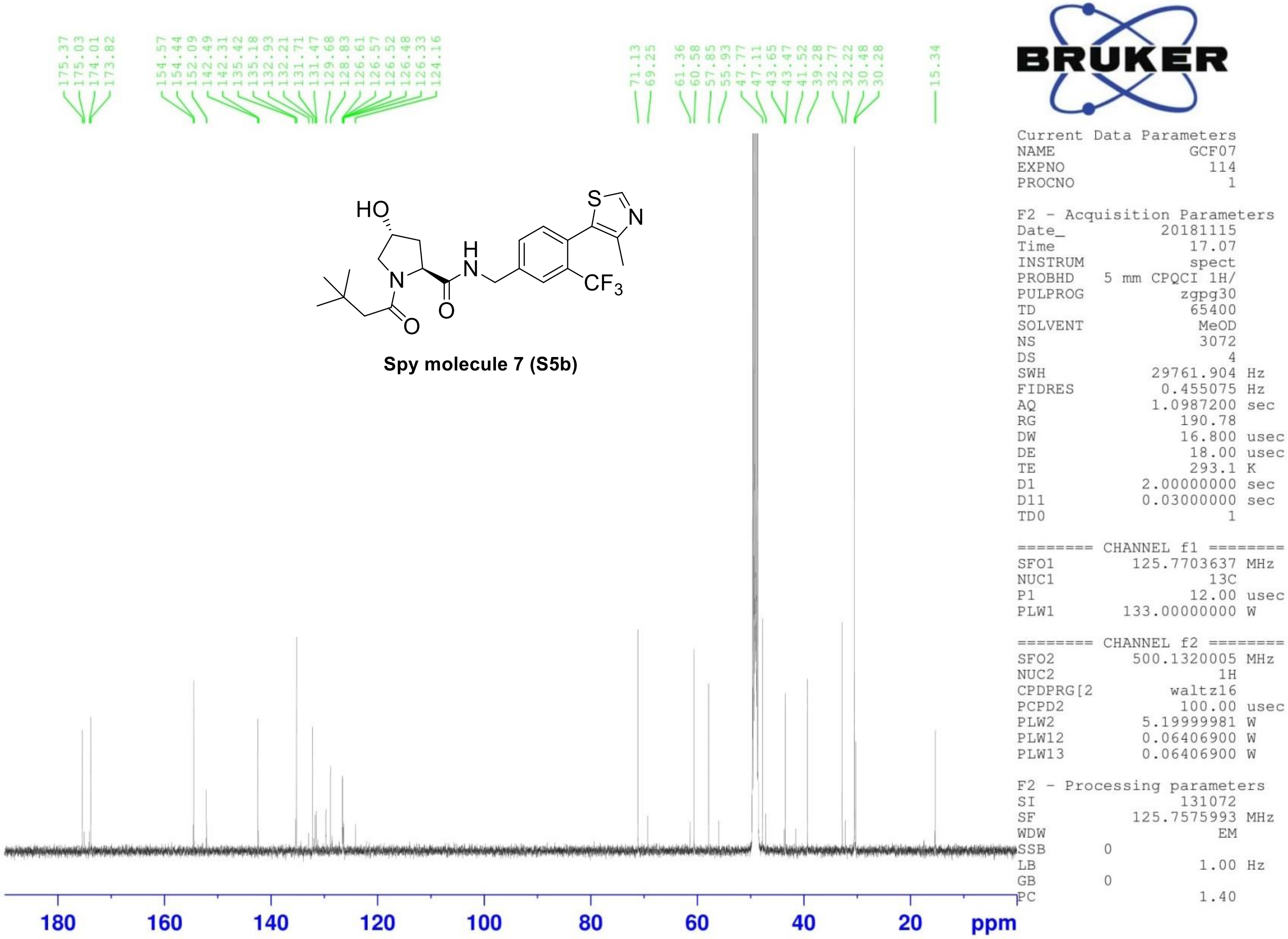

Spy molecule 7 (Compound S5b)

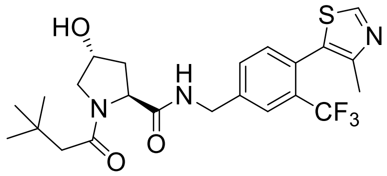

Spy molecule 7 (S5b)

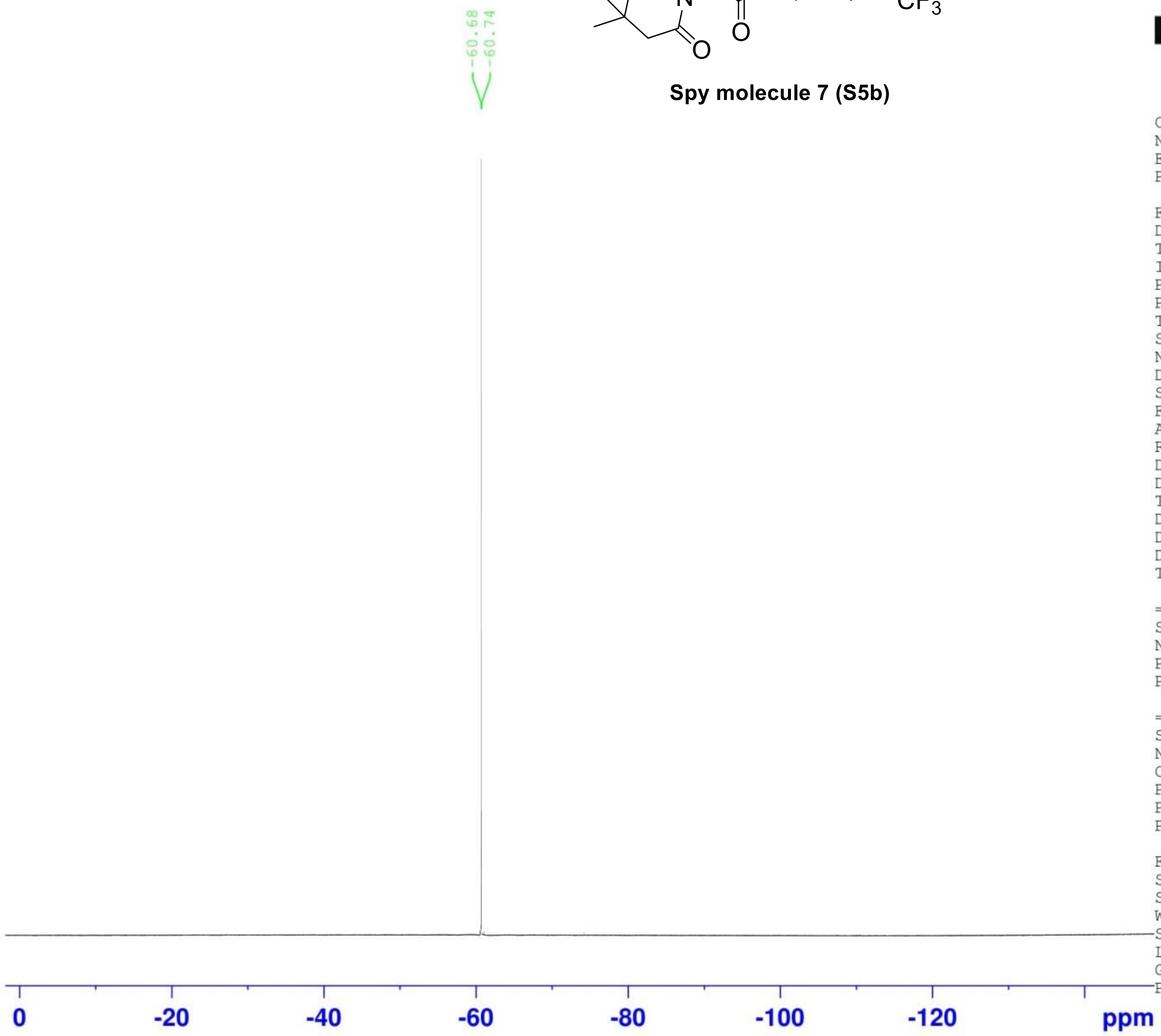


```
Current Data Parameters
NAME            GC-GCF007
EXPNO                  10
PROCNO                  1

F2 - Acquisition Parameters
Date_            20181122
Time                15.25
INSTRUM             spect
PROBHD    5 mm PABBO BB/
PULPROG       zgfhigqn.2
TD                 131072
SOLVENT              MeOD
NS                     16
DS                      4
SWH            113636.367 Hz
FIDRES           0.866977 Hz
AQ              0.5767168 sec
RG                    322
DW                  4.400 usec
DE                   6.50 usec
TE                  303.4 K
D1             1.00000000 sec
D11            0.03000000 sec
D12            0.00002000 sec
TD0                     1

======== CHANNEL f1 ========
SFO1          470.5453180 MHz
NUC1                  19F
P1                  14.50 usec
PLW1          45.00000000 W

======== CHANNEL f2 ========
SFO2          500.1320005 MHz
NUC2                   1H
CPDPRG[2          waltz16
PCPD2               80.00 usec
PLW2          20.85000038 W
PLW12          0.35916999 W

F2 - Processing parameters
SI                  65536
SF            470.5923772 MHz
WDW                    EM
SSB       0
LB                   0.30 Hz
GB        0
PC                   1.00
```

Spy molecule 11 (Compound S5c)

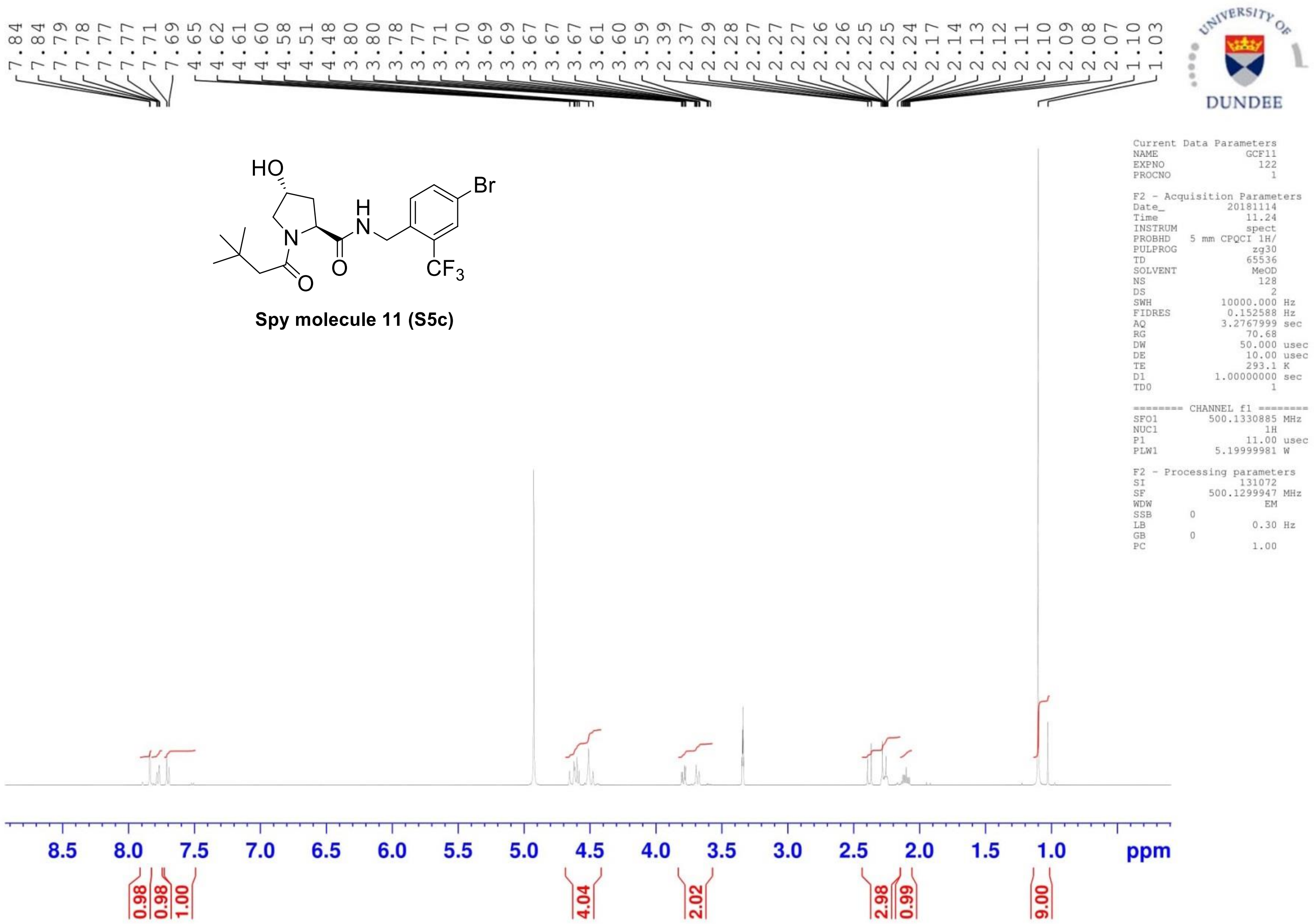

Spy molecule 11 (Compound S5c)

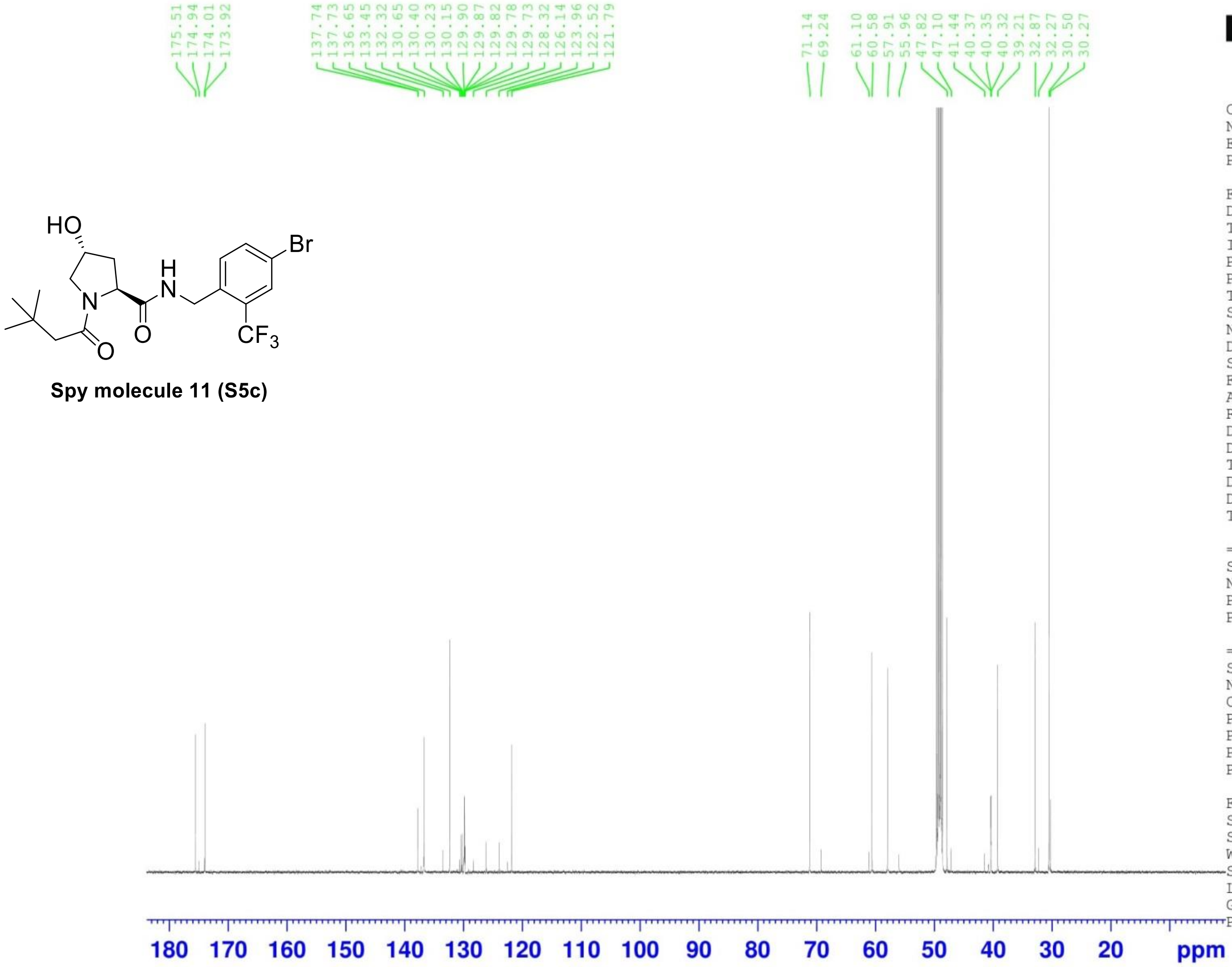

Spy molecule 11 (S5c)


```
Current Data Parameters
NAME                 GCF11
EXPNO                  124
PROCNO                   1

F2 - Acquisition Parameters
Date_             20181116
Time                 10.58
INSTRUM              spect
PROBHD   5 mm CPQCI 1H/
PULPROG            zgpg30
TD                   65400
SOLVENT               MeOD
NS                    3072
DS                       4
SWH              29761.904 Hz
FIDRES            0.455075 Hz
AQ               1.0987200 sec
RG                  190.78
DW                  16.800 usec
DE                   18.00 usec
TE                   293.1 K
D1              2.00000000 sec
D11             0.03000000 sec
TD0                      1

======== CHANNEL f1 ========
SFO1           125.7703637 MHz
NUC1                   13C
P1                   12.00 usec
PLW1          133.00000000 W

======== CHANNEL f2 ========
SFO2           500.1320005 MHz
NUC2                    1H
CPDPRG[2           waltz16
PCPD2               100.00 usec
PLW2            5.19999981 W
PLW12           0.06406900 W
PLW13           0.06406900 W

F2 - Processing parameters
SI                  131072
SF             125.7576002 MHz
WDW                     EM
SSB      0
LB                    1.00 Hz
GB       0
PC                    1.40
```

Spy molecule 11 (Compound S5c)

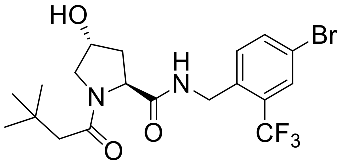

Spy molecule 11 (S5c)

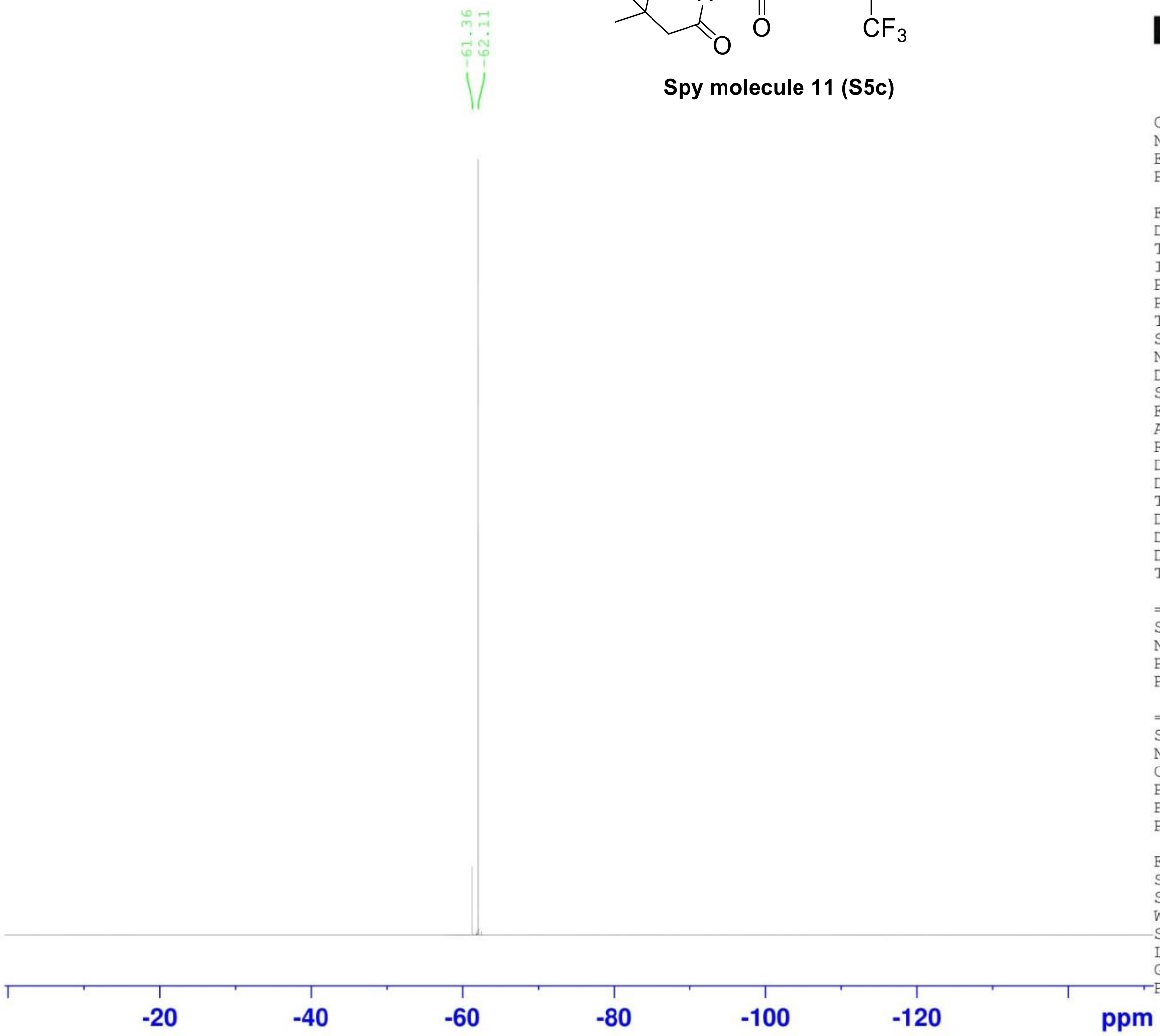

BRUKER

```
Current Data Parameters
NAME                GCF11
EXPNO                 120
PROCNO                  1

F2 - Acquisition Parameters
Date_            20181114
Time                11.14
INSTRUM             spect
PROBHD    5 mm CPQCI 1H/
PULPROG        zgfhigqn.2
TD                 131072
SOLVENT              MeOD
NS                    256
DS                      4
SWH            113636.367 Hz
FIDRES           0.866977 Hz
AQ              0.5767168 sec
RG                 190.78
DW                  4.400 usec
DE                  18.00 usec
TE                  293.1 K
D1             1.00000000 sec
D11            0.03000000 sec
D12            0.00002000 sec
TD0                     1

======== CHANNEL f1 ========
SFO1          470.5453180 MHz
NUC1                  19F
P1                  12.00 usec
PLW1           5.19999981 W

======== CHANNEL f2 ========
SFO2          500.1320005 MHz
NUC2                   1H
CPDPRG[2          waltz16
PCPD2              100.00 usec
PLW2           5.19999981 W
PLW12          0.06406900 W

F2 - Processing parameters
SI                 262144
SF            470.5923772 MHz
WDW                    EM
SSB      0
LB                   1.50 Hz
GB       0
PC                   1.00
```

Spy molecule 15 (Compound S5d)

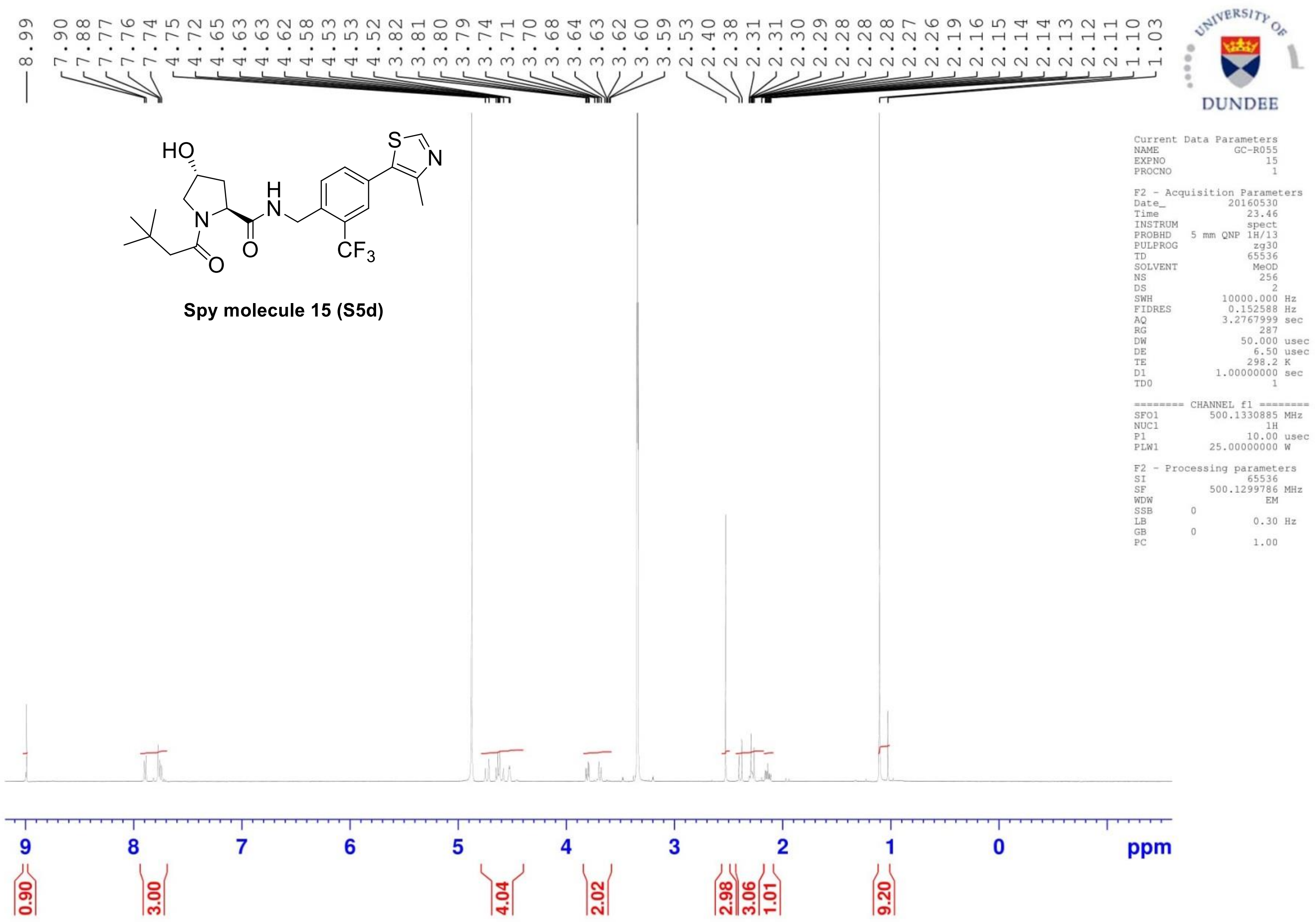

Spy molecule 15 (Compound S5d)

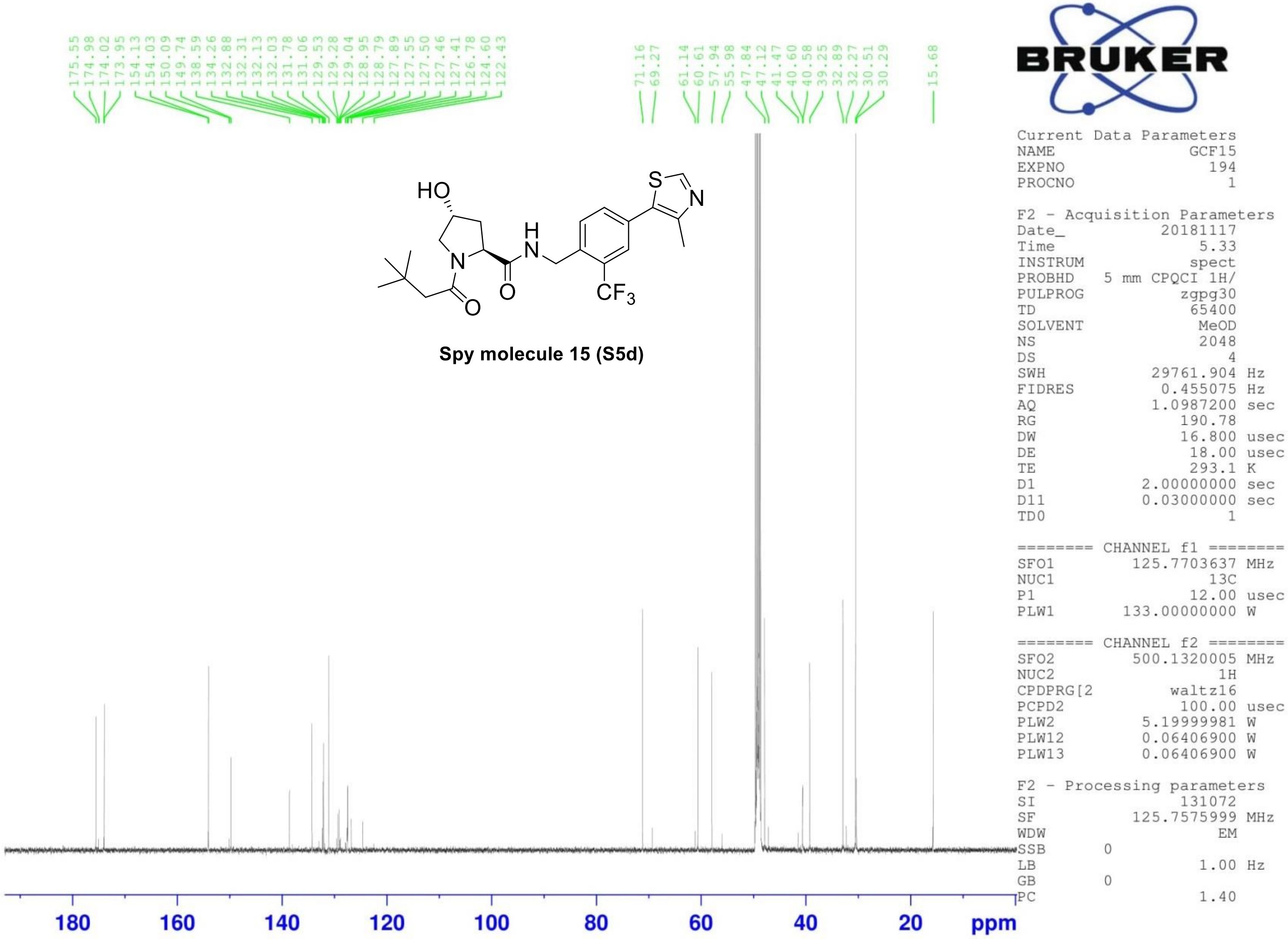

Spy molecule 15 (Compound S5d)

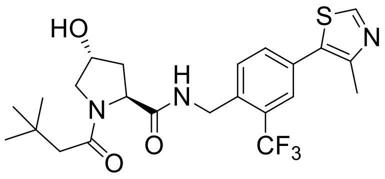

Spy molecule 15 (S5d)

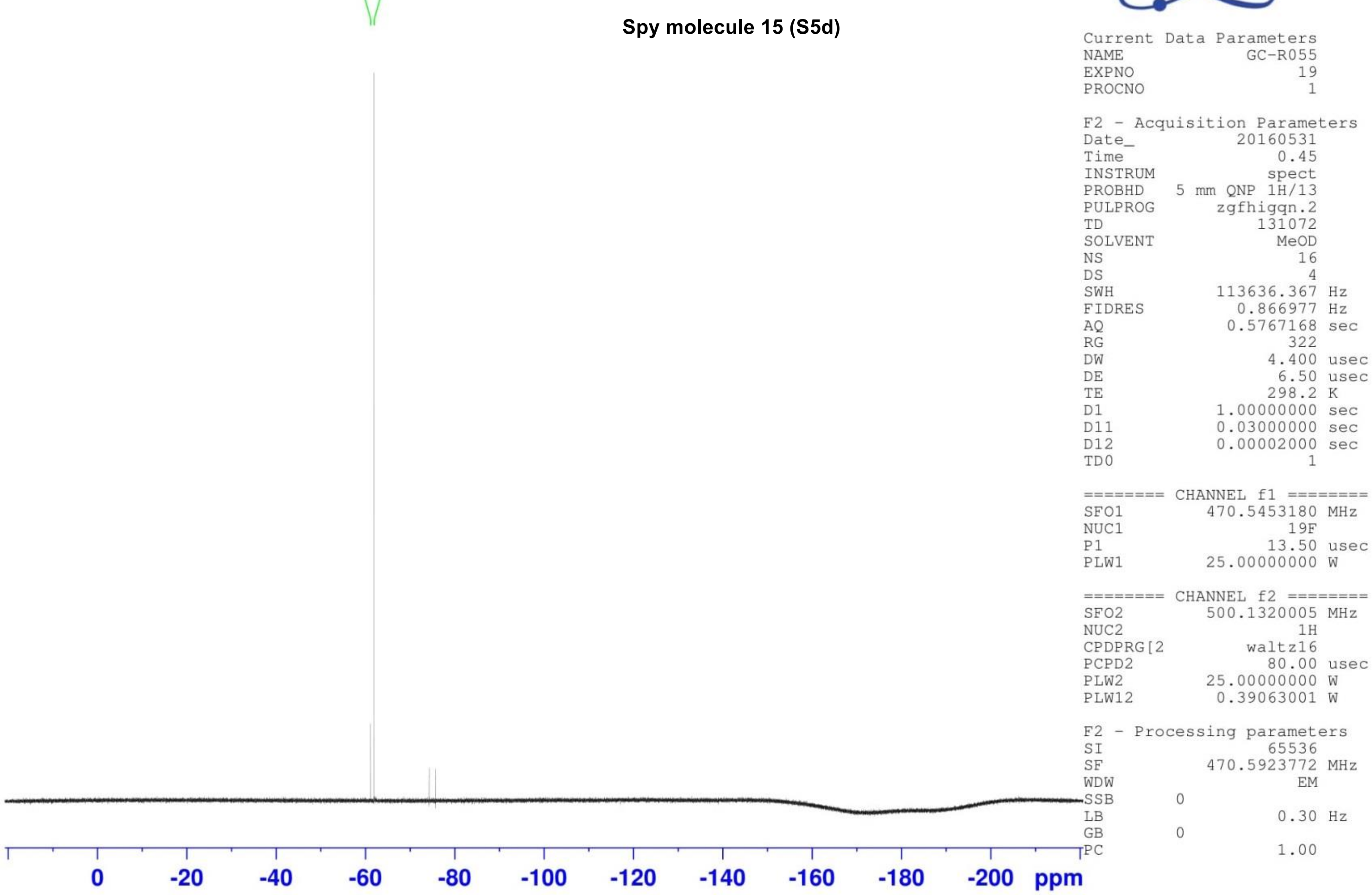

Spy molecule 4 (Compound S6a)

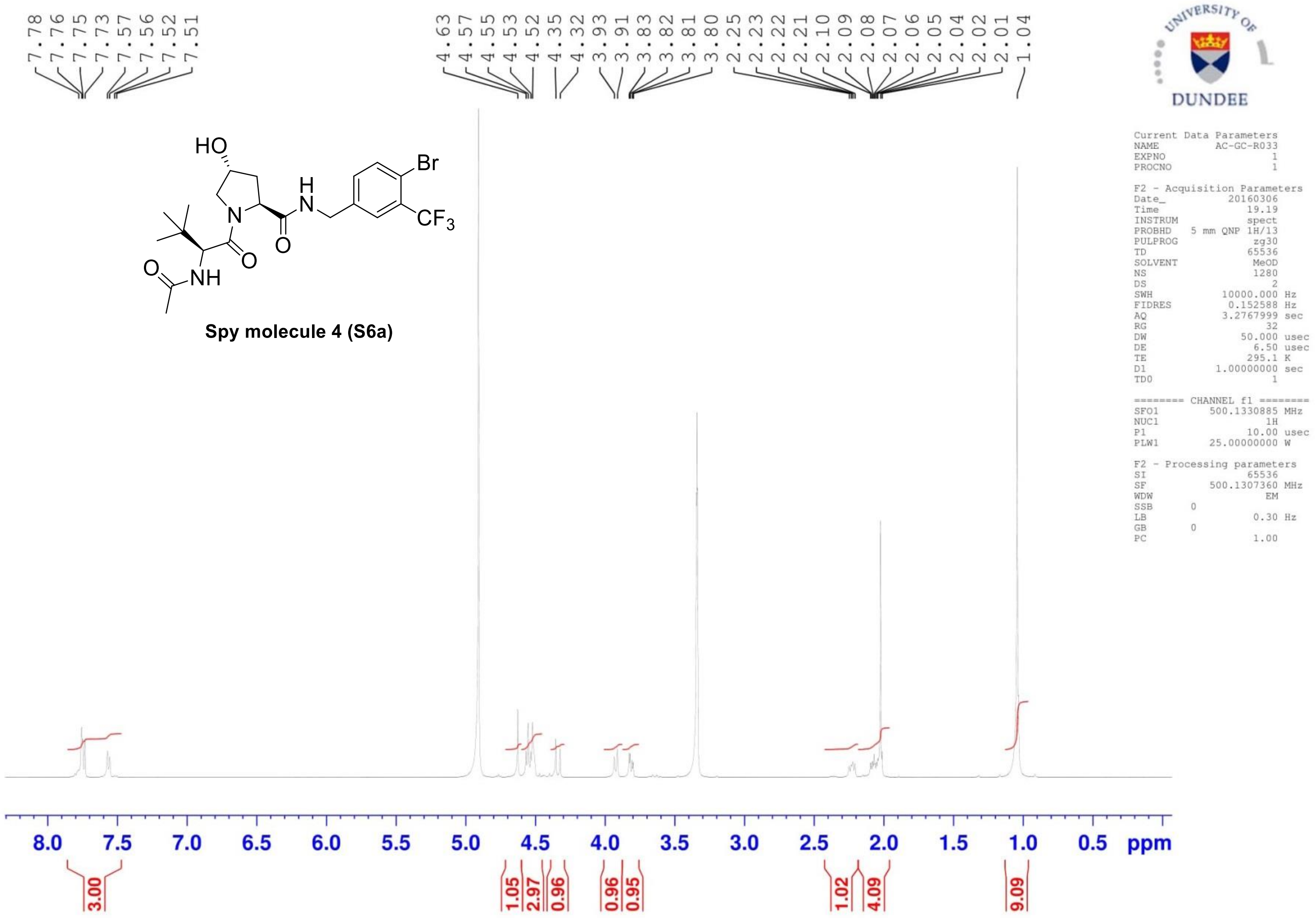

Spy molecule 4 (Compound S6a)

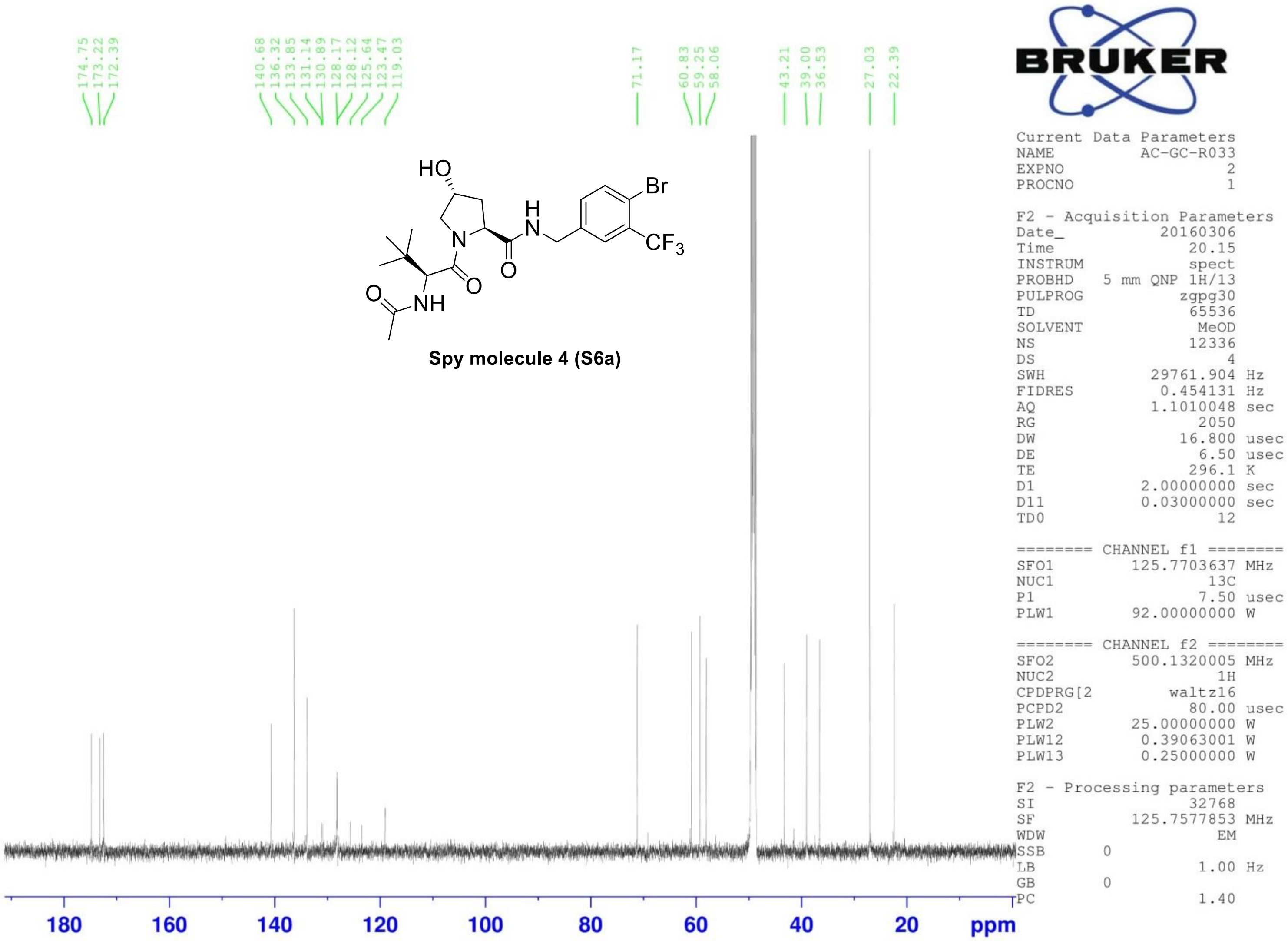

Spy molecule 4 (Compound S6a)

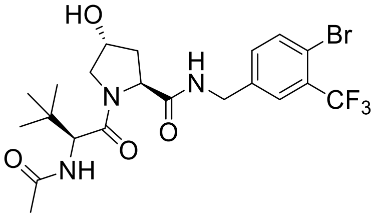

Spy molecule 4 (S6a)

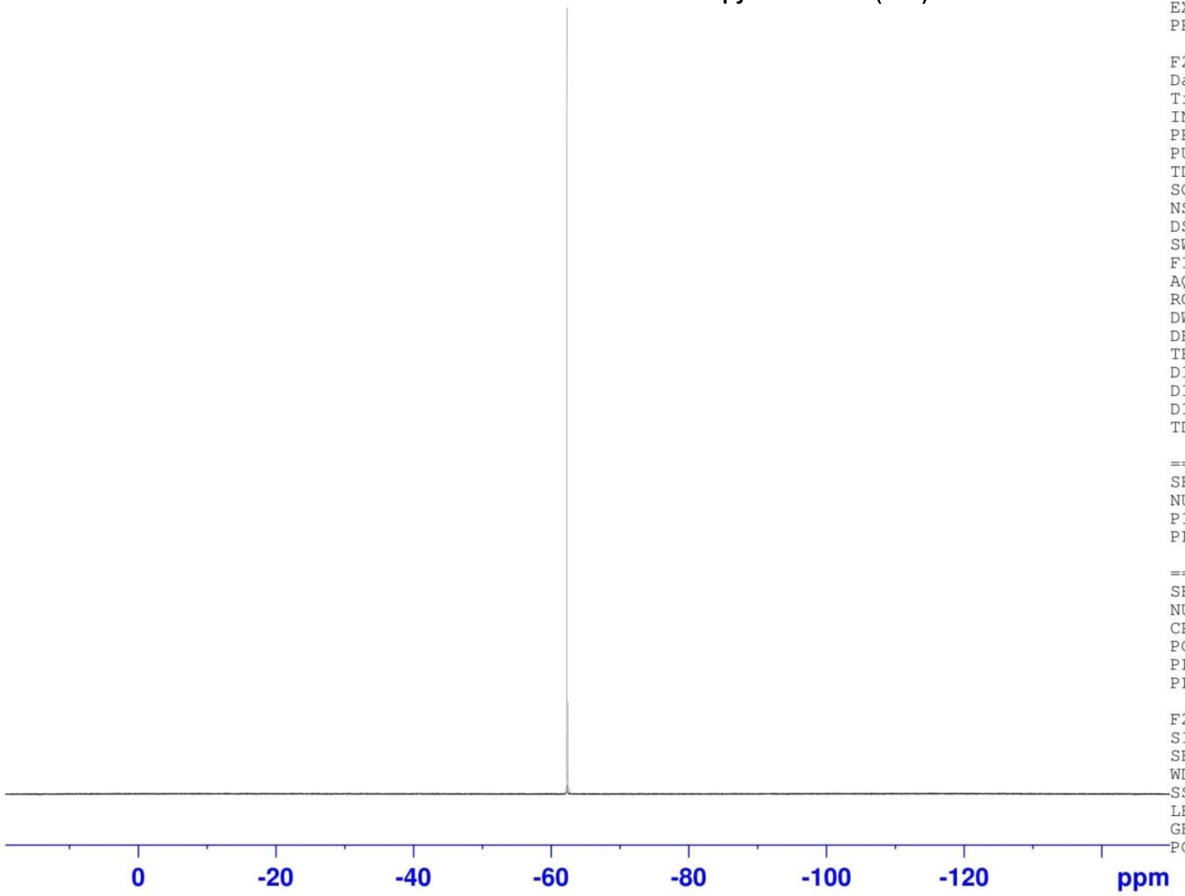


```
Current Data Parameters
NAME              AC-GC-R033
EXPNO                      3
PROCNO                     1

F2 - Acquisition Parameters
Date_               20160307
Time                    6.15
INSTRUM                spect
PROBHD     5 mm QNP 1H/13
PULPROG          zgfhigqn.2
TD                    131072
SOLVENT                 MeOD
NS                        64
DS                         4
SWH               113636.367 Hz
FIDRES              0.866977 Hz
AQ                 0.5767168 sec
RG                       645
DW                     4.400 usec
DE                      6.50 usec
TE                     295.8 K
D1                1.00000000 sec
D11               0.03000000 sec
D12               0.00002000 sec
TD0                        1

======== CHANNEL f1 ========
SFO1            470.5453180 MHz
NUC1                     19F
P1                     13.50 usec
PLW1             25.00000000 W

======== CHANNEL f2 ========
SFO2            500.1320005 MHz
NUC2                      1H
CPDPRG[2             waltz16
PCPD2                  80.00 usec
PLW2             25.00000000 W
PLW12             0.39063001 W

F2 - Processing parameters
SI                     65536
SF              470.5923772 MHz
WDW                       EM
SSB        0
LB                      0.30 Hz
GB         0
PC                      1.00
```

Spy molecule 8 (Compound S6b)

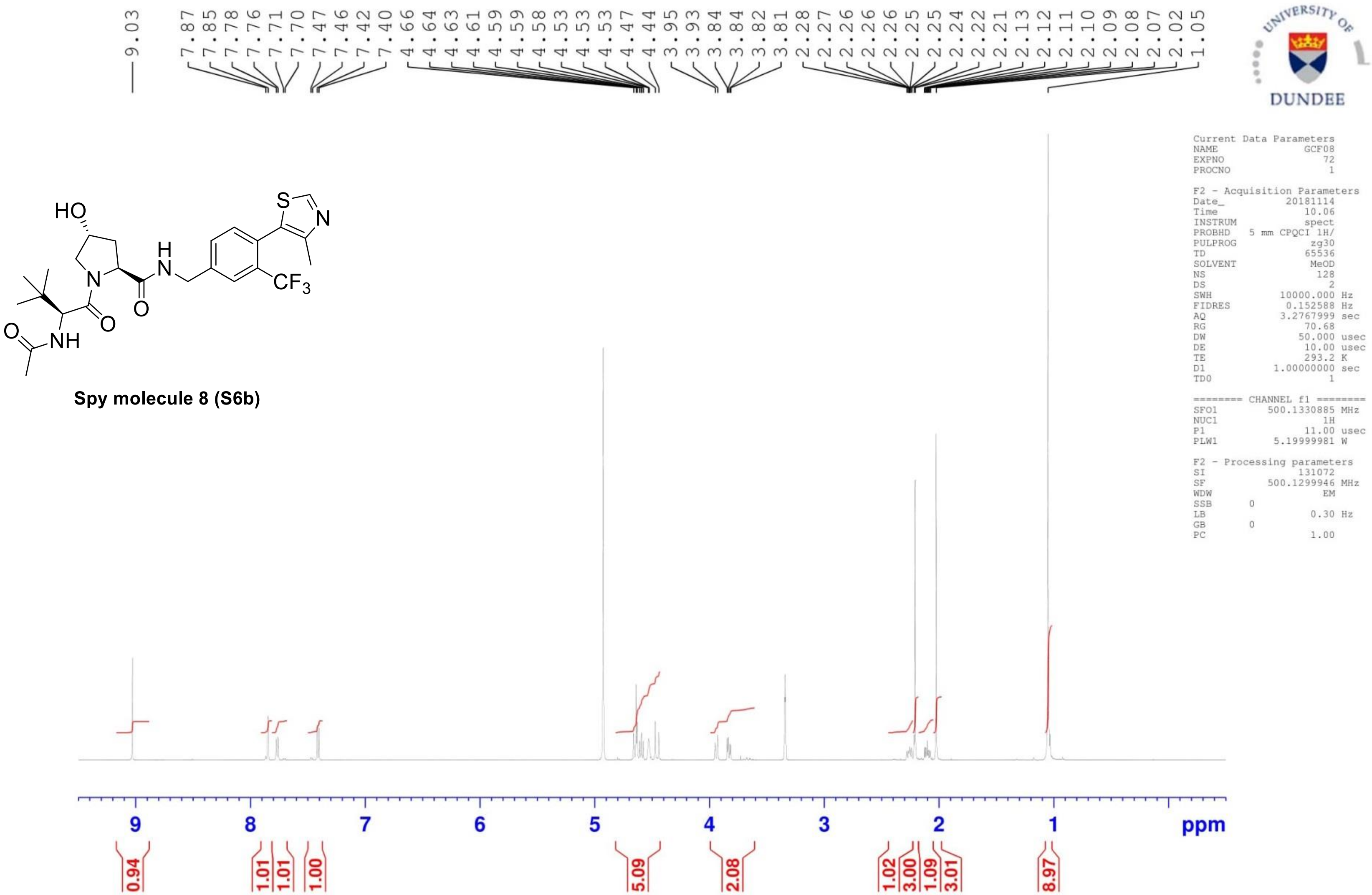

Spy molecule 8 (Compound S6b)

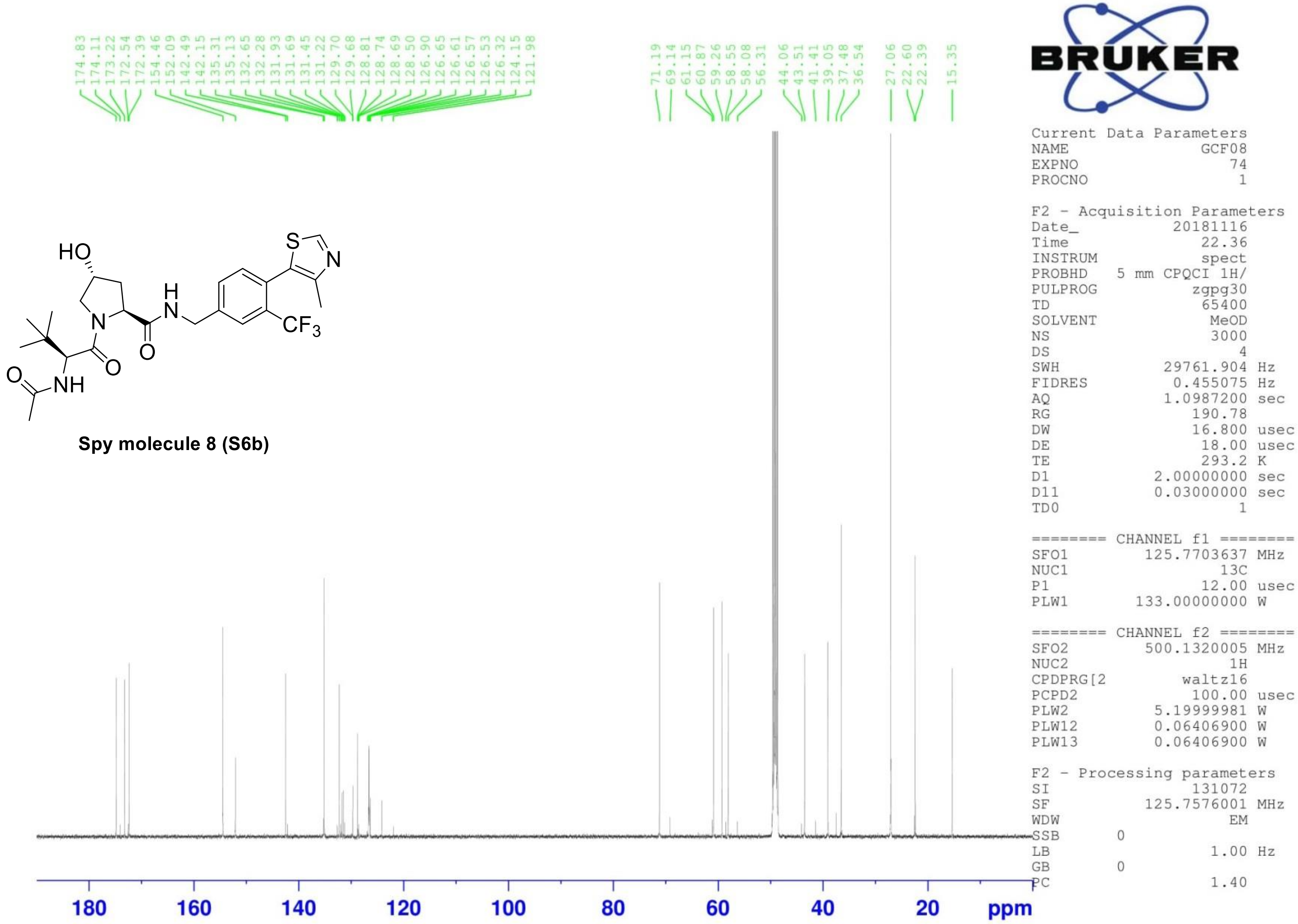

Spy molecule 8 (S6b)

Spy molecule 8 (Compound S6b)

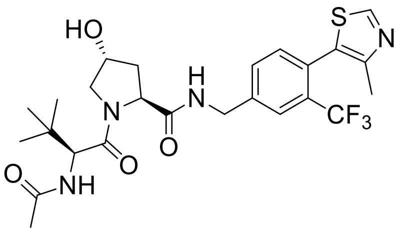

Spy molecule 8 (S6b)

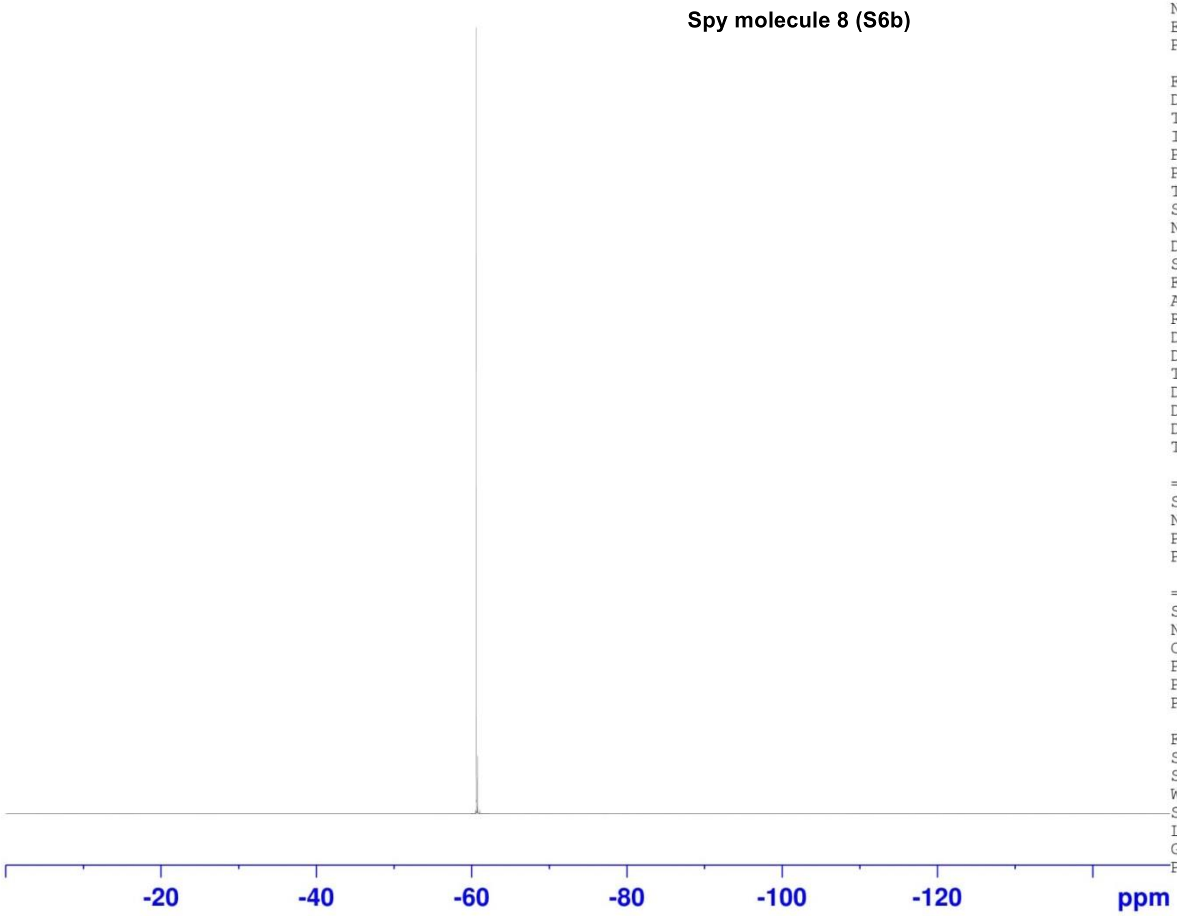

Spy molecule 12 (Compound S6c)

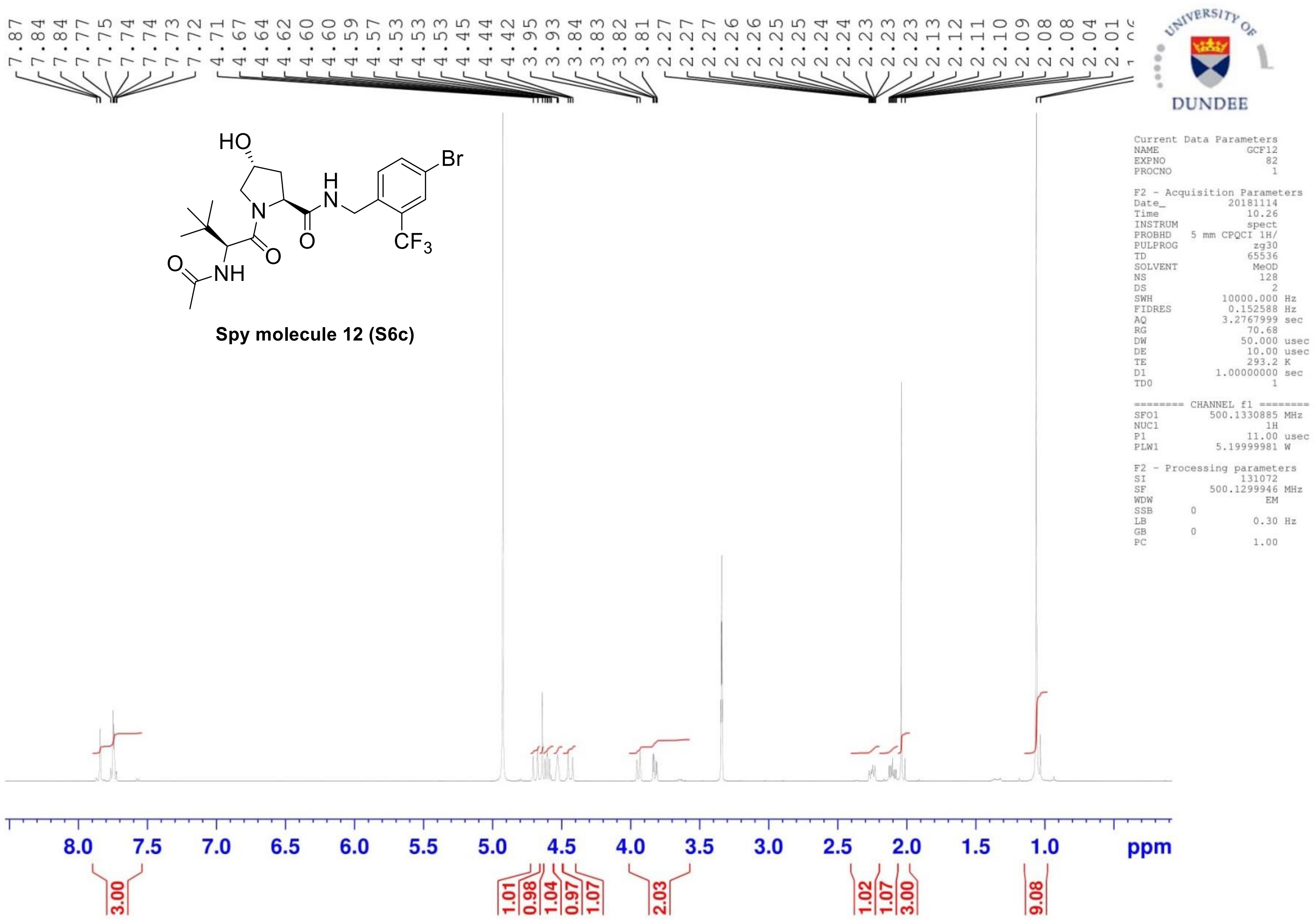

Spy molecule 12 (Compound S6c)

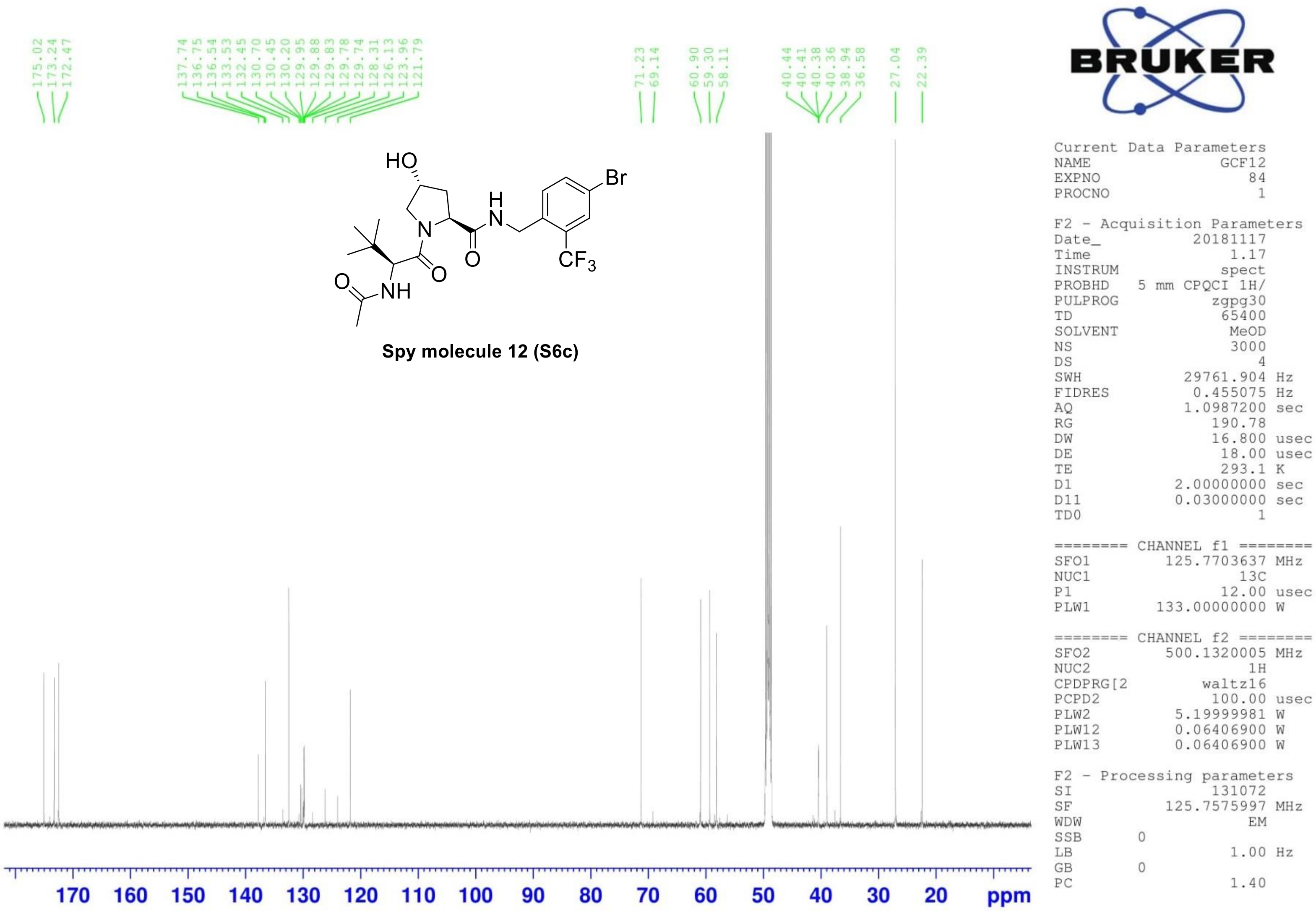

Spy molecule 12 (Compound S6c)

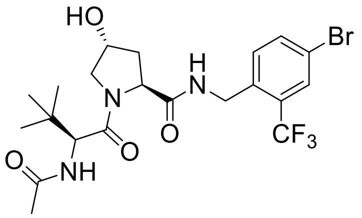

Spy molecule 12 (S6c)

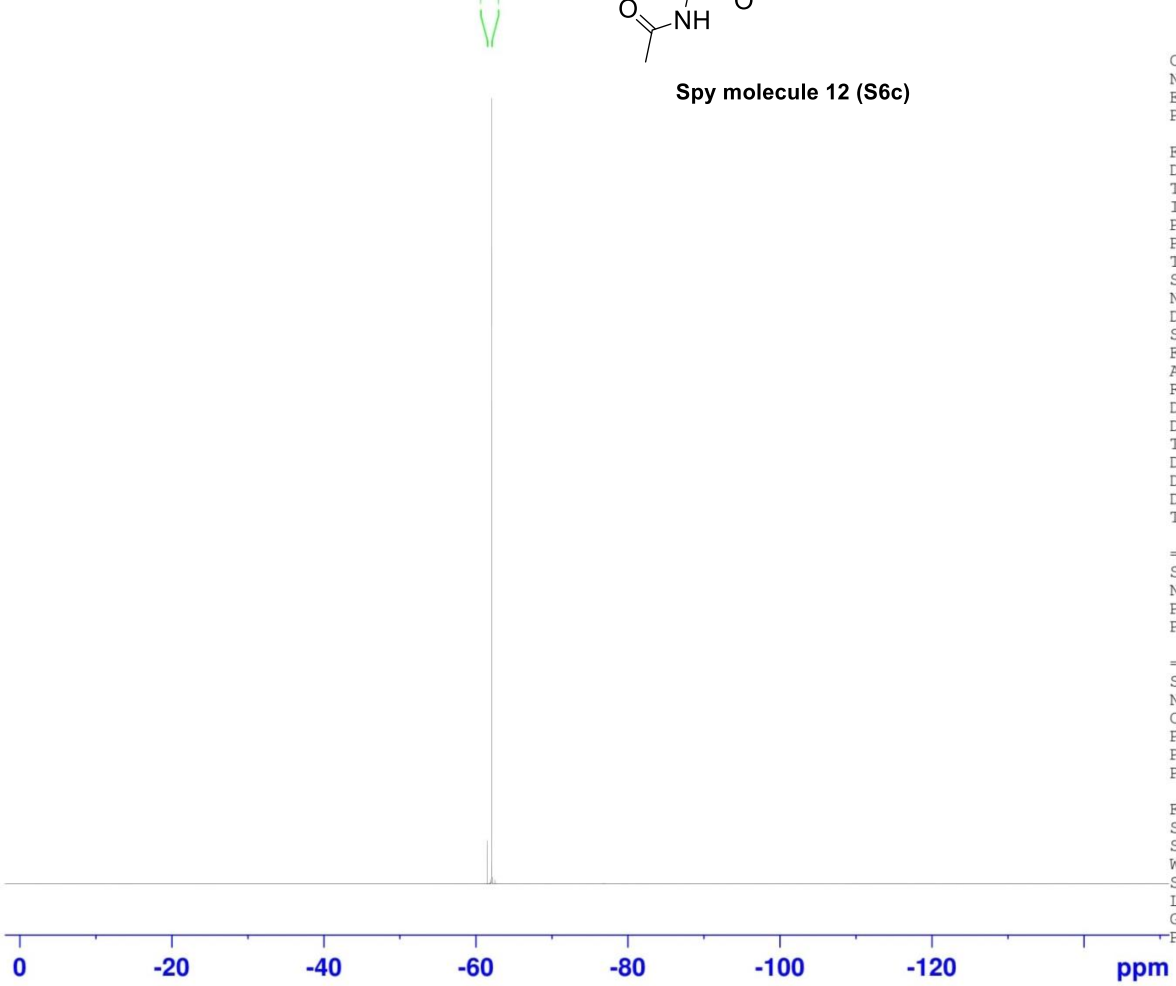


```
Current Data Parameters
NAME                 GCF12
EXPNO                   80
PROCNO                   1

F2 - Acquisition Parameters
Date_             20181114
Time                 10.16
INSTRUM              spect
PROBHD   5 mm CPQCI 1H/
PULPROG         zgfhigqn.2
TD                  131072
SOLVENT               MeOD
NS                     256
DS                       4
SWH             113636.367 Hz
FIDRES            0.866977 Hz
AQ               0.5767168 sec
RG                  190.78
DW                   4.400 usec
DE                   18.00 usec
TE                   293.1 K
D1              1.00000000 sec
D11             0.03000000 sec
D12             0.00002000 sec
TD0                      1

======== CHANNEL f1 ========
SFO1        470.5453180 MHz
NUC1                   19F
P1                   12.00 usec
PLW1            5.19999981 W

======== CHANNEL f2 ========
SFO2        500.1320005 MHz
NUC2                    1H
CPDPRG[2           waltz16
PCPD2               100.00 usec
PLW2            5.19999981 W
PLW12           0.06406900 W

F2 - Processing parameters
SI                  262144
SF          470.5923772 MHz
WDW                     EM
SSB      0
LB                    1.50 Hz
GB       0
PC                    1.00
```

Spy molecule 16 (Compound S6d)

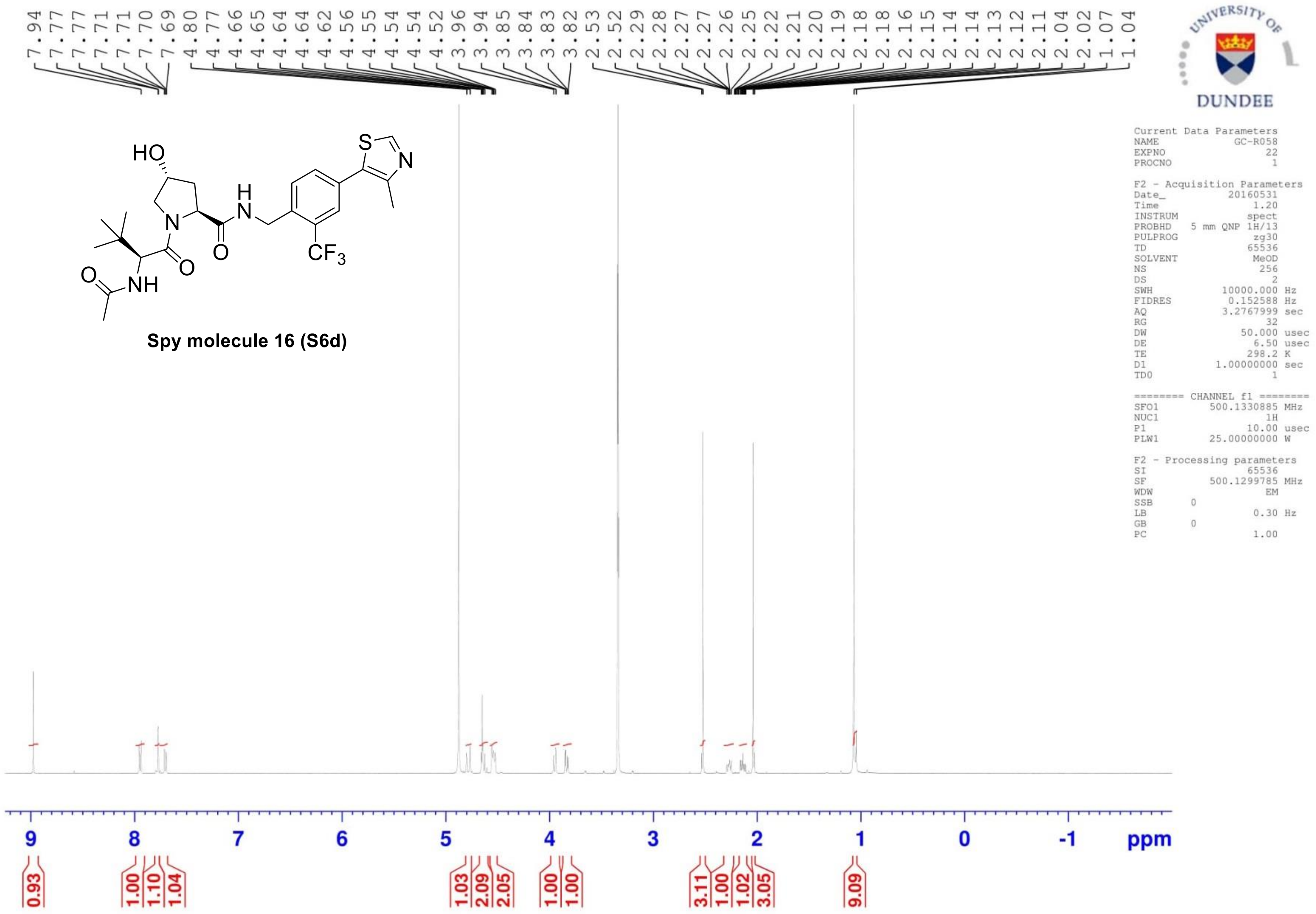

Spy molecule 16 (Compound S6d)

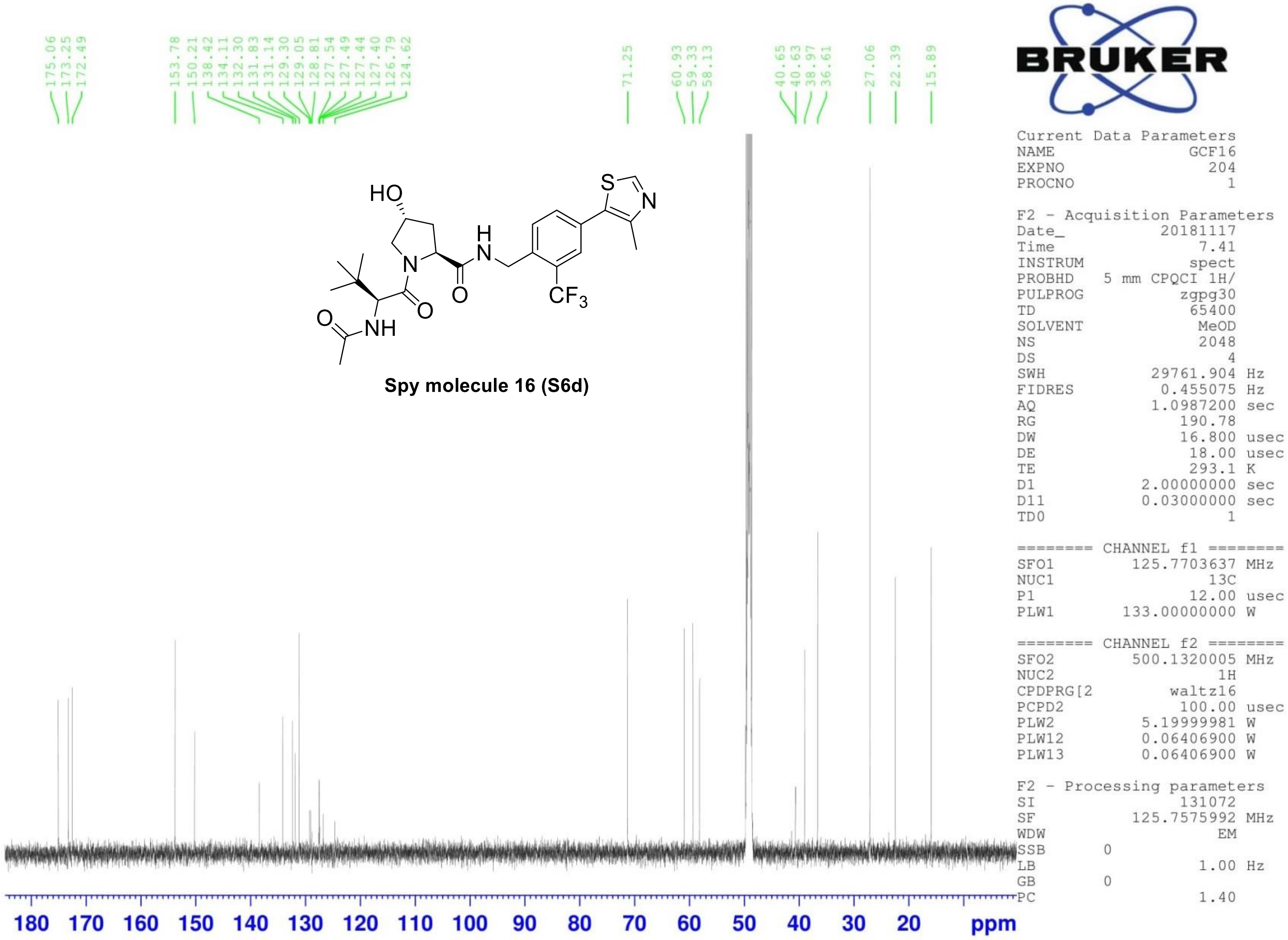

Spy molecule 16 (S6d)

BRUKER

```
Current Data Parameters
NAME                GCF16
EXPNO                 204
PROCNO                  1

F2 - Acquisition Parameters
Date_            20181117
Time                 7.41
INSTRUM             spect
PROBHD   5 mm CPQCI 1H/
PULPROG            zgpg30
TD                  65400
SOLVENT              MeOD
NS                   2048
DS                      4
SWH            29761.904 Hz
FIDRES          0.455075 Hz
AQ             1.0987200 sec
RG                 190.78
DW                 16.800 usec
DE                  18.00 usec
TE                  293.1 K
D1             2.00000000 sec
D11            0.03000000 sec
TD0                     1

======== CHANNEL f1 ========
SFO1         125.7703637 MHz
NUC1                  13C
P1                  12.00 usec
PLW1         133.00000000 W

======== CHANNEL f2 ========
SFO2         500.1320005 MHz
NUC2                   1H
CPDPRG[2             waltz16
PCPD2              100.00 usec
PLW2           5.19999981 W
PLW12          0.06406900 W
PLW13          0.06406900 W

F2 - Processing parameters
SI                 131072
SF           125.7575992 MHz
WDW                    EM
SSB        0
LB                   1.00 Hz
GB         0
PC                   1.40
```

Spy molecule 16 (Compound S6d)

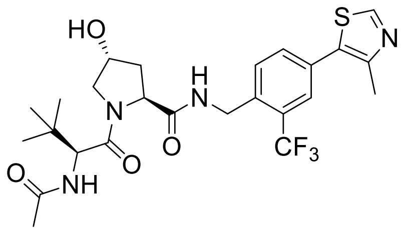

Spy molecule 16 (S6d)

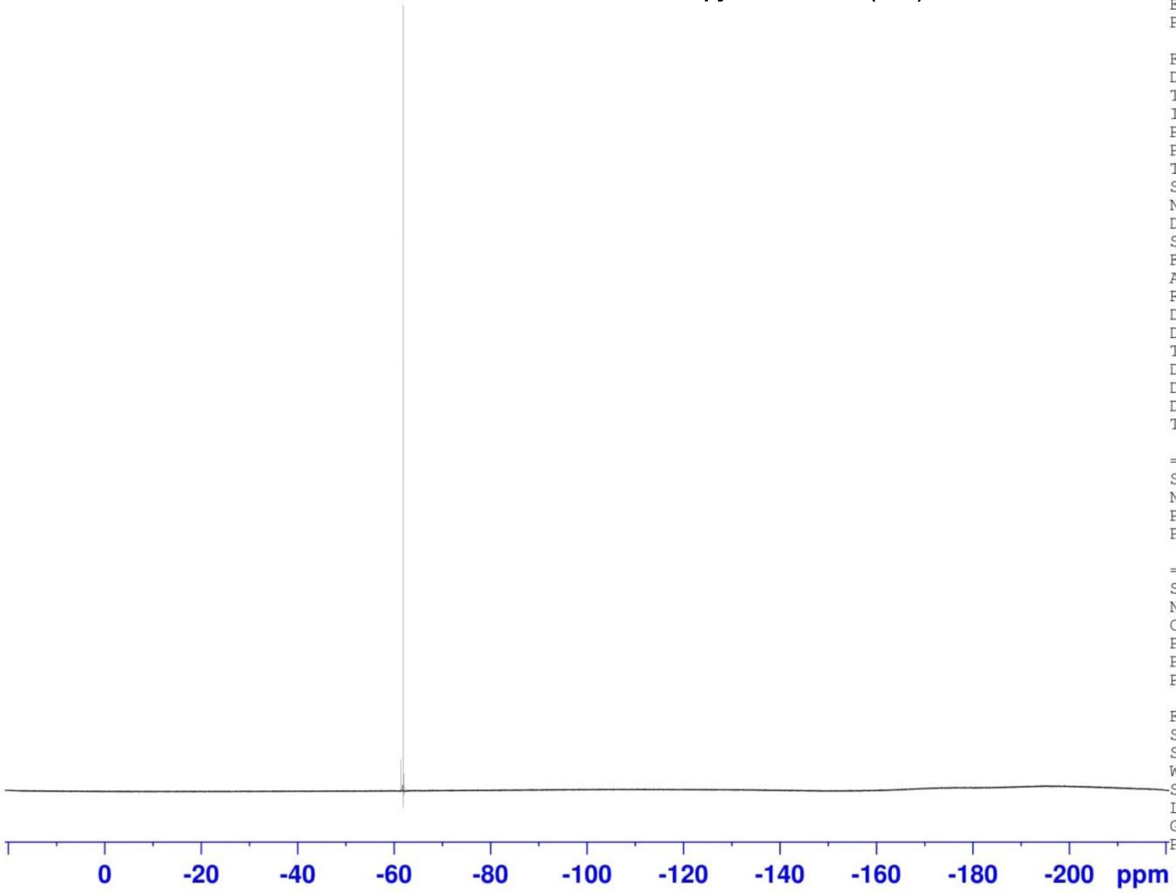


```
Current Data Parameters
NAME              GC-R058
EXPNO                  27
PROCNO                  1

F2 - Acquisition Parameters
Date_            20160531
Time                 2.19
INSTRUM             spect
PROBHD   5 mm QNP 1H/13
PULPROG        zgfhigqn.2
TD                 131072
SOLVENT              MeOD
NS                     16
DS                      4
SWH            113636.367 Hz
FIDRES           0.866977 Hz
AQ              0.5767168 sec
RG                    322
DW                  4.400 usec
DE                   6.50 usec
TE                  298.2 K
D1             1.00000000 sec
D11            0.03000000 sec
D12            0.00002000 sec
TD0                     1

======== CHANNEL f1 ========
SFO1          470.5453180 MHz
NUC1                  19F
P1                  13.50 usec
PLW1          25.00000000 W

======== CHANNEL f2 ========
SFO2          500.1320005 MHz
NUC2                   1H
CPDPRG[2          waltz16
PCPD2               80.00 usec
PLW2          25.00000000 W
PLW12          0.39063001 W

F2 - Processing parameters
SI                  65536
SF            470.5923772 MHz
WDW                    EM
SSB      0
LB                   0.30 Hz
GB       0
PC                   1.00
```

Compound S9a

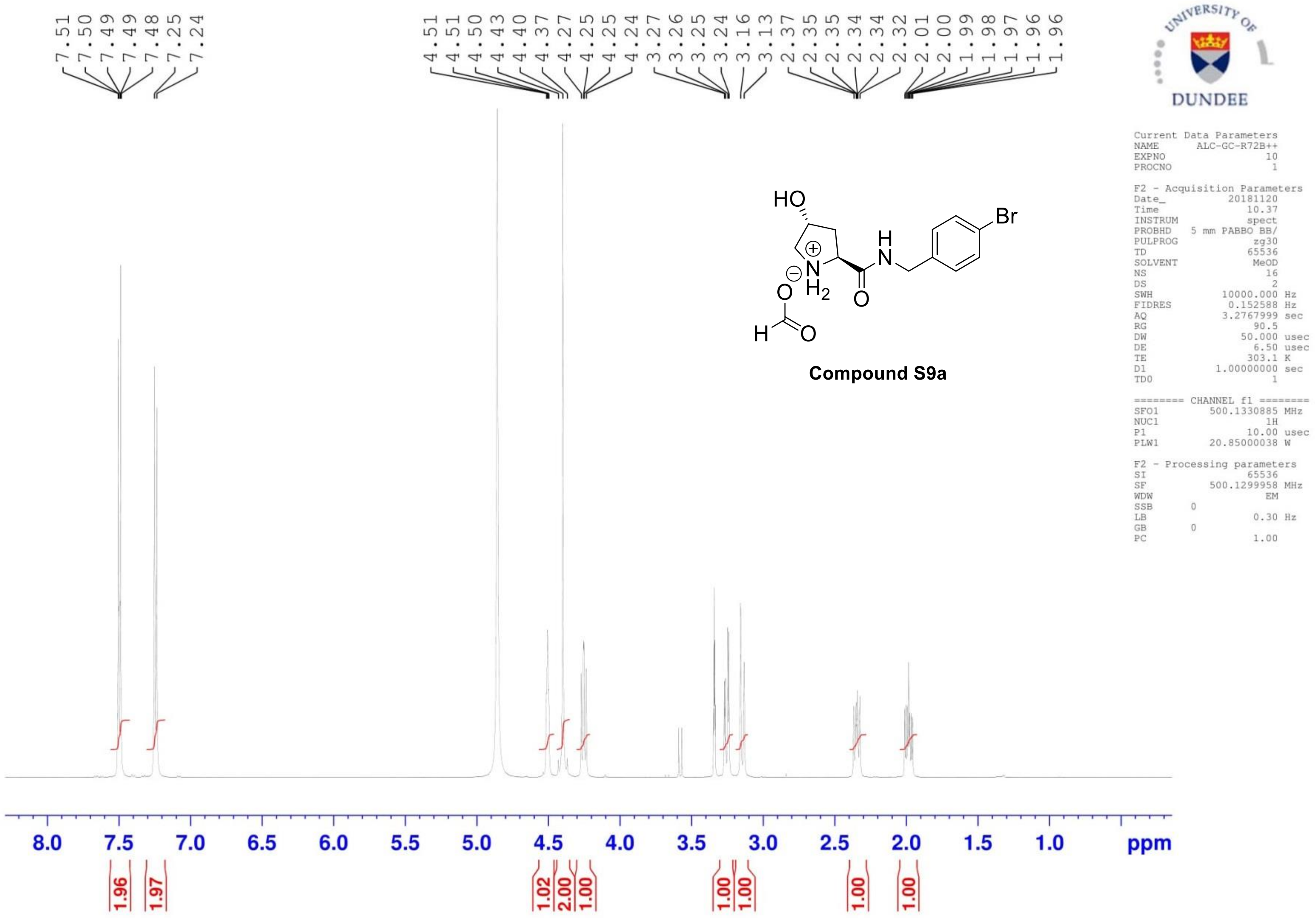

Compound S9a

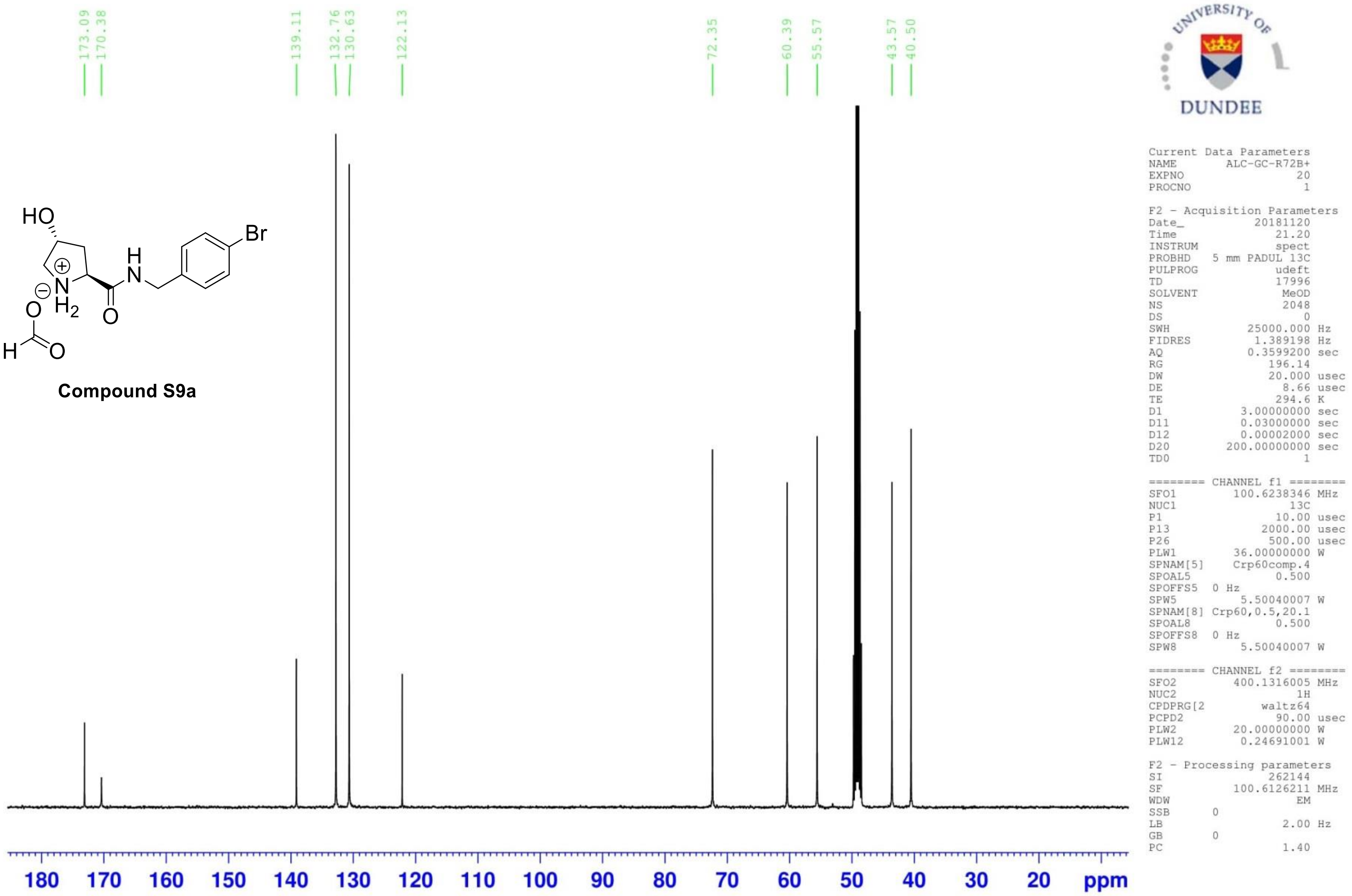

Spy molecule 17 (Compound S10a)

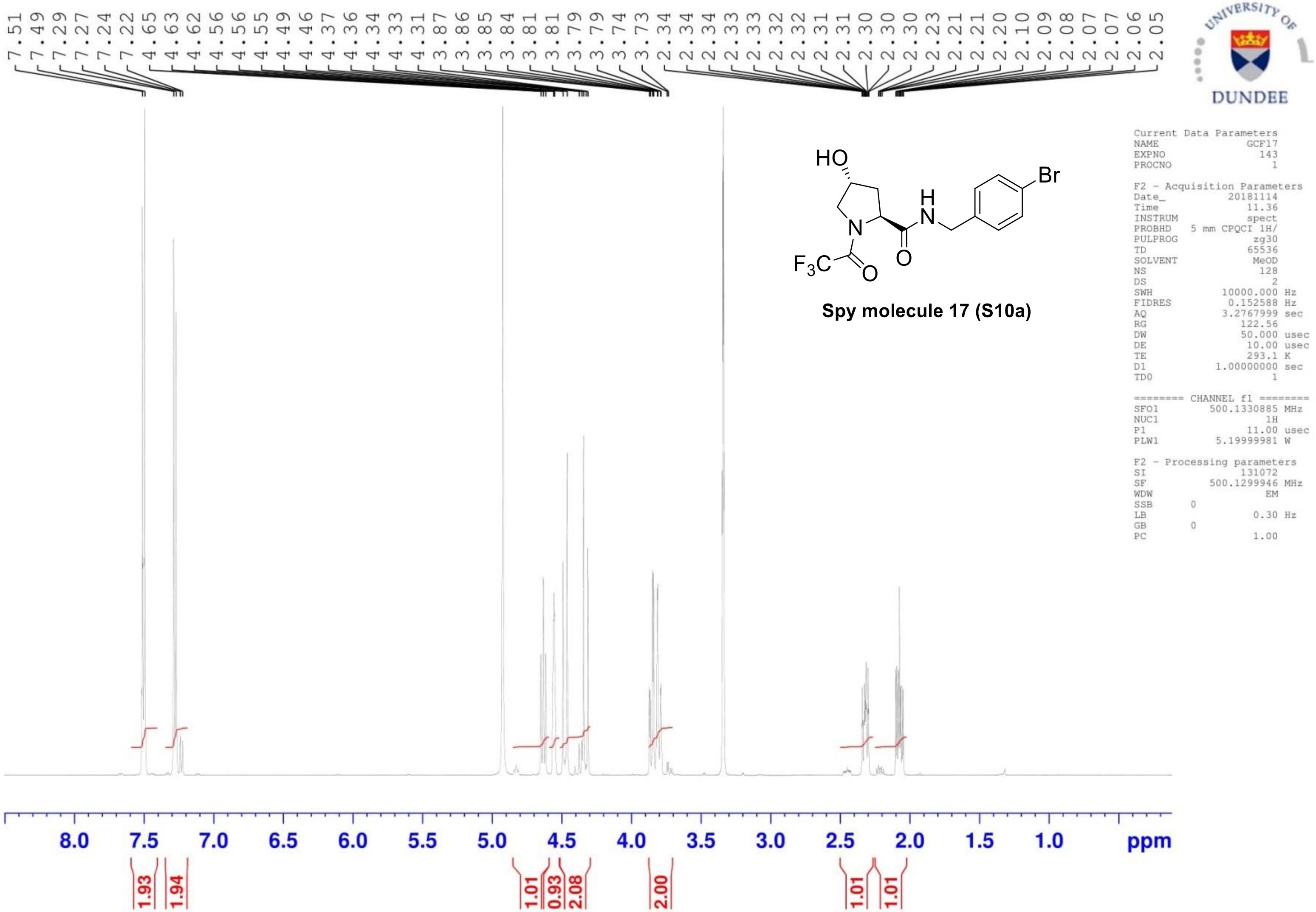

Spy molecule 17 (Compound S10a)

Spy molecule 17 (S10a)

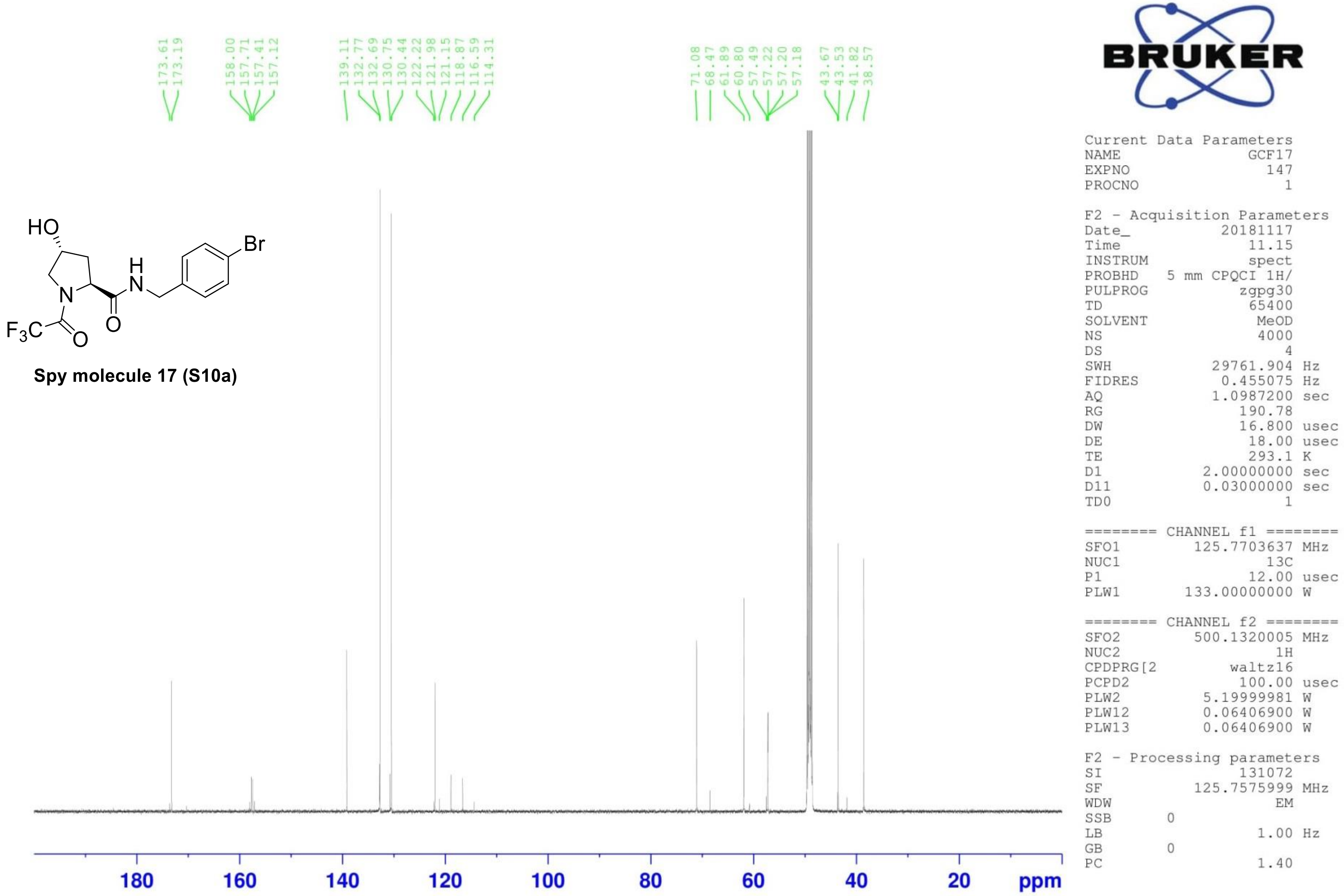

Spy molecule 17 (Compound S10a)

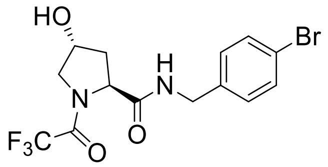

Spy molecule 17 (S10a)

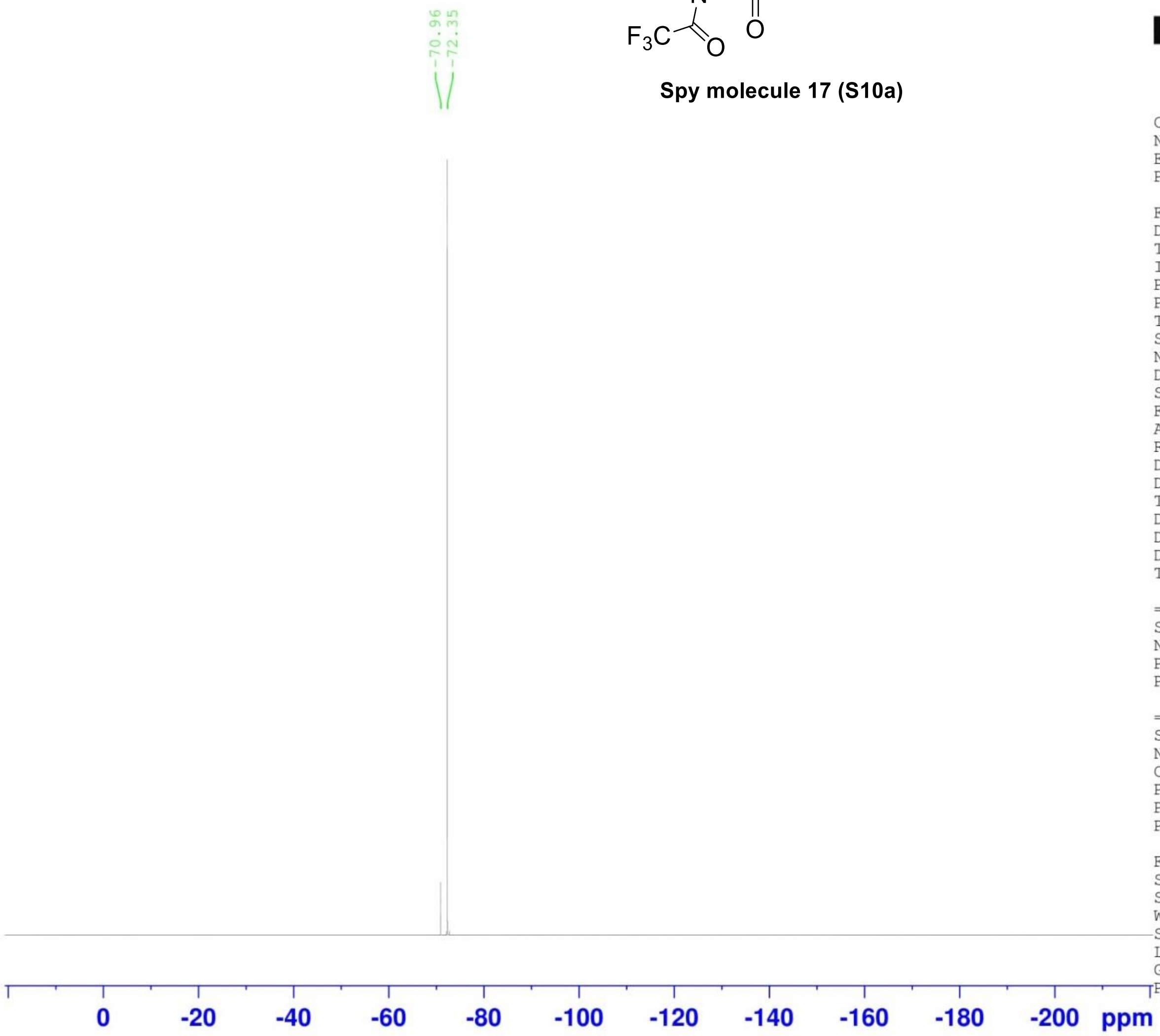

Current Data Parameters
NAME GCF17
EXPNO 141
PROCNO 1

F2 - Acquisition Parameters
Date_ 20181114
Time 0.15
INSTRUM spect
PROBHD 5 mm CPQCI 1H/
PULPROG zgfhigqn.2
TD 131072
SOLVENT H2O+D2O_salt
NS 256
DS 4
SWH 113636.367 Hz
FIDRES 0.866977 Hz
AQ 0.5767168 sec
RG 190.78
DW 4.400 usec
DE 18.00 usec
TE 293.1 K
D1 1.00000000 sec
D11 0.03000000 sec
D12 0.00002000 sec
TD0 1

======== CHANNEL f1 ========
SFO1 470.5453180 MHz
NUC1 19F
P1 12.00 usec
PLW1 5.19999981 W

======== CHANNEL f2 ========
SFO2 500.1320005 MHz
NUC2 1H
CPDPRG[2 waltz16
PCPD2 100.00 usec
PLW2 5.19999981 W
PLW12 0.06406900 W

F2 - Processing parameters
SI 262144
SF 470.5923772 MHz
WDW EM
SSB 0
LB 1.50 Hz
GB 0
PC 1.00

Spy molecule 18 (Compound S10b)

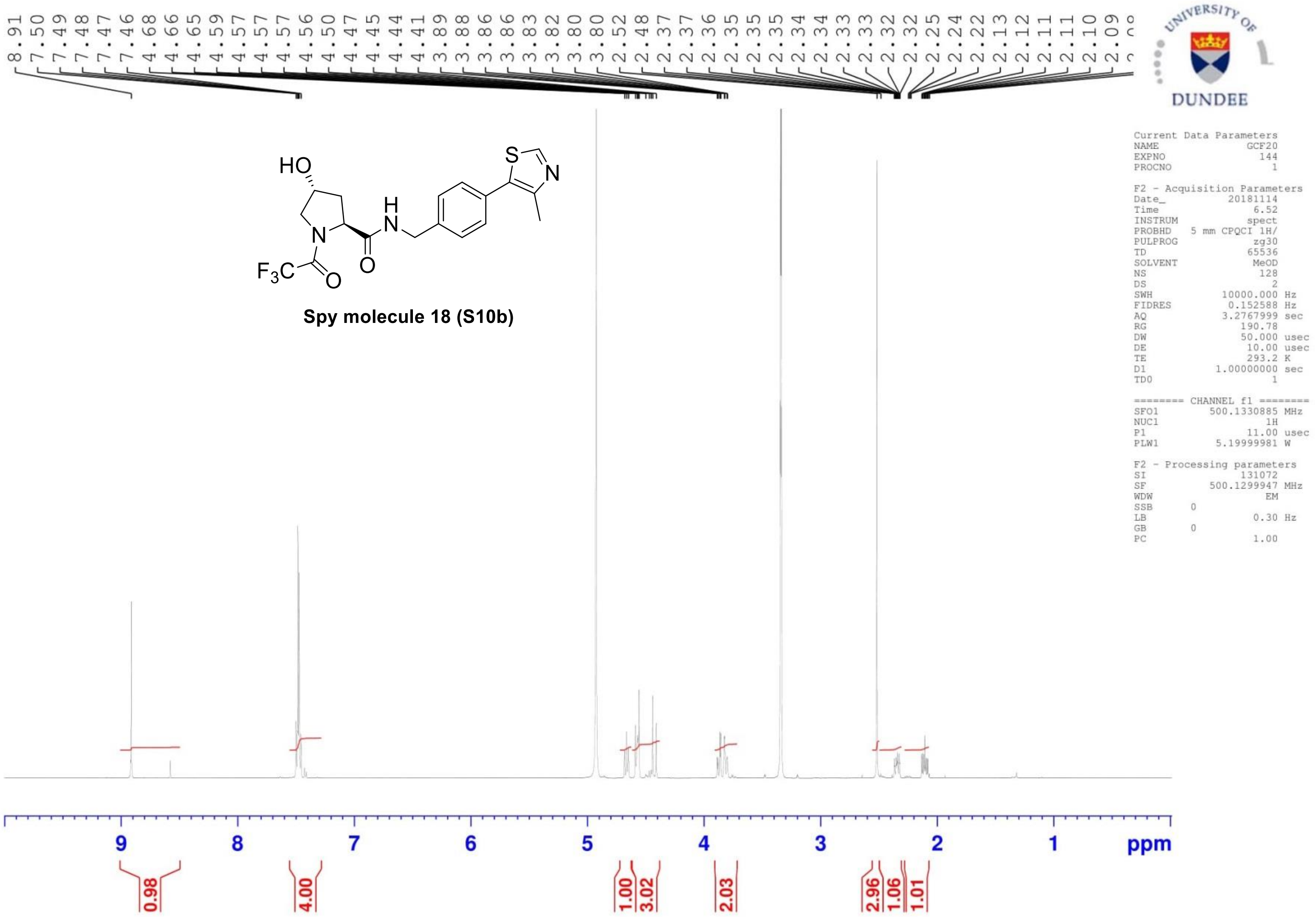

Spy molecule 18 (Compound S10b)

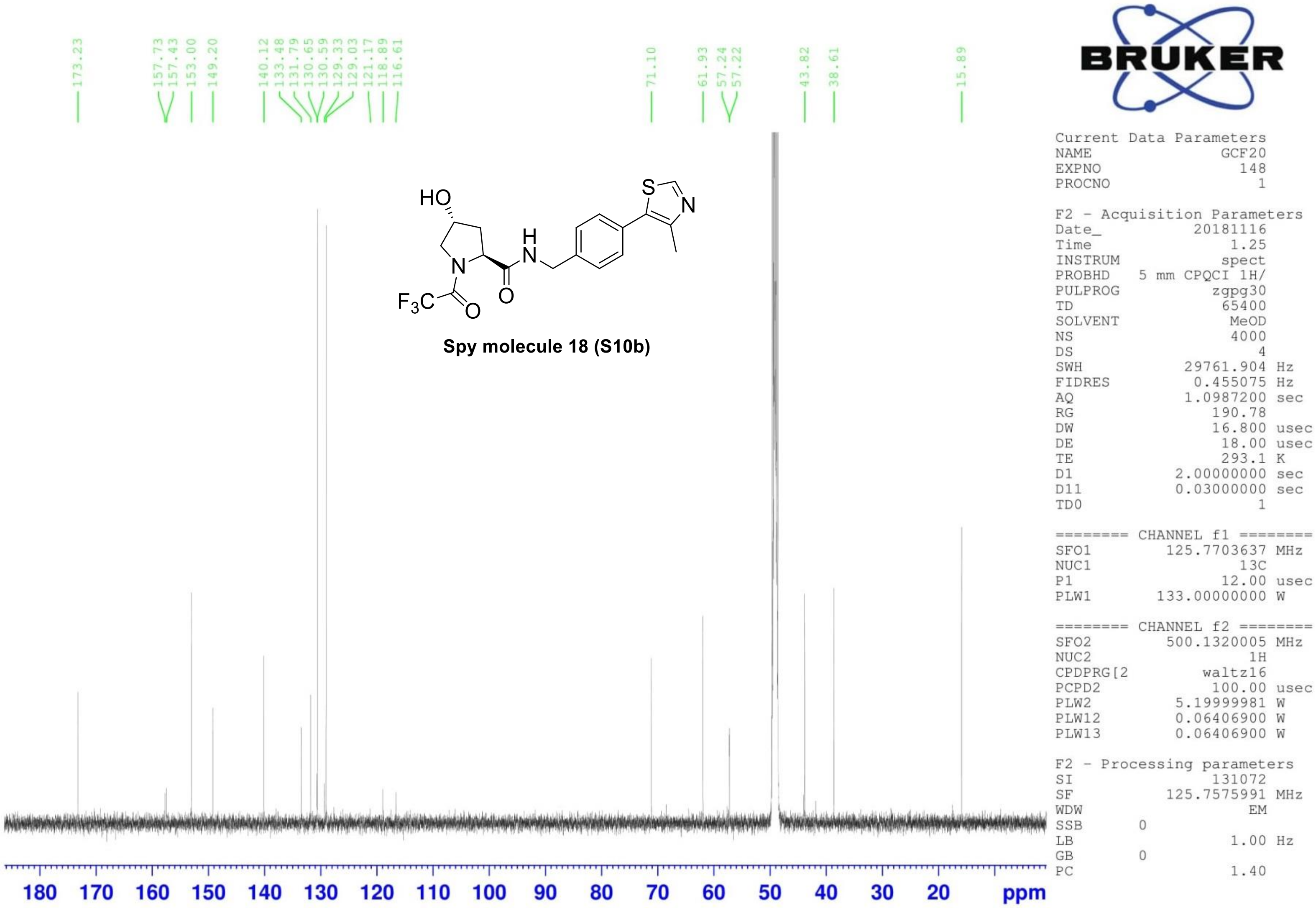

Spy molecule 18 (Compound S10b)

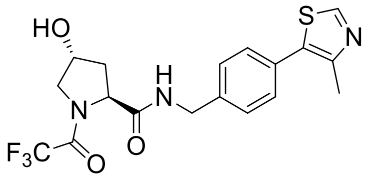

Spy molecule 18 (S10b)

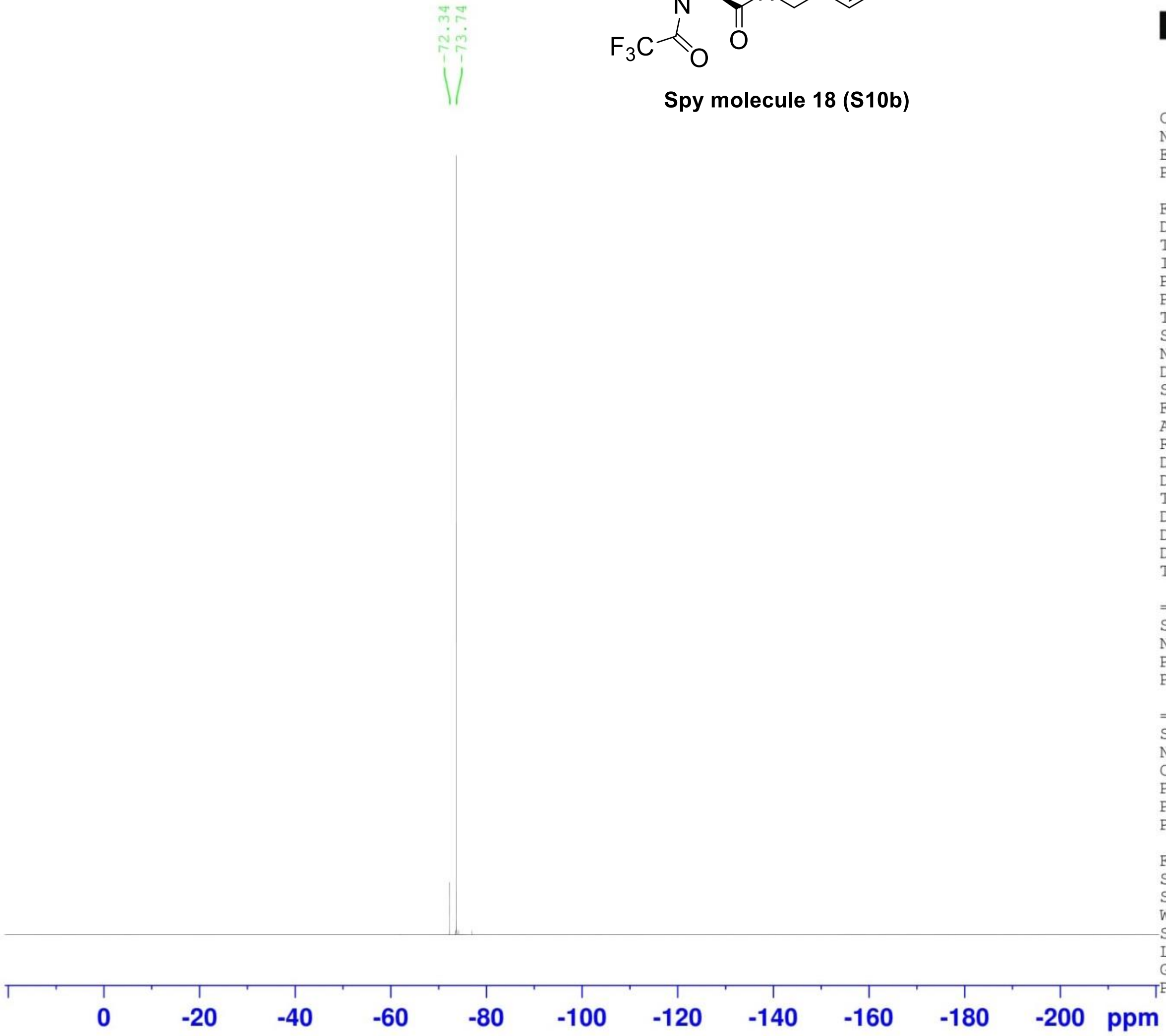


```
Current Data Parameters
NAME                 GCF20
EXPNO                  146
PROCNO                   1

F2 - Acquisition Parameters
Date_             20181114
Time                  8.48
INSTRUM              spect
PROBHD    5 mm CPQCI 1H/
PULPROG        zgfhigqn.2
TD                  131072
SOLVENT               MeOD
NS                     256
DS                       4
SWH          113636.367 Hz
FIDRES         0.866977 Hz
AQ            0.5767168 sec
RG                  190.78
DW                4.400 usec
DE                18.00 usec
TE                 293.2 K
D1           1.00000000 sec
D11          0.03000000 sec
D12          0.00002000 sec
TD0                      1

======== CHANNEL f1 ========
SFO1        470.5453180 MHz
NUC1                   19F
P1                12.00 usec
PLW1          5.19999981 W

======== CHANNEL f2 ========
SFO2        500.1320005 MHz
NUC2                    1H
CPDPRG[2           waltz16
PCPD2            100.00 usec
PLW2          5.19999981 W
PLW12         0.06406900 W

F2 - Processing parameters
SI                  262144
SF          470.5923772 MHz
WDW                     EM
SSB      0
LB                 1.50 Hz
GB       0
PC                    1.00
```

Spy molecule 19 (Compound S11a)

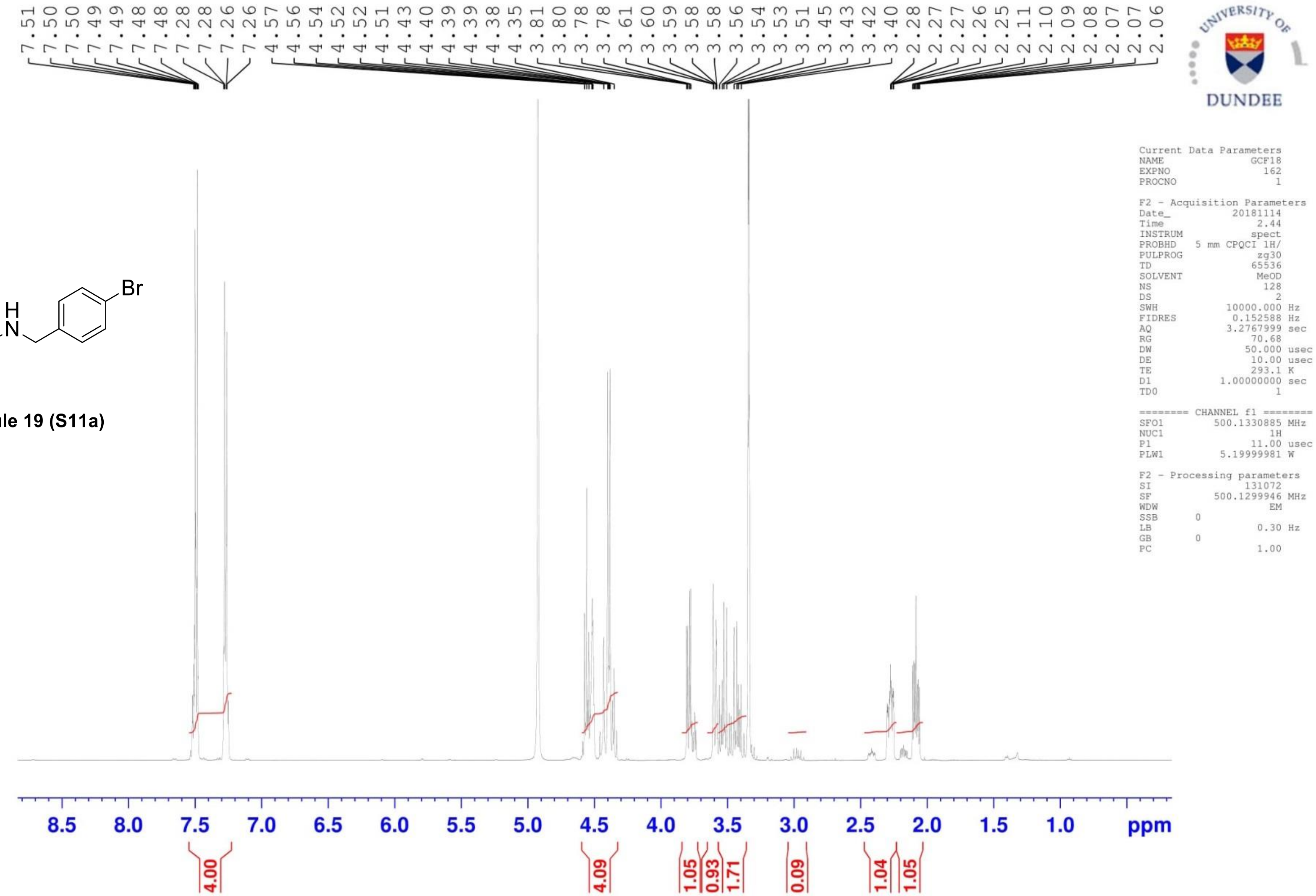

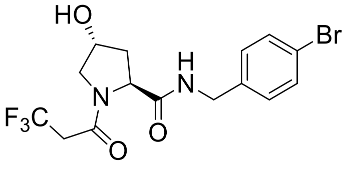

Spy molecule 19 (S11a)

Spy molecule 19 (Compound S11a)

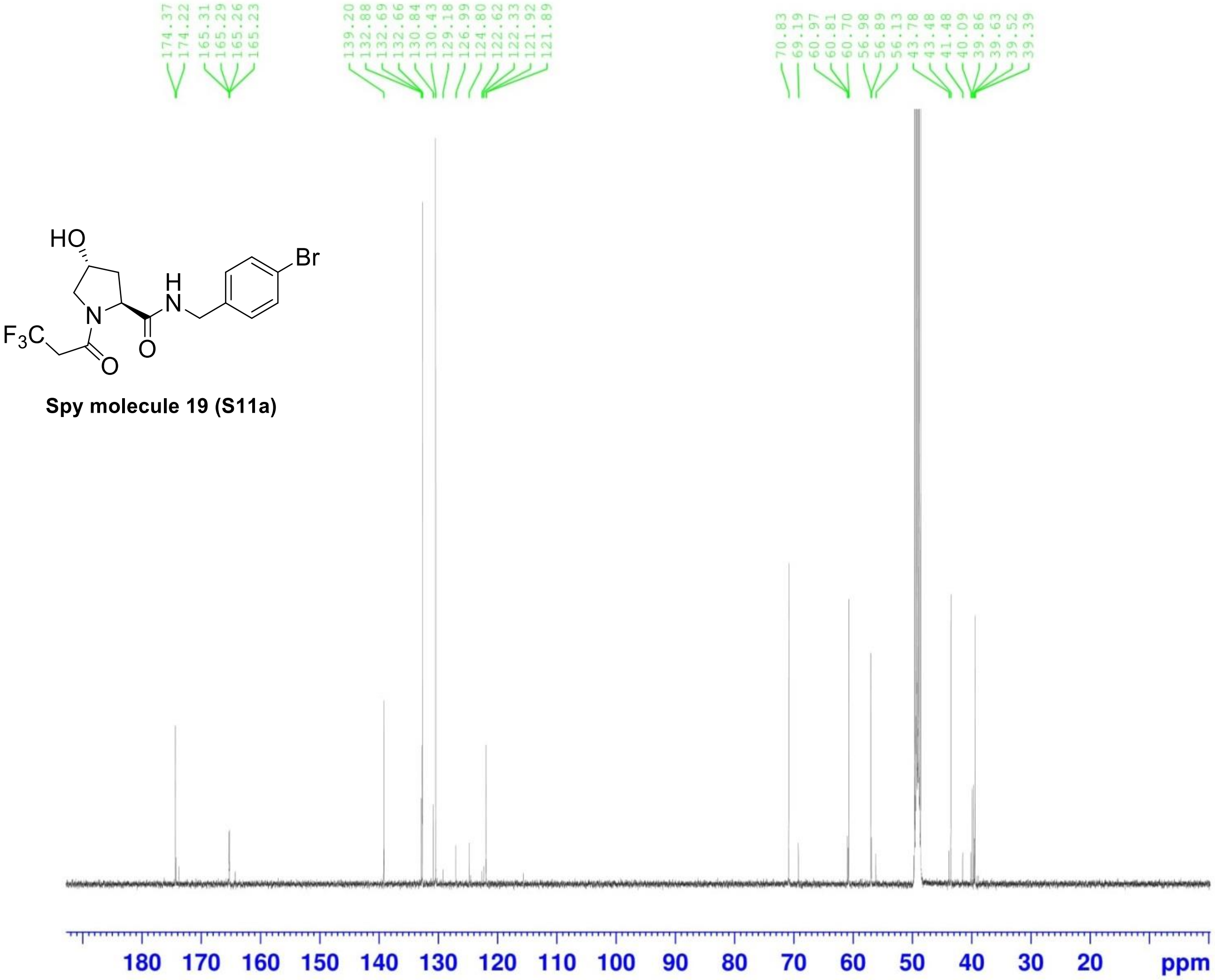

Spy molecule 19 (S11a)

```
Current Data Parameters
NAME                GCF18
EXPNO                 165
PROCNO                  1

F2 - Acquisition Parameters
Date_            20181114
Time                 4.32
INSTRUM             spect
PROBHD   5 mm CPQCI 1H/
PULPROG            zgpg30
TD                  65400
SOLVENT              MeOD
NS                   2048
DS                      4
SWH             29761.904 Hz
FIDRES           0.455075 Hz
AQ              1.0987200 sec
RG                 190.78
DW                 16.800 usec
DE                  18.00 usec
TE                  293.2 K
D1             2.00000000 sec
D11            0.03000000 sec
TD0                     1

======== CHANNEL f1 ========
SFO1          125.7703637 MHz
NUC1                  13C
P1                  12.00 usec
PLW1         133.00000000 W

======== CHANNEL f2 ========
SFO2          500.1320005 MHz
NUC2                   1H
CPDPRG[2          waltz16
PCPD2              100.00 usec
PLW2           5.19999981 W
PLW12          0.06406900 W
PLW13          0.06406900 W

F2 - Processing parameters
SI                 131072
SF            125.7575999 MHz
WDW                    EM
SSB      0
LB                   1.00 Hz
GB       0
PC                   1.40
```

Spy molecule 19 (Compound S11a)

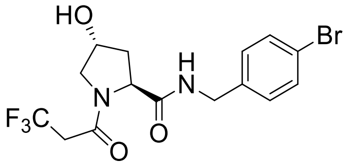

Spy molecule 19 (S11a)

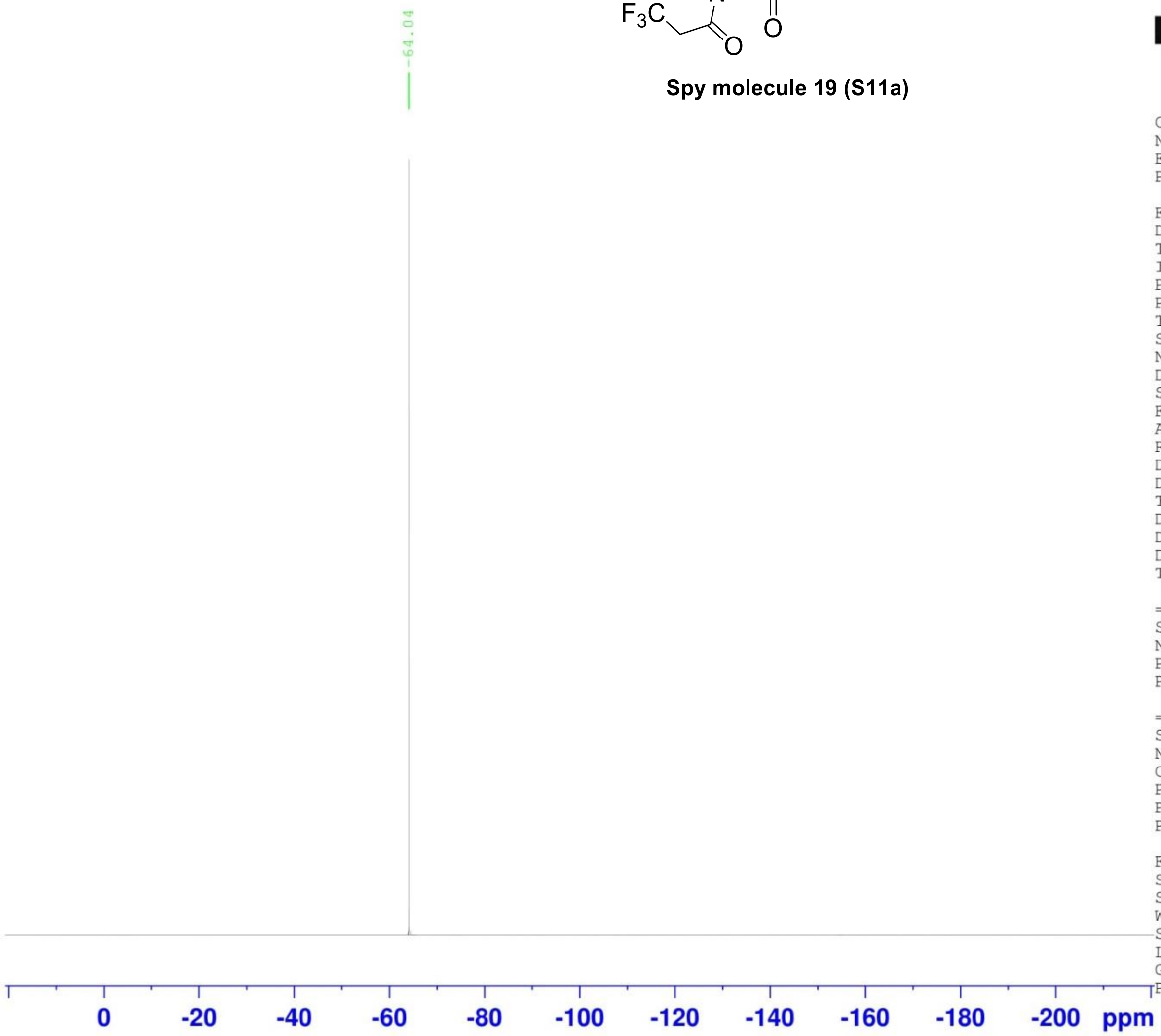


```
Current Data Parameters
NAME                 GCF18
EXPNO                  160
PROCNO                   1

F2 - Acquisition Parameters
Date_             20181114
Time                  2.34
INSTRUM              spect
PROBHD   5 mm CPQCI 1H/
PULPROG        zgfhigqn.2
TD                  131072
SOLVENT               MeOD
NS                     256
DS                       4
SWH            113636.367 Hz
FIDRES           0.866977 Hz
AQ              0.5767168 sec
RG                  190.78
DW                   4.400 usec
DE                   18.00 usec
TE                   293.1 K
D1              1.00000000 sec
D11             0.03000000 sec
D12             0.00002000 sec
TD0                      1

======== CHANNEL f1 ========
SFO1          470.5453180 MHz
NUC1                   19F
P1                   12.00 usec
PLW1            5.19999981 W

======== CHANNEL f2 ========
SFO2          500.1320005 MHz
NUC2                    1H
CPDPRG[2           waltz16
PCPD2               100.00 usec
PLW2            5.19999981 W
PLW12           0.06406900 W

F2 - Processing parameters
SI                  262144
SF            470.5923772 MHz
WDW                     EM
SSB      0
LB                    1.50 Hz
GB       0
PC                    1.00
```

Spy molecule 20 (Compound S11b)

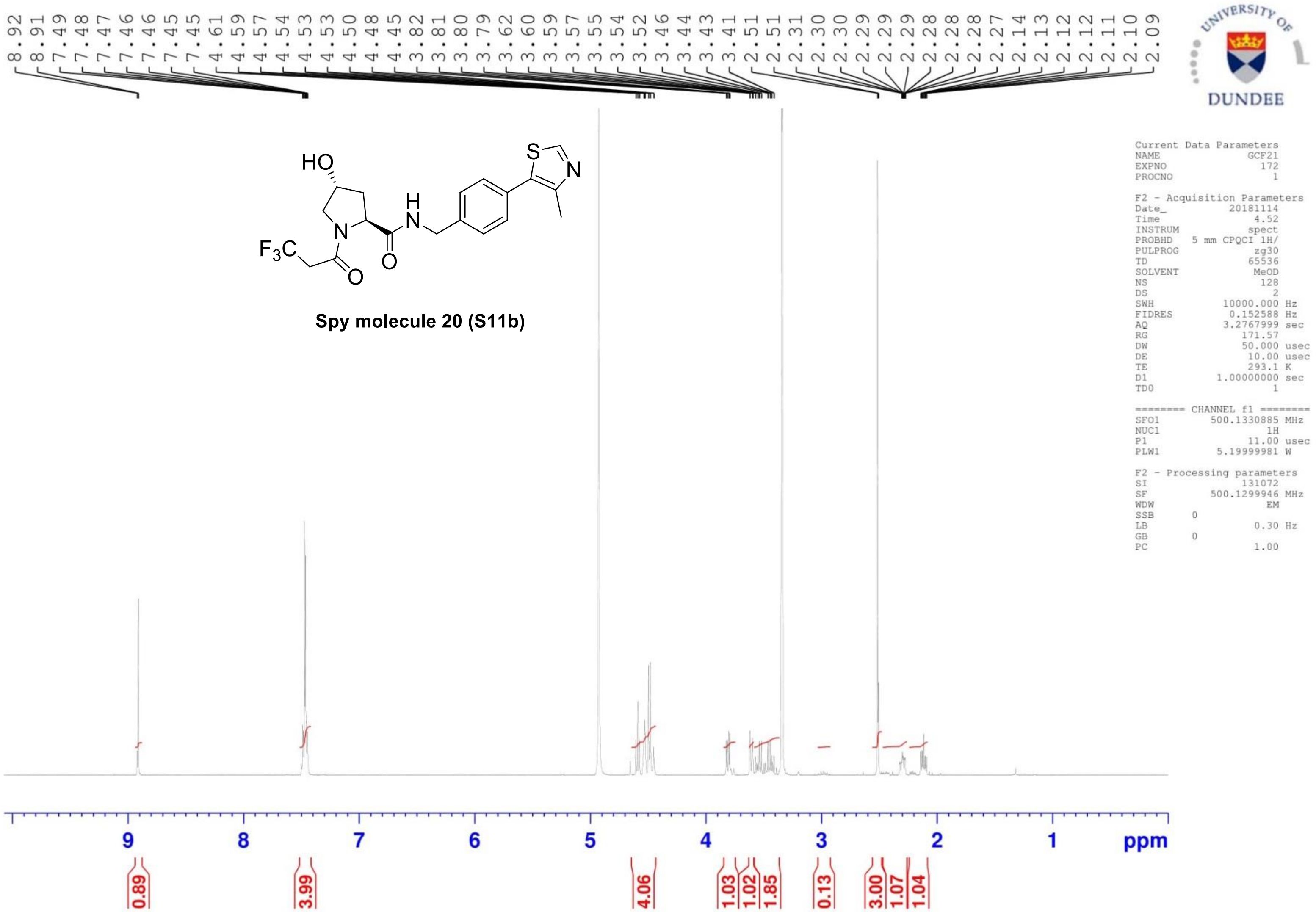

Spy molecule 20 (Compound S11b)

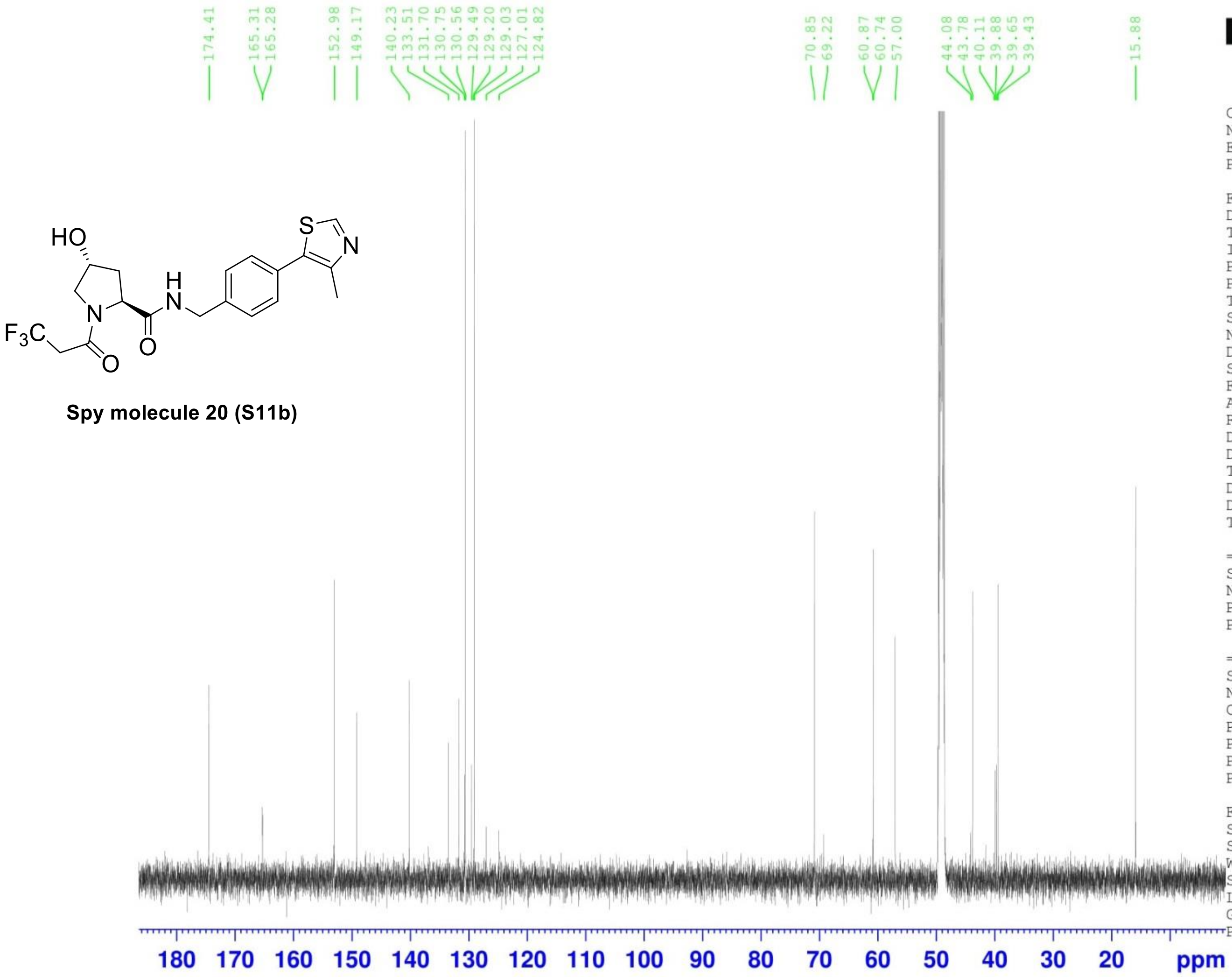

Spy molecule 20 (S11b)

```
Current Data Parameters
NAME                  GCF21
EXPNO                   174
PROCNO                    1

F2 - Acquisition Parameters
Date_              20181114
Time                   6.40
INSTRUM               spect
PROBHD   5 mm CPQCI 1H/
PULPROG              zgpg30
TD                    65400
SOLVENT                MeOD
NS                     2048
DS                        4
SWH              29761.904 Hz
FIDRES            0.455075 Hz
AQ               1.0987200 sec
RG                   190.78
DW                   16.800 usec
DE                    18.00 usec
TE                    293.2 K
D1              2.00000000 sec
D11             0.03000000 sec
TD0                       1

======== CHANNEL f1 ========
SFO1           125.7703637 MHz
NUC1                    13C
P1                    12.00 usec
PLW1          133.00000000 W

======== CHANNEL f2 ========
SFO2           500.1320005 MHz
NUC2                     1H
CPDPRG[2            waltz16
PCPD2                100.00 usec
PLW2             5.19999981 W
PLW12            0.06406900 W
PLW13            0.06406900 W

F2 - Processing parameters
SI                   131072
SF              125.7575992 MHz
WDW                      EM
SSB      0
LB                     1.00 Hz
GB       0
PC                     1.40
```

Spy molecule 20 (Compound S11b)

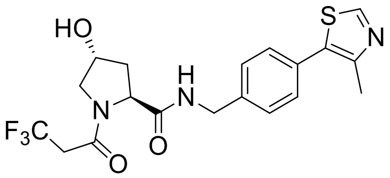

Spy molecule 20 (S11b)

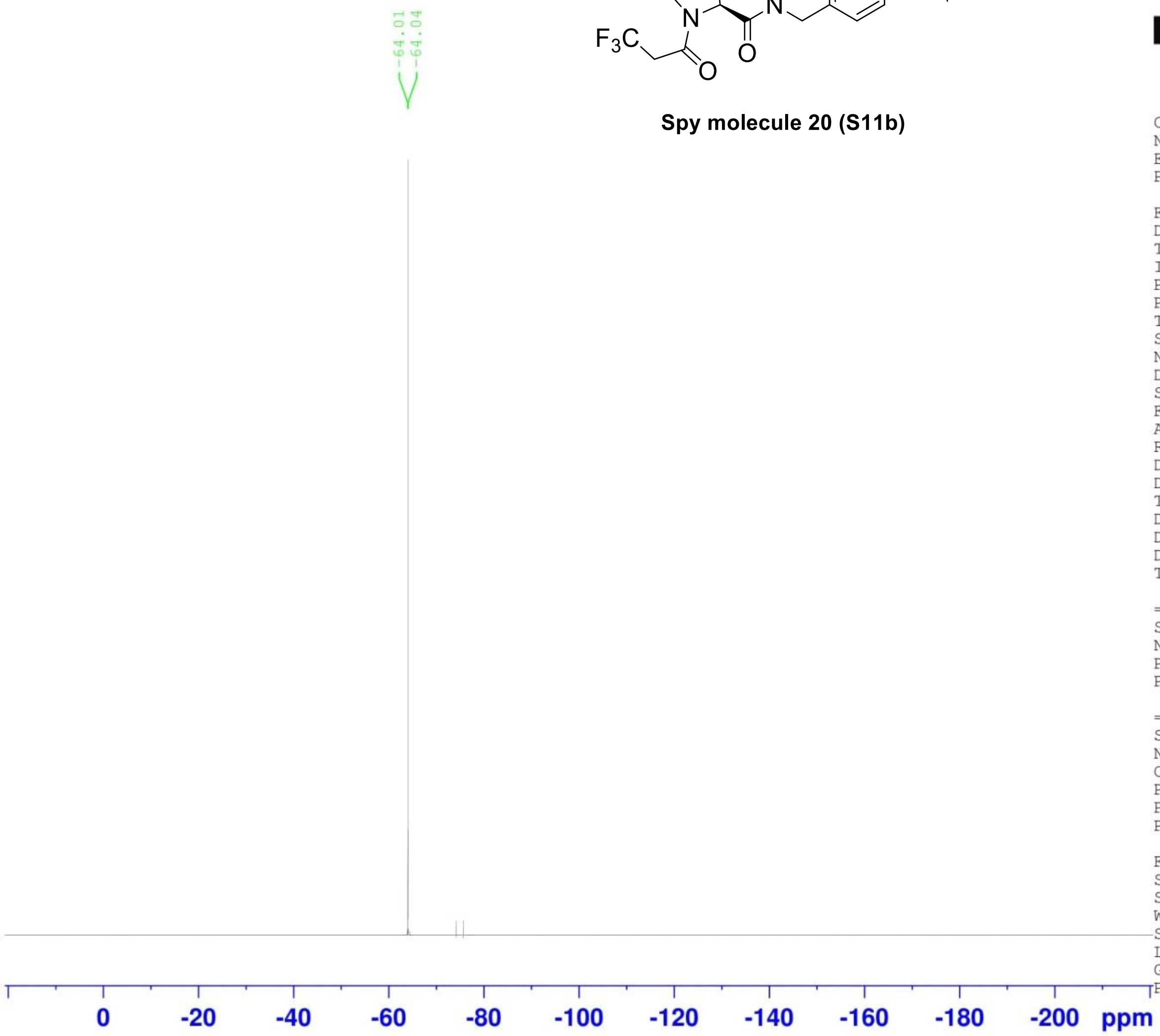


```
Current Data Parameters
NAME                GCF21
EXPNO                 170
PROCNO                  1

F2 - Acquisition Parameters
Date_            20181114
Time                 4.42
INSTRUM             spect
PROBHD   5 mm CPQCI 1H/
PULPROG       zgfhigqn.2
TD                 131072
SOLVENT              MeOD
NS                    256
DS                      4
SWH            113636.367 Hz
FIDRES           0.866977 Hz
AQ              0.5767168 sec
RG                 190.78
DW                  4.400 usec
DE                  18.00 usec
TE                  293.1 K
D1             1.00000000 sec
D11            0.03000000 sec
D12            0.00002000 sec
TD0                     1

======== CHANNEL f1 ========
SFO1         470.5453180 MHz
NUC1                  19F
P1                  12.00 usec
PLW1           5.19999981 W

======== CHANNEL f2 ========
SFO2         500.1320005 MHz
NUC2                   1H
CPDPRG[2          waltz16
PCPD2              100.00 usec
PLW2           5.19999981 W
PLW12          0.06406900 W

F2 - Processing parameters
SI                 262144
SF           470.5923772 MHz
WDW                    EM
SSB      0
LB                   1.50 Hz
GB       0
PC                   1.00
```

Spy molecule 21 (Compound S12a)

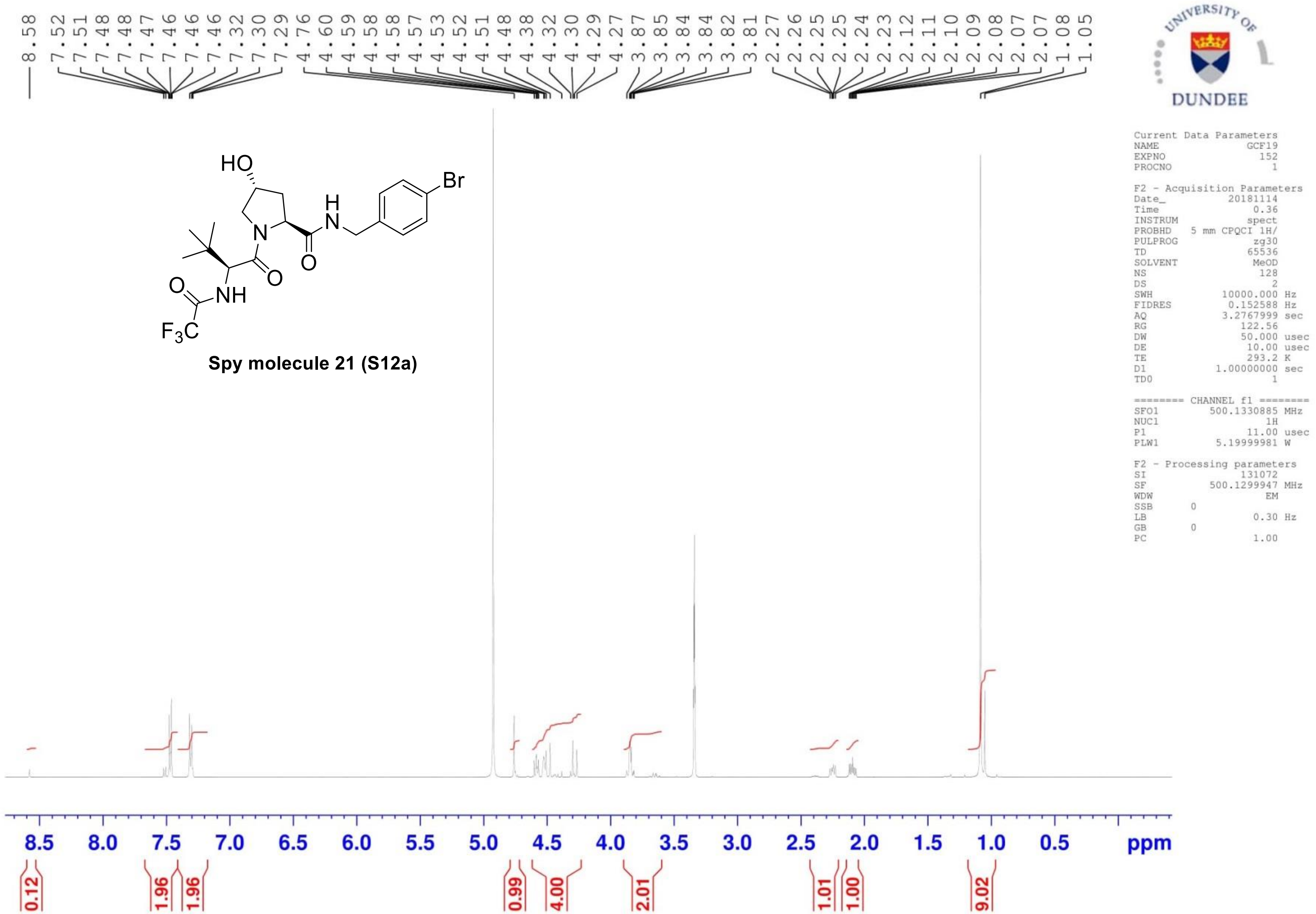

Spy molecule 21 (Compound S12a)

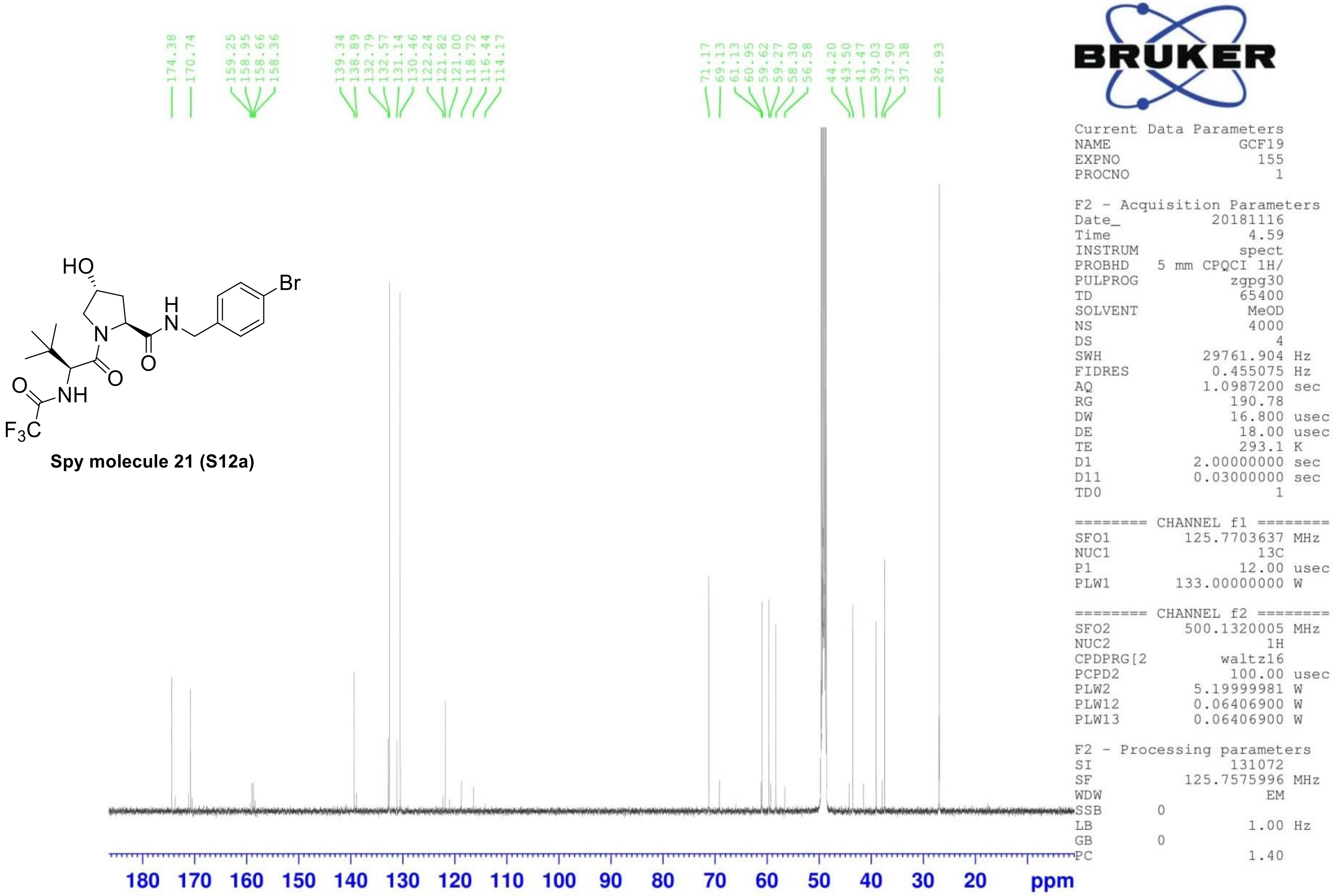

Spy molecule 21 (S12a)

Spy molecule 21 (Compound S12a)

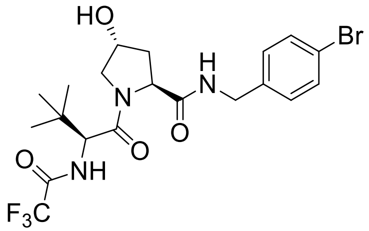

Spy molecule 21 (S12a)

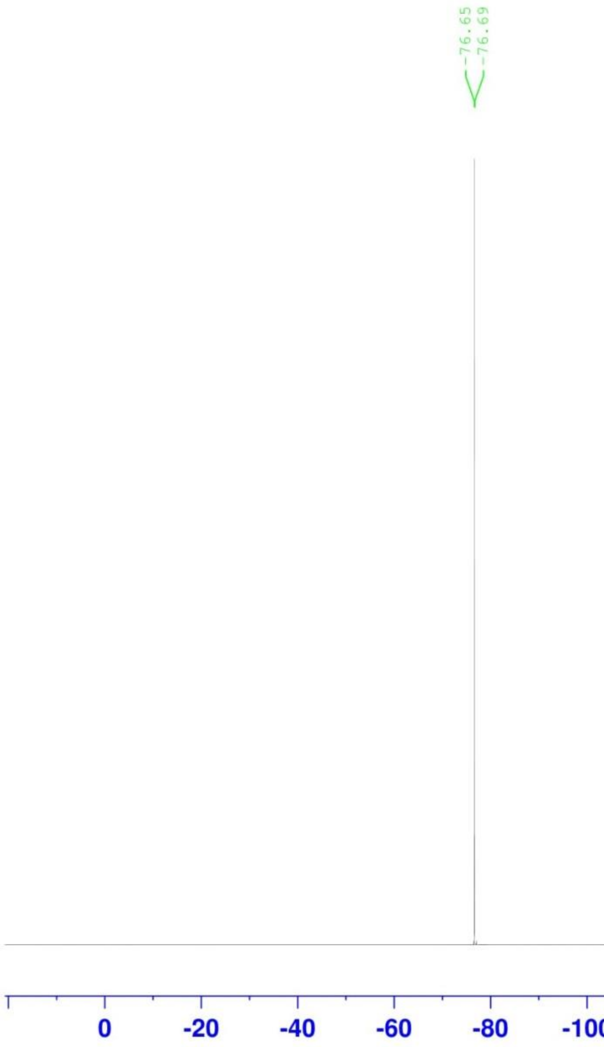


```
Current Data Parameters
NAME                GCF19
EXPNO                 150
PROCNO                  1

F2 - Acquisition Parameters
Date_            20181114
Time                 0.26
INSTRUM             spect
PROBHD   5 mm CPQCI 1H/
PULPROG        zgfhigqn.2
TD                 131072
SOLVENT              MeOD
NS                    256
DS                      4
SWH            113636.367 Hz
FIDRES           0.866977 Hz
AQ              0.5767168 sec
RG                 190.78
DW                  4.400 usec
DE                  18.00 usec
TE                  293.1 K
D1             1.00000000 sec
D11            0.03000000 sec
D12            0.00002000 sec
TD0                     1

======== CHANNEL f1 ========
SFO1         470.5453180 MHz
NUC1                 19F
P1                 12.00 usec
PLW1          5.19999981 W

======== CHANNEL f2 ========
SFO2         500.1320005 MHz
NUC2                  1H
CPDPRG[2         waltz16
PCPD2             100.00 usec
PLW2          5.19999981 W
PLW12         0.06406900 W

F2 - Processing parameters
SI                262144
SF           470.5923772 MHz
WDW                   EM
SSB      0
LB                  1.50 Hz
GB       0
PC                  1.00
```

Spy molecule 22 (Compound S12b)

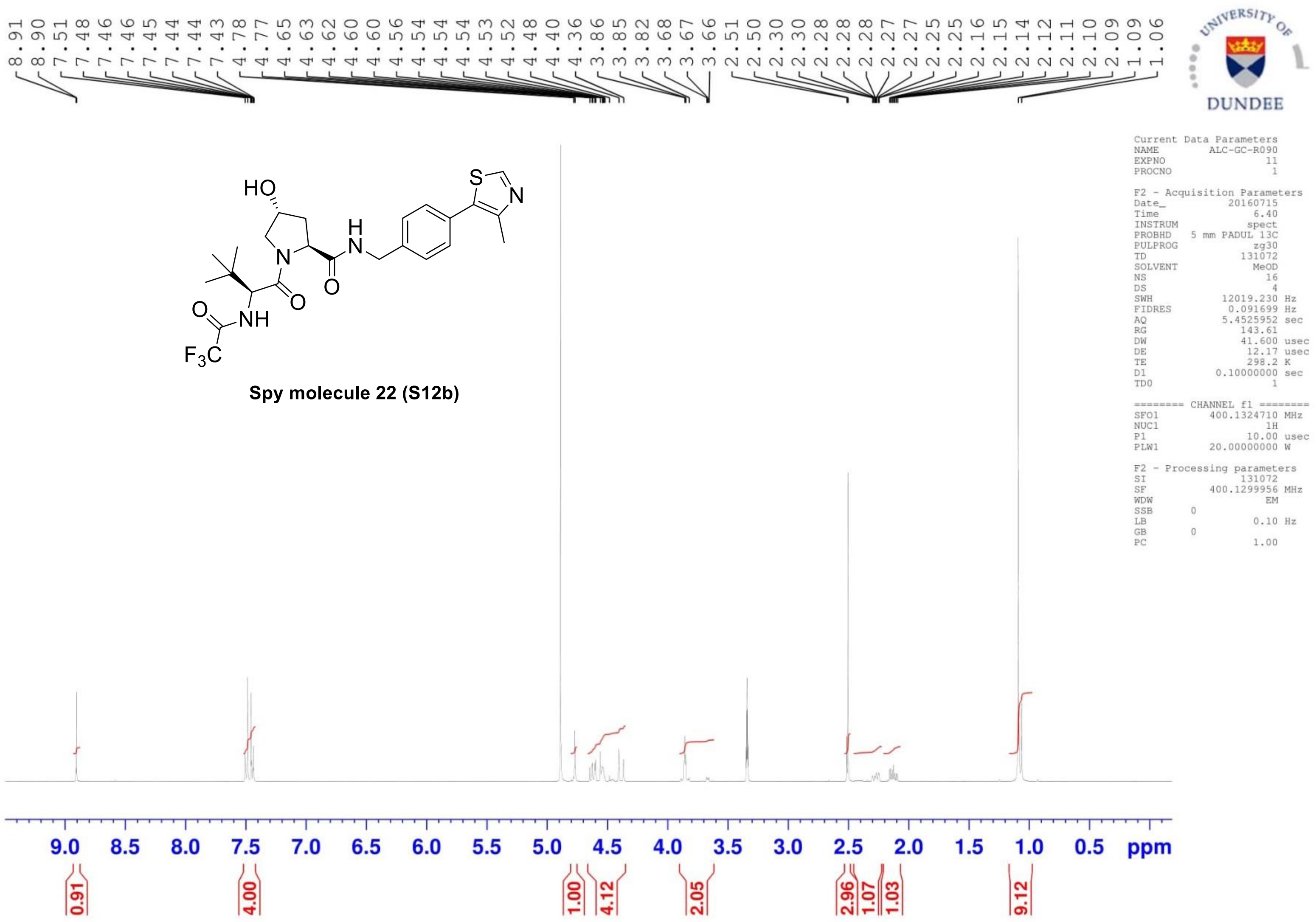

Spy molecule 22 (S12b)

Spy molecule 22 (Compound S12b)

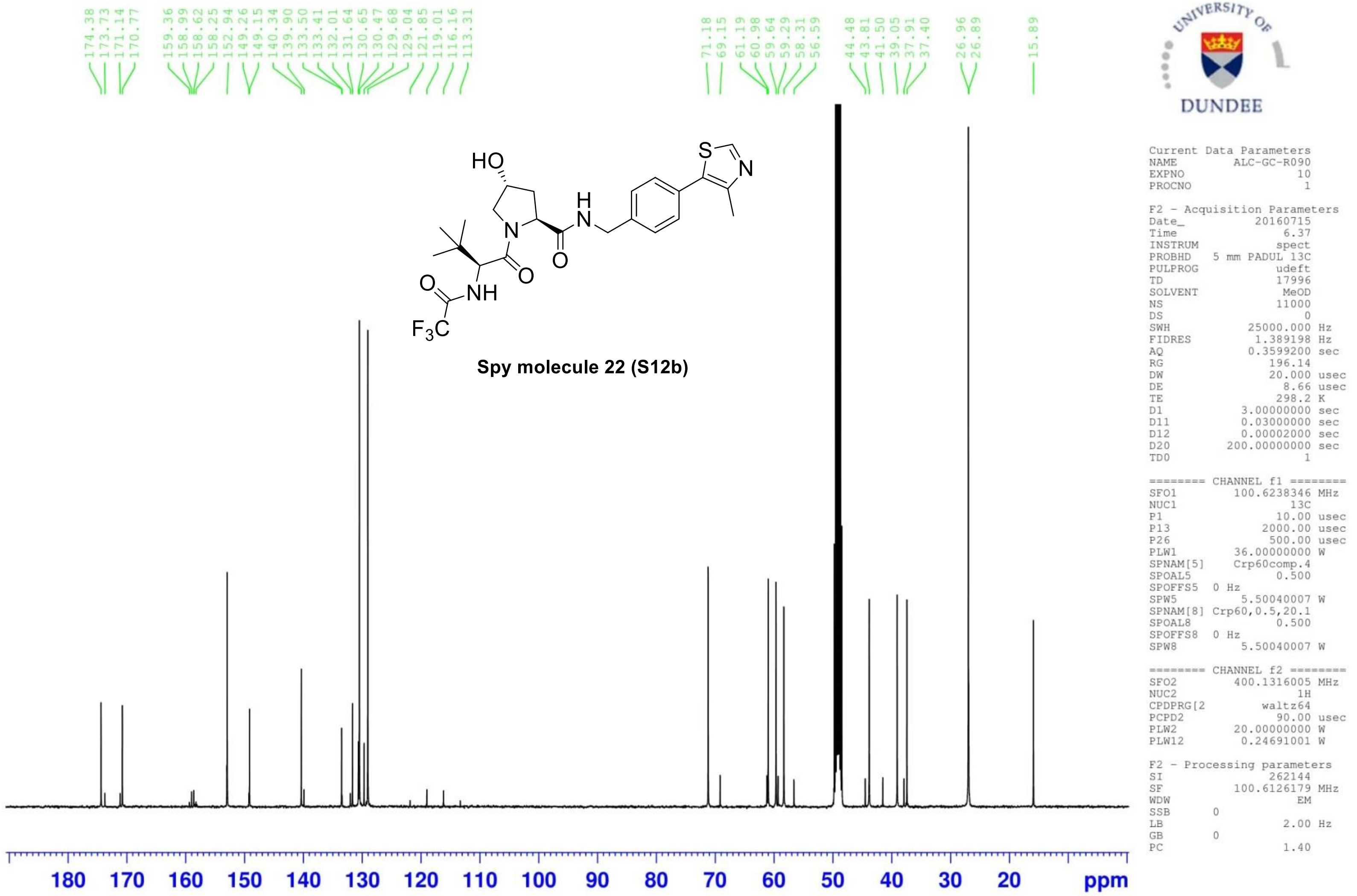

Spy molecule 22 (S12b)

Spy molecule 22 (Compound S12b)

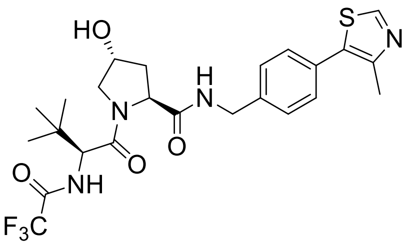

Spy molecule 22 (S12b)

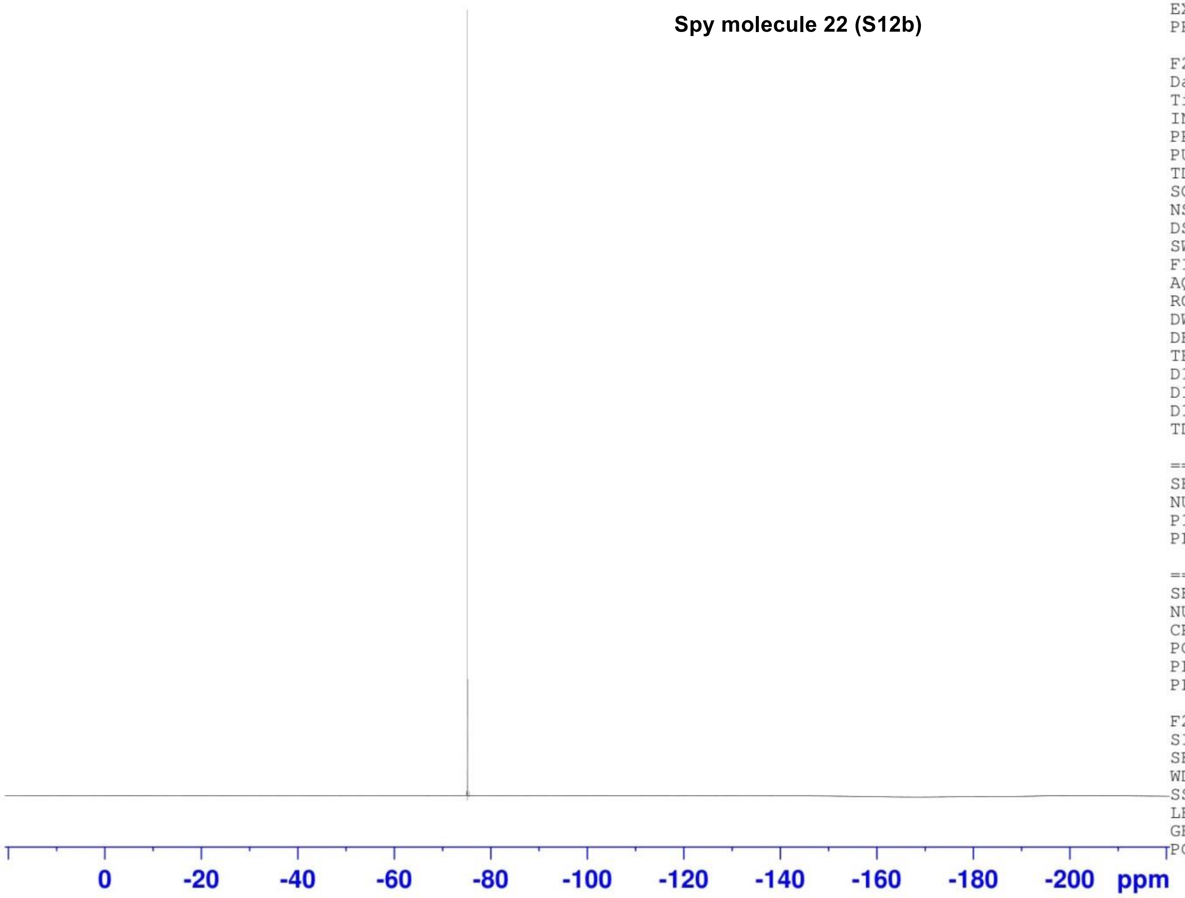


```
Current Data Parameters
NAME              AC-GC-R090
EXPNO                      2
PROCNO                     1

F2 - Acquisition Parameters
Date_               20160717
Time                   14.34
INSTRUM                spect
PROBHD      5 mm QNP 1H/13
PULPROG          zgfhigqn.2
TD                    131072
SOLVENT                 MeOD
NS                        16
DS                         4
SWH               113636.367 Hz
FIDRES              0.866977 Hz
AQ                 0.5767168 sec
RG                       362
DW                     4.400 usec
DE                      6.50 usec
TE                     298.2 K
D1                1.00000000 sec
D11               0.03000000 sec
D12               0.00002000 sec
TD0                        1

======== CHANNEL f1 ========
SFO1             470.5453180 MHz
NUC1                     19F
P1                     13.50 usec
PLW1             25.00000000 W

======== CHANNEL f2 ========
SFO2             500.1320005 MHz
NUC2                      1H
CPDPRG[2             waltz16
PCPD2                  80.00 usec
PLW2             25.00000000 W
PLW12             0.39063001 W

F2 - Processing parameters
SI                     65536
SF               470.5923772 MHz
WDW                       EM
SSB      0
LB                      0.30 Hz
GB       0
PC                      1.00
```

10. References

[1] X. Lucas, I. Van Molle, A. Ciulli, *J. Med. Chem.* **2018**, *61*, 7387-7393.

[2] C. Galdeano, M. S. Gadd, P. Soares, S. Scaffidi, I. Van Molle, I. Birced, S. Hewitt, D. M. Dias, A. Ciulli, *J. Med. Chem.* **2014**, *57*, 8657-8663.

[3] a) T. Kamura, D. M. Koepp, M. N. Conrad, D. Skowyra, R. J. Moreland, O. Iliopoulos, W. S. Lane, W. G. Kaelin, Jr., S. J. Elledge, R. C. Conaway, J. W. Harper, J. W. Conaway, *Science* **1999**, *284*, 657-661; b) A. Pause, S. Lee, R. A. Worrell, D. Y. Chen, W. H. Burgess, W. M. Linehan, R. D. Klausner, *Proc. Natl. Acad. Sci. U.S.A* **1997**, *94*, 2156-2161.

[4] C. E. Stebbins, W. G. Kaelin, Jr., N. P. Pavletich, *Science* **1999**, *284*, 455-461.

[5] T. A. F. Cardote, M. S. Gadd, A. Ciulli, *Structure* **2017**, *25*, 901-911 e903.

[6] M. Fairhead, M. Howarth, *Methods Mol. Biol.* **2015**, *1266*, 171-184.

[7] C. Dalvit, *Prog. Nucl. Magn. Reson. Spectrosc.* **2007**, *51*, 243-I.

[8] R. Buratto, D. Mammoli, E. Chiarparin, G. Williams, G. Bodenhausen, *Angew. Chem., Int. Ed.* **2014**, *53*, 11376-11380.

[9] a) R. Z. Cer, U. Mudunuri, R. Stephens, F. J. Lebeda, *Nucleic Acids Res.* **2009**, *37*, W441-W445; b) Z. Nikolovska-Coleska, R. Wang, X. Fang, H. Pan, Y. Tomita, P. Li, P. P. Roller, K. Krajewski, N. G. Saito, J. A. Stuckey, S. Wang, *Anal. Biochem.* **2004**, *332*, 261-273.
